# Supplementary material for: General alkyl fluoride functionalization via short-lived carbocation-organozincate ion pairs
Source: Nat Commun. 2024 Feb 29;15:1866. doi: 10.1038/s41467-024-45756-4 (PMC10904780; doi:10.1038/s41467-024-45756-4)
Supplement: Supplementary file 1 — Supplementary Information [file 41467_2024_45756_MOESM1_ESM.pdf]

Supplementary Information

General Alkyl Fluoride Functionalization via Short-lived Carbocation-Organozincate Ion Pairs

D. Lucas Kane, Bryan C. Figula, Kaluvu Balaraman, Jeffery A. Bertke and Christian Wolf*
Georgetown University, Chemistry Department, Washington, DC 20057, USA

Table of Contents

1. General Information S2

2. Mechanistic Studies S3

2.1. Initial Studies of the Structures, Properties and Reactivities of Organozinc Compounds S3

2.2. Isolation and Characterization of a Supramolecular Zinc Cluster Intermediate S10

2.3. Control Experiments S15

2.4. Alkyl Chain Rearrangement Studies S22

2.5. C-C Bond Formation with Enantiomerically Enriched Alkyl Fluorides S26

2.6 Selectivity for C(sp3)-F Bonds S29

3. Reaction Optimization S30

3.1. Screening of Reaction Conditions for Representative 1°, 2°, and 3° Alkyl Fluorides S30

3.2. Screening of C-F Bond Functionalization with Various Organometallic Reagents S32

4. General C-F Bond Arylation Procedures S33

5. Product Synthesis and Characterization S34

5.1. Synthesis of Diarylzinc Reagents S34

5.2. Synthesis of Alkyl Fluorides S36

5.3. C-F Bond Functionalization with Diphenylzinc S38

5.4. C-F Bond Functionalization with Other Diarylzinc Reagents S51

6. ^{1}H, ^{13}C and ^{19}F NMR Spectra S63

7. X-Ray Crystallography S142

8. References S156

1. General Information

Commercially available organofluorines, diphenylzinc, and bis(pentafluorophenyl)zinc were used as purchased without further purification. Solvents were stored over 4Å molecular sieves prior to use. All reaction products were purified by column chromatography on silica gel (particle size 40-63 μm) as described below. Air sensitive reactions were carried out in a nitrogen-filled glovebox or with standard Schlenk techniques. NMR spectra were obtained at 400 MHz (^{1}H NMR), 100 MHz (^{13}C NMR) and 376 MHz (^{19}F NMR) and chemical shifts were referenced to residual solvent peaks. GC-MS measurements were acquired on an Agilent 5977C GC/MSD equipped with an HP-5ms Ultra Inert (5%-phenyl)-methylpolysiloxane column (30 m, 0.25 mm, 0.25 μm). HRMS data were obtained using electron spray ionization time-of-flight (ESI-TOF) spectrometry. Single crystals of each compound were mounted under parabar oil on a Mitegen micromount and immediately placed in a cold nitrogen stream at 100(2) K prior to data collection. Data were collected on either a Bruker D8 Quest equipped with a Photon100 CMOS detector and a Mo ImS source or a Bruker DUO equipped with an APEXII CCD detector and Mo fine-focus sealed source. Data were integrated with the Bruker SAINT program. Structure solution and refinement was performed using the SHELXTL/PC suite[1] and ShelXle.[2] Intensities were corrected for Lorentz and polarization effects and an empirical absorption correction was applied using Blessing's method as incorporated into the program SADABS.[3] Non-hydrogen atoms were refined with anisotropic thermal parameters. Hydrogen atoms were included in idealized positions unless otherwise noted.

2. Mechanistic Studies

2.1. Initial Studies of the Structures, Properties and Reactivities of Organozinc Compounds

Formation and X-ray characterization of a diphenylzinc trimer

In a nitrogen-filled glovebox, diphenylzinc (200 mg, 0.91 mmol) was dissolved in a minimal amount of toluene (4.0 mL), filtered through a 0.2 μm PVDF syringe filter, and the resulting solution was allowed to stand at -40 °C overnight. Colorless crystals of the trimer were collected, in addition to crystals of the known dinuclear species.[4]

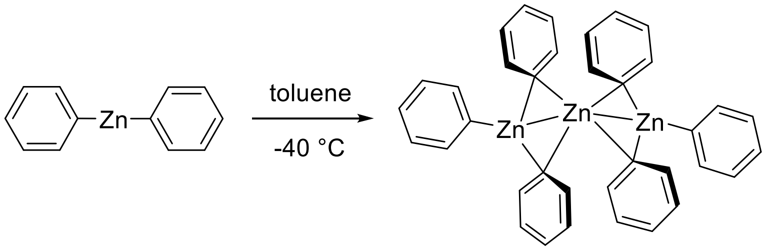

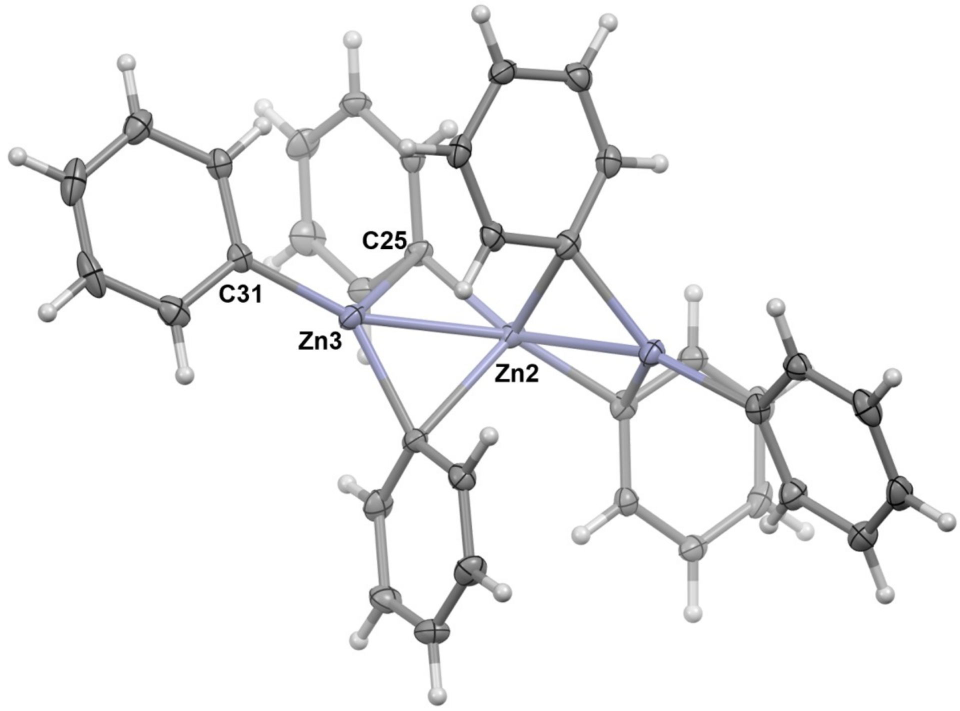

Supplementary Fig. 1. X-ray structure of $[Ph_2Zn]_3$.
Thermal ellipsoids are displayed at the 50% probability level. Zn3-C31 1.948(3) Å, Zn3-Zn2 2.5845(7) Å, Zn3-C25 2.376(3) Å, Zn2-C25 2.018(3) Å, Zn3-Zn2-Zn1 117.312°(19), Zn1-C1 1.951(3) Å, Zn1-C7 2.024(3) Å, Zn1-C13 2.351(3) Å.

Crystal growth and X-ray characterization of Fc_2Zn

Single crystals were grown from a saturated pentane solution under inert atmosphere at -40 °C.

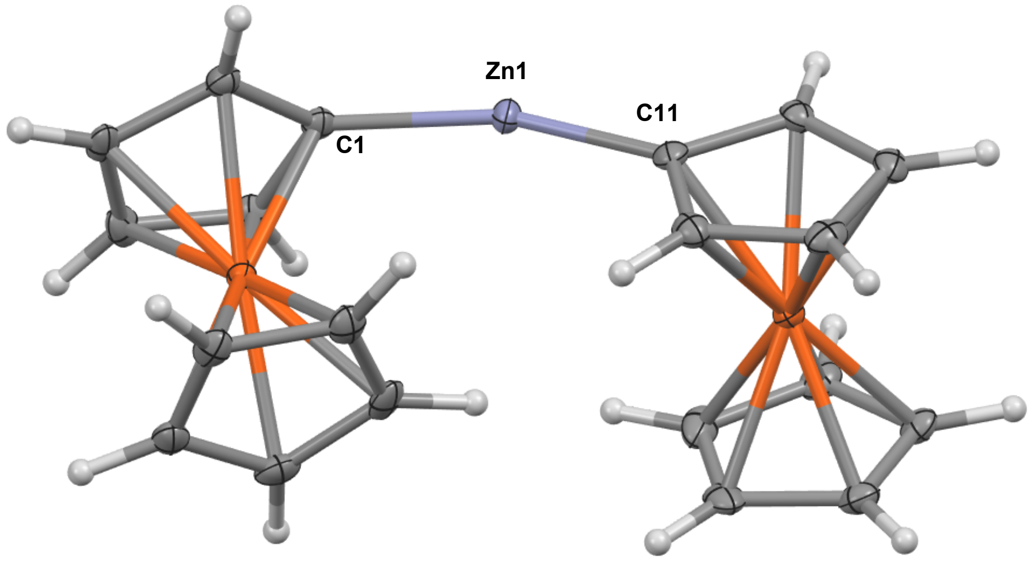

Supplementary Fig. 2. X-ray structure of diferrocenylzinc.
One molecule of Fc_2Zn among two crystallographically distinct species is shown for clarity. Thermal ellipsoids are displayed at the 50% probability level. Select bond distances: Zn1-C1 1.950(3) Å, Zn11-C1 1.949(3) Å, C11-Zn1-C1 157.41°(11), Fe1-C1 2.092(3) Å, C1-C2 1.449(4) Å, C2-C1-Zn1 128.2°(2).

Isolation and characterization of ion pair 1

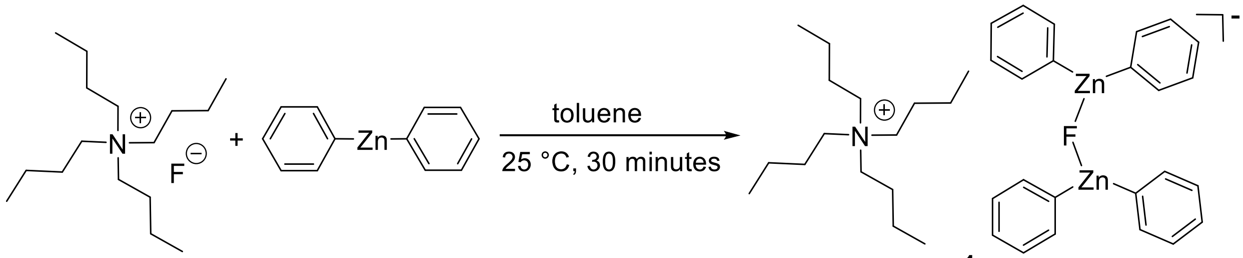

1

In a nitrogen-filled glovebox, a tetrabutylammonium fluoride (TBAF) solution in THF (120 µL, 0.12 mmol) was evaporated under reduced pressure to remove the solvent. The resulting residue

was redissolved in toluene (1.0 mL). Diphenylzinc (52 mg, 0.24 mmol) was added, and the mixture was stirred for 30 minutes at room temperature affording ion pair **1**. X-ray quality single crystals were obtained from a dichloroethane solution layered with one volume of pentane at -40 °C.

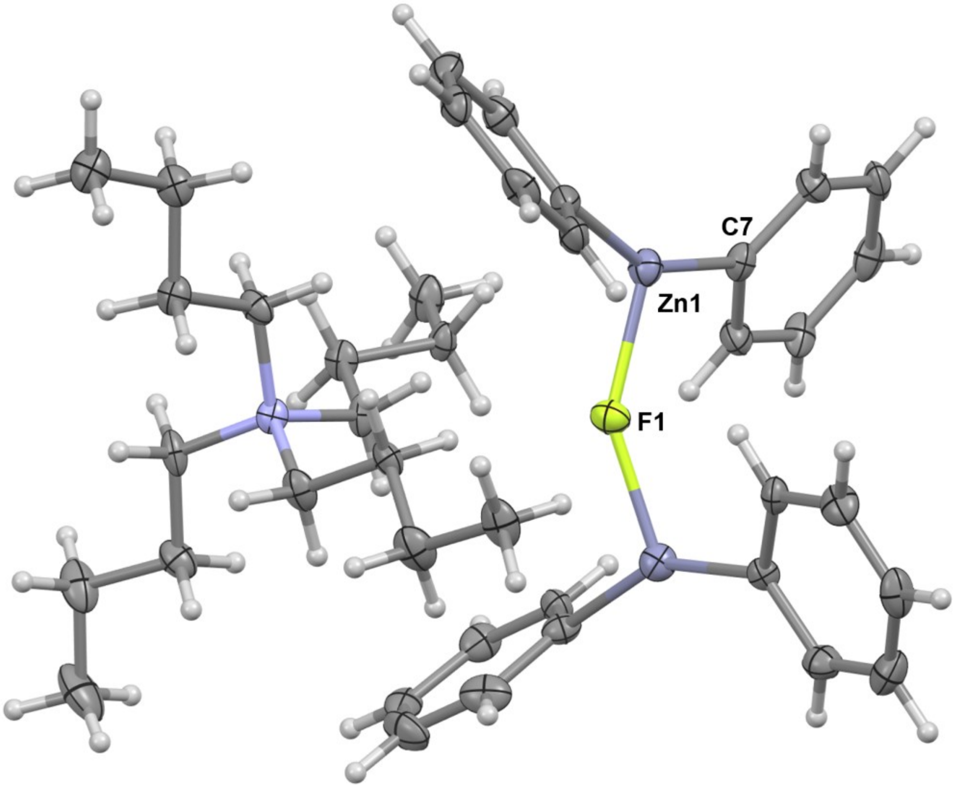

Supplementary Fig. 3. X-ray structure of **1**.
Thermal ellipsoids are displayed at the 50% probability level. Zn1-C7 1.955(5) Å, Zn1-C1 1.967(6) Å, Zn2-C13 1.969(6) Å, Zn2-C19 1.970(6) Å, Zn1-F1 1.990(3) Å, Zn2-F1 1.996(3) Å, C7-Zn1-C1 149.9°(2), C7-Zn1-F1 105.90°(19), Zn1-F1-Zn2 148.04°(18).

Characterization of an unsolvated lithium triarylzincate complex 2

In a nitrogen-filled glovebox, diphenylzinc (100 mg, 0.45 mmol) was dissolved in toluene (2 mL). Phenyl lithium (38.0 mg, 0.45 mmol) was added as solid, and the mixture was allowed to stir at room temperature for 30 minutes. The mixture was filtered through a 0.2 μm PVDF syringe filter and allowed to stand at room temperature, affording crystals of **2** in one hour.

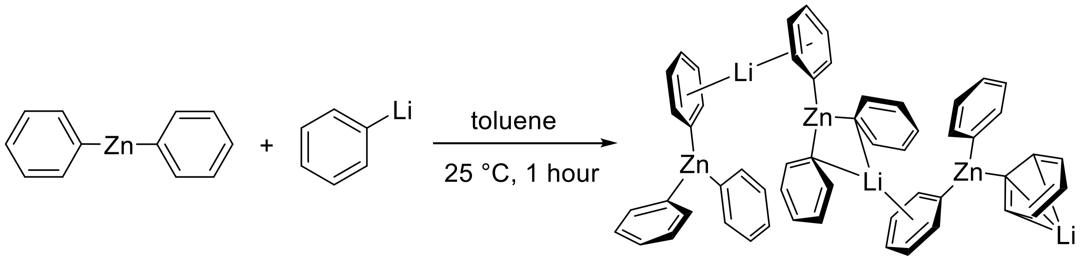

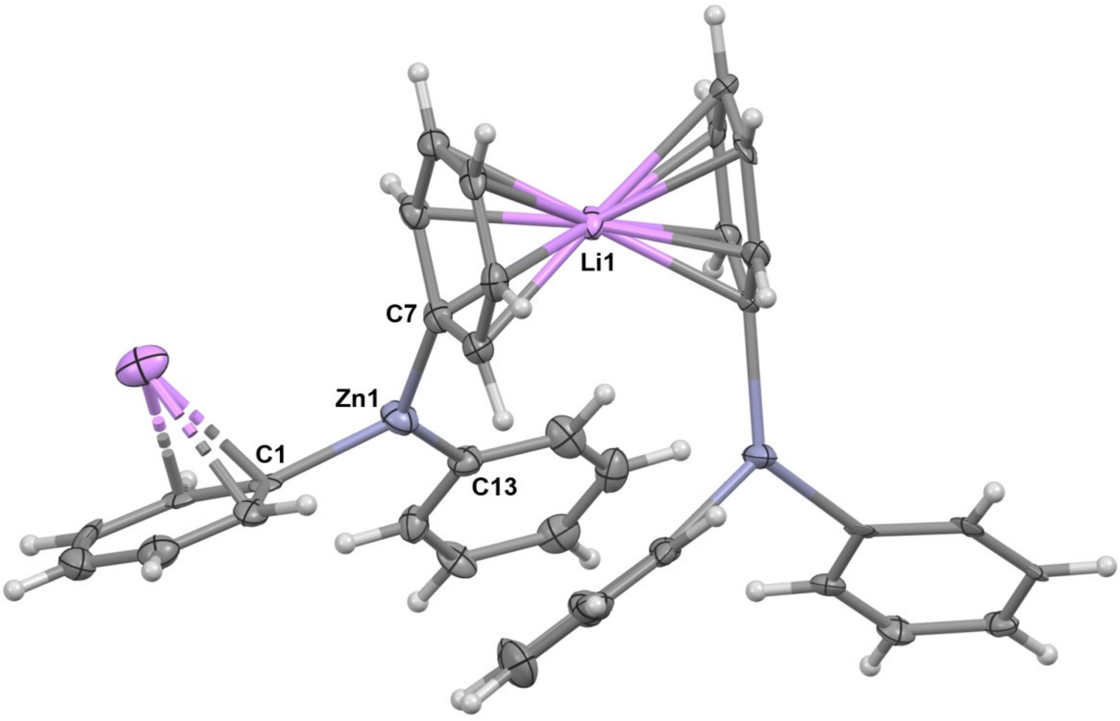

Supplementary Fig. 4. X-ray structure of **2**.

Thermal ellipsoids are displayed at the 50% probability level. An additional lithium triphenyl zincate species in the asymmetric unit has been omitted for clarity. Zn1-C1 2.03(1) Å, Zn1-C7 2.03(1) Å, Zn1-C13 1.98(1) Å, Zn2-C19 2.015(10) Å, Zn2-C31 2.036(11) Å, Zn2-C25 2.046(11) Å, Zn3-C49 1.998(11) Å, Zn3-C43 2.028(12) Å, Zn3-C37 2.037(11) Å, C13-Zn1-C7 120.2°(5), C13-Zn1-C1 133.5°(5), C7-Zn1-C1 106.3°(4).

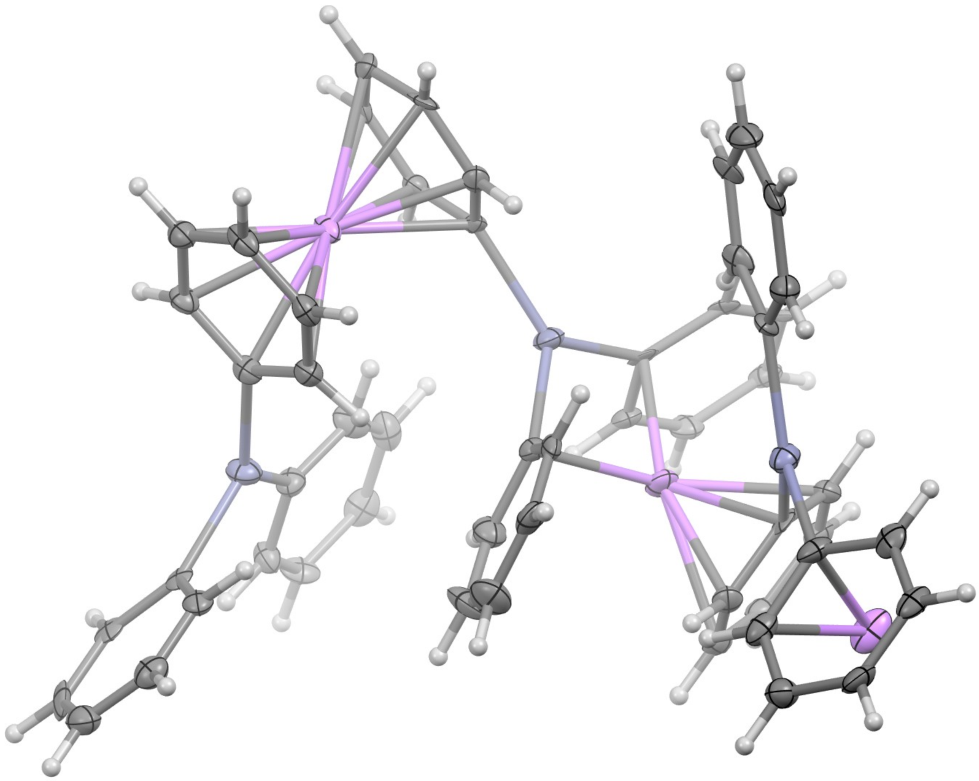

Supplementary Fig. 5. Complete asymmetric unit of the X-ray structure of **2**. Thermal ellipsoids are displayed at the 50% probability level.

Trityl arylation with activated phenylzincate species 1 or 2

In order to assess the reactivity of diarylzincate fluorides and triarylzincates, the aforementioned species **1** and **2** were treated with tritylium tetrafluoroborate, **3**, and the reactions were monitored by GC-MS. The μ_2-fluoride bridged diphenyl zincate **1** arylates poorly, affording the undesired hydrodefluorination compound **5** as the major product. The two products **4** and **5** were obtained in a 1:6 ratio. In contrast, lithium triphenylzincate is an efficient arylating agent, giving full conversion and outcompeting hydrodefluorination. The products **4** and **5** were formed in a 3:1 ratio.

In a nitrogen-filled glove box, a solution of the zincate (0.091 mmol) in the either toluene or deuterated chloroform (1.0 mL) was treated with tritylium tetrafluoroborate (0.091 mmol). The reactions were monitored by GC-MS until full conversion was reached.

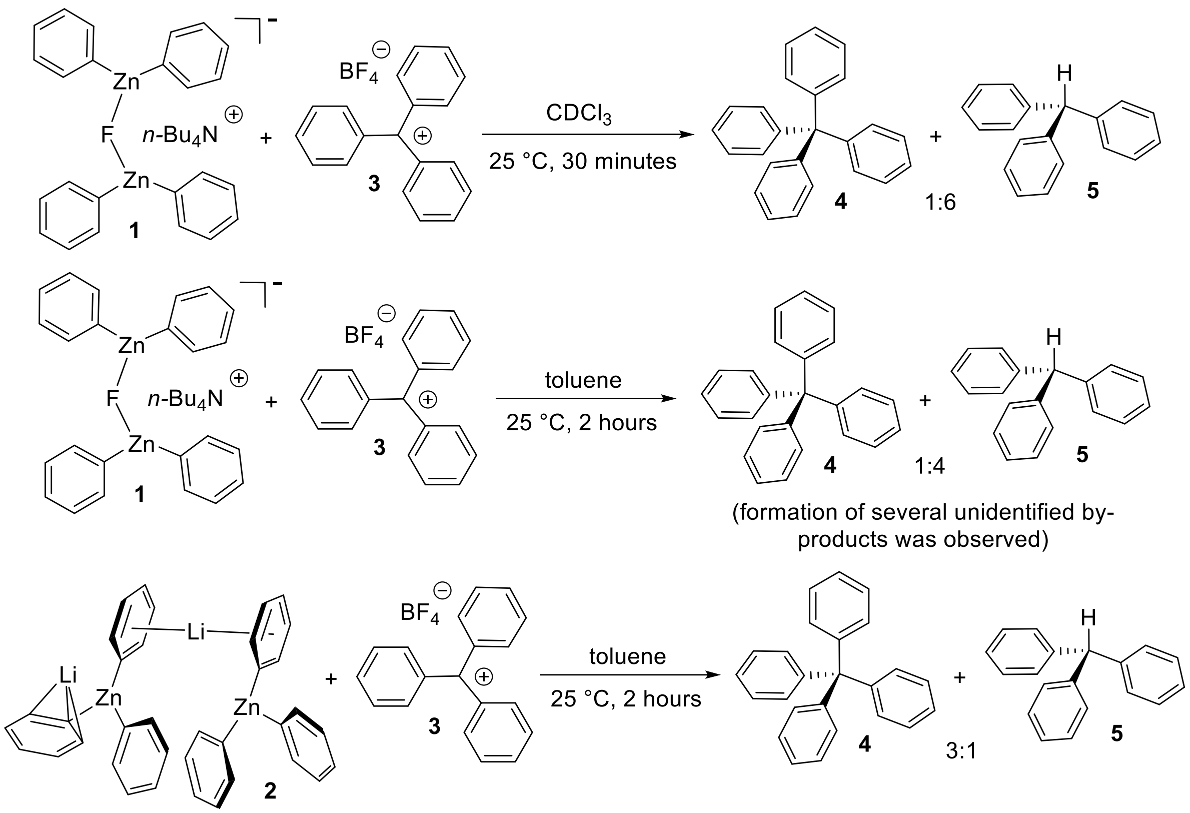

Supplementary Fig. 6. Initial testing of the phenylation of **3** using either **1** or **2** under similar conditions.

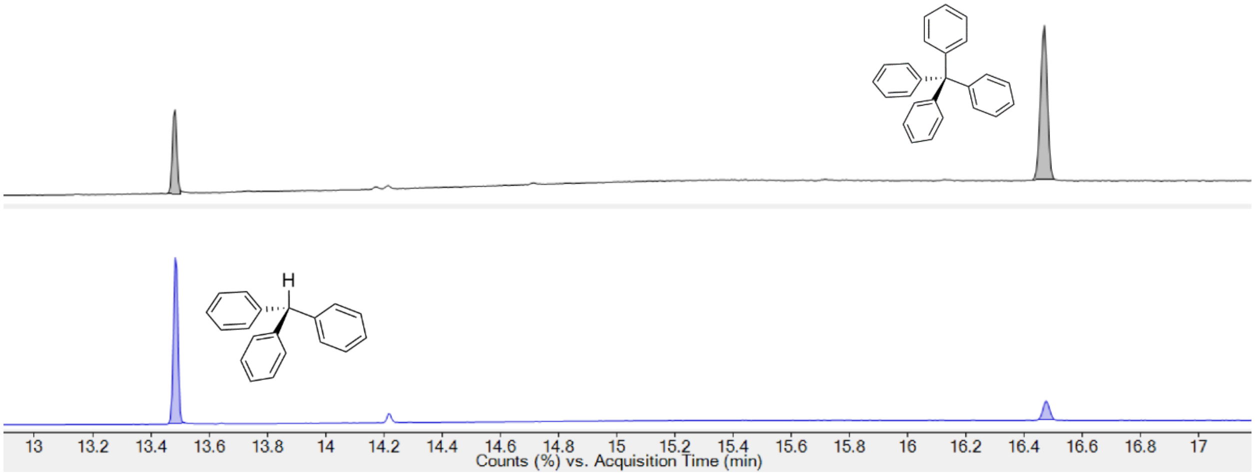

Supplementary Fig. 7. GC-MS analysis of the reaction between **1** and **2** with tritylium tetrafluoroborate, **3**. Top: The reaction with **2** gives **4** and **5** in a 3:1 ratio, favoring arylation over hydrodefluorination. Bottom: The reaction with **1** gives **4** and **5** in a 1:6 ratio, favoring hydrodefluorination over arylation.

Arylation vs fluorination competition experiment

The reaction of stoichiometric amounts of TBAF (0.091 mmol) and diphenylzinc (0.091 mmol) gives the μ_2-fluoride bridged diphenyl zincate **1**, with one equivalent of free TBAF remaining in the solution (Supplementary Figure 8). Treatment of this mixture with trityllium tetrafluoroborate **3** gives rise to near quantitative conversion to trityl fluoride showing that fluorination largely outcompetes C-C bond formation (Supplementary Figure 9).

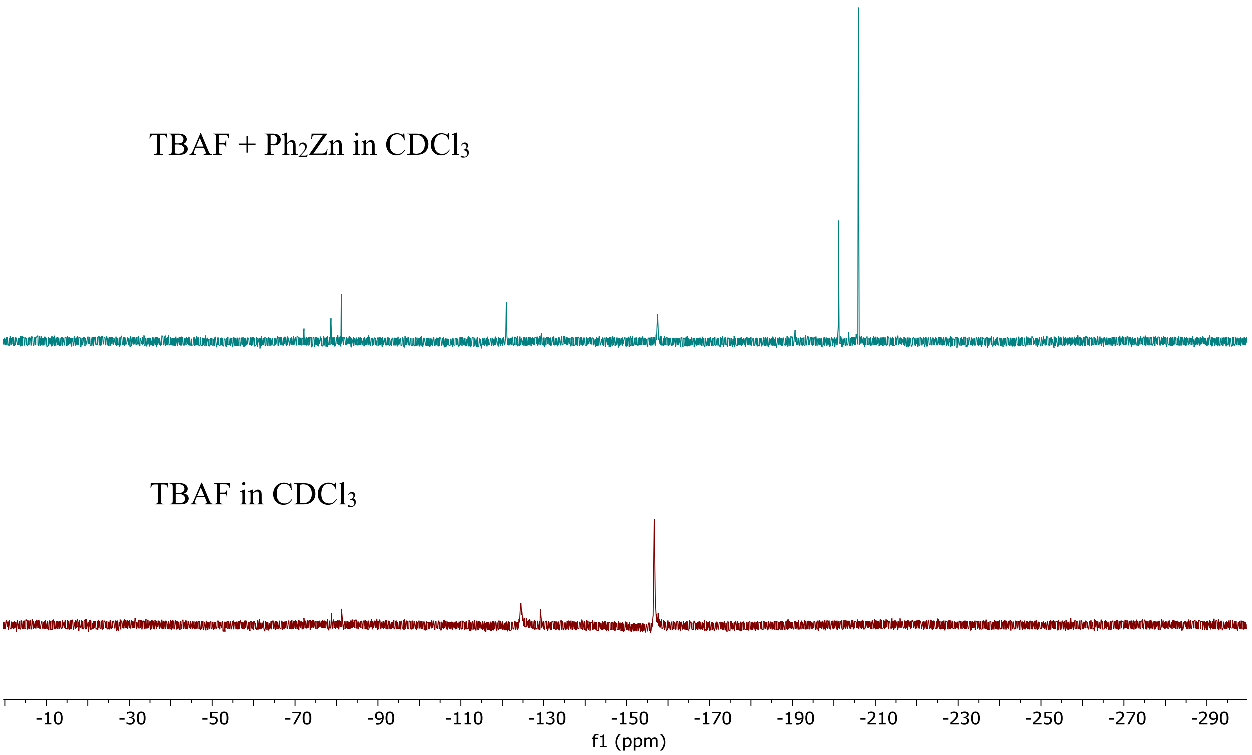

Supplementary Fig. 8. Top: ^{19}F NMR analysis (376 MHz, $CDCl_3$) of the reaction between equimolar equivalents of diphenylzinc and TBAF. The upfield shifted resonances indicate formation of the bridged fluorozincate. Bottom: ^{19}F NMR analysis of free TBAF.

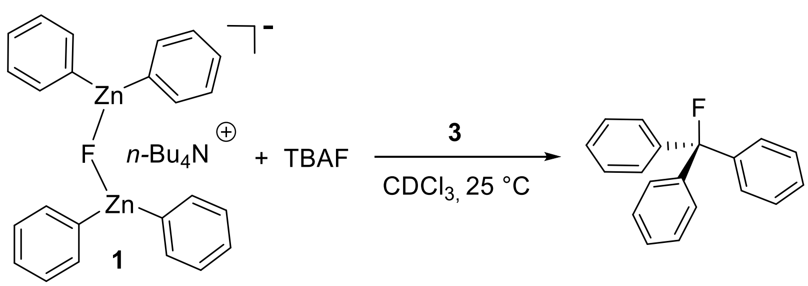

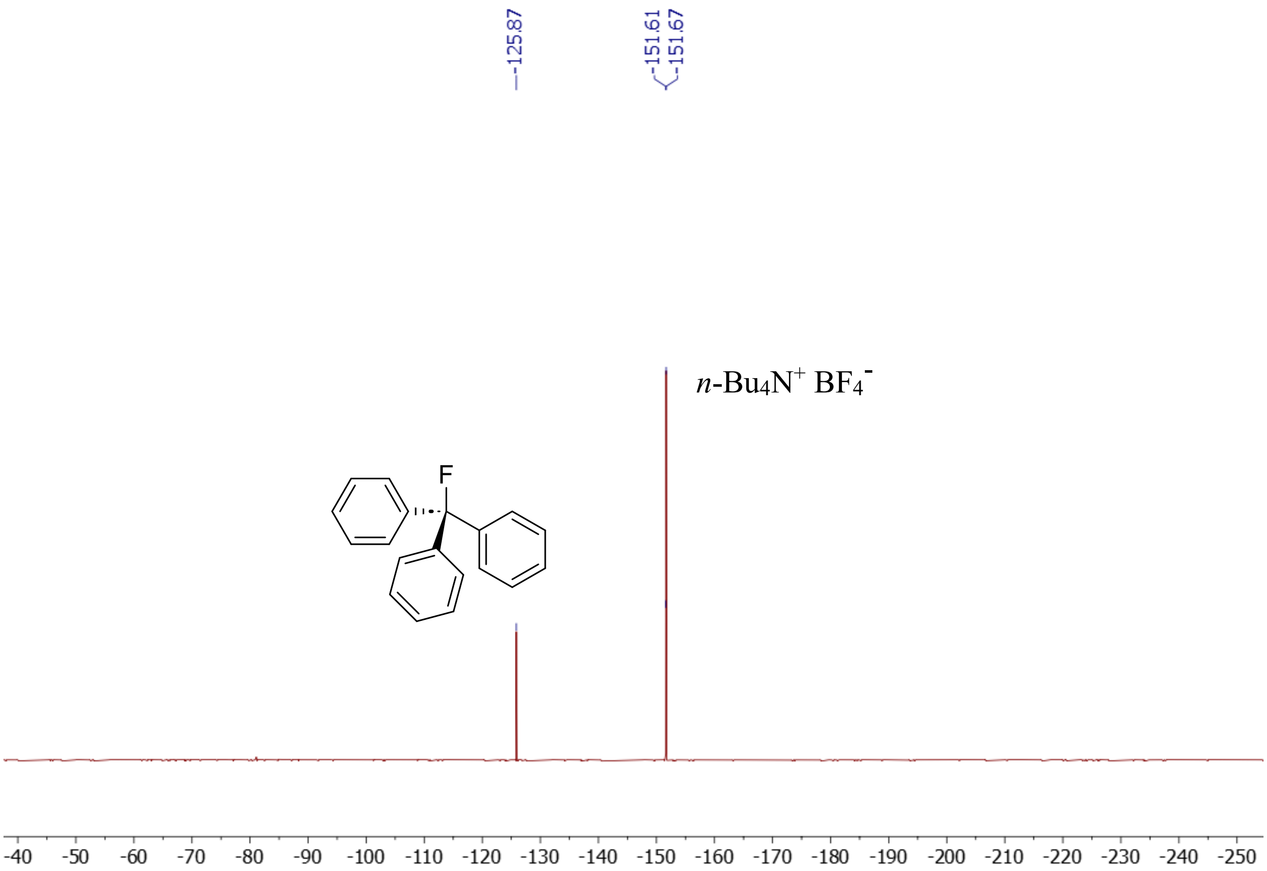

Supplementary Fig. 9. ^{19}F NMR analysis (376 MHz, $CDCl_3$) following the addition of trityllium tetrafluoroborate, **3**, to the equimolar mixture of TBAF and diphenylzinc (Supplementary Figure 8). Near quantitative formation of trityl fluoride is observed, in addition to the tetrabutylammonium tetrafluoroborate byproduct.

2.2. Isolation and Characterization of a Supramolecular Zinc Cluster Intermediate

At low temperature, the reaction of tri(4-tolyl)fluoromethane with diphenylzinc gave a persisting yellow color consistent with the formation of a trityl cation. Crystallization and X-ray analysis gave the ion pair shown below. Dichloromethane was used as solvent due to the fairly high freezing point of $PhCF_3$ (-29 °C).

In a nitrogen filled glovebox, dichloromethane solutions of diphenylzinc (76.0 mg, 0.35 mmol, 0.50 mL) and tri(4-tolyl)fluoromethane (76.0 mg, 0.25 mmol, 0.50 mL) were cooled to -40 °C. The colorless diphenylzinc solution was added to the colorless alkyl fluoride solution, resulting in an immediate color change to deep yellow. The resulting yellow solution was filtered through a chilled 0.2 μm PVDF syringe filter, and the filtrate was allowed to stand at -40 °C overnight, affording single crystals suitable for X-ray diffraction.

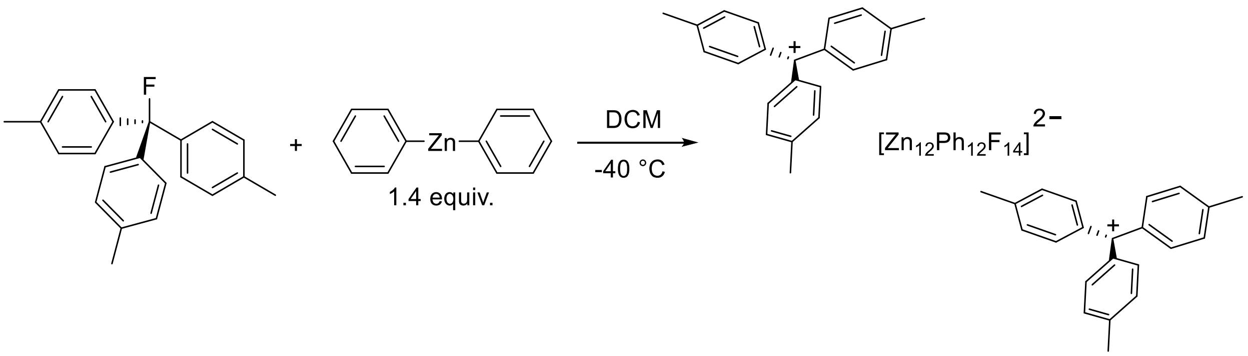

Supplementary Fig. 10. Formation of the supramolecular ion pair **33**.

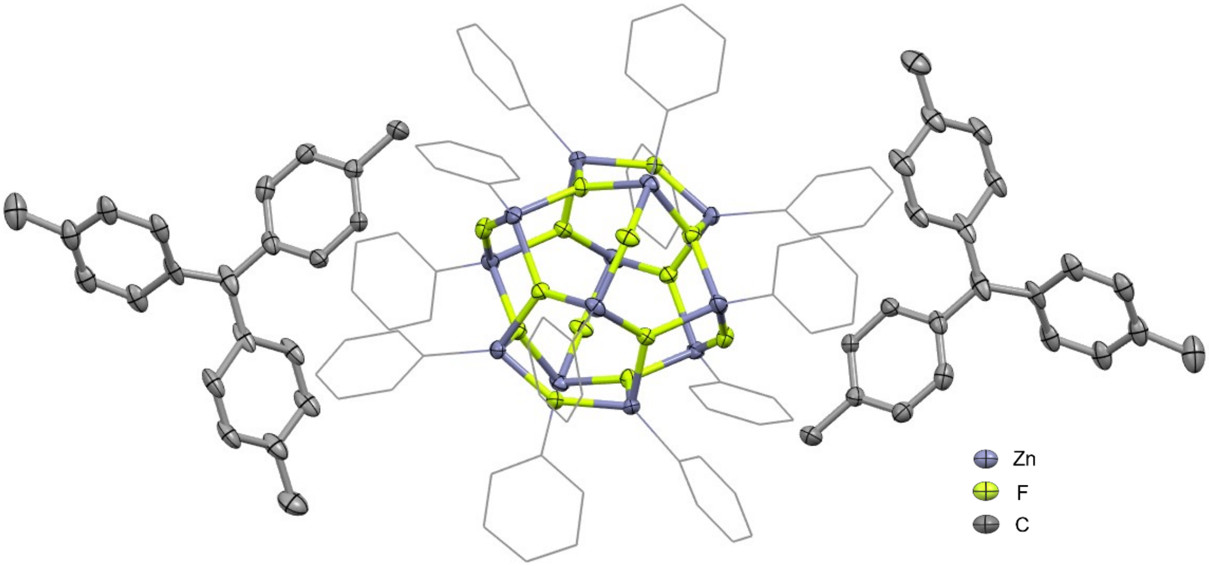

Supplementary Fig. 11. X-ray structure of **33**.

Single crystals were grown from a solution in dichloromethane at -40 °C. The inorganic interior and the trityl cations are displayed as ball-and-sticks. The exterior phenyl rings are shown as wireframe drawings and hydrogens are omitted for clarity.

The asymmetric unit consists of one cation and one half of the dianion. Four phenyl rings of the dianion are disordered over two orientations. The like C-C and Zn-C distances were restrained to be similar. The C7/C7B atom pair was constrained to have equal x,y,z positions and equal anisotropic displacement parameters. Two of the aryl groups on the cation are disordered over two positions. The like C-C distances were restrained to be similar. Similar displacement amplitudes were imposed on disordered sites overlapping by less than the sum of the van der Waals radii. A total of eight highly disordered dichloromethane solvate molecules per unit cell were removed from the model via the Squeeze routine in PLATON. Disordered groups are displayed in the sight of highest occupancy for clarity. Thermal ellipsoids are displayed at the 50% probability level. Selected bond lengths and angles: Zn1-F1 2.0261(16) Å, Zn1-F4 1.9198(19) Å, Zn2-C7 1.945(3) Å, Zn4-F3-Zn2 118.95°(8), Zn4-F3-Zn5 120.91°(8), Zn2-F3-Zn5 120.03°(8), Zn1-F4-Zn5 149.07°(10), Zn2-F2-Zn3 142.80°(9).

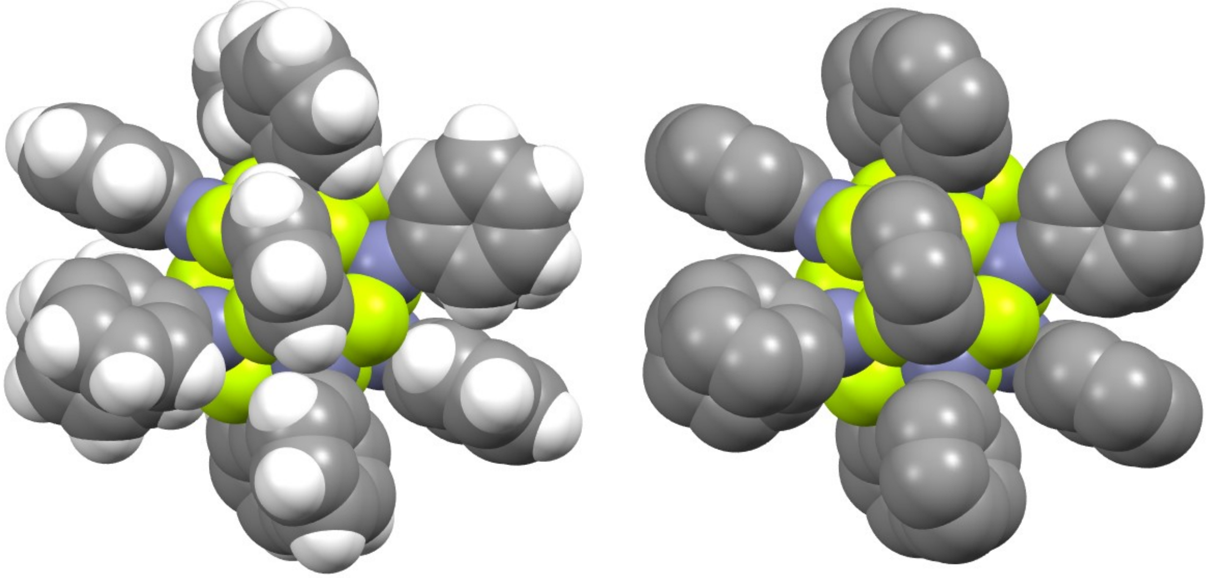

Supplementary Fig 12. Spacefilling representations highlighting the lipophilic exterior of the dodecanuclear zinc cluster **33**. Left: Hydrogen atoms are shown. Right: Hydrogen atoms are hidden.

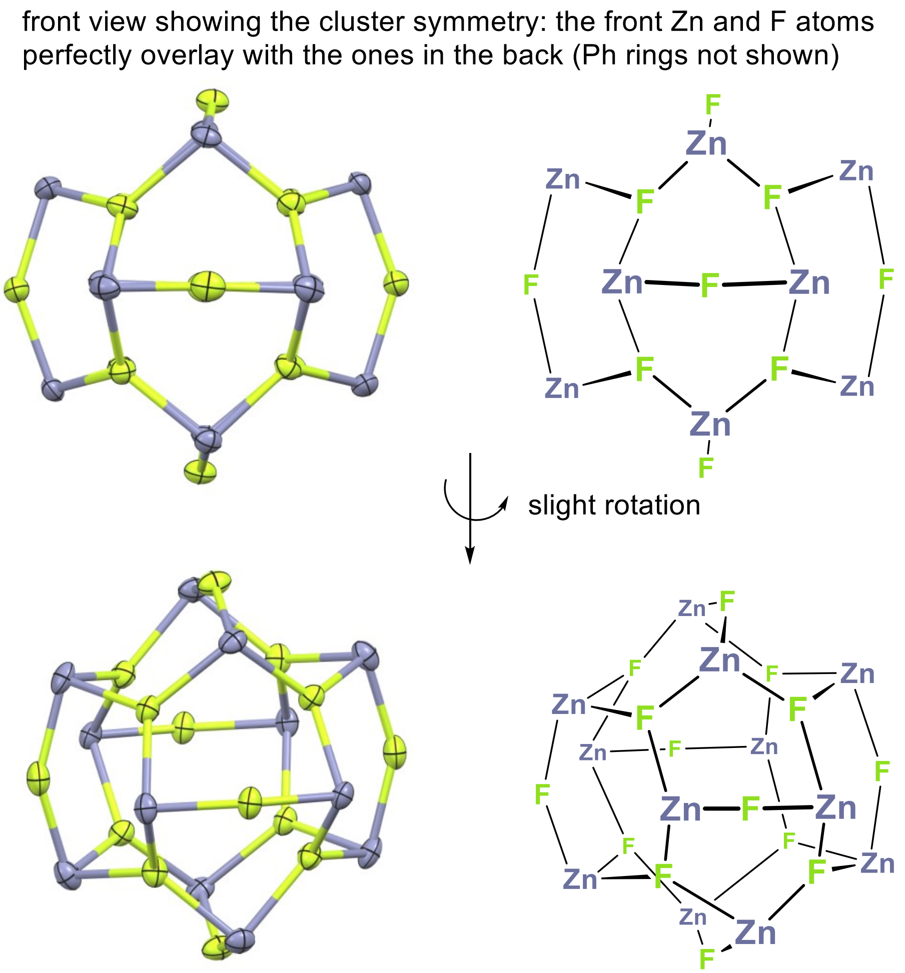

Supplementary Fig. 13. Crystallographic views of the dodecanuclear zinc cluster of **33**. Phenyl rings are hidden.

In a nitrogen filled glovebox, a solution of diphenylzinc (10.0 mg, 0.045 mmol) in $CDCl_3$ (0.5 mL) was added to a solution of tri(4-tolyl)fluoromethane (10.0 mg, 0.032 mmol) in $CDCl_3$ (0.5 mL) at -40 °C. An immediate color change from colorless to bright yellow was observed. The resulting homogenous yellow solution was immediately transferred to an NMR tube, sealed, removed from the glovebox, and analyzed in 5-minute intervals by ^{19}F NMR.

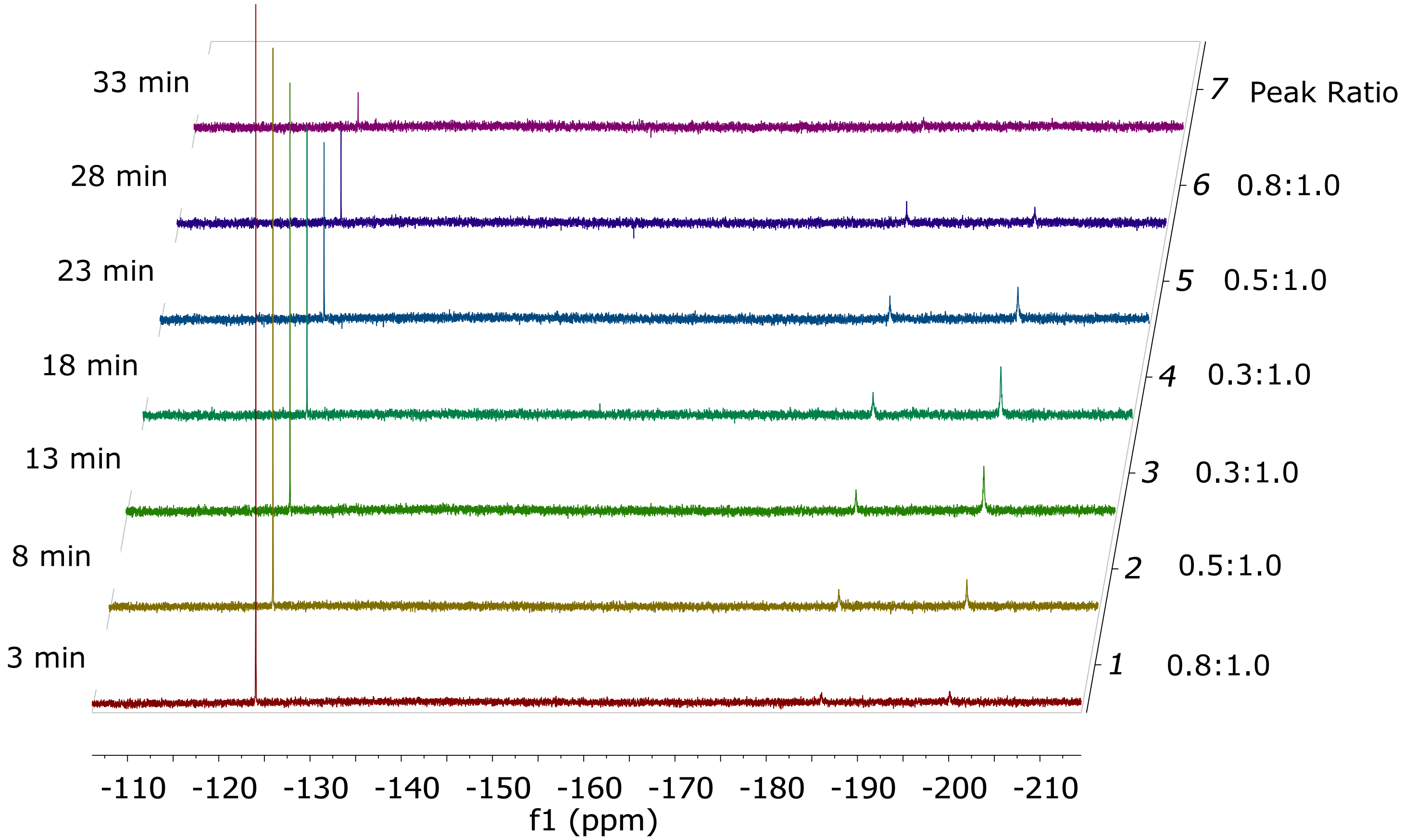

Supplementary Fig. 14. ^{19}F NMR (376 MHz, $CDCl_3$) spectroscopic monitoring of the C-F bond functionalization of tri(4-tolyl)fluoromethane.

Chemical shift values: -124.08 ppm (starting material), new species: -186.12, -200.13 ppm

2.3. Control Experiments

Radical clock reaction

The reaction of 3-fluoro-3-(pent-4-en-1-yl)-1-phenylindolin-2-one and diphenylzinc gave no evidence of radical intermediates. The C-F bond functionalization product **35** was isolated in 70% yield, and no cyclic by-products were detected.

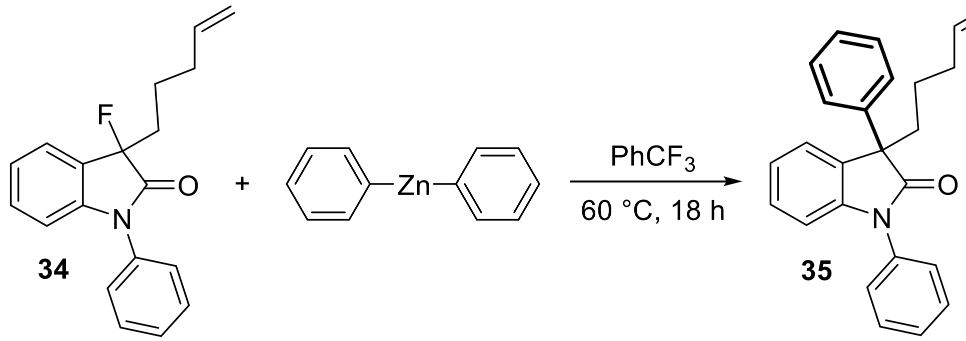

Supplementary Fig. 15. Radical clock experiment with **34**.

3-(Pent-4-en-1-yl)-1,3-diphenylindolin-2-one, 35. 3-Fluoro-3-(pent-4-en-1-yl)-1-phenylindolin-2-one, **34**, (74.0 mg, 0.25 mmol) and diphenylzinc (76.0 mg, 0.35 mmol) were combined in 1.0 mL of dry $PhCF_3$ and stirred at 60 ºC for 18 hours. The product was isolated by column purification using 5% ethyl acetate in hexanes as mobile phase as a white crystalline solid in 70% yield (62 mg, 0.18 mmol). Melting point range: 78-79 °C. ^{1}H NMR (400 MHz, chloroform-*d*) δ = 7.56 – 7.47 (m, 2H), 7.47 – 7.37 (m, 5H), 7.36 – 7.27 (m, 4H), 7.24 (m, 1H), 7.15 (m, 1H), 6.89 (m, 1H), 5.71 (m, 1H), 4.95 (m, 2H), 2.51 (m, 1H), 2.29 (m, 1H), 2.06 (m, 2H), 1.39 (m, 1H), 1.14 (m, 1H); ^{13}C NMR (100 MHz, chloroform-*d*) δ = 177.97, 143.84, 140.38, 138.02, 134.56, 132.12, 129.50, 129.47, 128.54, 127.96, 127.28, 126.89, 126.61, 124.98, 123.04, 114.97, 109.54, 56.61, 37.75, 33.72, 23.82. HRMS (ESI-QTOF) *m/z:* $[M+H]^+$ calculated for $C_{25}H_{23}NO$ 354.1858, found 354.1854.

Arylation of a primary alkyl fluoride in solvents of varying polarity

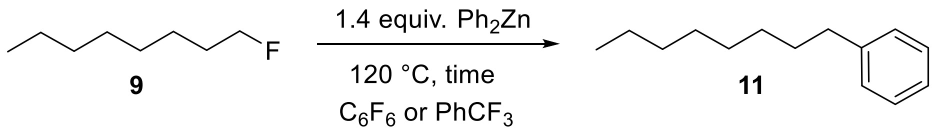

Supplementary Fig. 16. Phenylation of **9** to **11** in different solvents.

To assess the dependence of the reaction progress of a primary alkyl fluoride on solvent polarity, the reaction of 1-fluorooctane with diphenylzinc was carried out in hexafluorobenzene (ε = 2.05) and trifluorotoluene (ε = 9.40), respectively. Given that the fluorophilicity of organozinc compounds is sensitive to Lewis-basic solvents, the range of solvent polarity and dielectric constants investigated was limited. The progress of the phenylation of 1-fluorooctane under otherwise identical conditions was followed by GC-MS. The analyses of the reactions in hexafluorobenzene and trifluorotoluene gave very similar results. No discernable trend was observed with respect to solvent polarity.

In a nitrogen filled glovebox, 1-fluorooctane (5.0 mg, 0.038 mmol) and diphenylzinc were dissolved in 20.0 µL of hexafluorobenzene or trifluorotoluene. The solutions were heated to 120 °C and stirred. The reaction mixture was analyzed by GC-MS at 12, 24 and 48 hours.

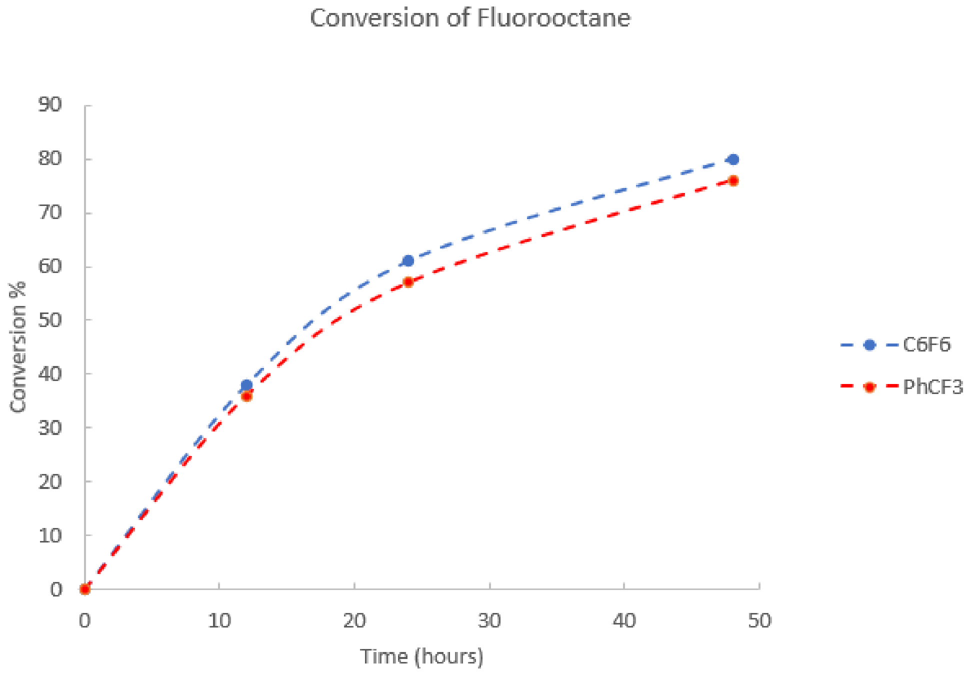

Supplementary Fig. 17. Conversion of **9** to **11** in different solvents.

Intermediate Trapping via Friedel-Crafts Reaction and with TEMPO

In an attempt to trap the intermediate adamantyl cation, the reaction of 1-fluoroadamantane and diphenylzinc was carried out in toluene, rather than trifluorotoluene as solvent. Surprisingly, this reaction gave only the 1-phenyladamantane product and no toluene adduct was observed. The reaction was therefore repeated with 1,3-dimethoxybenzene as solvent, which effectively trapped the adamantyl cation as described below.

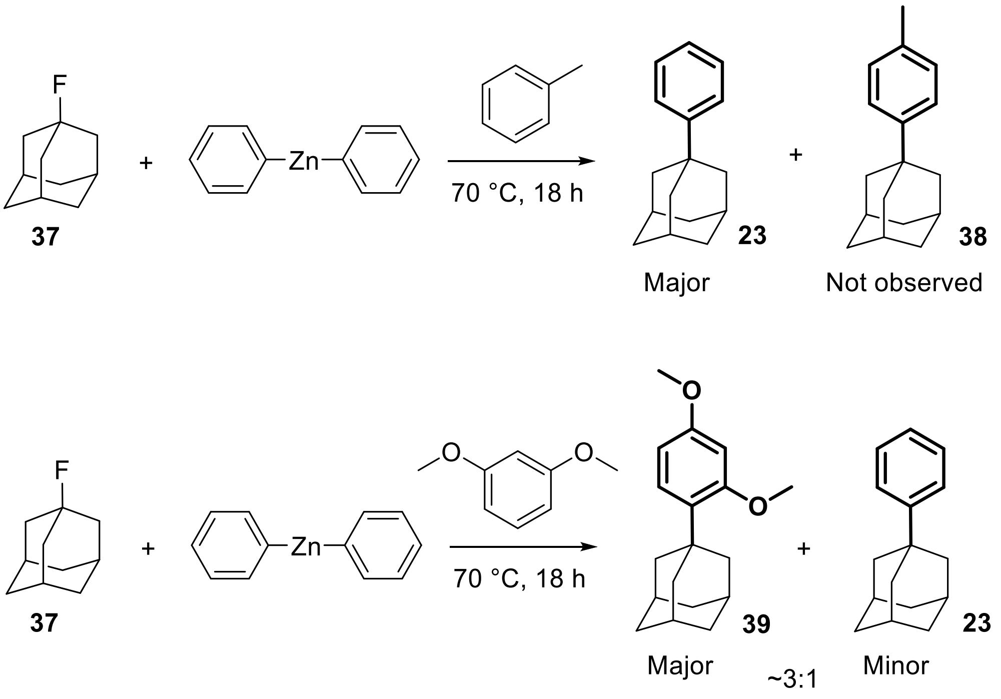

Supplementary Fig. 18. Friedel-Crafts trapping experiments with toluene and 1,3-dimethoxybenzene as solvent.

1-(2,4-Dimethoxyphenyl)adamantine, 39. 1-Fluoroadamantane, **37**, (38.0 mg, 0.25 mmol) and diphenylzinc (76.0 mg, 0.35 mmol) were combined in 1.0 mL of dry 1,3-dimethoxybenzene at 70 ºC for 18 hours. The reaction mixture was analyzed by GC-MS (see below). Compound **39** was isolated by column purification using 100% hexanes as mobile phase as a white crystalline solid in 46% yield (48.0 mg, 0.23 mmol). Melting point range: 92-95 °C. ^{1}H NMR (400 MHz, chloroform-*d*) δ = 7.11 (d, *J* = 8.5 Hz, 1H), 6.50 – 6.39 (m, 2H), 3.81 (s, 3H), 3.79 (s, 3H), 2.06 (bs, 9H), 1.76 (bs, 6H); ^{13}C NMR (100 MHz, chloroform-*d*) δ = 159.66, 158.67, 131.29, 126.74, 103.37, 99.70, 55.20, 54.90, 40.86, 37.14, 36.35, 29.12. The spectroscopic data of 1-(2,4-

dimethoxyphenyl)adamantane are in accordance with the literature [5]. Single crystals were grown by slow evaporation of a saturated pentane solution.

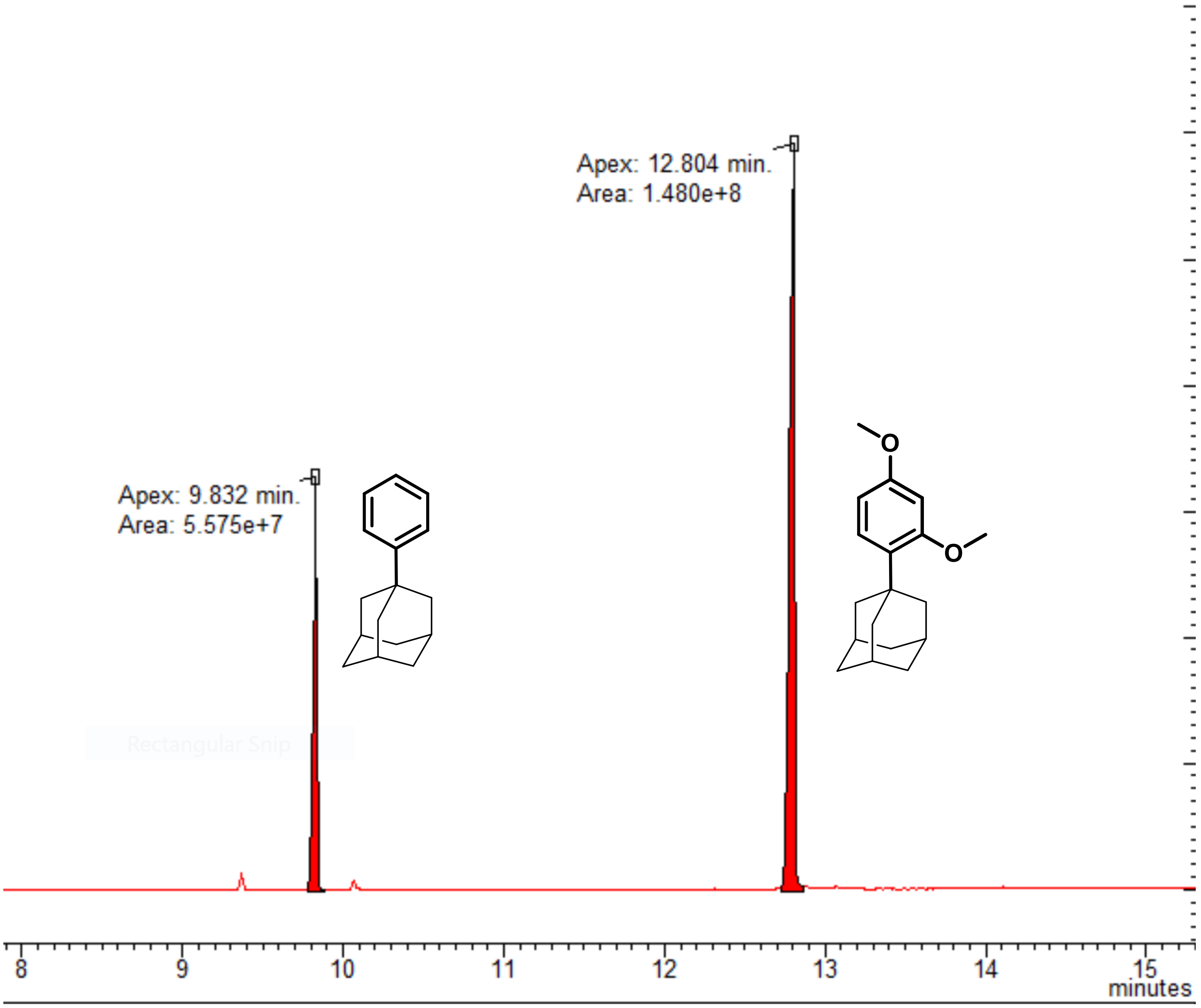

Supplementary Fig. 19. GC-MS analysis of the Friedel-Crafts trapping experiment with dimethoxybenzene as solvent. The product distribution of 1-(2,4-dimethoxyphenyl)adamantane and 1-phenyladamantane is approximately 3:1.

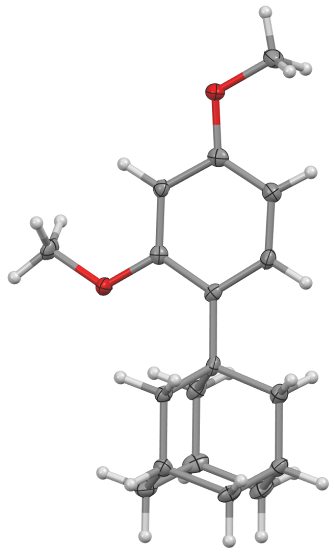

Supplementary Fig. 20. X-ray structure of 1-(2,4-dimethoxyphenyl)adamantine, **39**.
One molecule of **39** among two crystallographically distinct species is shown for clarity. Thermal ellipsoids are displayed at the 50% probability level. Single crystals were grown by slow evaporation of a saturated pentane solution.

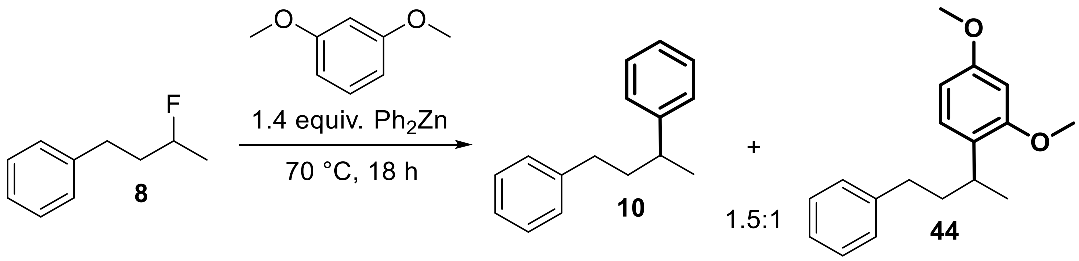

Supplementary Fig. 21. Friedel-Crafts trapping with **8**.

In a nitrogen filled glove box, (3-fluorobutyl)benzene (5.0 mg, 0.033 mmol) was treated with diphenylzinc (10.0 mg, 0.046 mmol) in the presence of 10.0 μL of 1,3-dimethoxybenzene. The reaction mixture was stirred at 70 °C for 18 hours and was monitored by GC-MS. Even though it was used in small amounts to allow full conversion of the starting material, 1,3-dimethoxybenzene effectively traps the secondary carbocation intermediate formed in this reaction.

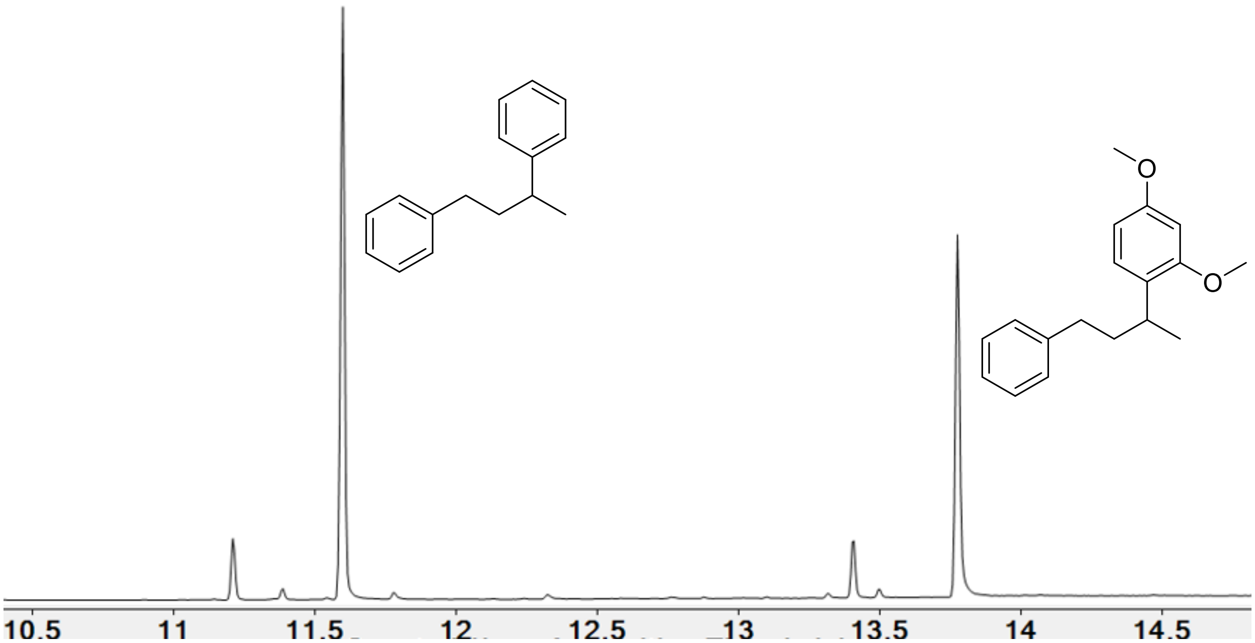

Supplementary Fig. 22. GC-MS analysis of the Friedel-Crafts trapping experiment of a secondary carbocation intermediate with 1,3-dimethoxybenzene as solvent. The product distribution of 1,3-diphenylbutane and 2,4-dimethoxy-1-(4-phenylbutan-2-yl)benzene is approximately 1.5:1.

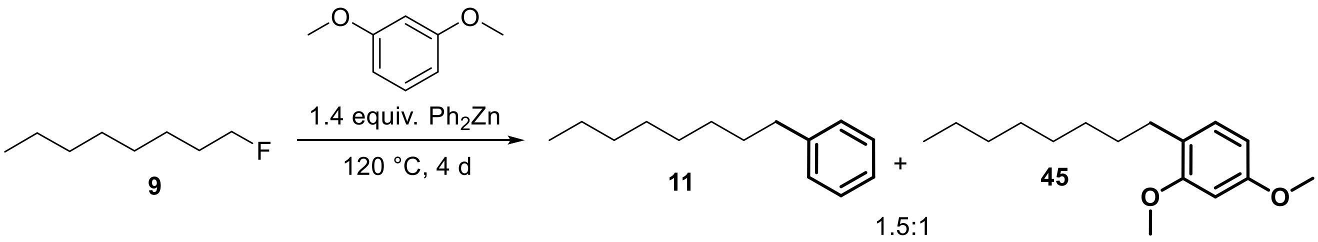

Supplementary Fig. 23. Friedel-Crafts trapping with **9**.

In a nitrogen filled glovebox, 1-fluorooctane (5.0 mg, 0.038 mmol) was treated with diphenylzinc (11.0 mg, 0.050 mmol) in 100 μL of 1,3-dimethoxybenzene. The reaction mixture was stirred at 120 °C for 4 days and was monitored by GC-MS. Even with primary substrates 1,3-dimethoxybenzene effectively traps the carbocation intermediate.

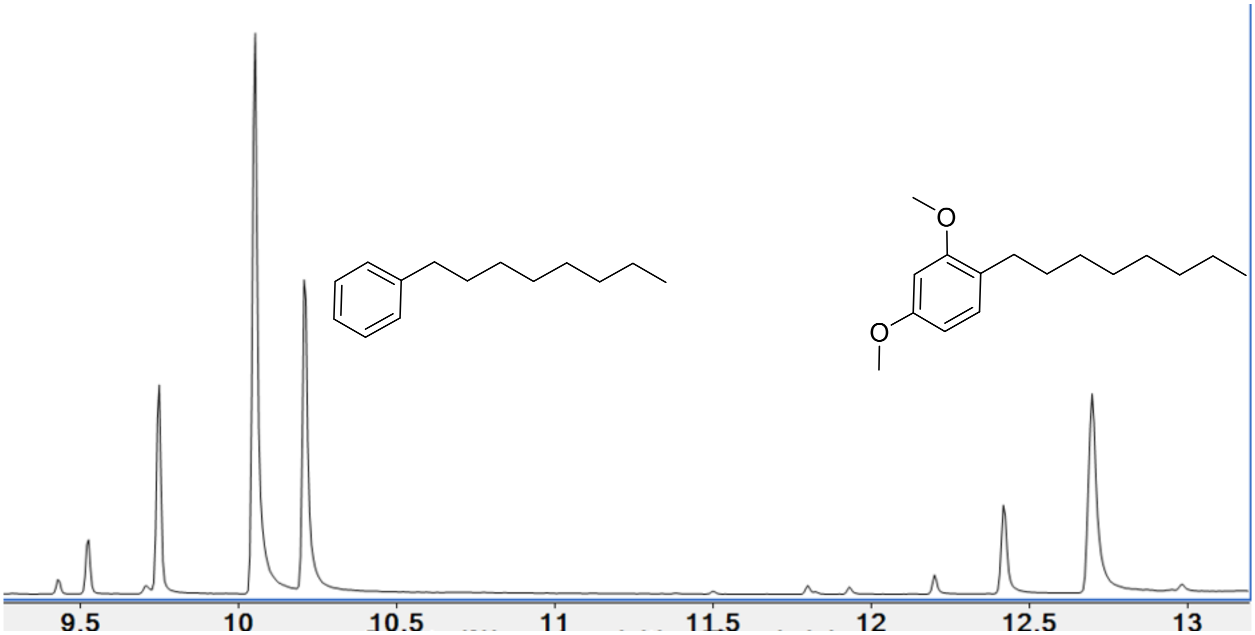

Supplementary Fig. 24. GC-MS analysis of the Friedel-Crafts trapping experiment of a primary alkyl fluoride with 1,3-dimethoxybenzene as solvent. The product distribution of phenyloctane isomers and 2,4-dimethoxy-1-octylbenzene isomers is approximately 1.5:1.

The reaction of 1-fluoroadamantane and diphenylzinc was carried out in presence of one equivalent of TEMPO. The reaction was monitored by GC-MS. The reaction proceeded with full conversion to 1-phenyladamantane and TEMPO adducts were not detected.

1-Fluoroadamantane (5.0 mg, 0.032 mmol) and TEMPO (5.0 mg, 0.032 mmol) were dissolved in $PhCF_3$ (1.0 mL). Diphenylzinc (9.9 mg, 0.045 mmol) was added and the reaction was heated to 70 °C for 18 hours. The reaction was analyzed by GC-MS.

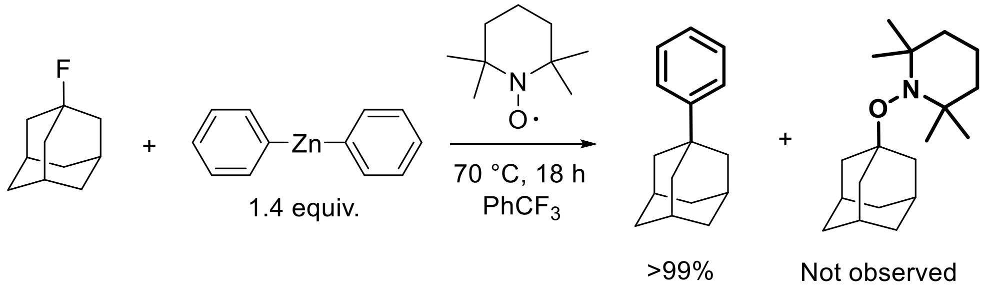

Supplementary Fig. 25. Trapping reaction with TEMPO.

2.4. Alkyl Chain Rearrangement Studies

In a nitrogen filled glovebox, (3-fluorobutyl)benzene (38.0 mg, 0.25 mmol) and diphenylzinc (76.0 mg, 0.35 mmol) were combined neat at 70 ºC for 18 hours. The mixture was analyzed by NMR and GC-MS. The competing hydride migration proved to be slow compared to the C-C bond formation step. 1,3-Diphenylbutane was formed in 92%, while 1,2-diphenylbutane and 1,1-diphenylbutane were present in only 6% and 2% respectively.

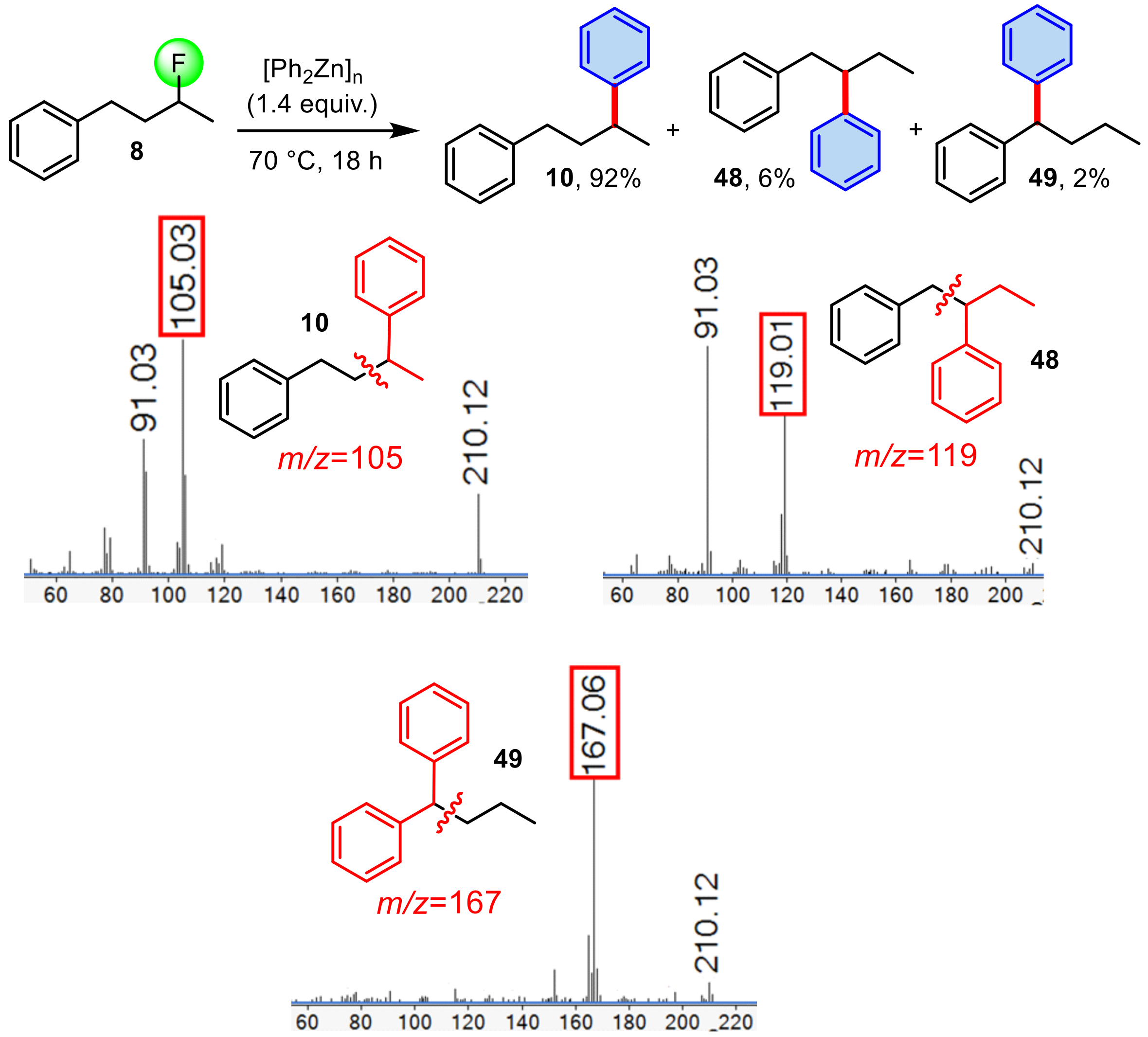

Supplementary Fig. 26. GC-MS analysis for diphenylbutane isomer identification.

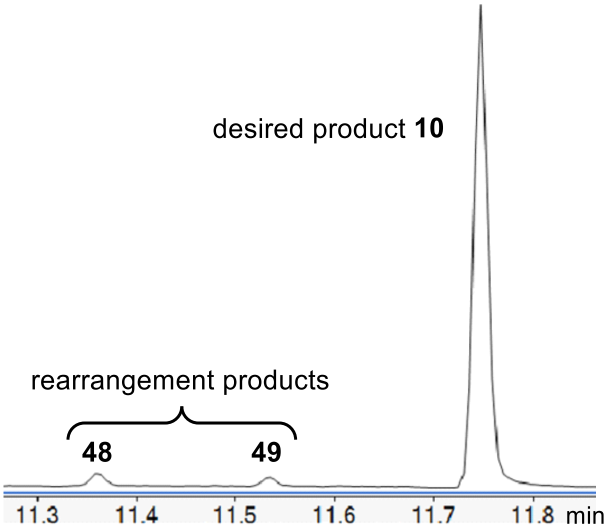

Supplementary Fig. 27. GC-MS analysis of the isomers of diphenylbutane obtained in the reaction between (3-fluorobutyl)benzene and diphenylzinc.

In a nitrogen filled glovebox, fluorooctane (5.0 mg, 0.038 mmol) and diphenylzinc (16.0 mg, 0.053 mmol) were dissolved in $PhCF_3$ (0.100 mL). The mixture was heated to 115 °C and the crude reaction mixture was analyzed by NMR and GC-MS after days. The competing hydride migration proved to be slow compared to the C-C bond formation step. 1-Phenyloctane was present in 70% by NMR, while 2-phenyloctane and 3-phenyloctane were detected in only 20% and 10% respectively. 4-Phenyloctane was detected in trace amounts by GC-MS, but was not visible by NMR.

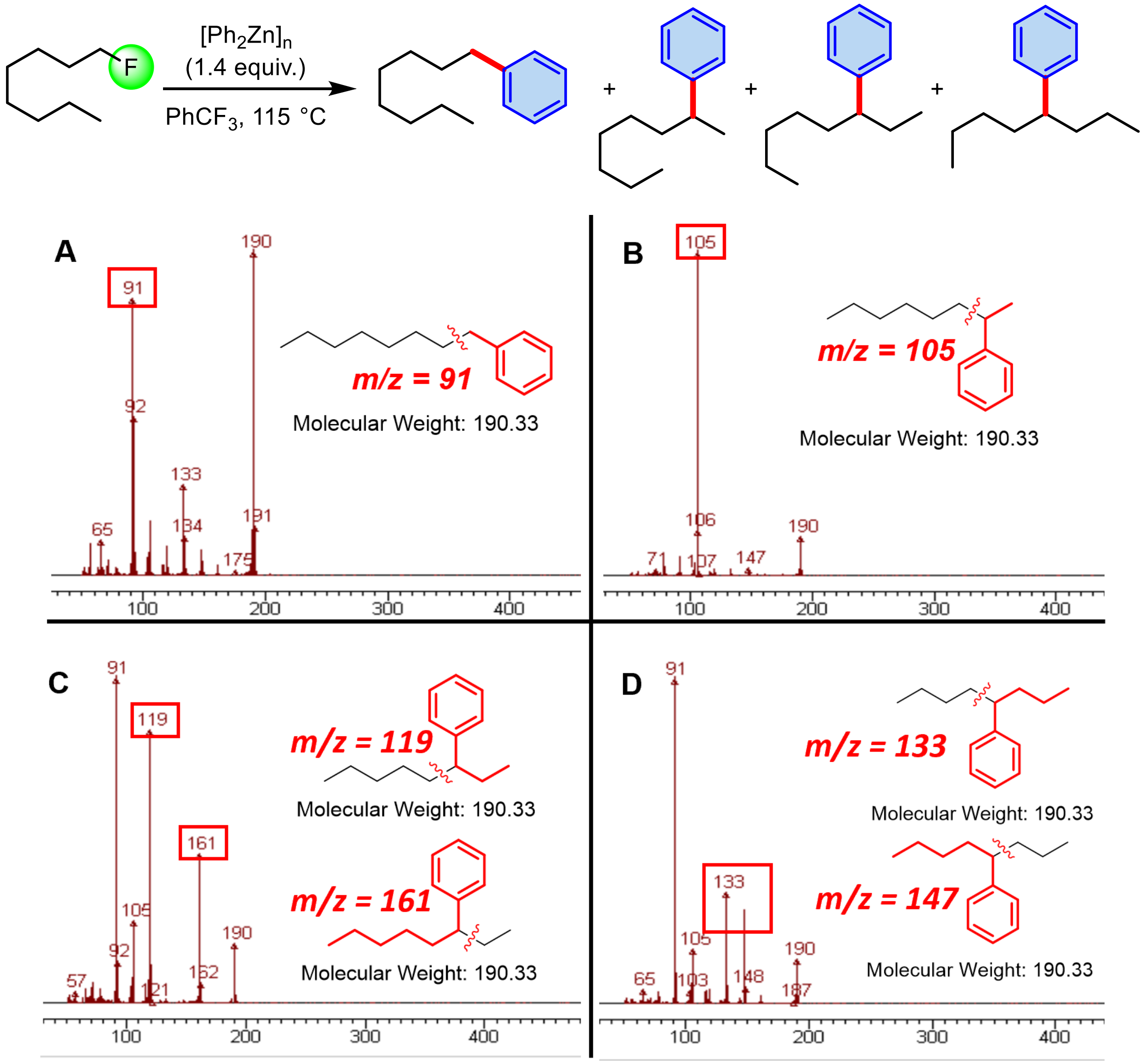

Supplementary Fig. 28. GC-MS analysis of the reaction between 1-fluorooctane and diphenylzinc.

In a nitrogen-filled glovebox, 1-fluorododecane (47.0 mg, 0.25 mmol) and bis(3-methylbenzo[b]thiophen-2-yl)zinc • THF (150.0 mg, 0.35 mmol) were combined in 0.1 mL of dry $PhCF_3$. The reaction was heated at 120 ºC for 3 days. The product was isolated by silica gel chromatography using 100% hexanes, and the product was characterized by NMR. The electron-rich bis(3-methylbenzo[b]thiophen-2-yl)zinc gives rise to only one isomer, the desired product 2-

dodecyl-3-methylbenzo[b]thiophene, while no sign of formation of a rearrangement product was observed.

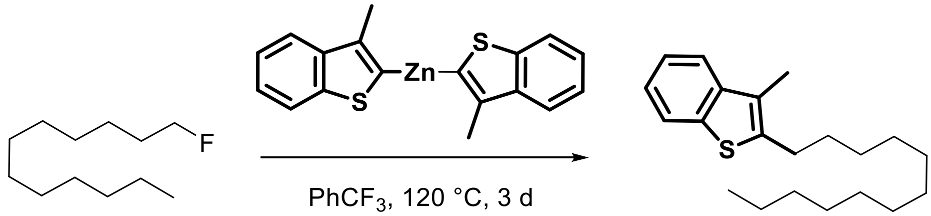

Supplementary Fig. 29. Reaction between 1-fluorododecane and bis(3-methylbenzo[b]thiophen-2-yl)zinc.

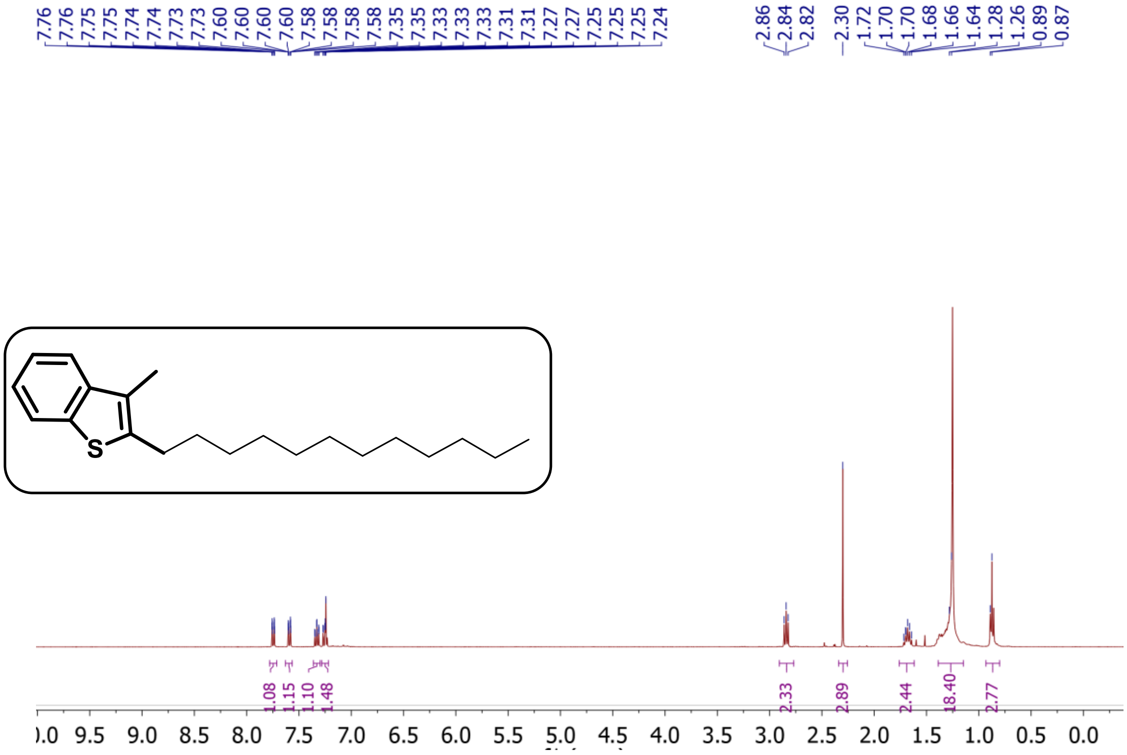

Supplementary Fig. 30. ^{1}H NMR (400 MHz, $CDCl_3$) spectrum of 2-dodecyl-3-methylbenzo[b]thiophene (**51**).

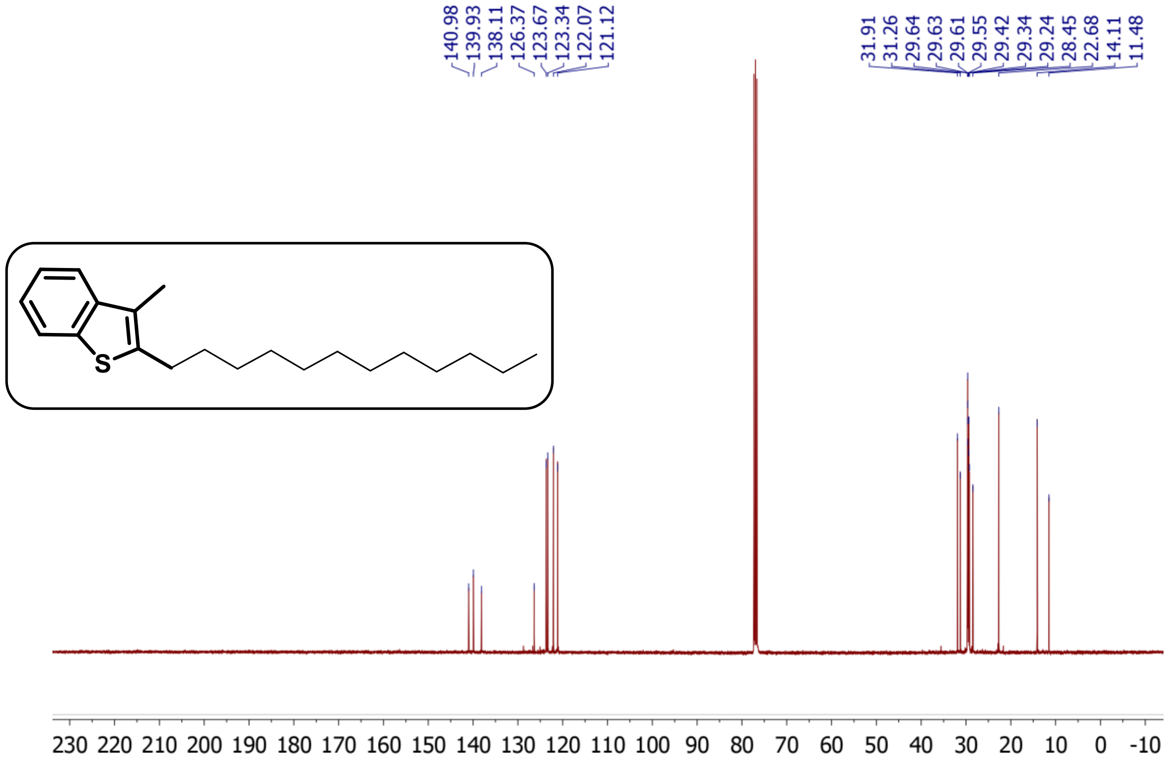

Supplementary Fig. 31. ^{13}C NMR (100 MHz, $CDCl_3$) spectrum of 2-dodecyl-3-methylbenzo[b]thiophene (**51**).

2.5. C-C Bond Formation with Enantiomerically Enriched Alkyl Fluorides

Retention of configuration in the reaction with (*S*)-(3-fluorobutyl)benzene.

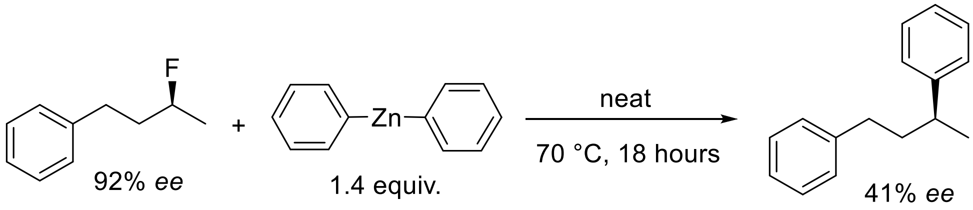

Supplementary Fig. 32. Reaction with (*S*)-(3-Fluorobutyl)benzene.

(*S*)-(3-Fluorobutyl)benzene was prepared from (*R*)-4-phenylbutan-2-ol according to a literature procedure [6]. The *ee* was determined as 92% by chiral HPLC (CHIRALCEL OJ-H, mobile phase: *n*-hexanes = 100%, flow rate = 1.0 mL/min, UV detection at 214 nm), t_R = 9.9 min (major), t_R = 9.1 min (minor).

In a nitrogen filled glove box, (*S*)-(3-fluorobutyl)benzene (5.0 mg, 0.033 mmol, 92% *ee*) was treated with diphenylzinc (10.0 mg, 0.046 mmol) without solvent. The reaction mixture was stirred at 70 °C for 18 hours to afford 1,3-diphenylbutane. The *ee* was determined by GC-MS (2,6-dimethyl-3-pentyl-γ-cyclodextrin, 120 °C) as 41%, t_R (major) = 5.7 min, t_R (minor) = 5.2 min. The absolute configuration of the major enantiomer was determined as *S* via polarimetry according to the literature [7]. The dominant, yet incomplete, racemization observed in this reaction is in agreement with a short-lived ion pair intermediate and rapid aryl nucleophile delivery that competes with molecular tumbling and dissociation processes.

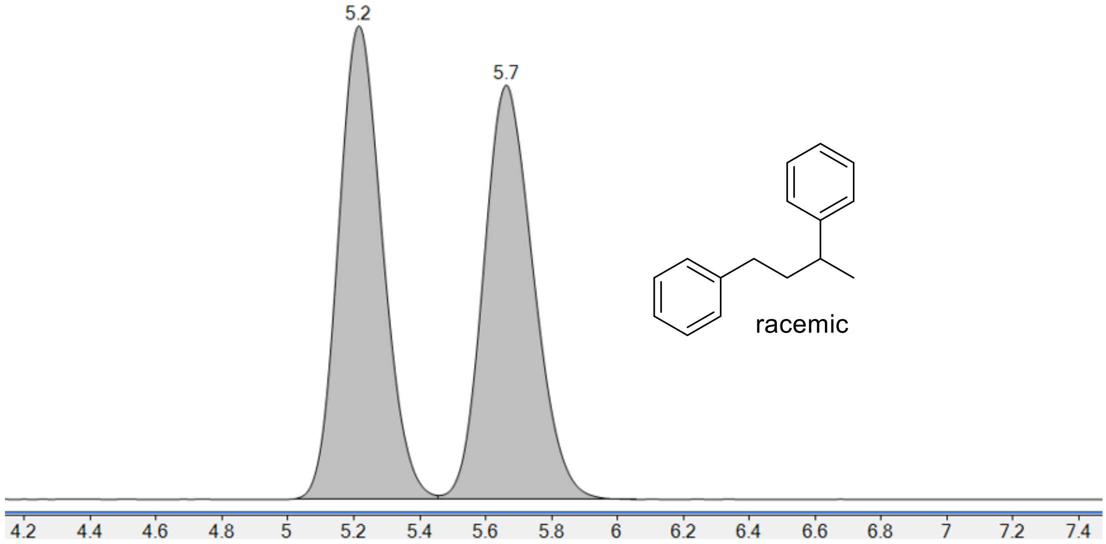

Supplementary Fig. 33. GC-MS Chromatogram of racemic 1,3-diphenylbutane. GC Capillary: 2,6-dimethyl-3-pentyl-γ-cyclodextrin, 120 °C.

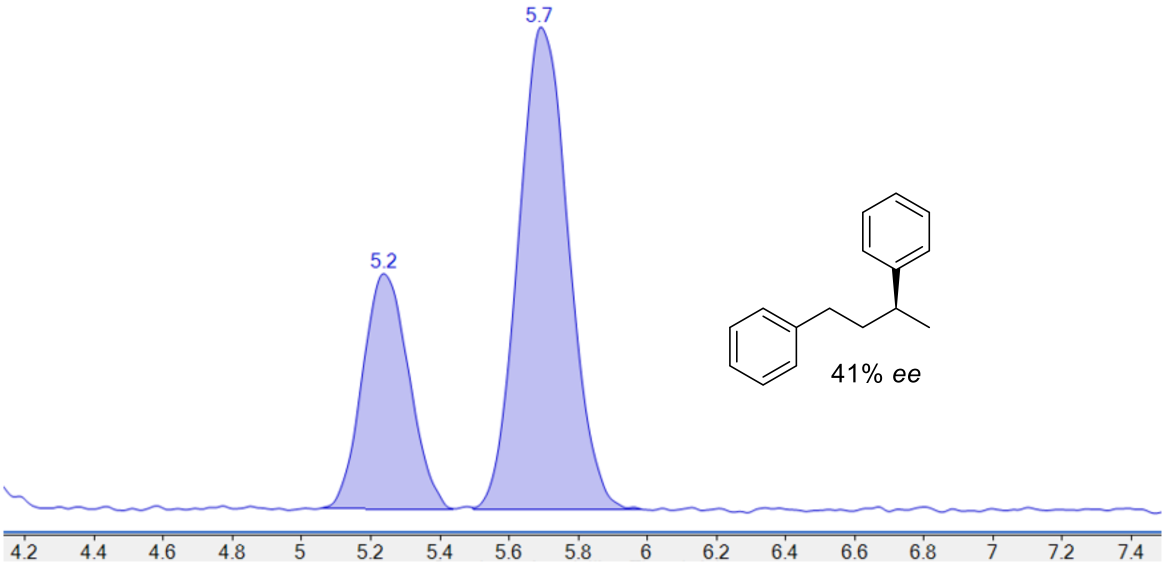

Supplementary Fig. 34. GC-MS Chromatogram of 1,3-diphenylbutane obtained from (*S*)-(3-fluorobutyl)benzene and Ph_2Zn. GC Capillary: 2,6-dimethyl-3-pentyl-γ-cyclodextrin, 120 °C.

Synthesis of (*R*)-4-ethyl-4-phenyl-1,12-dihydro-14*H*-pyrano[3',4':6,7]indolizino[1,2-*b*]quinoline-3,14(4*H*)-dione.

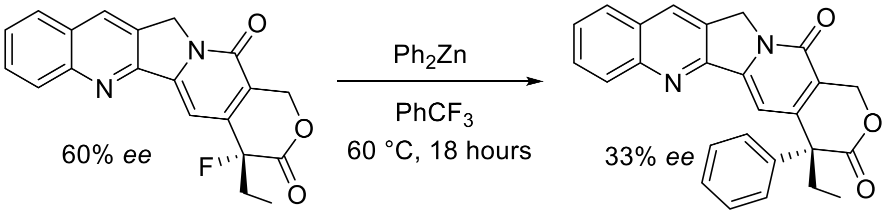

Supplementary Fig. 35. Reaction of Ph_2Zn with ((*R*)-4-ethyl-4-fluoro-1,12-dihydro-14*H*-pyrano[3',4':6,7]indolizino[1,2-*b*]quinoline-3,14(4*H*)-dione.

(*R*)-4-Ethyl-4-fluoro-1,12-dihydro-14*H*-pyrano[3',4':6,7]indolizino[1,2-*b*]quinoline-3,14(4*H*)-dione was prepared according to a literature procedure [8]. The *ee* was determined as 60% by chiral HPLC (CHIRALCEL OD-H, mobile phase: ethanol = 100%, flow rate = 0.5 mL/min, UV detection at 254 nm), t_R = 40.1 min (major), t_R = 32.4 min (minor).

(*R*)-4-Ethyl-4-phenyl-1,12-dihydro-14*H*-pyrano[3',4':6,7]indolizino[1,2-*b*]quinoline-3,14(4*H*)-dione was obtained from 4-ethyl-4-fluoro-1,12-dihydro-14H-pyrano[3',4':6,7]indolizino[1,2-

b]quinoline-3,14(4H)-dione (88.0 mg, 0.25 mmol) and diphenylzinc (76.0 mg, 0.35 mmol) in 1.0 mL of dry $PhCF_3$ at 60 °C for 18 hours. The product was isolated by column purification using 5% methanol in dichloromethane as mobile phase as a white amorphous solid in 61% yield (62 mg, 0.16 mmol). The *ee* was determined using a chiral HPLC (*S,S*)-Whelk-O 1 column.

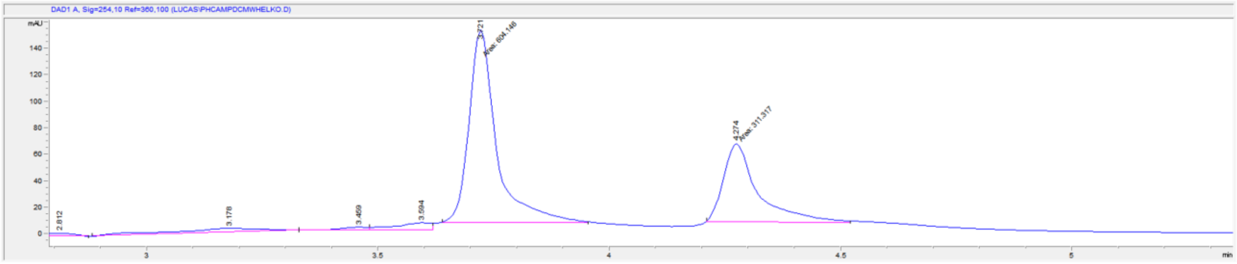

Supplementary Fig. 36. HPLC Chromatogram of (*R*)-4-ethyl-4-phenyl-1,12-dihydro-14*H*-pyrano[3',4':6,7]indolizino[1,2-*b*]quinoline-3,14(4*H*)-dione obtained from (*R*)-4-ethyl-4-fluoro-1,12-dihydro-14*H*-pyrano[3',4':6,7]indolizino[1,2-*b*]quinoline-3,14(4*H*)-dione with Ph_2Zn.

HPLC (*S,S*)-Whelk-O 1, mobile phase: dichloromethane = 100%, flow rate = 1.0 mL/min, UV detection at 254 nm) t_R = 3.7 min (major), t_R = 4.3 min (minor).

2.6. Selectivity for C(sp3)-F Bonds

The method is selective for C(sp^3)-F bonds. We found that C(sp^2)-F and even activated CF_2 and CF_3 groups (for example compounds **28**, **29**, **55**, **57**, **60**, **64** and **72**) are tolerated and remain intact. In fact, $PhCF_3$ is a preferred solvent for this reaction. Control experiments with activated difluorides under standard conditions showed no reaction.

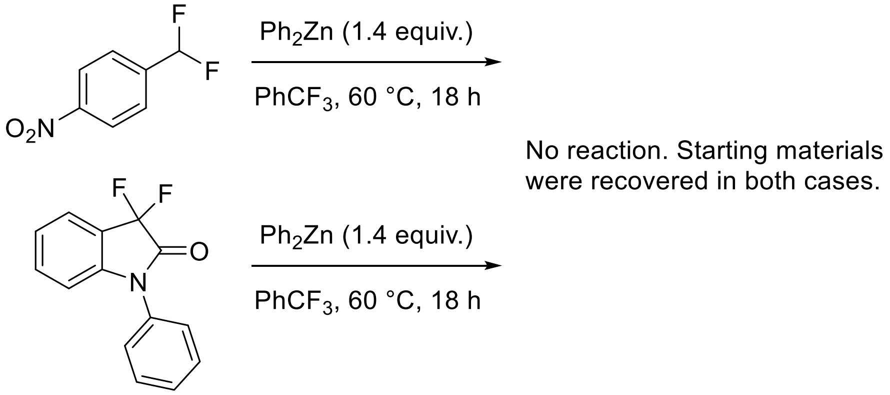

Supplementary Fig. 37. Tolerance of activated difluoromethylene groups.

3. Reaction Optimization

3.1. Screening of Reaction Conditions for Representative 1°, 2°, and 3° Alkyl Fluorides

3.1.1. 3-Fluoro-3-methyl-1-phenylindolin-2-one

3-Fluoro-3-methyl-1-phenylindolin-2-one (5.0 mg, 0.021 mmol) and diphenylzinc were dissolved in the desired solvent (1.0 mL). The reactions were heated to the specified temperature and stirred for 1-18 hours. Reaction mixtures were analyzed by GC-MS.

Supplementary Table 1. Optimization of the reaction between 3-fluoro-3-methyl-1-phenylindolin-2-one and diphenylzinc.

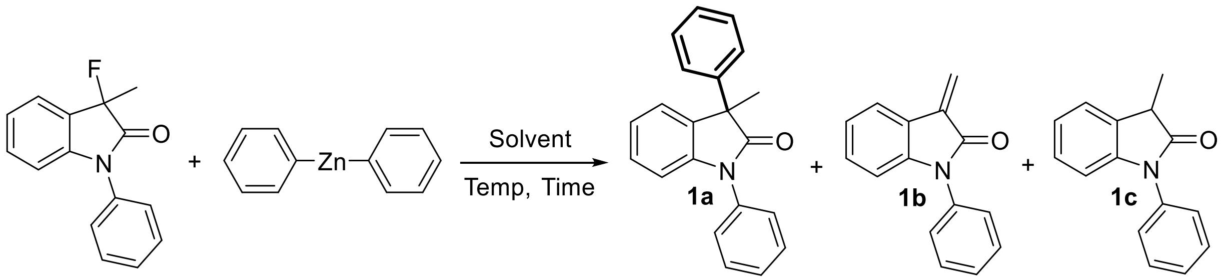

Solvent	Temperature (°C)	Time (h)	Equiv. diphenylzinc	Conversion (%)	% 1a	% 1b	% 1c
$PhCF_3$	90	18	1.4	99	80	10	9
cyclohexane	90	18	1.4	79	55	16	8
DCE	90	18	1.4	74	59	8	7
$PhCF_3$	25	1	1.4	0	0	0	0
$PhCF_3$	25	18	1.4	0	0	0	0
$PhCF_3$	40	18	1.4	80	64	8	8
**$PhCF_3$**	**60**	**18**	**1.4**	**99**	**80**	**10**	**9**
$PhCF_3$	60	6	1.4	92	74	9	9
$PhCF_3$	60	18	0.5	7	7	0	0

All conversions were determined by GCMS and ^1H NMR

3.1.2. (3-Fluorobutyl)benzene

In a nitrogen filled glovebox, (3-fluorobutyl)benzene (5.0 mg, 0.033 mmol) and diphenylzinc (1.4 equiv.) were dissolved in the desired solvent. The reactions were heated to the specified temperature and stirred for 18 hours. Reaction mixtures were analyzed by GC-MS.

Supplementary Table 2. Optimization of the reaction between (3-fluorobutyl)benzene and diphenylzinc.

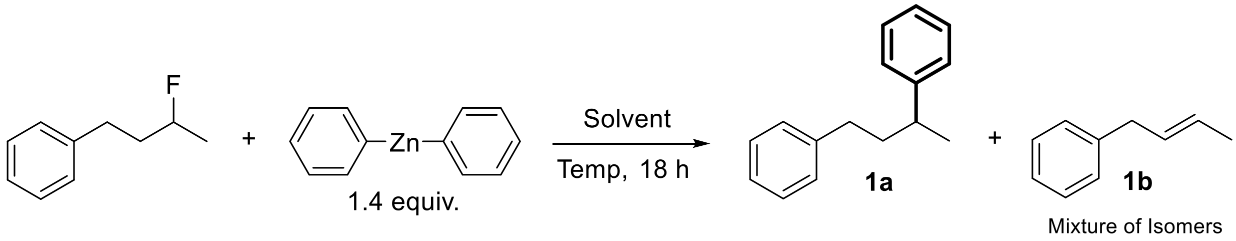

Solvent	Solvent volume (mL)	Temperature (°C)	Conversion (%)	1a %	1b %
$PhCF_3$	1.0	60	24	6	18
$PhCF_3$	1.0	90	70	17	53
$PhCF_3$	1.0	120	76	36	40
DCE	1.0	90	32	12	20
$PhNO_2$	1.0	90	0	-	-
THF	1.0	90	0	-	-
ACN	1.0	90	0	-	-
ACN	1.0	90[a]	0	-	-
$PhCF_3$	1.0	90[a]	32	12	20
$PhCF_3$	1.0	90[b]	50	9	41
$PhCF_3$	1.0	90	95[c]	27	67
$PhCF_3$	1.0	90	0[d]	-	-
$PhCF_3$	1.0	90	0[e]	-	-
$PhCF_3$	1.0	90	99[f]	68	32
$PhCF_3$	1.0	50	95[f]	41	15
$PhCF_3$	0.1	90	85	34	51
neat	-	90	99	62	38
**neat**	**-**	**70**	**99**	**73**	**27**
neat	-	50	59	19	40

All conversions were determined by GC-MS

[a]The reaction was run under microwave conditions: 100 W, 90 °C, 1.5 bar, 1 h

[b]The reaction was run under microwave conditions: 300 W, 90 °C, 1.5 bar, 5 h

[c]The reaction was run for 4 d

[d]30 mol% PPh_3 additive

[e]30 mol% TMEDA additive

[f]2 equiv. LiI additive

3.1.3. 1-Fluorooctane

In a nitrogen filled glovebox, 1-fluorooctane (5.0 mg, 0.038 mmol) and diphenylzinc were dissolved in $PhCF_3$ or combined without solvent. The reaction was heated to the specified temperature and stirred. The reaction mixture was analyzed by GC-MS.

Supplementary Table 3. Optimization of the reaction between 1-fluorooctane and diphenylzinc.

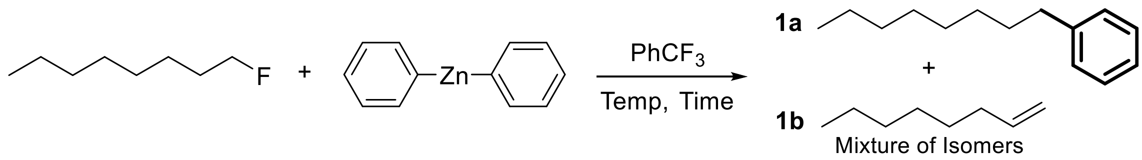

Solvent volume (mL)	Temperature (°C)	Ph_2Zn (Equiv.)	Time	Conversion (%)	% 1a	% 1b
1.0	70	1.4	18 h	0	0	0
1.0	90	1.4	18 h	0	0	0
1.0	120	1.4	18 h	0	0	0
0.1	70	1.4	18 h	0	0	0
0.1	90	1.4	18 h	0	0	0
0.1	120	1.4	18 h	28	22	6
0.5	90	1.4	18 h	60[a]	10[a]	0[a]
0.2	90	1.4	18 h	0[b]	0[b]	0[b]
-	90	1.4	18 h	0	0	0
-	120	1.4	4 d	15	12	3
0.05	120	1.4	18 h	36	29	7
0.05	120	1.4	3 d	74	59	15
0.02	120	1.4	18 h	54	43	11
**0.02**	**120**	**1.4**	**4 d**	**92**	**72**	**20**
0.02	120	2.0	4 d	91	72	19
0.02	120	4.0	4 d	90	71	19

All conversions were determined by GC-MS and ^1H NMR
[a]2 equiv LiI additive - octyl iodide was the major product
[b]1 equiv of DMAP additive

3.2. Screening of C-F Bond Functionalization with Various Organometallic Reagents

3-Fluoro-3-methyl-1-phenylindolin-2-one (5.0 mg, 0.021 mmol) and the organometallic reagent (1.4 equiv.) were dissolved in the desired solvent (1.0 mL). The reactions were heated to the specified temperature and stirred for 18 hours. Reaction mixtures were analyzed by GC-MS.

Supplementary Table 4. Initial reaction screening with 3-fluoro-3-methyl-1-phenylindolin-2-one.

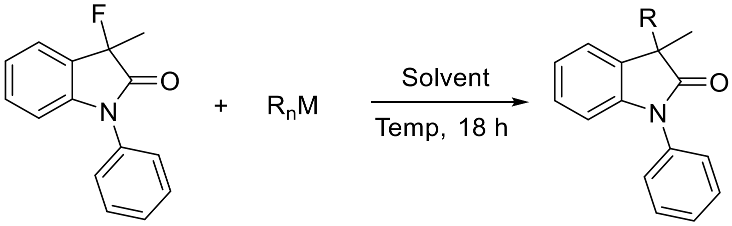

Reagent	Solvent	Temperature (°C)	Conversion (%)
Ph_4Si	toluene	25	0
Ph_4Si	toluene	50	0
Ph_4Si	toluene	90	0
$(C_3H_4)_4Si$	toluene	90	0
$(CH_3)_3SiCF_3$	toluene	90	0
$Ph_3SiF_2^-\ {}^nBu_4N^+$	toluene	90	0
Ph_3B	1,2-dichlorobenzene	90	0
Ph_3B	nitrobenzene	90	0
Ph_3B	$PhCF_3$	90	26
Ph_4Si	$PhCF_3$	90	0
Ph_4Sn	$PhCF_3$	90	0
**Ph_2Zn**	**$PhCF_3$**	**90**	**99**

All conversions were determined by GC-MS

4. General C-F Bond Arylation Procedures

Method A: The alkyl fluoride (0.25 mmol, 1 equiv.) and the diarylzinc reagent (0.35 mmol, 1.4 equiv.) were added into anhydrous $PhCF_3$ (volumes specified below) under nitrogen atmosphere. The reaction mixture was stirred at 50-120 °C (specified below) for 18 hours. The crude mixture was loaded onto silica and purified by flash column chromatography as described below.

Method B: The alkyl fluoride (0.25 mmol, 1 equiv.) and the diarylzinc reagent (0.35 mmol, 1.4 equiv.) were combined without solvent under nitrogen atmosphere. The reaction mixture was stirred at 50-120 °C (specified below) for 18 hours. The crude mixture was loaded onto silica and purified by flash column chromatography as described below.

5. Product Synthesis and Characterization

5.1. Synthesis of Diarylzinc Reagents

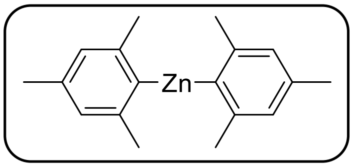

Dimesitylzinc. In a nitrogen filled glovebox, a solution of anhydrous zinc(II)chloride (100 mg, 0.733 mmol) in 3.0 mL of dry diethyl ether was added dropwise to a THF solution of mesityl magnesium bromide (1.46 mL, 1.46 mmol) at room temperature. The mixture was allowed to stir overnight, filtered through a 0.2 μm PVDF syringe filter, and the filtrate was dried *in vacuo* to yield the desired compound as a white crystalline solid that was used without further purification. ^{1}H NMR (400 MHz, acetonitrile-*d*3) δ = 6.69 (s, 4H), 2.42 (s, 12H), 2.19 (s, 6H).

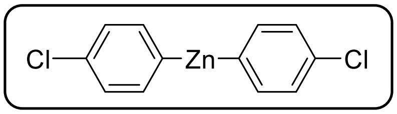

Bis(4-chlorophenyl)zinc · 2 THF. In a nitrogen filled glovebox, a solution of anhydrous zinc(II)chloride (100 mg, 0.733 mmol) in 3.0 mL of dry diethyl ether was added dropwise to a THF solution of 4-chlorophenyl magnesium bromide (1.46 mL, 1.46 mmol) at room temperature. The mixture was allowed to stir overnight, filtered through a 0.2 μm PVDF syringe filter, and the filtrate was dried *in vacuo* to yield the desired compound as a colorless amorphous solid that was used without further purification. ^{1}H NMR (400 MHz, acetonitrile-*d*3) δ = 7.72 (d, *J* = 8.0 Hz, 4H), 7.16 (d, *J* = 8.1 Hz, 4H).

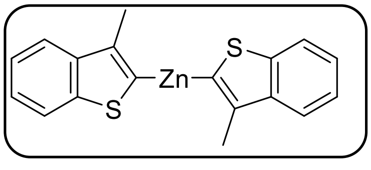

Bis(3-methylbenzo[b]thiophen-2-yl)zinc · THF. In a nitrogen filled glovebox, a solution of nBuLi (0.300 mL, 0.711 mmol) in hexanes was added dropwise to a solution of 3-methylbenzo[b]thiophene (100 mg, 0.674 mmol) in 3.0 mL of THF at -40 °C. The solution was

allowed to stir for 2 hours while warming to room temperature. A solution of anhydrous zinc(II)chloride (46 mg, 0.337 mmol) in 2.0 mL of THF was added dropwise, and the resulting solution was allowed to stir at room temperature overnight. The solution was dried *in vacuo*, and the residue was dissolved in 5 mL of dichloromethane, filtered through a 0.2 μm PVDF syringe filter, and dried to yield the desired compound as a tan colored oil that was used without further purification. ^{1}H NMR (400 MHz, acetonitrile-*d*3) δ = 7.82 (d, *J* = 8.0 Hz, 2H), 7.63 (d, *J* = 8.0 Hz, 2H), 7.27 (dd, *J* = 8.0, 7.0 Hz, 2H), 7.15 (dd, *J* = 8.0, 7.0 Hz, 2H), 2.50 (s, 6H).

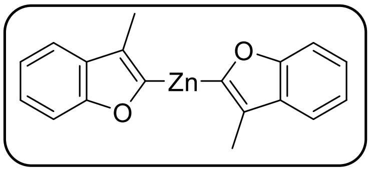

Bis(3-methylbenzofuran-2-yl)zinc · THF. In a nitrogen filled glovebox, a solution of nBuLi (0.666 mL, 1.66 mmol) in hexanes was added dropwise to a solution of 3-methylbenzofuran (0.200 mg, 1.51 mmol) in 6.0 mL of THF at -40 °C. The solution was allowed to stir for 2 hours while warming to room temperature. A solution of anhydrous zinc(II)chloride (103 mg, 0.755 mmol) in 3.0 mL of THF was added dropwise, and the resulting solution was allowed to stir at room temperature overnight. The solution was dried *in vacuo*, and the residue was dissolved in 5.0 mL of dichloromethane, filtered through a 0.2 μm PVDF syringe filter, and dried to yield the desired compound as a tan colored oil that was used without further purification. ^{1}H NMR (400 MHz, acetonitrile-*d*3) δ = 7.46 – 7.32 (m, 4H), 7.15 – 7.03 (m, 4H), 2.32 (s, 6H).

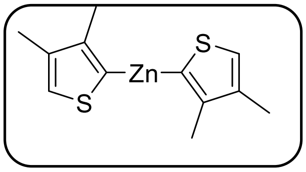

Bis(3,4-dimethylthiophen-2-yl)zinc · 2 THF. In a nitrogen filled glovebox, a solution of nBuLi (0.784 mL, 1.96 mmol) in hexanes was added dropwise to a solution of 3,4-dimethylthiophene (0.200 mg, 1.78 mmol) in 6.0 mL of THF at -40 °C. The solution was allowed to stir for 2 hours while warming to room temperature. A solution of anhydrous zinc(II)chloride (121 mg, 0.89 mmol) in 3.0 mL of THF was added dropwise, and the resulting solution was allowed to stir at

room temperature overnight. The solution was dried *in vacuo*, and the residue was dissolved in 5.0 mL of dichloromethane, filtered through a 0.2 μm PVDF syringe filter, and dried to yield the desired compound as a tan colored oil that was used without further purification. ^{1}H NMR (400 MHz, acetonitrile-*d*3) δ = 7.02 (s, 2H), 2.21 (s, 6H), 2.15 (s, 6H).

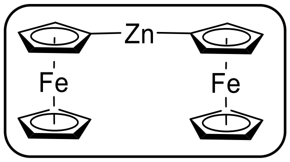

Diferrocenylzinc. In a nitrogen filled glovebox, a solution of nBuLi (0.362 mL, 0.906 mmol) in hexanes was added dropwise to a solution of bromoferrocene (0.200 mg, 0.755 mmol) in 3.0 mL of pentane at room temperature. The solution was allowed to stir for 2 hours, upon which an orange precipitate was observed. The precipitate was collected, washed three times with pentane, then dissolved in 3.0 mL of THF. A solution of anhydrous zinc(II)chloride (0.51 mg, 0.377 mmol) in 2.0 mL THF was added dropwise, and the resulting solution was allowed to stir at room temperature overnight. The solution was dried *in vacuo*, and the residue was dissolved in 5.0 mL of dichloromethane, filtered through a 0.2 μm PVDF syringe filter, and dried to yield the desired compound as an orange solid that was used without further purification. ^{1}H NMR (400 MHz, acetonitrile-*d*3) δ = 4.23 (m, 2H), 4.09 (s, 5H), 4.07 (m, 2H). Single crystals were grown from a saturated pentane solution at – 40 °C.

5.2. Synthesis of Alkyl Fluorides

1-Fluorooctane, 1-fluorododecane, 4-fluoro-1,3-dioxolan-2-one, 1-fluoroadamantane, 3-chloro-2-fluorobenzoyl fluoride, 4-(trifluoromethyl)benzoyl fluoride, benzoyl fluoride, and 4-bromo-1-(fluoromethyl)-2-methylbenzene were purchased and used without further purification. Alkyl fluorides that were not commercially available were synthesized according to the procedures described below. The identity and purity of all products were confirmed by NMR spectroscopy, see below.

Method A: Under nitrogen, the corresponding alcohols were dissolved in dichloromethane and cooled to 0 °C in an ice bath. One equivalent of diethylaminosulfurtrifluoride (DAST) was added

dropwise, and the resulting mixture was allowed to stir at room temperature until completion. The mixture was quenched with aqueous ammonium chloride and extracted with three portions of dichloromethane. The combined organic layers were dried over sodium sulfate, filtered, and dry-loaded onto silica gel for purification by flash chromatography.

Method B: Under nitrogen, the corresponding carbonyl compounds were dissolved in THF and cooled to 0 °C in an ice bath. One equivalent of sodium hydride was added, and the mixture was allowed to stir at room temperature for two hours. Then, 1.5 equivalents of NFSI were added, and the resulting mixture was allowed to stir at room temperature until completion. The mixture was quenched with aqueous ammonium chloride and was extracted with three portions of dichloromethane. The combined organic layers were dried over sodium sulfate, filtered, and dry-loaded onto silica gel for purification by flash chromatography.

Triphenylfluoromethane, (3-fluorobutyl)benzene, 1-fluorodec-2-yne, 1-(fluoromethyl)-4-nitrobenzene, 1-(3-fluorobutyl)-4-methoxybenzene, 1-(1-fluoroethyl)-3-nitrobenzene, (cyclohexylfluoromethyl)benzene, (3-fluorobut-1-yn-1-yl)benzene, 4-(fluoro(*o*-tolyl)methyl)benzonitrile, 2-(1-fluoro-1-phenylethyl)pyridine, tri(4-tolyl)fluoromethane, (1*S*,2*S*,4*S*,5*R*)-2-((*S*)-fluoro(6-methoxyquinolin-4-yl)methyl)-5-vinylquinuclidene, 5-((2-chlorophenyl)fluoro(4-fluorophenyl)methyl)pyrimidine, 4-ethyl-4-fluoro-1,12-dihydro-14H-pyrano[3',4':6,7]indolizino[1,2-b]quinoline-3,14(4H)-dione, 2-(3-fluoropropyl)isoindoline-1,3-dione, 5-(3-fluorobutyl)benzo[d][1,3]dioxole, and 1-bromo-4-(fluoro(phenyl)methyl)benzene were prepared in one step from the corresponding alcohol using diethylamino sulfurtrifluoride by method A.

3-Fluoro-3-methyl-1-phenylindolin-2-one, 2-fluoro-2-methyl-1-phenylpropan-1-one, and 2-((1-benzylpiperidin-4-yl)methyl)-2-fluoro-5,6-dimethoxy-2,3-dihydro-1H-inden-1-one were prepared in one step using electrophilic fluorination by method B.

5.3. C-F Bond Functionalization with Diphenylzinc

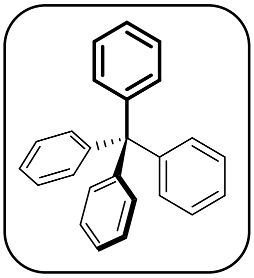

Tetraphenylmethane (4). Compound **4** was obtained from of triphenylfluoromethane (66.0 mg, 0.25 mmol) and diphenylzinc (76.0 mg, 0.35 mmol) in 1.0 mL of dry $PhCF_3$ at 60 ºC for 18 hours by following method A. The product was isolated by column purification using 100% hexanes as mobile phase, followed by sublimation of volatile impurities to yield a white crystalline solid in 73% yield (58 mg, 0.18 mmol). Melting point range: 280-282 °C. ^{1}H NMR (400 MHz, chloroform-*d*) δ = 7.29 – 7.13 (m, 20H); ^{13}C NMR (100 MHz, chloroform-*d*) δ = 146.75, 131.14, 127.40, 125.84, 29.67. The spectroscopic data of tetraphenylmethane (**4**) are in accordance with the literature.[9]

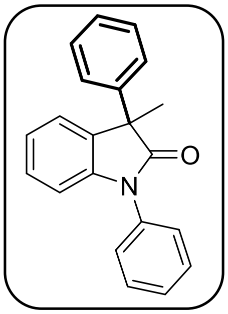

3-Methyl-1,3-diphenylindolin-2-one (6). Compound **6** was obtained from 3-fluoro-3-methyl-1-phenylindolin-2-one (60.0 mg, 0.25 mmol) and diphenylzinc (76.0 mg, 0.35 mmol) in 1.0 mL of dry $PhCF_3$ at 60 ºC for 18 hours by following method A. The product was isolated by column purification using 5% ethyl acetate in hexanes as mobile phase as a white crystalline solid in 68% yield (51 mg, 0.17 mmol). Melting point range: 125-126 °C. ^{1}H NMR (400 MHz, chloroform-*d*) δ = 7.52 (m, 2H), 7.45 – 7.39 (m, 4H), 7.33 (m, 2H), 7.29 – 7.22 (m, 4H), 7.13 (m, 1H), 6.92 (m, 1H), 1.92 (s, 3H); ^{13}C NMR (100 MHz, chloroform-*d*) δ = 178.74, 143.08, 140.87, 134.67, 134.57, 129.49, 128.58, 127.93, 127.27, 126.65, 126.57, 124.49, 123.19, 115.25, 109.61, 52.20, 23.99. The spectroscopic data of 3-methyl-1,3-diphenylindolin-2-one (**6**) are in accordance with the

literature.[10] X-ray quality single crystals were grown from evaporation of a saturated ethyl acetate solution.

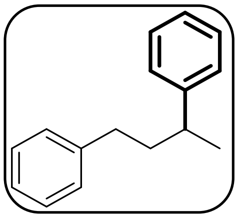

1,3-Diphenylbutane (10). Compound **10** was obtained from (3-fluorobutyl)benzene (38.0 mg, 0.25 mmol) and diphenylzinc (76.0 mg, 0.35 mmol) neat at 70 ºC for 18 hours by following method B. The product was isolated by column purification using 100% hexanes as mobile phase as a colorless oil in 64% yield (34 mg, 0.16 mmol). ^{1}H NMR (400 MHz, chloroform-*d*) δ = 7.36 – 7.27 (m, 2H), 7.25 – 7.11 (m, 8H), 2.72 (m, 1H), 2.52 (m, 2H), 1.92 (m, 2H), 1.28 (d, *J* = 6.9 Hz, 3H); ^{13}C NMR (100 MHz, chloroform-*d*) δ = 147.25, 142.51, 128.34, 128.32, 128.21, 127.02, 125.90, 125.58, 39.93, 39.47, 33.89, 22.45. The reaction gives a mixture of isomers that could not be separated. The given values correspond to the major isomer – 1,3-diphenylbutane. The spectroscopic data of 1,3-diphenylbutane (**10**) are in accordance with the literature.[11]

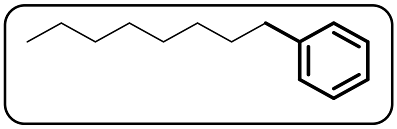

Octylbenzene (11). Compound **11** was obtained from 1-fluorooctane (33.0 mg, 0.25 mmol) and diphenylzinc (76.0 mg, 0.35 mmol) in 0.1 mL of dry $PhCF_3$ at 120 ºC for 4 days by following method A. The product was isolated by column purification using 100% hexanes as mobile phase as a colorless oil in 64% yield (30 mg, 0.16 mmol). ^{1}H NMR (400 MHz, chloroform-*d*) δ = 7.29 (m, 2H), 7.21 – 7.15 (m, 3H), 2.60 (t, *J* = 7.8 Hz, 2H), 1.60 (m, 2H), 1.30 – 1.19 (m, 10H), 0.90 (t, *J* = 6.3 Hz 3H); ^{13}C NMR (100 MHz, chloroform-*d*) δ = 142.93, 128.35, 128.16, 125.49, 35.97, 31.86, 31.50, 29.45, 29.32, 29.23, 22.64, 14.07. The reaction gives a mixture of isomers that could not be separated. The given values correspond to the major isomer – 1-phenyloctane. The spectroscopic data of octylbenzene (**11**) are in accordance with the literature.[12]

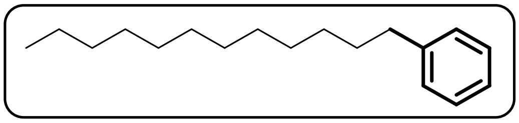

Dodecylbenzene (14). Compound **14** was obtained from 1-fluorododecane (47.0 mg, 0.25 mmol) and diphenylzinc (76.0 mg, 0.35 mmol) in 0.1 mL of dry $PhCF_3$ at 120 °C for 4 days by following method A. The product was isolated by column purification using 100% hexanes as mobile phase as a colorless oil in 59% yield (36 mg, 0.15 mmol). ^{1}H NMR (400 MHz, chloroform-*d*) δ = 7.29 (m, 2H), 7.20 – 7.16 (m, 3H), 2.60 (t, *J* = 7.8 Hz, 2H), 1.60 (m, 2H), 1.35 – 1.20 (m, 18H), 0.88 (m, 3H); ^{13}C NMR (100 MHz, chloroform-*d*) δ = 142.93, 128.35, 128.16, 125.49, 39.91, 38.42, 35.97, 31.90, 31.50, 29.65, 29.64, 29.57, 29.49, 29.33, 22.66, 14.09. The reaction gives a mixture of isomers that could not be separated. The given values correspond to the major isomer – 1-phenyldodecane. The spectroscopic data of dodecylbenzene (**14**) are in accordance with the literature.[12]

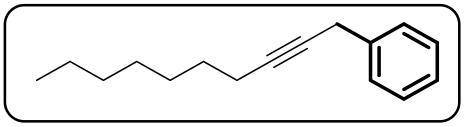

Dec-2-yn-1-ylbenzene (15). Compound **15** was obtained from 1-fluorodec-2-yne (39.0 mg, 0.25 mmol) and diphenylzinc (76.0 mg, 0.35 mmol) in 1.0 mL of dry $PhCF_3$ at 100 °C for 18 hours by following method A. The product was isolated by column purification using 100% hexanes as mobile phase as a colorless oil in 64% yield (34 mg, 0.16 mmol). ^{1}H NMR (400 MHz, chloroform-*d*) δ = 7.38 – 7.27 (m, 4H), 7.21 (m, 1H), 3.58 (t, *J* = 2.5 Hz, 2H), 2.22 (m, 2H), 1.53 (m, 2H), 1.41 (m, 2H), 1.34 – 1.28 (m, 6H), 0.90 (t, *J* = 6.9 Hz, 3H); ^{13}C NMR (100 MHz, chloroform-*d*) δ = 137.61, 128.34, 127.79, 126.31, 82.70, 77.44, 31.74, 29.01, 28.84, 28.79, 25.12, 22.59, 18.81, 14.06. HRMS (ESI-QTOF) *m/z:* $[M+H]^+$ calculated for $C_{16}H_{22}$ 215.1800, found 215.1787.

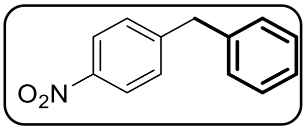

1-Benzyl-4-nitrobenzene (16). Compound **16** was obtained from 1-(fluoromethyl)-4-nitrobenzene (39.0 mg, 0.25 mmol) and diphenylzinc (76.0 mg, 0.35 mmol) in 1.0 mL of dry

$PhCF_3$ at 70 °C for 18 hours by following method A. The product was isolated by column purification using 5% ethyl acetate in hexanes as mobile phase as a brown oil in 66% yield (35 mg, 0.17 mmol). ^{1}H NMR (400 MHz, chloroform-*d*) δ = 8.15 (d, *J* = 8.6 Hz, 2H), 7.36 – 7.30 (m, 5H), 7.17 (m, 2H), 4.08 (s, 2H); ^{13}C NMR (100 MHz, chloroform-*d*) δ = 148.81, 139.14, 129.61, 129.10, 128.92, 128.78, 126.72, 123.73, 41.70. The spectroscopic data of 1-benzyl-4-nitrobenzene (**16**) are in accordance with the literature.[13]

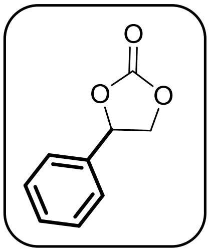

4-Phenyl-1,3-dioxolan-2-one (17). Compound **17** was obtained from 4-fluoro-1,3-dioxolan-2-one (27.0 mg, 0.25 mmol) and diphenylzinc (76.0 mg, 0.35 mmol) neat at 50 °C for 18 hours by following method B. The product was isolated by column purification using 20% ethyl acetate in hexanes as mobile phase as a colorless oil in 58% yield (24 mg, 0.15 mmol). ^{1}H NMR (400 MHz, chloroform-*d*) δ = 7.45 – 7.42 (m, 3H), 7.36 (m, 2H), 5.68 (dd, *J* = 8.0, 8.0 Hz, 1H), 4.80 (dd, *J* = 8.4, 8.4 Hz, 1H), 4.34 (dd, *J* = 8.2, 8.2 Hz, 1H); ^{13}C NMR (100 MHz, chloroform-*d*) δ = 154.78, 135.76, 129.71, 129.21, 125.84, 77.96, 71.14. The spectroscopic data of 4-phenyl-1,3-dioxolan-2-one (**17**) are in accordance with the literature.[14]

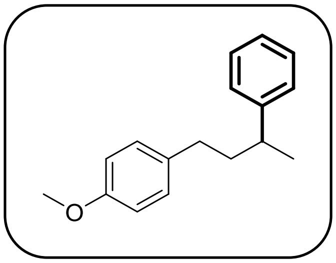

1-Methoxy-4-(3-phenylbutyl)benzene (18). Compound **18** was obtained from 1-(3-fluorobutyl)-4-methoxybenzene (46.0 mg, 0.25 mmol) and diphenylzinc (76.0 mg, 0.35 mmol) neat at 70 °C for 18 hours by following method B. The product was isolated by column purification using 100%

hexanes as mobile phase as a colorless oil in 68% yield (41 mg, 0.16 mmol). ^{1}H NMR (400 MHz, chloroform-*d*) δ = 7.34 (m, 2H), 7.25 – 7.16 (m, 3H), 7.06 (m, 2H), 6.83 (m, 2H), 3.80 (s, 3H), 2.72 (m, 1H), 2.48 (m, 2H), 1.90 (m, 2H), 1.29 (d, *J* = 7.0 Hz, 3H); ^{13}C NMR (100 MHz, chloroform-*d*) δ = 157.62, 147.34, 134.58, 129.20, 128.35, 127.05, 125.90, 113.67, 55.23, 40.19, 39.39, 32.96, 22.49. The reaction gives a mixture of isomers that could not be separated. The given values correspond to the major isomer - 1-methoxy-4-(3-phenylbutyl)benzene. The spectroscopic data of 1-methoxy-4-(3-phenylbutyl)benzene (**18**) are in accordance with the literature.[15]

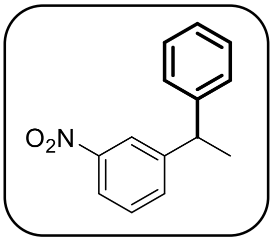

1-Nitro-3-(1-phenylethyl)benzene (19). Compound **19** was obtained from 1-(1-fluoroethyl)-3-nitrobenzene (42.0 mg, 0.25 mmol) and diphenylzinc (76.0 mg, 0.35 mmol) in 1.0 mL of dry $PhCF_3$ at 60 °C for 18 hours by following method A. The product was isolated by column purification using 5% ethyl acetate in hexanes as mobile phase as a brown oil in 75% yield (43 mg, 0.19 mmol). ^{1}H NMR (400 MHz, chloroform-*d*) δ = 8.12 (m, 1H), 8.05 (ddd, *J* = 8.2, 2.3, 1.1 Hz, 1H), 7.54 (m, 1H), 7.44 (m, 1H), 7.32 (m, 2H), 7.26 – 7.19 (m, 3H), 4.27 (q, *J* = 7.2 Hz, 1H), 1.70 (d, *J* = 7.2 Hz, 3H); ^{13}C NMR (100 MHz, chloroform-*d*) δ = 148.44, 148.40, 144.68, 133.96, 129.21, 128.69, 127.48, 126.64, 122.35, 121.24, 44.52, 21.58. The spectroscopic data of 1-nitro-3-(1-phenylethyl)benzene (**19**) are in accordance with the literature.[16]

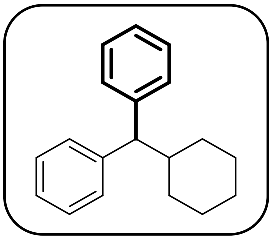

(Cyclohexylmethylene)dibenzene (20). Compound **20** was obtained from (cyclohexylfluoromethyl)benzene (48.0 mg, 0.25 mmol) and diphenylzinc (76.0 mg, 0.35 mmol) in 1.0 mL of dry $PhCF_3$ at 60 °C for 18 hours by following method A. The product was isolated

by column purification using 100% hexanes as mobile phase as a white crystalline solid in 72% yield (45 mg, 0.18 mmol). Melting point range: 47-49 °C. ^{1}H NMR (400 MHz, chloroform-*d*) δ = 7.34 – 7.23 (m, 8H), 7.15 (m, 2H), 3.50 (d, *J* = 10.9 Hz, 1H), 2.14 (m, 1H), 1.77 – 1.56 (m, 5H), 1.35 – 1.10 (m, 3H), 0.90 (m, 2H); ^{13}C NMR (100 MHz, chloroform-*d*) δ = 144.47, 128.36, 128.10, 125.88, 59.56, 41.25, 32.10, 26.55, 26.35. The spectroscopic data of (cyclohexylmethylene)dibenzene (**20**) are in accordance with the literature.[17]

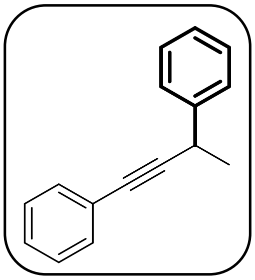

But-1-yne-1,3-diyldibenzene (21). Compound **21** was obtained from (3-fluorobut-1-yn-1-yl)benzene (37.0 mg, 0.25 mmol) and diphenylzinc (76.0 mg, 0.35 mmol) in 1.0 mL of dry $PhCF_3$ at 60 °C for 18 hours by following method A. The product was isolated by column purification using 100% hexanes as mobile phase as a colorless oil in 75% yield (39 mg, 0.19 mmol). ^{1}H NMR (400 MHz, chloroform-*d*) δ = 7.50 – 7.44 (m, 4H), 7.36 (m, 2H), 7.33 – 7.24 (m, 4H), 4.00 (q, *J* = 7.1 Hz, 1H), 1.60 (d, *J* = 7.1 Hz, 3H); ^{13}C NMR (100 MHz, chloroform-*d*) δ = 143.30, 131.60, 128.53, 128.17, 127.71, 126.90, 126.63, 123.72, 92.58, 82.42, 32.46, 24.49. The spectroscopic data of but-1-yne-1,3-diyldibenzene (**21**) are in accordance with the literature.[18]

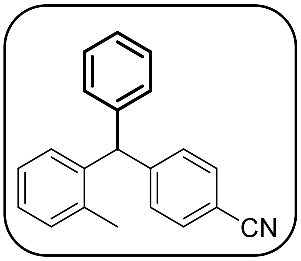

4-(Phenyl(*o*-tolyl)methyl)benzonitrile (22). Compound **22** was obtained from 4-(fluoro(*o*-tolyl)methyl)benzonitrile (56.0 mg, 0.25 mmol) and diphenylzinc (76.0 mg, 0.35 mmol) in 1.0 mL

of dry $PhCF_3$ at 60 °C for 18 hours by following method A. The product was isolated by column purification using 3% ethyl acetate in hexanes as mobile phase as a colorless oil in 70% yield (49 mg, 0.18 mmol). ^{1}H NMR (400 MHz, chloroform-*d*) δ = 7.58 (m, 2H), 7.35 – 7.23 (m, 3H), 7.23 – 7.15 (m, 4H), 7.13 (m, 1H), 7.03 (m, 2H), 6.75 (m, 1H), 5.72 (s, 1H), 2.21 (s, 3H); ^{13}C NMR (100 MHz, chloroform-*d*) δ = 149.21, 141.84, 140.87, 136.51, 132.12, 130.69, 130.31, 129.48, 129.25, 128.60, 126.91, 126.81, 126.01, 118.90, 110.23, 53.51, 19.86. HRMS (ESI-QTOF) *m/z:* $[M+H]^+$ calculated for $C_{21}H_{17}N$ 284.1439, found 284.1436.

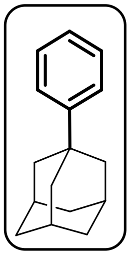

1-Phenyladamantane (23). Compound **23** was obtained from 1-fluoroadamantane (38.0 mg, 0.25 mmol) and diphenylzinc (76.0 mg, 0.35 mmol) in 1.0 mL of dry $PhCF_3$ at 70 °C for 18 hours by following method A. The product was isolated by column purification using 100% hexanes as mobile phase as a white crystalline solid in 91% yield (48 mg, 0.23 mmol). Melting point range: 78-79 °C. ^{1}H NMR (400 MHz, chloroform-*d*) δ = 7.43 – 7.31 (m, 4H), 7.21 (m, 1H), 2.13 (m, 3H), 1.96 (d, J = 2.9 Hz, 6H), 1.81 (m, 6H); ^{13}C NMR (100 MHz, chloroform-*d*) δ = 151.29, 128.08, 128.07, 125.48, 124.81, 43.18, 36.83, 36.17, 28.99. The spectroscopic data of 1-phenyladamantane (**23**) are in accordance with the literature.[19]

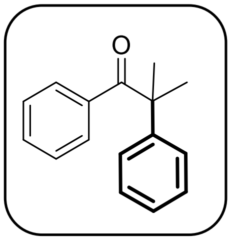

2-Methyl-1,2-diphenylpropane-1-one (24). Compound **24** was obtained from 2-fluoro-2-methyl-1-phenylpropan-1-one (41.0 mg, 0.25 mmol) and diphenylzinc (76.0 mg, 0.35 mmol) in 1.0 mL of dry $PhCF_3$ at 70 °C for 36 hours by following method A. The product was isolated by column purification using 5% ethyl acetate in hexanes as mobile phase as a colorless oil in 81% yield (45

mg, 0.20 mmol). ^{1}H NMR (400 MHz, chloroform-*d*) δ = 7.49 (m, 2H), 7.39 – 7.30 (m, 5H), 7.30 – 7.19 (m, 3H), 1.61 (s, 6H); ^{13}C NMR (100 MHz, chloroform-*d*) δ = 203.66, 145.24, 136.22, 131.60, 129.66, 128.95, 127.89, 126.73, 125.67, 51.38, 27.80. The spectroscopic data of 2-methyl-1,2-diphenylpropane-1-one (**24**) are in accordance with the literature.[20]

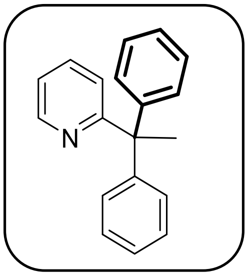

2-(1,1-Diphenylethyl)pyridine (25). Compound **25** was obtained from 2-(1-fluoro-1-phenylethyl)pyridine (54.0 mg, 0.25 mmol) and diphenylzinc (76.0 mg, 0.35 mmol) in 1.0 mL of dry $PhCF_3$ at 60 °C for 18 hours by following method A. The product was isolated by column purification using 3% ethyl acetate in hexanes as mobile phase as a colorless oil in 60% yield (39 mg, 0.15 mmol). ^{1}H NMR (400 MHz, chloroform-*d*) δ = 8.62 (ddd, *J* = 4.8, 1.9, 0.9 Hz, 1H), 7.52 (ddd, *J* = 8.1, 7.5, 1.9 Hz, 1H), 7.31 – 7.23 (m, 4H), 7.23 – 7.16 (m, 2H), 7.14 – 7.06 (m, 5H), 6.97 (m, 1H), 2.24 (s, 3H); ^{13}C NMR (100 MHz, chloroform-*d*) δ = 167.10, 148.84, 148.32, 135.73, 128.62, 127.93, 126.06, 123.60, 120.97, 55.13, 29.40. HRMS (ESI-QTOF) *m/z:* $[M+H]^+$ calculated for $C_{19}H_{17}N$ 260.1439, found 260.1434.

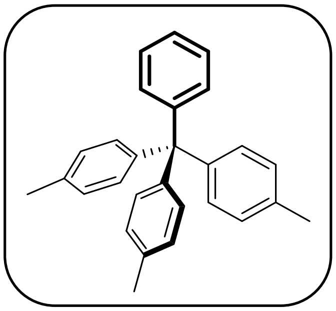

4,4',4''-(Phenylmethanetriyl)tris(methylbenzene) (26). Compound **26** was obtained from of tri(4-tolyl)fluoromethane (76.0 mg, 0.25 mmol) and diphenylzinc (76.0 mg, 0.35 mmol) in 1.0 mL of dry $PhCF_3$ at 60 °C for 18 hours by following method A. The product was isolated by column purification using 100% hexanes as mobile phase as a white crystalline solid in 91% yield (82 mg,

0.23 mmol). Melting point range: 167-170 °C. ^{1}H NMR (400 MHz, chloroform-*d*) δ = 7.26 – 7.14 (m, 5H), 7.13 – 7.03 (m, 12H), 2.32 (s, 9H); ^{13}C NMR (100 MHz, chloroform-*d*) δ = 147.30, 144.18, 135.18, 131.06, 130.96, 128.09, 127.33, 125.66, 63.96, 20.90. HRMS (ESI-QTOF) *m/z:* [M+H]$^+$ calculated for $C_{28}H_{26}$ 363.2113, found 363.2108.

.

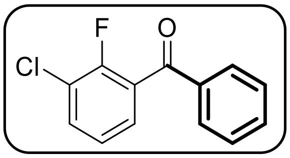

(3-Chloro-2-fluorophenyl)(phenyl)methanone (28). Compound **28** was obtained from 3-chloro-2-fluorobenzoyl fluoride (44.0 mg, 0.25 mmol) and diphenylzinc (76.0 mg, 0.35 mmol) in 1.0 mL of dry $PhCF_3$ at 50 °C for 18 hours by following method A. The product was isolated by column purification using 5% ethyl acetate in hexanes as mobile phase as a colorless oil in 63% yield (37 mg, 0.16 mmol). ^{1}H NMR (400 MHz, chloroform-*d*) δ = 7.83 (d, J = 7.2 Hz, 2H), 7.66 – 7.55 (m, 2H), 7.49 (m, 2H), 7.43 (m, 1H), 7.22 (m, 1H); ^{13}C NMR (100 MHz, chloroform-*d*) δ = 192.20, 155.49 (d, $J_{C\text{-}F}$ = 254.4 Hz), 136.79, 133.75, 133.28, 129.80, 129.53, 128.75 (d, $J_{C\text{-}F}$ = 2.5 Hz), 128.59, 124.75 (d, $J_{C\text{-}F}$ = 4.7 Hz), 121.49; ^{19}F NMR (376 MHz, chloroform-*d*) δ = -113.72. The spectroscopic data of (3-chloro-2-fluorophenyl)(phenyl)methanone (**28**) are in accordance with the literature.[21]

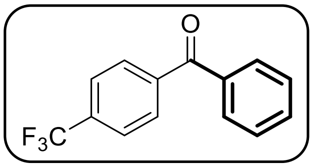

Phenyl(4-(trifluoromethyl)phenyl)methanone (29). Compound **29** was obtained from 4-(trifluoromethyl)benzoyl fluoride (48.0 mg, 0.25 mmol) and diphenylzinc (76.0 mg, 0.35 mmol) in 1.0 mL of dry $PhCF_3$ at 50 °C for 18 hours by following method A. The product was isolated by column purification using 5% ethyl acetate in hexanes as mobile phase as a white crystalline solid in 77% yield (48 mg, 0.19 mmol). Melting point range: 106-108 °C. ^{1}H NMR (400 MHz, chloroform-*d*) δ = 7.90 (m, 2H), 7.82 (m, 2H), 7.76 (m, 2H), 7.63 (m, 1H), 7.51 (dd, J = 8.3, 7.0 Hz, 2H); ^{13}C NMR (100 MHz, chloroform-*d*) δ = 195.49, 140.68 (q, $J_{C\text{-}F}$ = 1.2 Hz), 136.69, 133.70

(q, J_{C-F} = 32.0 Hz), 133.06, 130.11, 130.08, 128.50, 125.31 (q, J_{C-F} = 3.7 Hz), 123.68 (q, J_{C-F} = 271.2 Hz); ^{19}F NMR (376 MHz, chloroform-*d*) δ = -63.05. The spectroscopic data of phenyl(4-(trifluoromethyl)phenyl)methanone (**29**) are in accordance with the literature.[22]

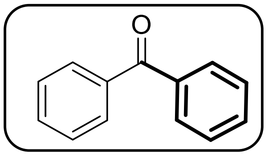

Benzophenone (30). Compound **30** was obtained from benzoyl fluoride (31.0 mg, 0.25 mmol) and diphenylzinc (76.0 mg, 0.35 mmol) in 1.0 mL of dry $PhCF_3$ at 50 ºC for 18 hours by following method A. The product was isolated by column purification using 5% ethyl acetate in hexanes as mobile phase as a white crystalline solid in 80% yield (36 mg, 0.20 mmol). Melting point range: 47-48 °C. ^{1}H NMR (400 MHz, chloroform-*d*) δ = 7.81 (m, 4H), 7.59 (m, 2H), 7.49 (m, 4H); ^{13}C NMR (100 MHz, chloroform-*d*) δ = 196.70, 137.57, 132.38, 130.02, 128.24. The spectroscopic data of benzophenone (**30**) are in accordance with the literature.[23]

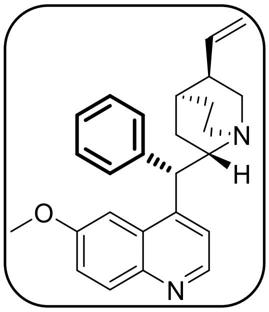

(1*S*,2*S*,4*S*,5*R*)-2-((*S*)-(6-Methoxyquinolin-4-yl)(phenyl)methyl)-5-vinylquinuclidene (71). Compound **71** was obtained from (1*S*,2*S*,4*S*,5*R*)-2-((*S*)-fluoro(6-methoxyquinolin-4-yl)methyl)-5-vinylquinuclidene (82.0 mg, 0.25 mmol) and diphenylzinc (76.0 mg, 0.35 mmol) in 1.0 mL of dry $PhCF_3$ at 60 ºC for 18 hours by following method A. The product was isolated by column purification using 5% methanol in dichloromethane as mobile phase as an amorphous white solid in 65% yield (62 mg, 0.16 mmol). Melting point range: 135-137 °C. ^{1}H NMR (400 MHz, chloroform-*d*) δ = 8.75 (d, *J* = 4.6 Hz, 1H), 7.99 (d, *J* = 9.2 Hz, 1H), 7.50 (bs, 1H), 7.45 – 7.29 (m, 4H), 7.29 – 7.18 (m, 3H), 7.14 (m, 1H), 5.96 (m, 1H), 5.07 (m, 2H), 4.75 (d, *J* = 11.2 Hz, 1H),

3.95 (s, 3H), 3.72 (bs, 1H), 3.30 (m, 1H), 3.23 (dd, J = 13.9, 10.1 Hz, 1H), 2.75 (m, 2H), 2.28 (m, 1H), 1.91 (m, 1H), 1.70 (m, 1H), 1.58 (m, 2H), 0.84 (m, 1H); ^{13}C NMR (100 MHz, chloroform-*d*) δ = 157.70, 147.62, 146.71, 144.76, 142.09, 141.98, 131.91, 128.76, 128.49, 127.86, 126.70, 120.91, 119.68, 114.31, 102.06, 59.53, 59.47, 56.54, 55.48, 49.45, 40.93, 39.56, 28.80, 28.05. HRMS (ESI-QTOF) *m/z:* $[M+H]^+$ calculated for $C_{26}H_{28}N_2O$ 385.2280, found 385.2273. For single crystal X-ray analysis, compound (**71**) was titrated with aqueous 1 M HCl, and the HCl salt was crystallized via slow evaporation of a saturated acetone solution.

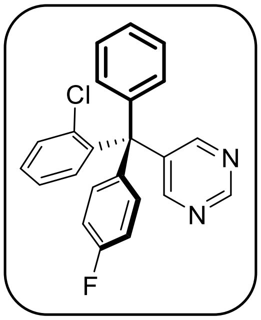

5-((2-Chlorophenyl)(4-fluorophenyl)(phenyl)methyl)pyrimidine (72). Compound **72** was obtained from 5-((2-chlorophenyl)fluoro(4-fluorophenyl)methyl)pyrimidine (79.0 mg, 0.25 mmol) and diphenylzinc (76.0 mg, 0.35 mmol) in 1.0 mL of dry $PhCF_3$ at 60 °C for 18 hours by following method A. The product was isolated by column purification using 20% ethyl acetate in hexanes as mobile phase as a yellow oil in 82% yield (77 mg, 0.21 mmol). ^{1}H NMR (400 MHz, chloroform-*d*) δ = 8.51 (m, 2H), 8.43 (m, 2H), 7.52 – 7.41 (m, 4H), 7.26 – 7.20 (m, 2H), 7.12 – 7.01 (m, 4H), 6.95 (m, 1H), 5.95 (s, 1H); ^{13}C NMR (100 MHz, chloroform-*d*) δ = 163.08, 163.02, 160.62, 157.85, 139.25, 137.12, 136.06 (d, $J_{C\text{-}F}$ = 3.3 Hz), 134.28, 133.05, 130.75, 130.74, 130.03 (d, $J_{C\text{-}F}$ = 26.9 Hz), 129.71 (d, $J_{C\text{-}F}$ = 215.7 Hz), 128.62, 128.00, 127.09, 115.76 (d, $J_{C\text{-}F}$ = 21.4 Hz), 48.05; ^{19}F NMR (376 MHz, chloroform-*d*) δ = -115.11. HRMS (ESI-QTOF) *m/z:* $[M+H]^+$ calculated for $C_{23}H_{16}N_2ClF$ 375.1064, found 375.1061.

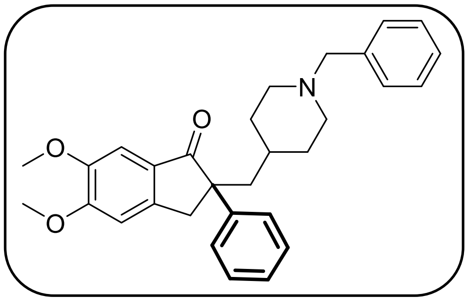

2-((1-Benzylpiperidin-4-yl)methyl)-5,6-dimethoxy-2-phenyl-2,3-dihydro-1H-inden-1-one (73). Compound **73** was obtained from 2-((1-benzylpiperidin-4-yl)methyl)-2-fluoro-5,6-dimethoxy-2,3-dihydro-1H-inden-1-one (99.0 mg, 0.25 mmol) and diphenylzinc (76.0 mg, 0.35 mmol) in 1.0 mL of dry $PhCF_3$ at 90 ºC for 18 hours by following method A. The product was isolated by column purification using 5% methanol in dichloromethane as mobile phase as a tan colored oil in 75% yield (85 mg, 0.19 mmol). ^{1}H NMR (400 MHz, methylene chloride-*d₂*) δ = 7.50 (m, 2H), 7.40 – 7.28 (m, 5H), 7.22 – 7.03 (m, 3H), 6.83 (s, 1H), 6.65 (s, 1H), 3.81 (s, 3H), 3.68 (s, 3H), 3.41 (d, *J* = 15.8 Hz, 1H), 3.14 (d, *J* = 15.8 Hz, 1H), 3.05 (bs, 2H), 2.27 (bs, 2H), 2.09 (dd, *J* = 14.6, 5.0 Hz, 2H), 1.92 (m, 1H), 1.89 – 1.70 (m, 2H), 1.62 – 1.58 (m, 4H); ^{13}C NMR (100 MHz, methylene chloride-d_2) δ = 148.80, 148.42, 146.99, 142.15, 139.47, 132.46, 130.75, 129.37, 128.67, 128.13, 127.72, 127.67, 126.67, 125.80, 110.17, 108.78, 86.70, 71.63, 55.95, 55.83, 44.84, 42.20, 32.31. HRMS (ESI-QTOF) *m/z:* $[M+H]^+$ calculated for $C_{30}H_{33}NO_3$ 456.2539, found 456.2534.

.

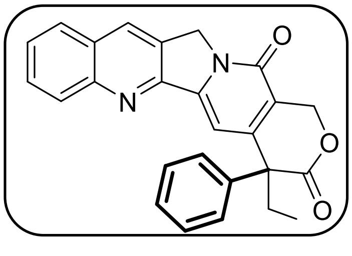

4-Ethyl-4-phenyl-1,12-dihydro-14H-pyrano[3',4':6,7]indolizino[1,2-b]quinoline-3,14(4H)-dione (47). Compound **47** was obtained from 4-ethyl-4-fluoro-1,12-dihydro-14H-pyrano[3',4':6,7]indolizino[1,2-b]quinoline-3,14(4H)-dione (88.0 mg, 0.25 mmol) and diphenylzinc (76.0 mg, 0.35 mmol) in 1.0 mL of dry $PhCF_3$ at 60 ºC for 18 hours by following method A. The product was isolated by column purification using 5% methanol in

dichloromethane as mobile phase as a white amorphous solid in 61% yield (62 mg, 0.16 mmol). Melting point range: 252 °C decomposition. ^{1}H NMR (400 MHz, chloroform-*d*) δ = 8.33 (s, 1H), 8.17 (m, 1H), 7.90 (m, 1H), 7.77 (m, 3H), 7.61 (m, 1H), 7.55 (s, 1H), 7.44 – 7.36 (m, 3H), 5.24 (s, 2H), 5.08 (d, *J* = 17.7 Hz, 1H), 5.02 (d, *J* = 17.7 Hz, 1H), 1.66 (m, 2H), 0.79 (t, *J* = 7.6 Hz, 3H); ^{13}C NMR (100 MHz, chloroform-*d*) δ = 157.95, 152.95, 148.84, 145.87, 145.64, 143.75, 139.66, 130.86, 130.33, 129.64, 128.87, 128.41, 127.99, 127.60, 126.98, 124.62, 99.28, 96.05, 95.04, 93.12, 60.92, 49.68, 30.33, 7.91. HRMS (ESI-QTOF) *m/z:* $[M+H]^+$ calculated for $C_{26}H_{20}N_2O_3$ 409.1552, found 409.1548.

5.4. C-F Bond Functionalization with Other Diarylzinc Reagents

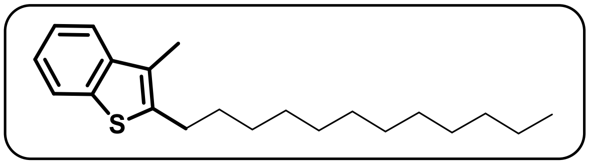

2-Dodecyl-3-methylbenzo[b]thiophene (51). Compound **51** was obtained from 1-fluorododecane (47.0 mg, 0.25 mmol) and bis(3-methylbenzo[b]thiophen-2-yl)zinc • THF (150.0 mg, 0.35 mmol) in 0.1 mL of dry $PhCF_3$ at 120 ºC for 3 days by following method A. The product was isolated by column purification using 100% hexanes as mobile phase as a colorless oil in 66% yield (52 mg, 0.17 mmol). ^1H NMR (400 MHz, chloroform-*d*) δ = 7.75 (m, 1H), 7.59 (m, 1H), 7.33 (m, 1H), 7.25 (m, 1H), 2.84 (t, *J* = 7.6 Hz, 2H), 2.30 (s, 3H), 1.69 (m, 2H), 1.42 – 1.06 (m, 18H), 0.88 (t, *J* = 6.6 Hz, 3H); ^{13}C NMR (100 MHz, chloroform-*d*) δ = 140.98, 139.93, 138.11, 126.37, 123.67, 123.34, 122.07, 121.12, 31.91, 31.26, 29.64, 29.63, 29.61, 29.55, 29.42, 29.34, 29.24, 28.45, 22.68, 14.11, 11.48. The spectroscopic data of 2-dodecyl-3-methylbenzo[b]thiophene (**51**) are in accordance with the literature.[24]

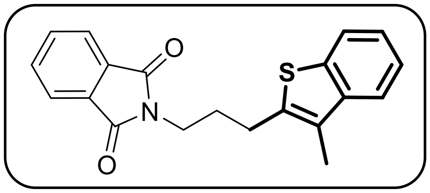

2-(3-(3-Methylbenzo[b]thiophen-2-yl)propyl)isoindoline-1,3-dione (52). Compound **52** was obtained from 2-(3-fluoropropyl)isoindoline-1,3-dione (52.0 mg, 0.25 mmol) and bis(3-methylbenzo[b]thiophen-2-yl)zinc • THF (150.0 mg, 0.35 mmol) in 0.1 mL of dry $PhCF_3$ at 70 ºC for 18 hours by following method A. The product was isolated by column purification using 5% ethyl acetate in hexanes as mobile phase as a colorless oil in 72% yield (60 mg, 0.18 mmol). ^1H NMR (400 MHz, chloroform-*d*) δ = 7.88 (m, 1H), 7.78 (d, *J* = 7.7 Hz, 1H), 7.69 (d, *J* = 7.8 Hz, 1H), 7.55 – 7.45 (m, 3H), 7.36 (m, 2H), 4.55 (m, 1H), 4.24 (t, *J* = 12.1 Hz, 1H), 4.10 (m, 1H), 3.35 (bs, 1H), 2.57 (bs, 3H), 1.94 (m, 1H), 1.56 (m, 1H); ^{13}C NMR (100 MHz, chloroform-*d*) δ = 167.09, 141.66, 138.62, 135.11, 132.60, 130.13, 129.76, 124.93, 124.14, 123.84, 122.32, 121.91, 63.77, 36.34, 24.64, 11.79. HRMS (ESI-QTOF) *m/z:* $[M+H]^+$ calculated for $C_{20}H_{17}O_2S$ 336.1058, found 336.1056.

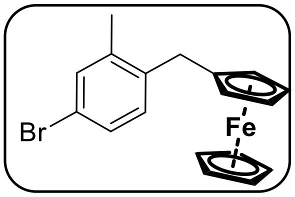

(4-Bromo-2-methylbenzyl)ferrocene (53). Compound **53** was obtained from 4-bromo-1-(fluoromethyl)-2-methylbenzene (51.0 mg, 0.25 mmol) and diferrocenylzinc (152.0 mg, 0.35 mmol) in 1.0 mL of dry DCE at 90 °C for 18 hours by following method A. The product was isolated by column purification using 100% hexanes as mobile phase, followed by sublimation of excess ferrocene to yield an orange oil in 71% yield (65 mg, 0.18 mmol). ^{1}H NMR (400 MHz, chloroform-*d*) δ = 7.26 (s, 1H), 7.21 (m, 1H), 6.92 (d, *J* = 8.1 Hz, 1H), 4.14 (s, 5H), 4.09 (m, 2H), 4.05 (m, 2H), 3.62 (s, 2H), 2.28 (s, 3H); ^{13}C NMR (100 MHz, chloroform-*d*) δ = 138.97, 137.95, 132.54, 130.38, 128.74, 119.56, 86.60, 68.79, 68.68, 67.50, 32.90, 19.38. HRMS (ESI-QTOF) *m/z:* $[M]^+$ calculated for $C_{18}H_{17}BrFe$ 367.9863, found 367.9860. DCE was used due to the limited solubility of diferrocenylzinc in $PhCF_3$.

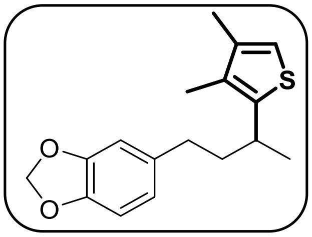

5-(3-(3,4-Dimethylthiophen-2-yl)butyl)benzo[d][1,3]dioxole (54). Compound **54** was obtained from 5-(3-fluorobutyl)benzo[d][1,3]dioxole (49.0 mg, 0.25 mmol) and bis(3,4-dimethylthiophen-2-yl)zinc • 2 THF (150.0 mg, 0.35 mmol) neat at 120 °C for 18 hours by following method B. The product was isolated by column purification using 2% ethyl acetate in hexanes as mobile phase as a yellow oil in 70% yield (50 mg, 0.18 mmol). ^{1}H NMR (400 MHz, chloroform-*d*) δ = 6.74 (m, 1H), 6.71 (m, 1H), 6.64 (m, 1H), 6.58 (m, 1H), 5.91 (s, 2H), 3.06 (m, 1H), 2.50 (m, 2H), 2.14 (s, 3H), 2.01 (s, 3H), 1.86 (m, 2H), 1.26 (d, *J* = 6.9 Hz, 3H); ^{13}C NMR (100 MHz, chloroform-*d*) δ = 147.44, 145.43, 144.96, 137.53, 136.15, 131.70, 121.01, 116.45, 108.82, 108.02, 100.66, 41.03,

33.58, 33.09, 23.12, 15.22, 12.30. HRMS (ESI-QTOF) *m/z:* $[M+H]^+$ calculated for $C_{17}H_{20}O_2S$ 289.1262, found 289.1251.

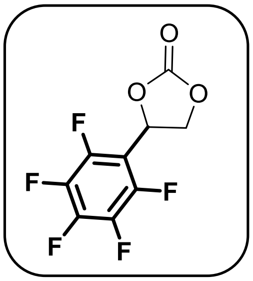

4-(Perfluorophenyl)-1,3-dioxolan-2-one (55). Compound **55** was obtained from 4-fluoro-1,3-dioxolan-2-one (27.0 mg, 0.25 mmol) and bis(pentafluorophenyl)zinc (139.0 mg, 0.35 mmol) neat at 50 ºC for 18 hours by following method B. The product was isolated by column purification using 20% ethyl acetate in hexanes as mobile phase as a colorless oil in 68% yield (43 mg, 0.17 mmol). ^{1}H NMR (400 MHz, chloroform-*d*) δ = 6.02 (m, 1H), 4.86 (m, 1H), 4.53 (m, 1H); ^{13}C NMR (100 MHz, chloroform-*d*) δ = 153.52, 145.45 (dm, J_{C-F} = 252.8 Hz), 142.57 (dm, J_{C-F} = 259.2 Hz), 137.85 (dm, J_{C-F} = 257.2 Hz), 109.85, 68.25, 67.59; ^{19}F NMR (376 MHz, chloroform-*d*) δ = -142.17 (m), -149.09 (m), -159.45 (m). HRMS (ESI-QTOF) *m/z:* $[M+H]^+$ calculated for $C_9H_3F_5O_3$ 255.0081, found 255.0079.

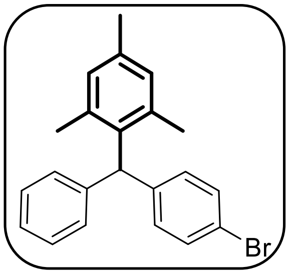

2-((4-Bromophenyl)(phenyl)methyl)-1,3,5-trimethylbenzene (56). Compound **56** was obtained from 1-bromo-4-(fluoro(phenyl)methyl)benzene (66.0 mg, 0.25 mmol) and dimesitylzinc (106.0 mg, 0.35 mmol) in 1.0 mL of dry $PhCF_3$ at 60 ºC for 18 hours by following method A. The product was isolated by column purification using 100% hexanes as mobile phase as a white crystalline solid in 80% yield (73 mg, 0.20 mmol). Melting point range: 160-161 °C. ^{1}H NMR (400 MHz, chloroform-*d*) δ = 7.61 (m, 1H), 7.30 – 7.17 (m, 4H), 7.12 (m, 1H), 7.01 – 6.95 (m, 3H), 6.85 (s,

2H), 6.07 (s, 1H), 2.29 (s, 3H), 1.97 (s, 6H); ^{13}C NMR (100 MHz, chloroform-*d*) δ = 142.23, 141.74, 137.60, 136.17, 136.12, 132.94, 131.63, 130.19, 129.00, 128.27, 127.96, 127.22, 126.11, 125.98, 52.21, 22.03, 20.79. HRMS (ESI-QTOF) *m/z:* [M-H]$^+$ calculated for $C_{22}H_{20}Br$ 363.0743, found 363.0742.

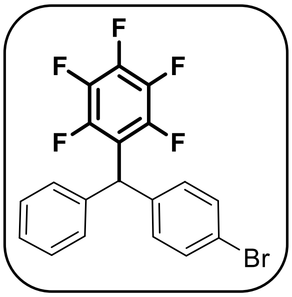

1-((4-Bromophenyl)(phenyl)methyl)-2,3,4,5,6-pentafluorobenzene (57). Compound **57** was obtained from 1-bromo-4-(fluoro(phenyl)methyl)benzene (66.0 mg, 0.25 mmol) and bis(pentafluorophenyl)zinc (139.0 mg, 0.35 mmol) in 1.0 mL of dry $PhCF_3$ at 60 °C for 18 hours by following method A. The product was isolated by column purification using 100% hexanes as mobile phase as a colorless oil in 44% yield (45 mg, 0.11 mmol). ^{1}H NMR (400 MHz, chloroform-*d*) δ = 7.58 (m, 1H), 7.39 – 7.30 (m, 3H), 7.22 (m, 1H), 7.19 – 7.10 (m, 3H), 7.03 (m, 1H), 6.09 (s, 1H); ^{13}C NMR (100 MHz, chloroform-*d*) δ = 145.35 (dm, J_{C-F} = 252.5 Hz), 140.29 (dm, J_{C-F} = 253.5 Hz), 139.64, 138.56, 137.50 (dm, J_{C-F} = 253.3 Hz), 133.03, 131.24 (dd, J_{C-F} = 2.7 Hz), 128.78 (dd, J_{C-F} = 9.5 Hz), 127.41, 127.26, 124.70, 115.80, 46.38; ^{19}F NMR (376 MHz, chloroform-*d*) δ = δ -139.50 (m), -155.50 (t, J = 21.1 Hz), -161.02 (m). HRMS (ESI-QTOF) *m/z:* [M-H]$^+$ calculated for $C_{19}H_9BrF_5$ 410.9802, found 410.9804.

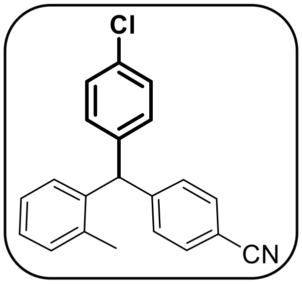

4-((4-Chlorophenyl)(o-tolyl)methyl)benzonitrile (58). Compound **58** was obtained from 4-(fluoro(*o*-tolyl)methyl)benzonitrile (56.0 mg, 0.25 mmol) and bis(4-chlorophenyl)zinc · THF

(126.0 mg, 0.35 mmol) in 1.0 mL of dry $PhCF_3$ at 60 °C for 18 hours by following method A. The product was isolated by column purification using 3% ethyl acetate in hexanes as mobile phase as a colorless oil in 52% yield (41 mg, 0.13 mmol). ^{1}H NMR (400 MHz, chloroform-*d*) δ = 7.60 (m, 2H), 7.29 (m, 2H), 7.20 – 7.12 (m, 5H), 6.95 (m, 2H), 6.71 (m, 1H), 5.68 (s, 1H), 2.19 (s, 3H); ^{13}C NMR (100 MHz, chloroform-*d*) δ = 148.62, 140.38, 140.34, 136.43, 132.73, 132.22, 130.80, 130.75, 130.20, 129.09, 128.77, 127.12, 126.13, 118.75, 110.50, 52.84, 19.80. HRMS (ESI-QTOF) *m/z:* $[M+H]^+$ calculated for $C_{21}H_{16}ClN$ 318.1050, found 318.1049.

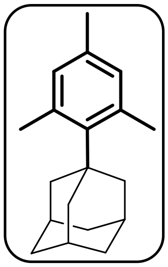

1-Mesityladamantane (59). Compound **59** was obtained from 1-fluoroadamantane (38.0 mg, 0.25 mmol) and dimesitylzinc (106.0 mg, 0.35 mmol) in 1.0 mL of dry $PhCF_3$ at 70 °C for 18 hours by following method A. The product was isolated by column purification using 100% hexanes as mobile phase as a white crystalline solid in 75% yield (48 mg, 0.19 mmol). Melting point range: 104-105 °C. ^{1}H NMR (400 MHz, chloroform-*d*) δ = 6.77 (s, 2H), 2.61 (s, 6H), 2.31 (m, 6H), 2.19 (s, 3H), 2.08 (m, 3H), 1.77 (m, 6H); ^{13}C NMR (100 MHz, chloroform-*d*) δ = 143.38, 136.69, 134.25, 133.01, 42.47, 41.52, 36.97, 29.48, 26.55, 20.07. HRMS (ESI-QTOF) *m/z:* $[M+H]^+$ calculated for $C_{19}H_{26}$ 255.2113, found 255.2111.

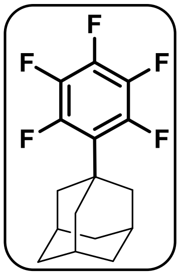

1-(Perfluorophenyl)adamantane (60). Compound **60** was obtained from 1-fluoroadamantane (38.0 mg, 0.25 mmol) and bis(pentafluorophenyl)zinc (139.0 mg, 0.35 mmol) in 1.0 mL of dry

$PhCF_3$ at 70 ºC for 18 hours by following method A. The product was isolated by column purification using 100% hexanes as mobile phase as a white crystalline solid in 52% yield (39 mg, 0.13 mmol). Melting point range: 104-106 °C. ^{1}H NMR (400 MHz, chloroform-*d*) δ = 2.23 (m, 6H), 2.07 (m, 3H), 1.79 (m, 6H); ^{13}C NMR (100 MHz, chloroform-*d*) δ = 146.20 (dm, $J_{C\text{-}F}$ = 247.4 Hz), 138.95 (dm, $J_{C\text{-}F}$ = 251.7 Hz), 137.90 (dm, $J_{C\text{-}F}$ = 246.5 Hz), 122.38, 41.08, 40.42, 36.46, 28.77; ^{19}F NMR (376 MHz, chloroform-*d*) δ = -138.15 (m), -158.56 (m), -163.08 (m). The spectroscopic data of 1-(perfluorophenyl)adamantane (**60**) are in accordance with the literature.[25]

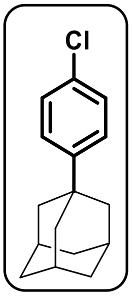

1-(4-Chlorophenyl)adamantane (61). Compound **61** was obtained from 1-fluoroadamantane (38.0 mg, 0.25 mmol) and bis(4-chlorophenyl)zinc • THF (126.0 mg, 0.35 mmol) in 1.0 mL of dry $PhCF_3$ at 70 ºC for 18 hours by following method A. The product was isolated by column purification using 100% hexanes as mobile phase as a white crystalline solid in 71% yield (44 mg, 0.18 mmol). Melting point range: 85-87 °C. ^{1}H NMR (400 MHz, chloroform-*d*) δ = 7.32 – 7.25 (m, 4H), 2.11 (m, 3H), 1.89 (m, 6H), 1.78 (m, 6H); ^{13}C NMR (100 MHz, chloroform-*d*) δ = 149.78, 131.08, 128.08, 126.33, 43.08, 36.65, 35.95, 28.84. The spectroscopic data of 1-(4-chlorophenyl)adamantane (**61**) are in accordance with the literature.[26]

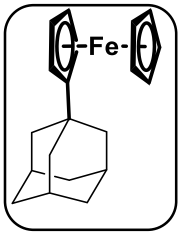

1-Ferrocenyladamantane (62). Compound **62** was obtained from 1-fluoroadamantane (38.0 mg, 0.25 mmol) and diferrocenylzinc (152.0 mg, 0.35 mmol) in 1.0 mL of dry DCE at 90 ºC for 18 hours by following method A. The product was isolated by column purification using 100%

hexanes as mobile phase, followed by sublimation of excess ferrocene to yield an orange solid in 59% yield (47 mg, 0.15 mmol). Melting point range: 121-122 °C. ^{1}H NMR (400 MHz, chloroform-*d*) δ = 4.16 (s, 5H), 4.07 (m, 2H), 4.03 (m, 2H), 2.02 (bs, 3H), 1.81 (m, 6H), 1.73 (m, 6H); ^{13}C NMR (100 MHz, chloroform-*d*) δ = 102.27, 68.02, 66.52, 64.10, 43.97, 37.07, 31.96, 28.82. HRMS (ESI-QTOF) *m/z:* $[M]^+$ calculated for $C_{20}H_{24}Fe$ 320.1227, found 320.1224. DCE was used due to the limited solubility of diferrocenylzinc in $PhCF_3$. Single crystals were grown from evaporation of a saturated pentane solution.

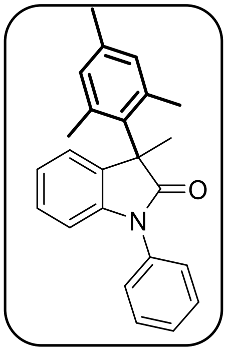

3-Mesityl-3-methyl-1-phenylindolin-2-one (63). Compound **63** was obtained from 3-fluoro-3-methyl-1-phenylindolin-2-one (60.0 mg, 0.25 mmol) and dimesitylzinc (106.0 mg, 0.35 mmol) in 1.0 mL of dry $PhCF_3$ at 60 ºC for 18 hours by following method A. The product was isolated by column purification using 5% ethyl acetate in hexanes as mobile phase as a white crystalline solid in 68% yield (58 mg, 0.17 mmol). Melting point range: 148-152 °C. ^{1}H NMR (400 MHz, chloroform-*d*) δ = 7.59 – 7.51 (m, 2H), 7.50 – 7.45 (m, 2H), 7.42 (m, 1H), 7.19 (m, 1H), 7.13 (m, 1H), 7.03 (m, 1H), 6.88 (m, 1H), 6.84 (s, 2H), 2.50 (bs, 6H), 2.23 (s, 3H), 2.05 (s, 3H); ^{13}C NMR (100 MHz, chloroform-*d*) δ = 180.46, 142.02, 137.96, 136.38, 135.76, 134.80, 133.55, 131.92, 129.59, 127.92, 127.43, 126.24, 123.34, 123.24, 109.50, 55.28, 29.71, 20.40. HRMS (ESI-QTOF) *m/z:* $[M+H]^+$ calculated for $C_{23}H_{24}NO$ 342.1858, found 342.1855. Single crystals were grown from evaporation of a saturated pentane solution.

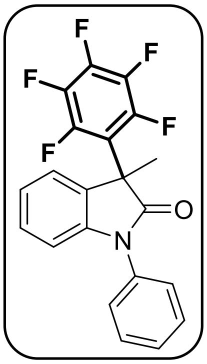

3-Methyl-3-(perfluorophenyl)-1-phenylindolin-2-one (64). Compound **64** was obtained from 3-fluoro-3-methyl-1-phenylindolin-2-one (60.0 mg, 0.25 mmol) and bis(pentafluorophenyl)zinc (139.0 mg, 0.35 mmol) in 1.0 mL of dry $PhCF_3$ at 60 ºC for 18 hours by following method A. The product was isolated by column purification using 5% ethyl acetate in hexanes as mobile phase as a white crystalline solid in 59% yield (57 mg, 0.15 mmol). Melting point range: 116-117 °C. ^{1}H NMR (400 MHz, chloroform-*d*) δ = 7.57 (m, 2H), 7.50 – 7.42 (m, 3H), 7.23 (m, 1H), 7.17 (m, 1H), 7.07 (m, 1H), 6.86 (m, 1H), 2.03 (t, $J_{C\text{-}F}$ = 3.2 Hz, 3H); ^{13}C NMR (100 MHz, chloroform-*d*) δ = 177.26 , 146.35 (dm, $J_{C\text{-}F}$ = 252.7 Hz), 142.59, 140.39 (dm, $J_{C\text{-}F}$ = 260.6 Hz), 137.92 (dm, $J_{C\text{-}F}$ = 251.4 Hz), 134.18, 132.72, 129.74, 128.73, 128.42, 126.61, 123.54, 123.20, 114.41, 109.99, 49.80, 24.34 (t, $J_{C\text{-}F}$ = 6.4 Hz); ^{19}F NMR (376 MHz, chloroform-*d*) δ = -138.88 (m), -154.77 (m), -161.58 (m). HRMS (ESI-QTOF) *m/z:* $[M+H]^+$ calculated for $C_{21}H_{12}F_5NO$ 390.0917, found 390.0915.

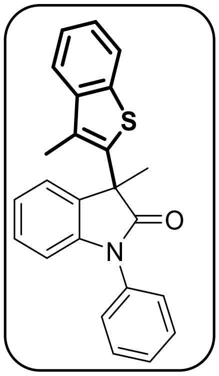

3-Methyl-3-(3-methylbenzo[b]thiophen-2-yl)-1-phenylindolin-2-one (65). Compound **65** was obtained from 3-fluoro-3-methyl-1-phenylindolin-2-one (60.0 mg, 0.25 mmol) and bis(3-methylbenzo[b]thiophen-2-yl)zinc · THF (150.0 mg, 0.35 mmol) in 1.0 mL of dry $PhCF_3$ at 60 ºC

for 18 hours by following method A. The product was isolated by column purification using 5% ethyl acetate in hexanes as mobile phase as a white amorphous solid in 63% yield (58 mg, 0.16 mmol). Melting point range: 134-138 °C. ^{1}H NMR (400 MHz, chloroform-*d*) δ = 7.82 (m, 1H), 7.62 – 7.52 (m, 3H), 7.53 – 7.47 (m, 2H), 7.44 (m, 1H), 7.33 (m, 2H), 7.22 (m, 2H), 7.08 (m, 1H), 6.94 (m, 1H), 2.06 (s, 3H), 2.02 (s, 3H); ^{13}C NMR (100 MHz, chloroform-*d*) δ = 177.27, 142.42, 141.36, 138.13, 137.50, 134.46, 134.06, 129.66, 129.50, 128.45, 128.14, 126.39, 124.04, 123.97, 123.95, 123.54, 121.95, 121.49, 109.58, 50.13, 26.31, 11.71. The spectroscopic data of 3-methyl-3-(3-methylbenzo[b]thiophen-2-yl)-1-phenylindolin-2-one (**65**) are in accordance with the literature.[27]

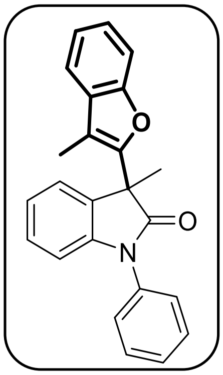

3-Methyl-3-(3-methylbenzofuran-2-yl)-1-phenylindolin-2-one (66). Compound **66** was obtained from 3-fluoro-3-methyl-1-phenylindolin-2-one (60.0 mg, 0.25 mmol) and bis(3-methylbenzofuran-2-yl)zinc • THF (140.0 mg, 0.35 mmol) in 1.0 mL of dry $PhCF_3$ at 60 °C for 18 hours by following method A. The product was isolated by column purification using 5% ethyl acetate in hexanes as mobile phase as a white amorphous solid in 51% yield (45 mg, 0.13 mmol). Melting point range: 155-158 °C. ^{1}H NMR (400 MHz, chloroform-*d*) δ = 7.56 (m, 2H), 7.52 – 7.40 (m, 5H), 7.33 – 7.18 (m, 4H), 7.09 (m, 1H), 6.91 (m, 1H), 2.06 (s, 3H), 1.95 (s, 3H); ^{13}C NMR (100 MHz, chloroform-*d*) δ = 176.38, 153.56, 149.69, 142.56, 134.42, 132.97, 130.65, 129.67, 128.27, 128.18, 126.50, 124.06, 123.87, 123.49, 122.20, 119.01, 111.07, 110.94, 109.64, 49.66, 22.59, 7.90. The spectroscopic data of 3-methyl-3-(3-methylbenzofuran-2-yl)-1-phenylindolin-2-one (**62**) are in accordance with the literature.[27] X-ray quality single crystals were grown from evaporation of a saturated ethyl acetate solution.

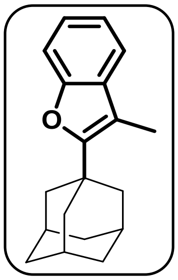

2-(Adamantan-1-yl)-3-methylbenzofuran (67). Compound **67** was obtained from 1-fluoroadamantane (38.0 mg, 0.25 mmol) and bis(3-methylbenzofuran-2-yl)zinc • THF (140.0 mg, 0.35 mmol) in 1.0 mL of dry $PhCF_3$ at 70 ºC for 18 hours by following method A. The product was isolated by column purification using 100% hexanes as mobile phase as a white crystalline solid in 71% yield (47 mg, 0.18 mmol). Melting point range: 99-102 °C. ^{1}H NMR (400 MHz, chloroform-*d*) δ = 7.43 (m, 1H), 7.36 (m, 1H), 7.23 – 7.15 (m, 2H), 2.33 (s, 3H), 2.16 (m, 6H), 2.09 (m, 3H), 1.80 (m, 6H); ^{13}C NMR (100 MHz, chloroform-*d*) δ = 159.53, 152.73, 131.54, 122.91, 121.58, 118.39, 110.32, 107.29, 40.92, 36.77, 36.68, 28.35, 8.98. HRMS (ESI-QTOF) *m/z:* $[M+H]^+$ calculated for $C_{19}H_{22}O$ 267.1749, found 267.1738.

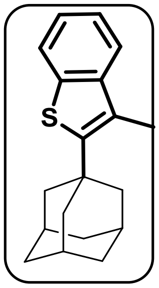

2-(Adamantan-1-yl)-3-methylbenzo[b]thiophene (68). Compound **68** was obtained from 1-fluoroadamantane (38.0 mg, 0.25 mmol) and bis(3-methylbenzo[b]thiophen-2-yl)zinc • THF (150.0 mg, 0.35 mmol) in 1.0 mL of dry $PhCF_3$ at 70 ºC for 18 hours by following method A. The product was isolated by column purification using 100% hexanes as mobile phase as a white crystalline solid in 76% yield (54 mg, 0.19 mmol). Melting point range: 110-114 °C. ^{1}H NMR (400 MHz, chloroform-*d*) δ = 7.78 (m, 1H), 7.64 (m, 1H), 7.35 (m, 1H), 7.27 (m, 1H), 2.55 (s, 3H), 2.22 (m, 6H), 2.12 (m, 3H), 1.81 (m, 6H); ^{13}C NMR (100 MHz, chloroform-*d*) δ = 149.57, 142.34, 136.82, 124.66, 123.53, 123.18, 121.83, 120.80, 42.43, 37.28, 36.67, 28.92, 13.97. HRMS (ESI-QTOF) *m/z:* $[M+H]^+$ calculated for $C_{19}H_{22}S$ 283.1520, found 283.1520.

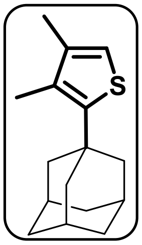

2-(Adamantan-1-yl)-3,4-dimethylthiophene (69). Compound **69** was obtained from 1-fluoroadamantane (38.0 mg, 0.25 mmol) and bis(3,4-dimethylthiophen-2-yl)zinc · 2 THF (150.0 mg, 0.35 mmol) in 1.0 mL of dry $PhCF_3$ at 90 ºC for 18 hours by following method A. The product was isolated by column purification using 100% hexanes as mobile phase as a brown solid in 98% yield (60 mg, 0.24 mmol). Melting point range: 88-92 °C. ^{1}H NMR (400 MHz, chloroform-*d*) δ = 6.69 (s, 1H), 2.25 (s, 3H), 2.12 (m, 3H), 2.11 – 2.04 (m, 9H), 1.78 (m, 6H); ^{13}C NMR (100 MHz, chloroform-*d*) δ = 148.87, 139.26, 130.54, 115.77, 42.54, 42.27, 36.71, 28.90, 15.64, 14.62. HRMS (ESI-QTOF) *m/z:* $[M+H]^+$ calculated for $C_{16}H_{22}S$ 247.1520, found 247.1520.

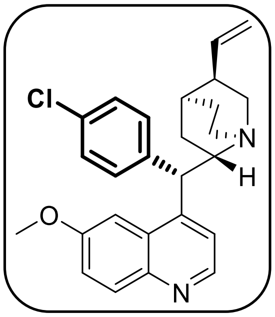

(1S,2S,4S,5R)-2-((S)-(4-chlorophenyl)(6-methoxyquinolin-4-yl)methyl)-5-vinylquinuclidine (74). Compound **74** was obtained from (1*S*,2*S*,4*S*,5*R*)-2-((*S*)-fluoro(6-methoxyquinolin-4-yl)methyl)-5-vinylquinuclidene (82.0 mg, 0.25 mmol) and bis(4-chlorophenyl)zinc · THF (126.0 mg, 0.35 mmol) in 1.0 mL of dry $PhCF_3$ at 60 ºC for 18 hours by following method A. The product was isolated by column purification using 5% methanol in dichloromethane as mobile phase as an amorphous white solid in 49% yield (51 mg, 0.13 mmol). Melting point range: 144-147 °C. ^{1}H NMR (400 MHz, acetonitrile-*d3*) δ = 9.47 (s, 1H), 8.89 (s, 1H), 8.65 (d, *J* = 9.3 Hz, 1H), 8.22 (s, 1H), 7.84 (m, 1H), 7.66 (dd, *J* = 9.4, 2.4 Hz, 2H), 7.31 (d, *J* = 7.9 Hz, 2H), 6.12 (m, 1H), 5.35 (m, 2H), 5.26 (m, 1H), 4.77 (m, 1H), 4.09 (s, 3H), 3.97 (m, 1H), 3.65 (m, 1H), 3.29 – 3.11 (m, 2H),

2.92 (m, 1H), 2.38 (m, 1H), 2.12 (m, 1H), 2.09 (m, 1H), 2.05 (m, 1H), 1.31 (m, 1H); ^{13}C NMR (100 MHz, acetonitrile-*d3*) δ = 160.29, 153.45, 139.85, 139.77, 138.78, 138.63, 134.79, 134.36, 134.29, 130.83, 130.00, 129.53, 126.56, 123.52, 103.44, 60.61, 56.61, 48.70, 41.39, 36.51, 29.33, 27.23, 26.38, 23.37. HRMS (ESI-QTOF) *m/z:* $[M+H]^+$ calculated for $C_{26}H_{27}ClN_2O$ 419.1890, found 419.1886. For single crystal X-ray analysis, compound (**74**) was titrated with aqueous 1 M HCl, and the HCl salt was crystallized via evaporation of a saturated methanol solution.

6. ^{1}H, ^{13}C and ^{19}F NMR Spectra

Supplementary Fig. 38. ^{1}H NMR (400 MHz, $CDCl_3$) spectrum of tetraphenylmethane (4).

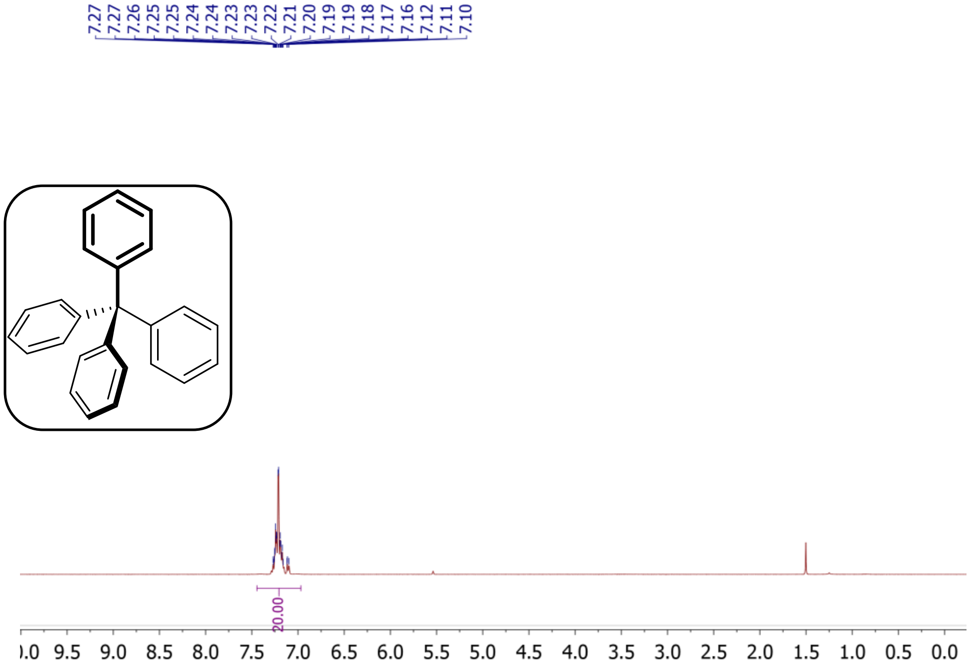

Supplementary Fig. 39. ^{13}C NMR (100 MHz, $CDCl_3$) spectrum of tetraphenylmethane (4).

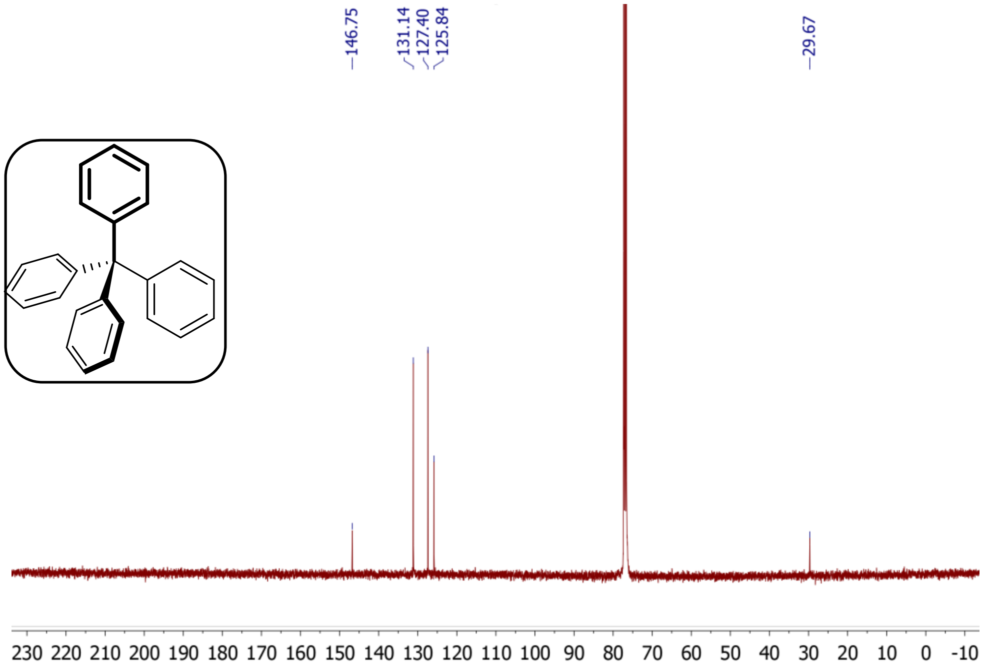

Supplementary Fig. 40. ^{1}H NMR (400 MHz, $CDCl_3$) spectrum of 3-methyl-1,3-diphenylindolin-2-one (6)

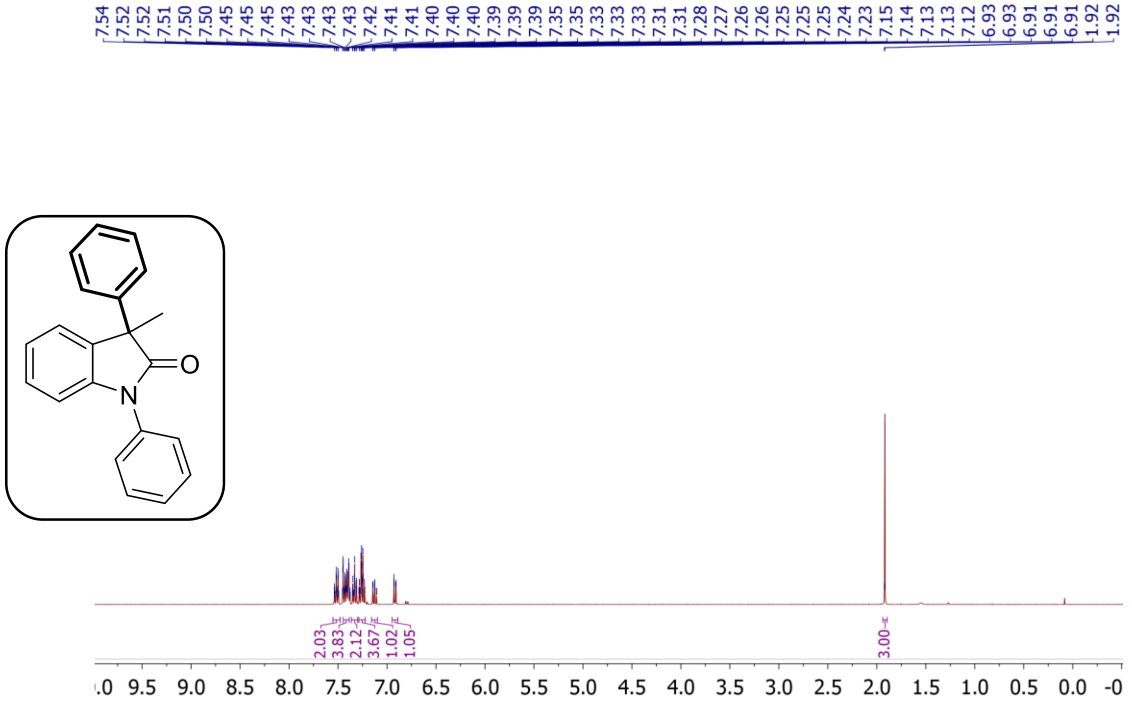

Supplementary Fig. 41. ^{13}C NMR (100 MHz, $CDCl_3$) spectrum of 3-methyl-1,3-diphenylindolin-2-one (6)

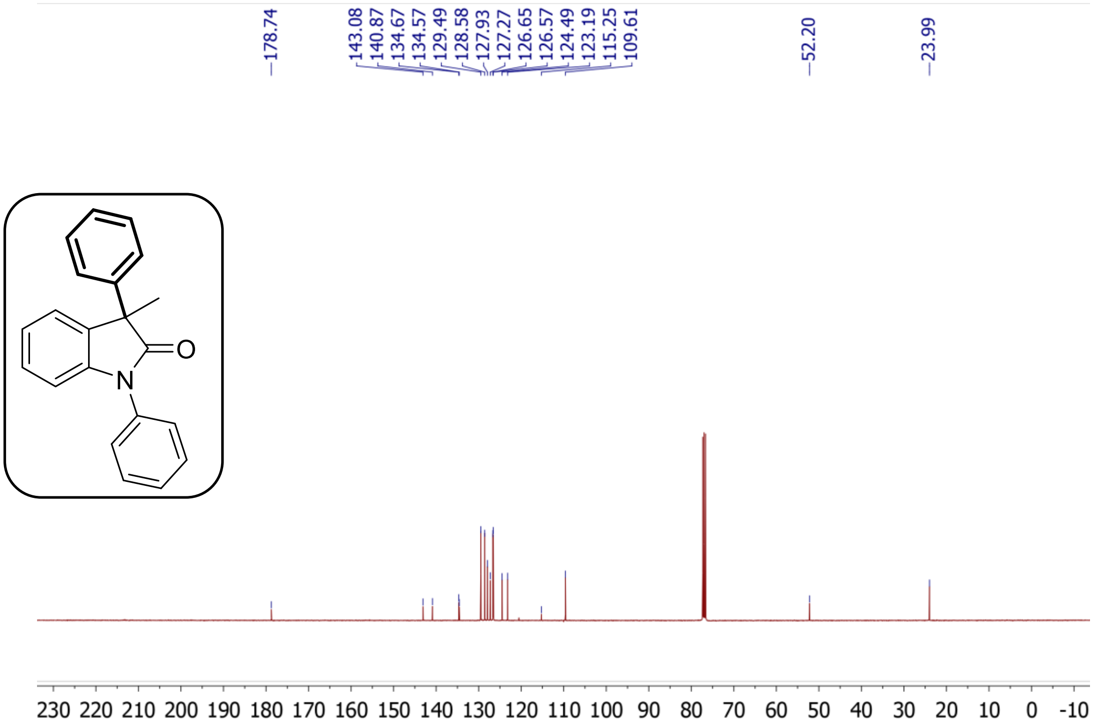

Supplementary Fig. 42. ¹H NMR (400 MHz, $CDCl_3$) spectrum of 1,3-diphenylbutane (10).

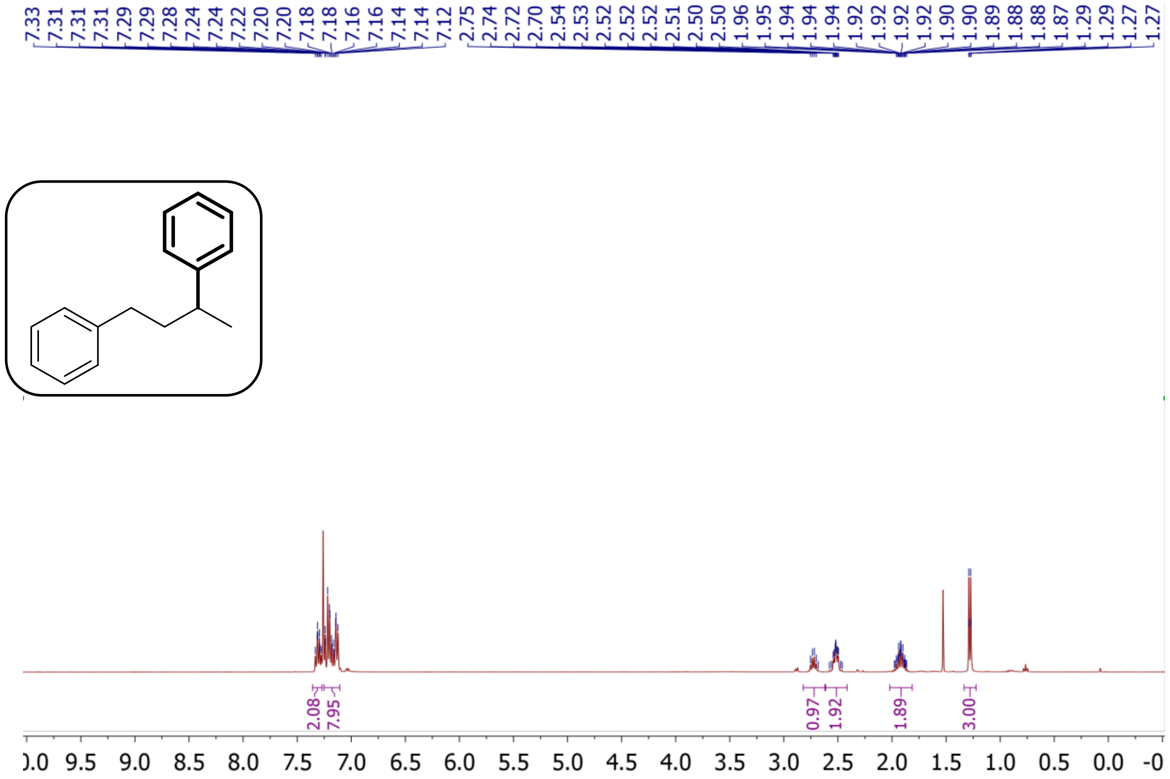

Supplementary Fig. 43. ¹³C NMR (100 MHz, $CDCl_3$) spectrum of 1,3-diphenylbutane (10).

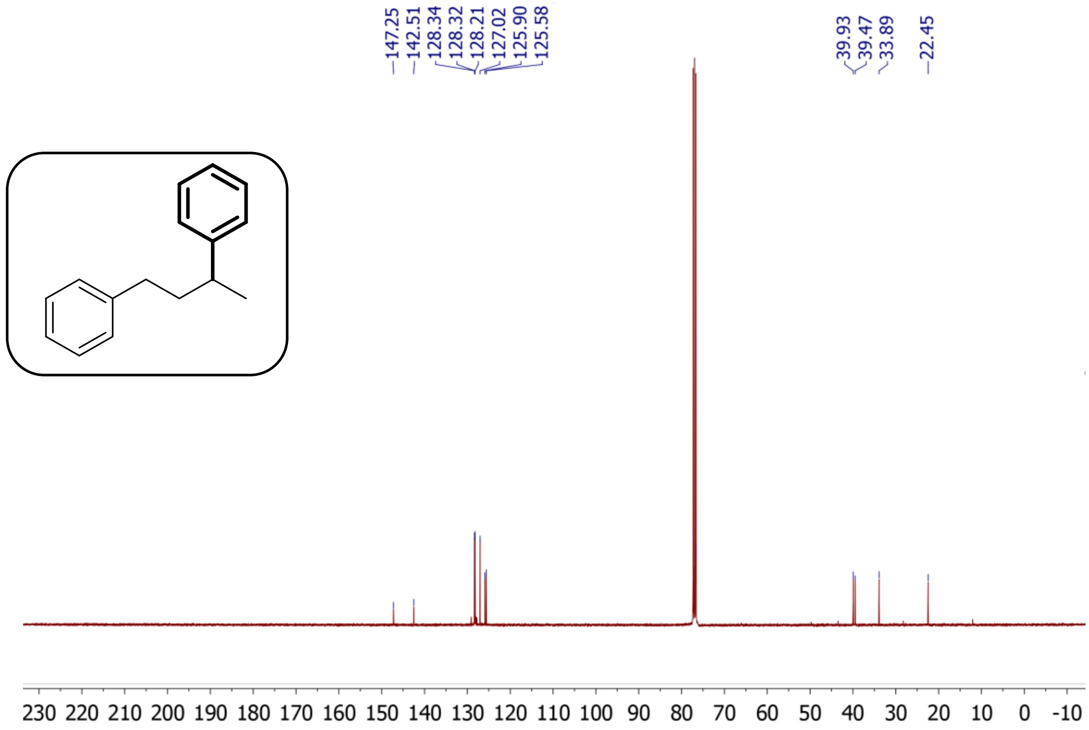

Supplementary Fig. 44. ^{1}**H NMR (400 MHz, CDCl$_3$) spectrum of octylbenzene (11).**

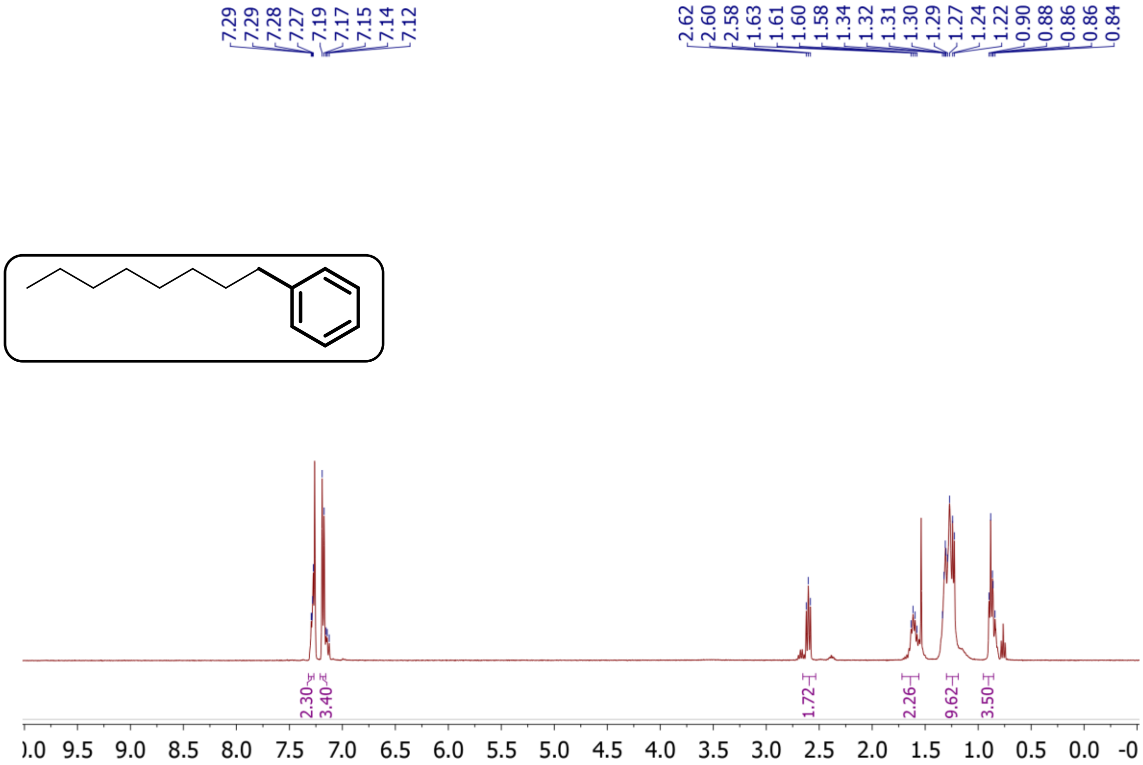

Supplementary Fig. 45. ^{13}**C NMR (100 MHz, CDCl$_3$) spectrum of octylbenzene (11).**

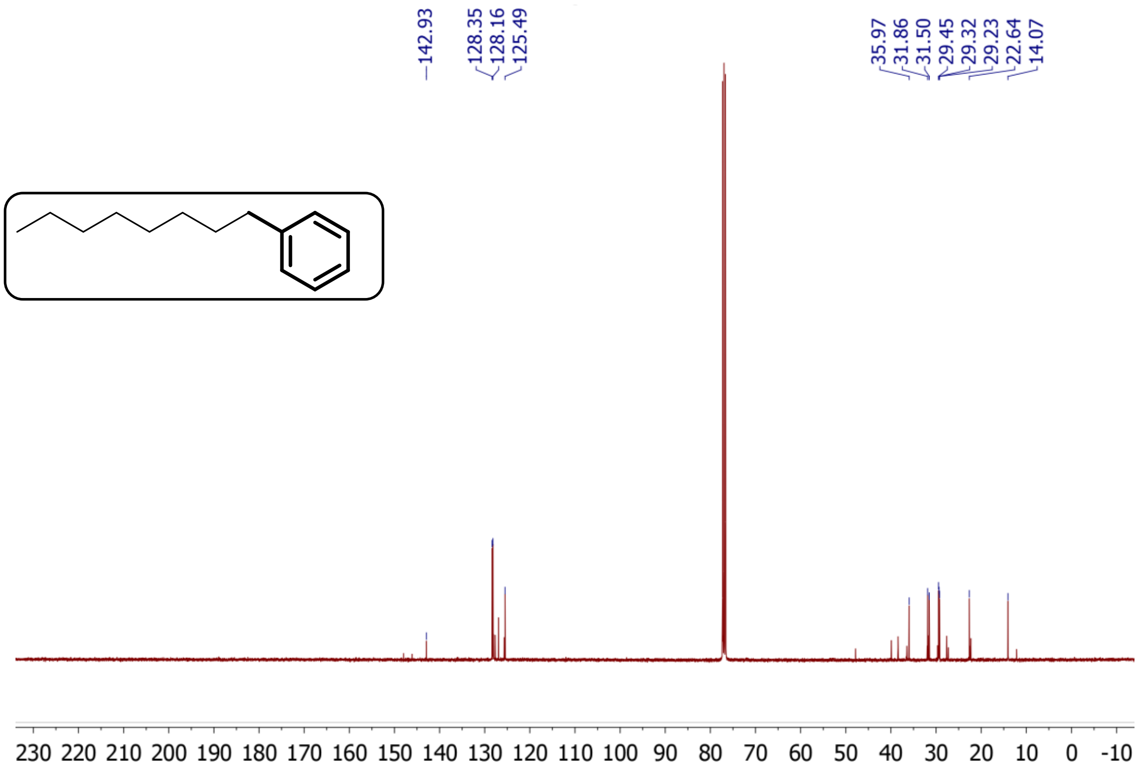

Supplementary Fig. 46. ^{1}H NMR (400 MHz, CDCl$_3$) spectrum of dodecylbenzene (14).

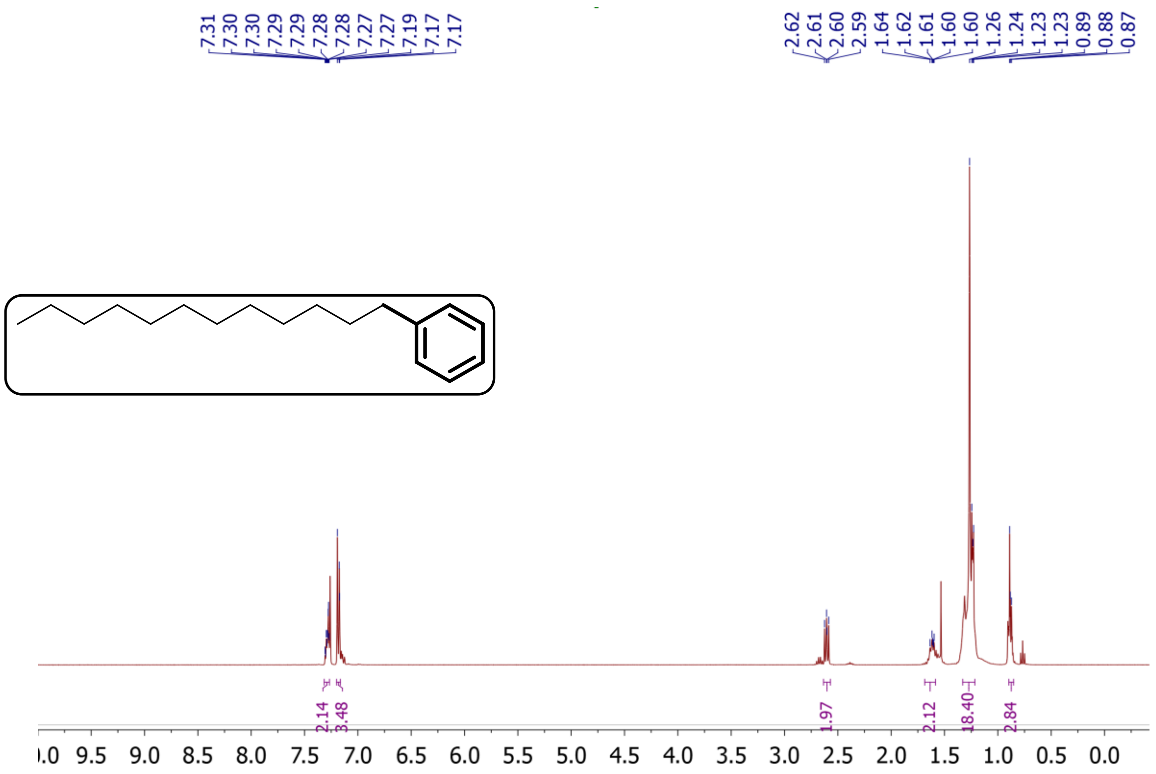

Supplementary Fig. 47. ^{13}C NMR (100 MHz, CDCl$_3$) spectrum of dodecylbenzene (14).

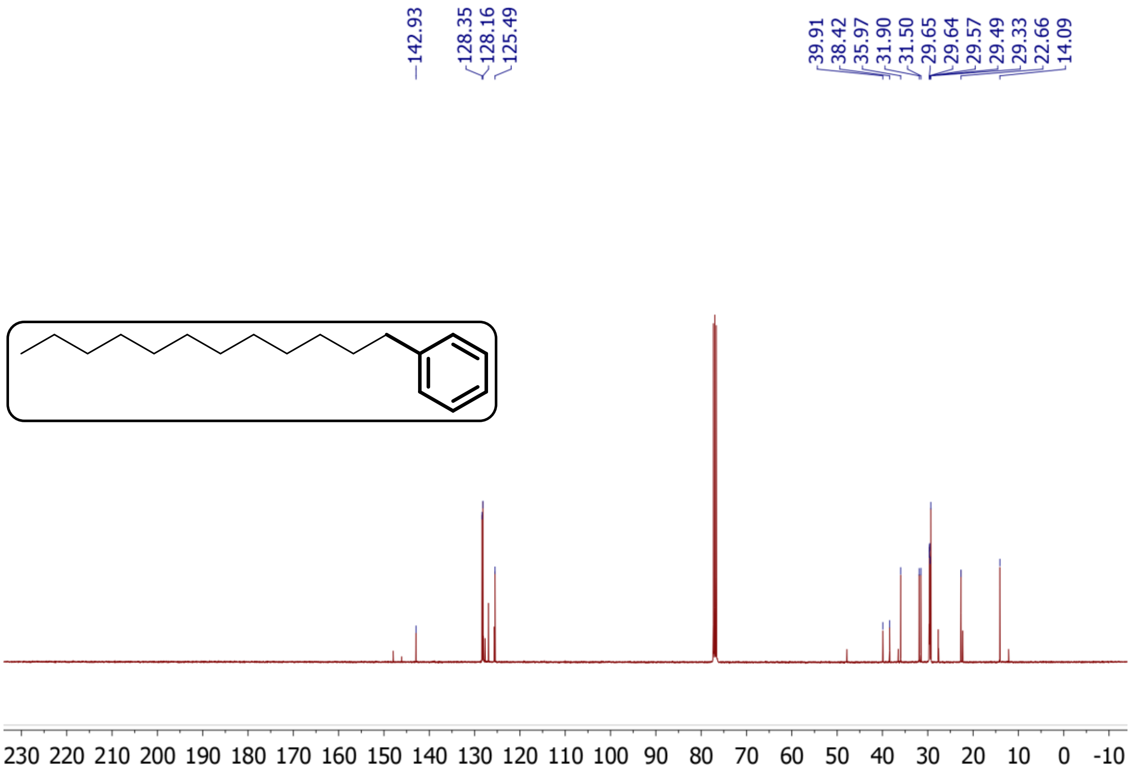

Supplementary Fig. 48. ^{1}H NMR (400 MHz, $CDCl_3$) spectrum of dec-2-yn-1-ylbenzene (15).

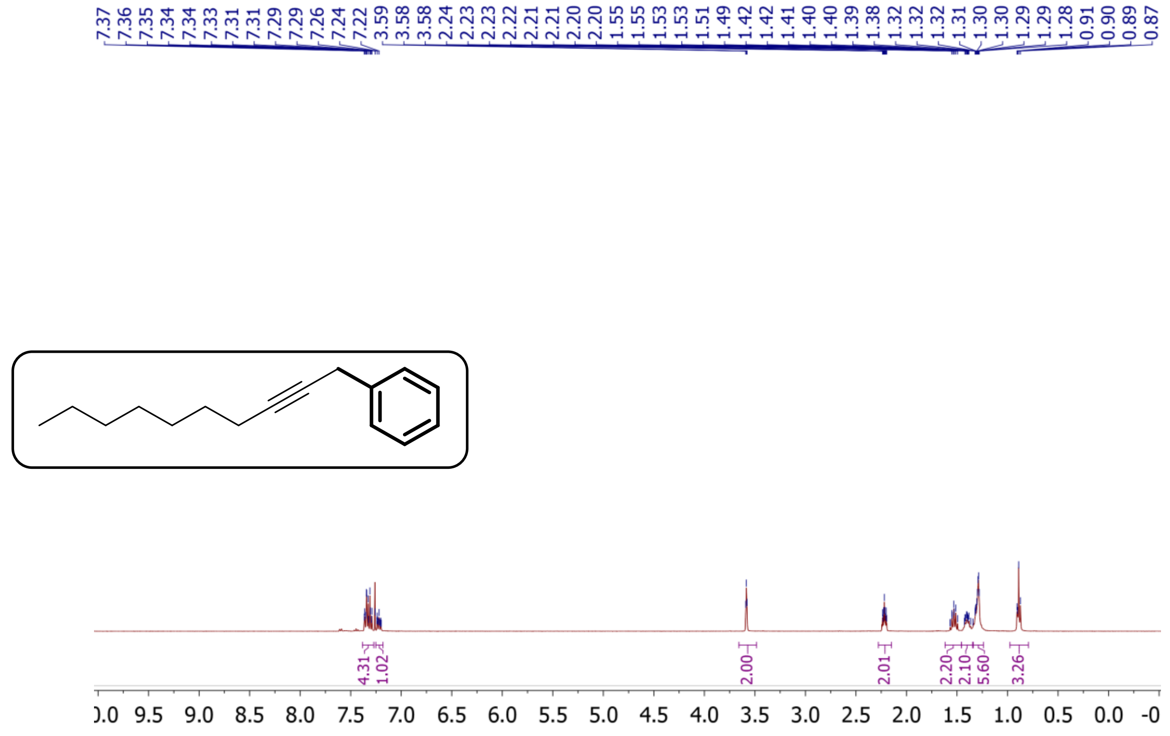

Supplementary Fig. 49. ^{13}C NMR (100 MHz, $CDCl_3$) spectrum of dec-2-yn-1-ylbenzene (15).

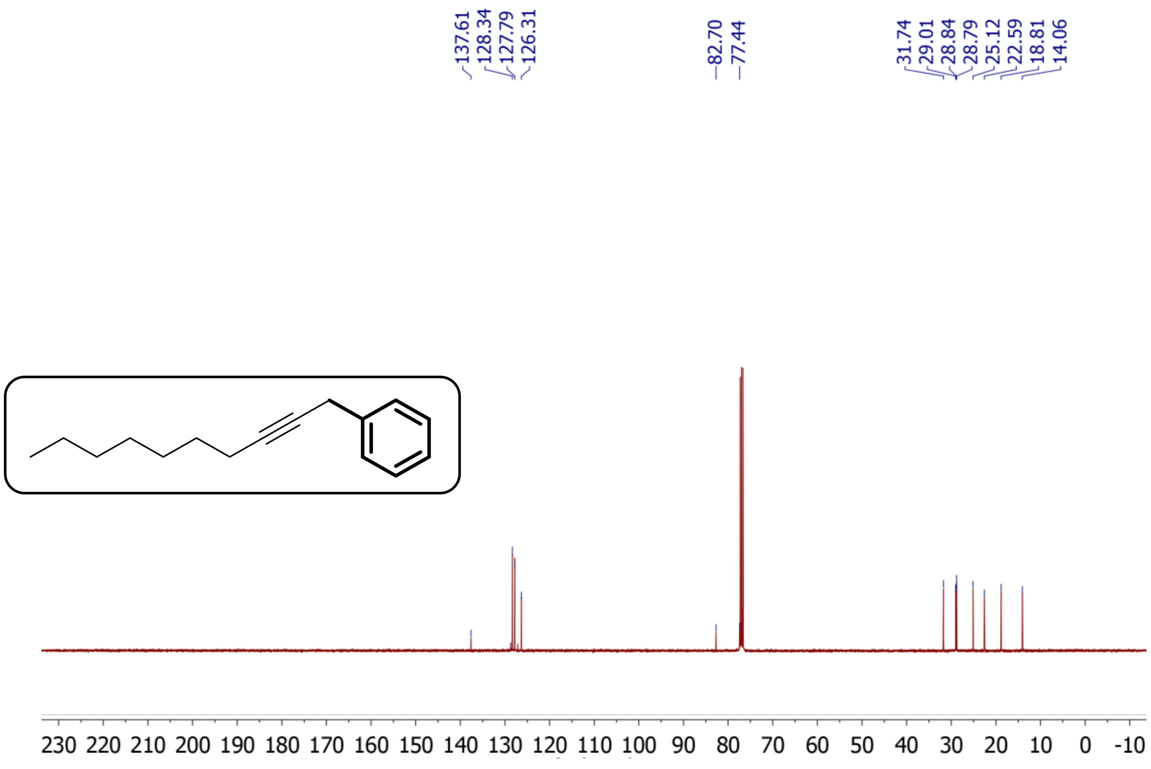

Supplementary Fig. 50. ^{1}H NMR (400 MHz, $CDCl_3$) spectrum of 1-benzyl-4-nitrobenzene (16).

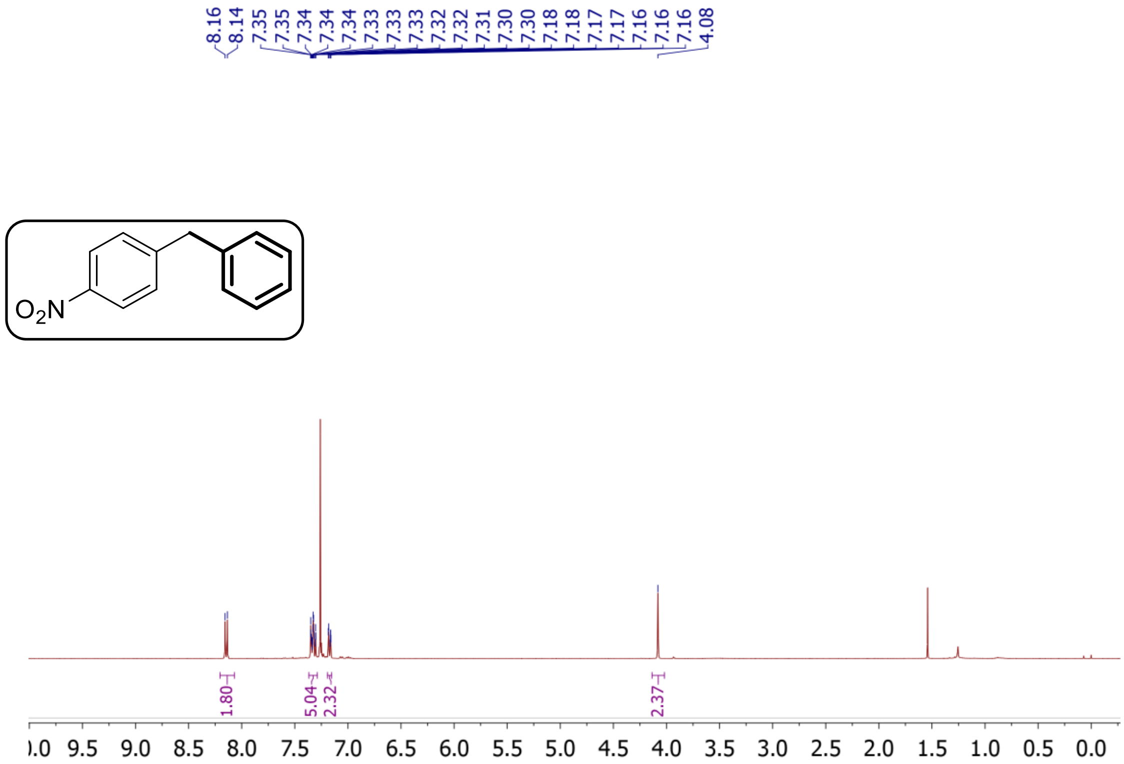

Supplementary Fig. 51. ^{13}C NMR (100 MHz, $CDCl_3$) spectrum of 1-benzyl-4-nitrobenzene (16).

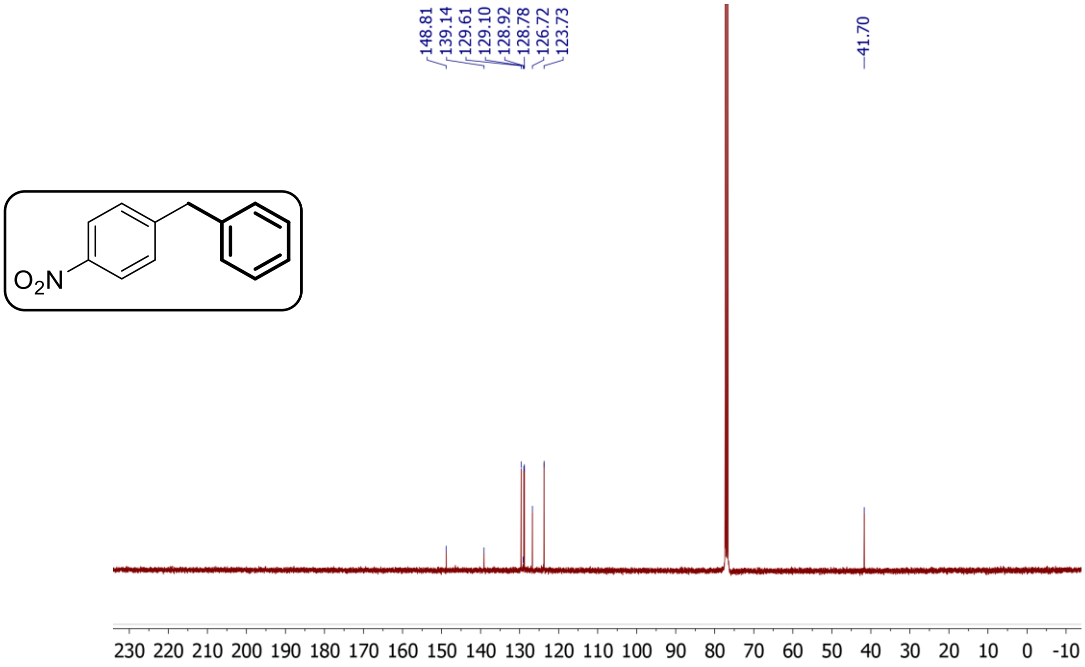

Supplementary Fig. 52. ^{1}H NMR (400 MHz, $CDCl_3$) spectrum of 4-phenyl-1,3-dioxolan-2-one (17).

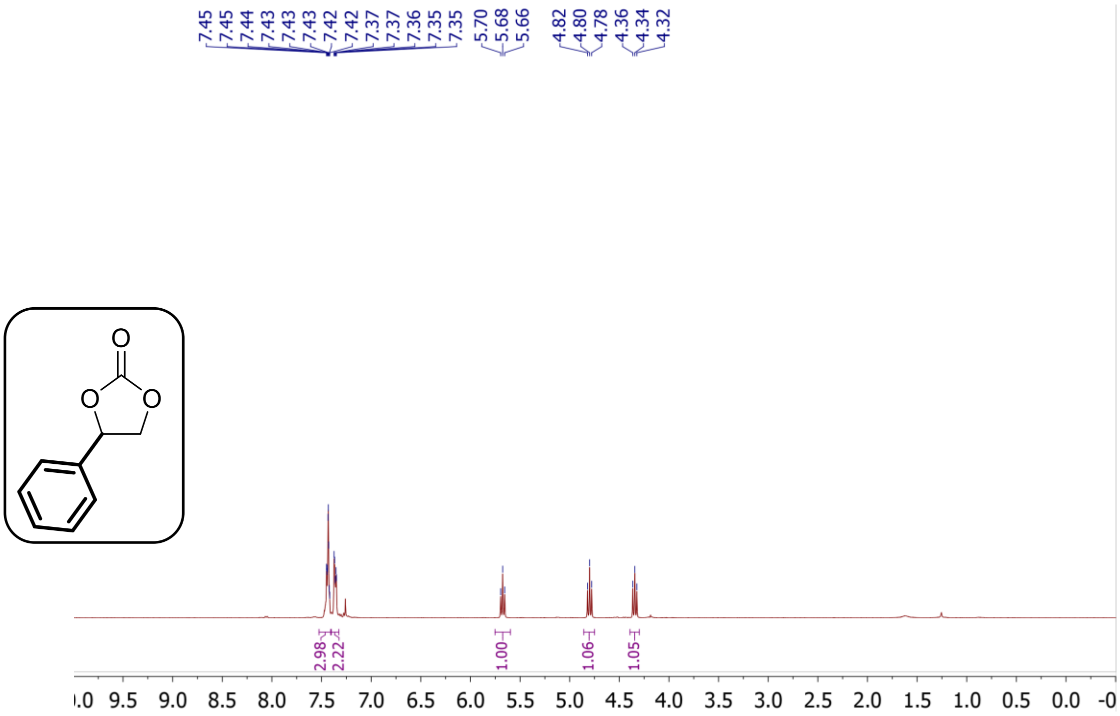

Supplementary Fig. 53. ^{13}C NMR (100 MHz, $CDCl_3$) spectrum of 4-phenyl-1,3-dioxolan-2-one (17).

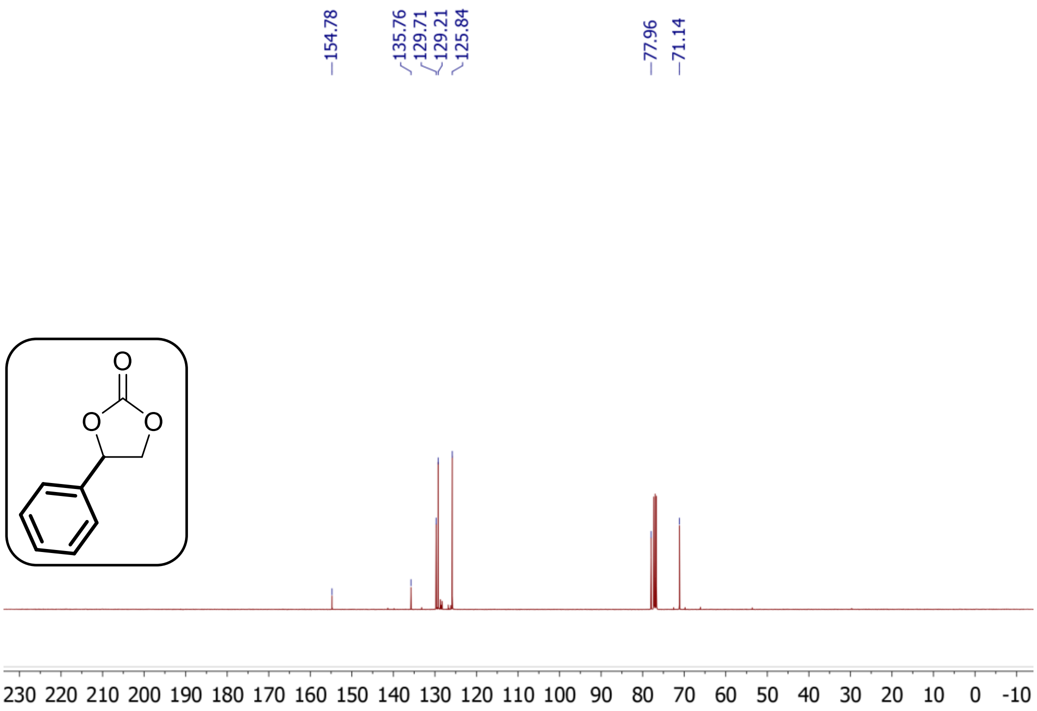

Supplementary Fig. 54. ^{1}H NMR (400 MHz, $CDCl_3$) spectrum of 1-methoxy-4-(3-phenylbutyl)benzene (18).

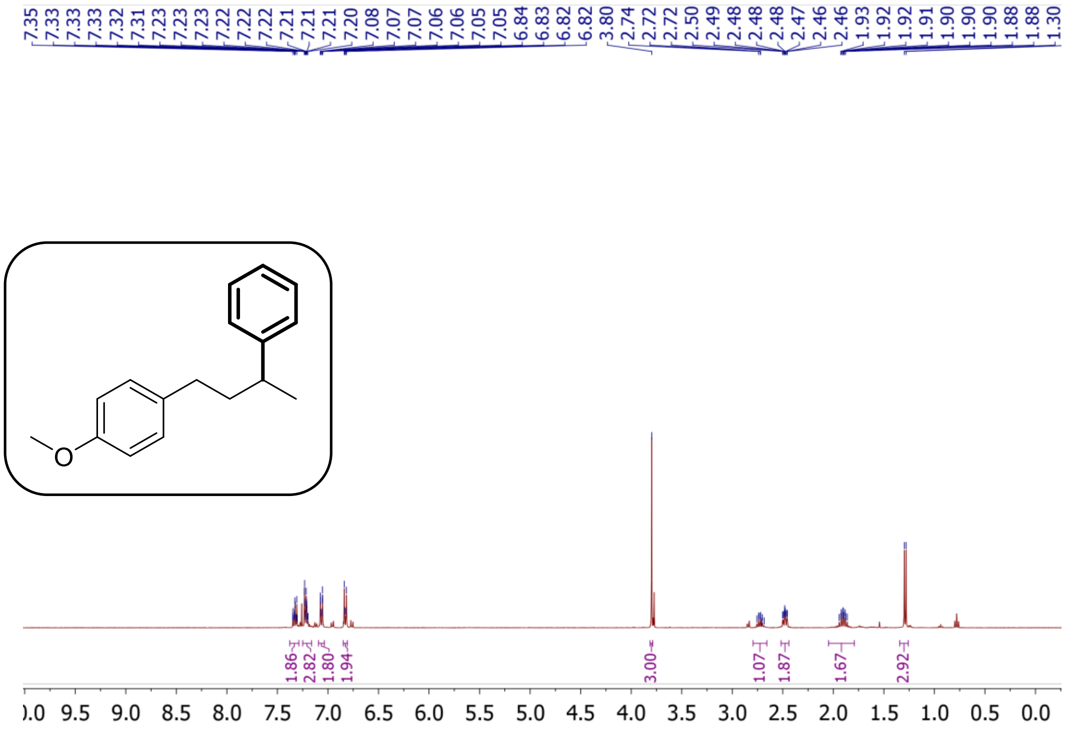

Supplementary Fig. 55. ^{13}C NMR (100 MHz, $CDCl_3$) spectrum of 1-methoxy-4-(3-phenylbutyl)benzene (18).

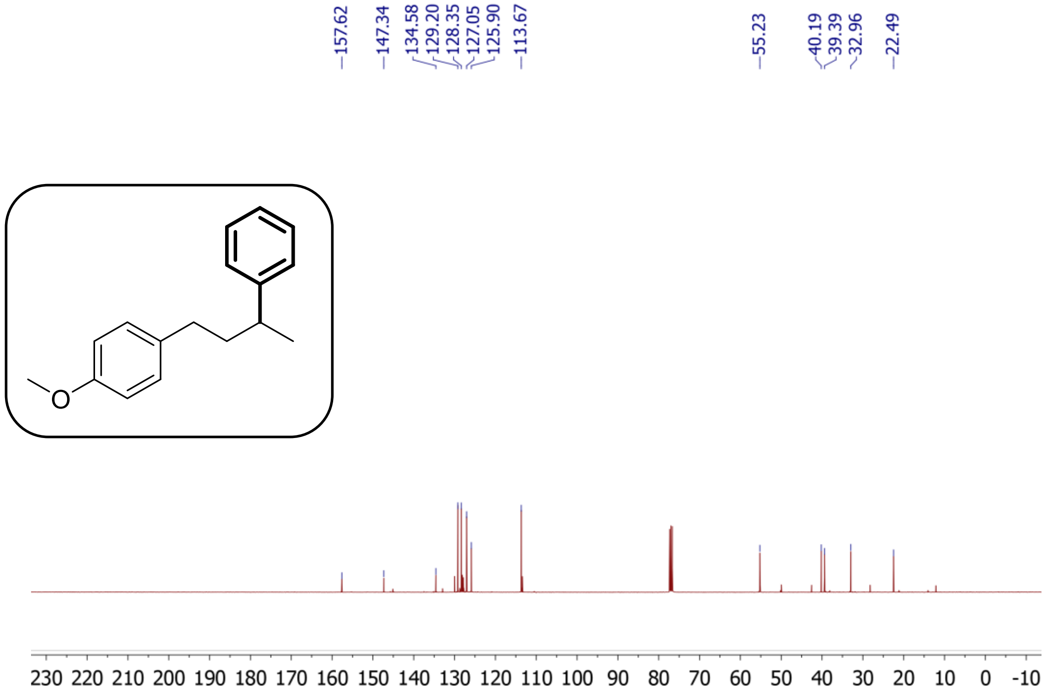

Supplementary Fig. 56. **^{1}H NMR (400 MHz, $CDCl_3$) spectrum of 1-nitro-3-(1-phenylethyl)benzene (19)**

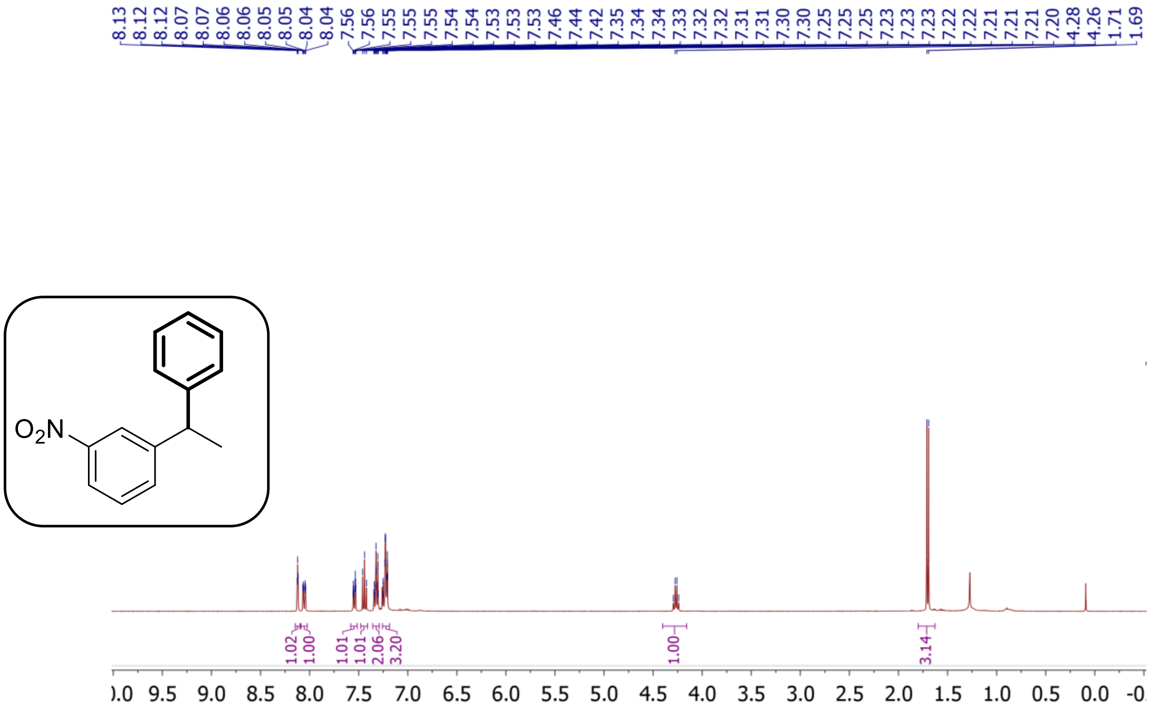

Supplementary Fig. 57. **^{13}C NMR (100 MHz, $CDCl_3$) spectrum of 1-nitro-3-(1-phenylethyl)benzene (19)**

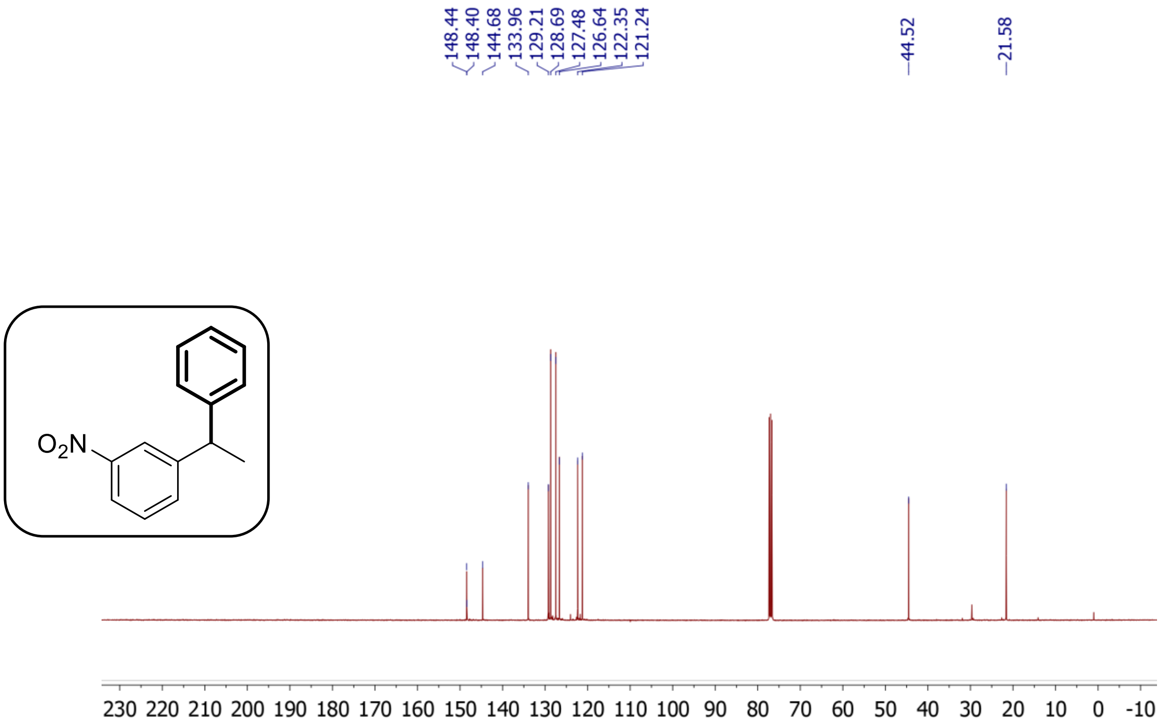

Supplementary Fig. 58. **^{1}H NMR (400 MHz, $CDCl_3$) spectrum of (cyclohexylmethylene)dibenzene (20).**

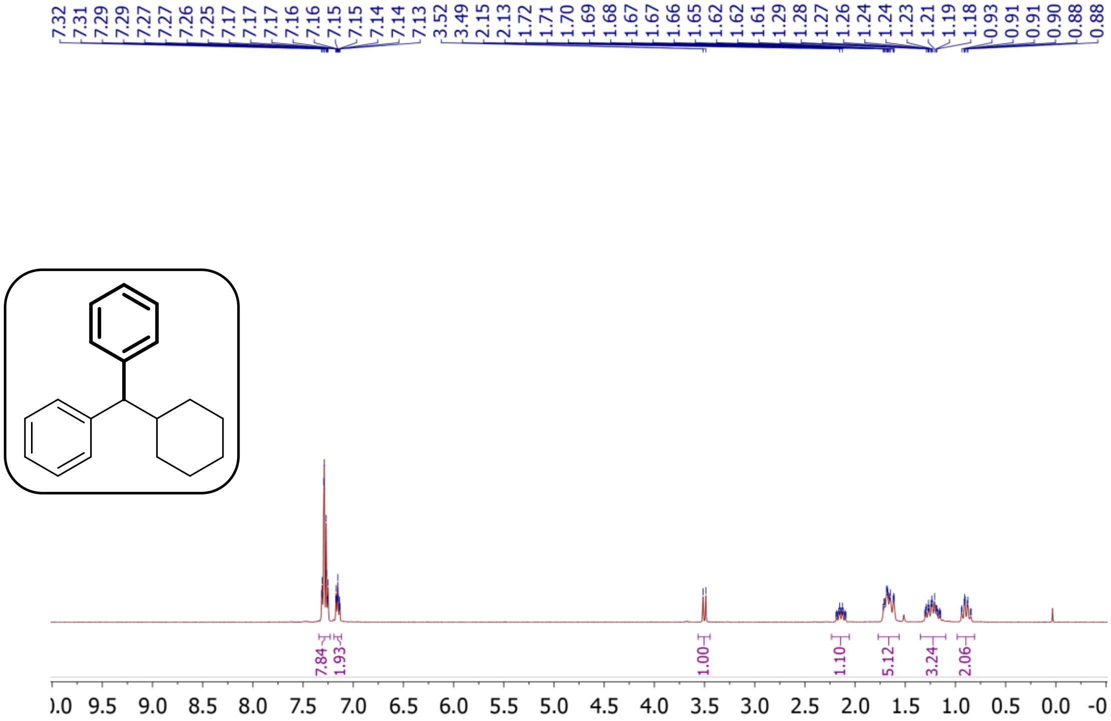

Supplementary Fig. 59. **^{13}C NMR (100 MHz, $CDCl_3$) spectrum of (cyclohexylmethylene)dibenzene (20).**

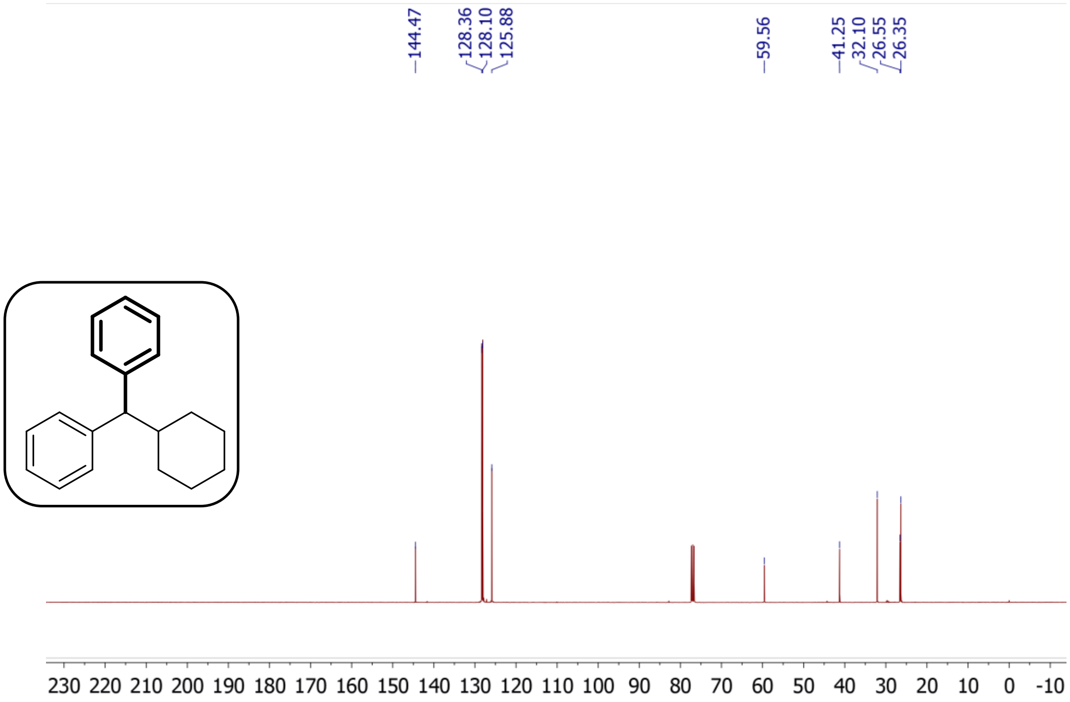

Supplementary Fig. 60. ^{1}H NMR (400 MHz, $CDCl_3$) spectrum of but-1-yne-1,3-diyldibenzene (21).

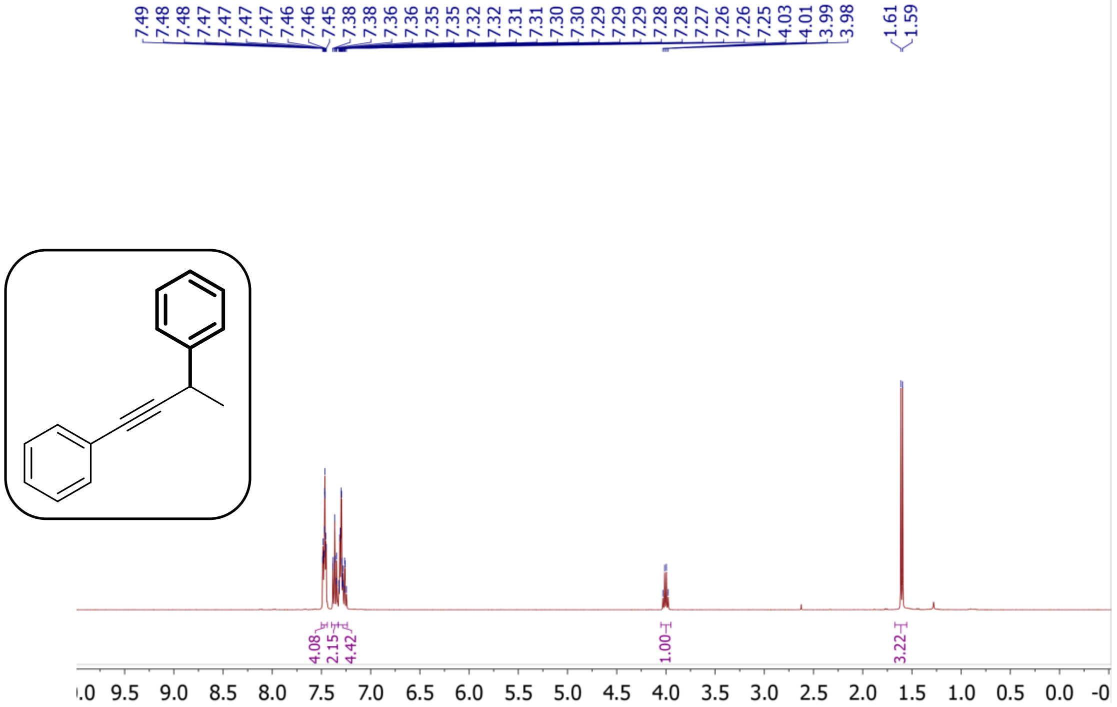

Supplementary Fig. 61. ^{13}C NMR (100 MHz, $CDCl_3$) spectrum of but-1-yne-1,3-diyldibenzene (21).

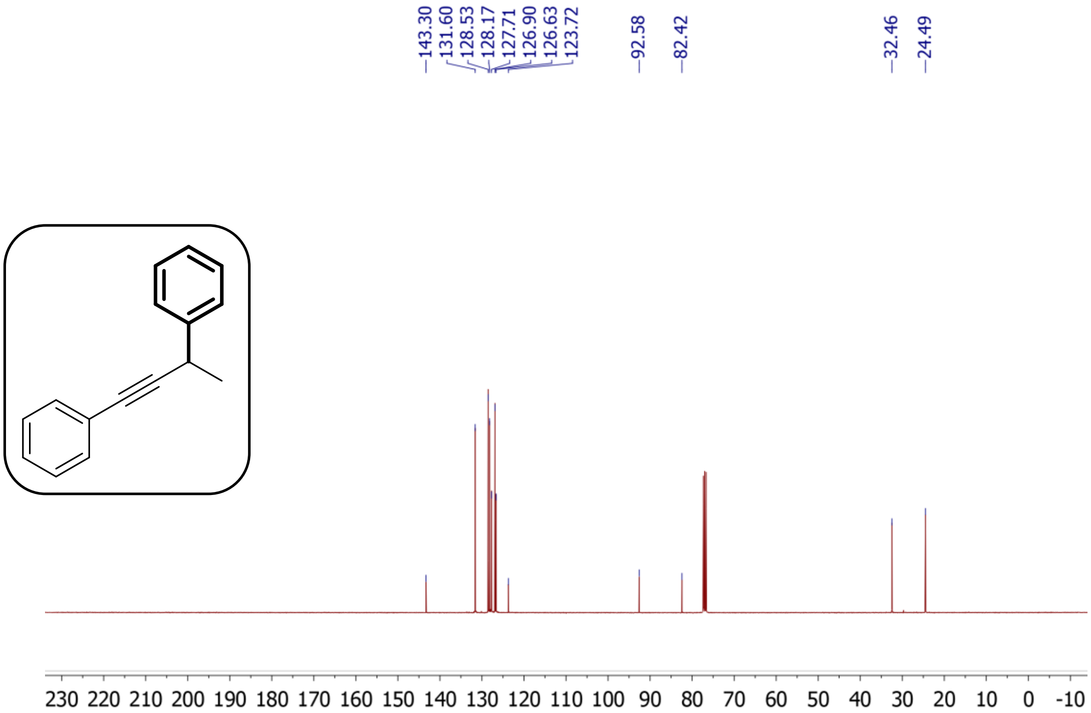

Supplementary Fig. 62. ^{1}H NMR (400 MHz, $CDCl_3$) spectrum of 4-(phenyl(*o*-tolyl)methyl)benzonitrile (22).

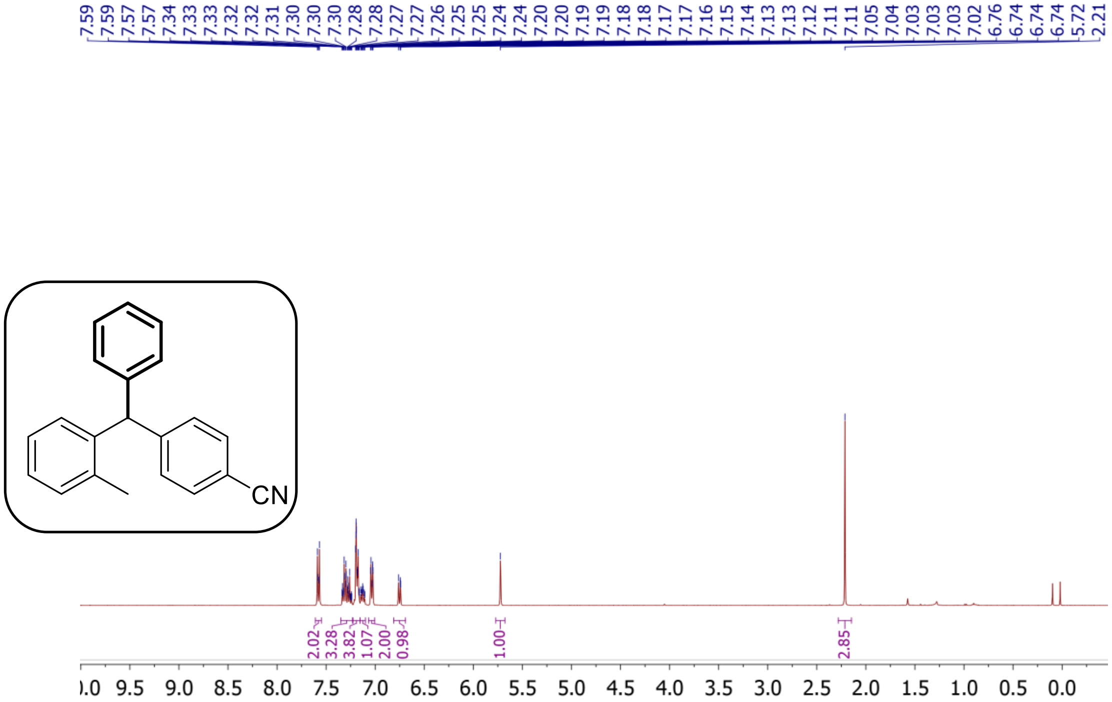

Supplementary Fig. 63. ^{13}C NMR (100 MHz, $CDCl_3$) spectrum of 4-(phenyl(*o*-tolyl)methyl)benzonitrile (22).

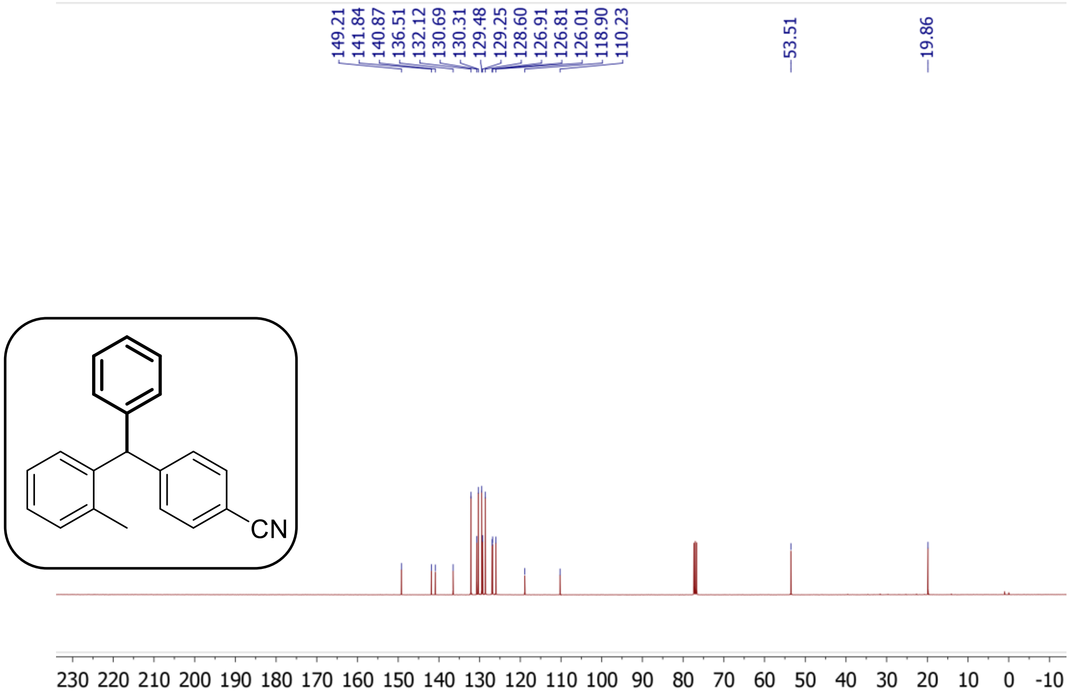

Supplementary Fig. 64. ^{1}H NMR (400 MHz, $CDCl_3$) spectrum of 1-phenyladamantane (23)

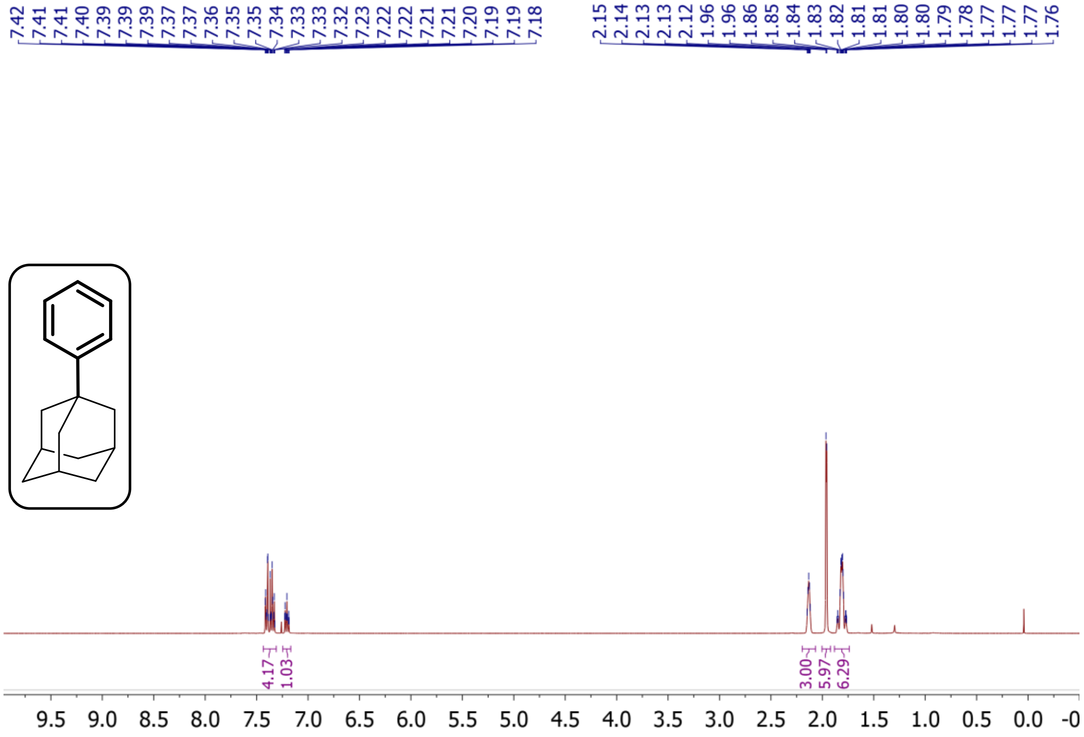

Supplementary Fig. 65. ^{13}C NMR (100 MHz, $CDCl_3$) spectrum of 1-phenyladamantane (23)

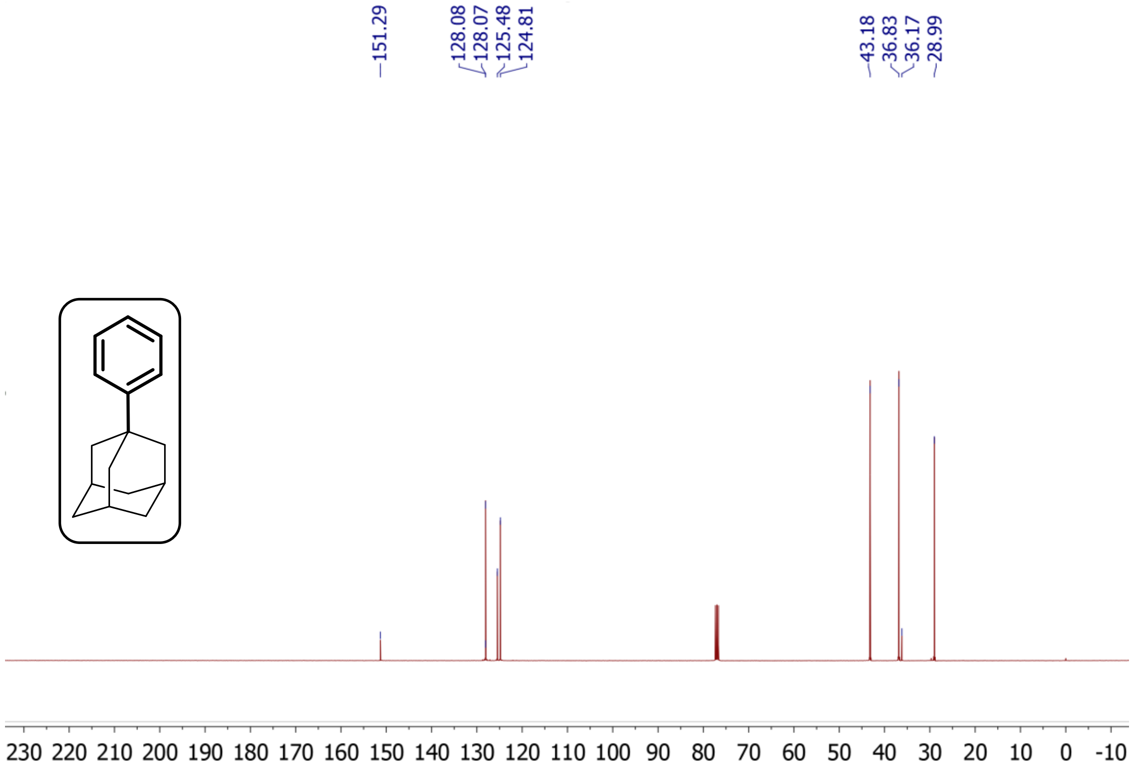

Supplementary Fig. 66. ^{1}H NMR (400 MHz, $CDCl_3$) spectrum of 2-methyl-1,2-diphenylpropane-1-one (24).

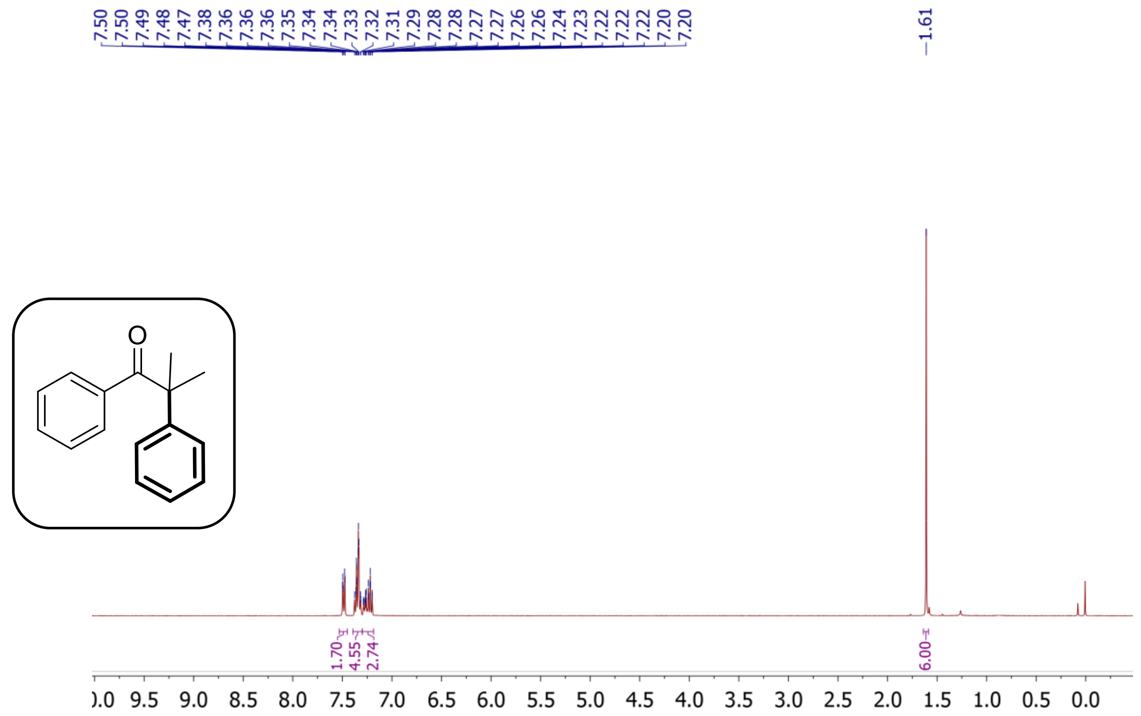

Supplementary Fig. 67. ^{13}C NMR (100 MHz, $CDCl_3$) spectrum of 2-methyl-1,2-diphenylpropane-1-one (24).

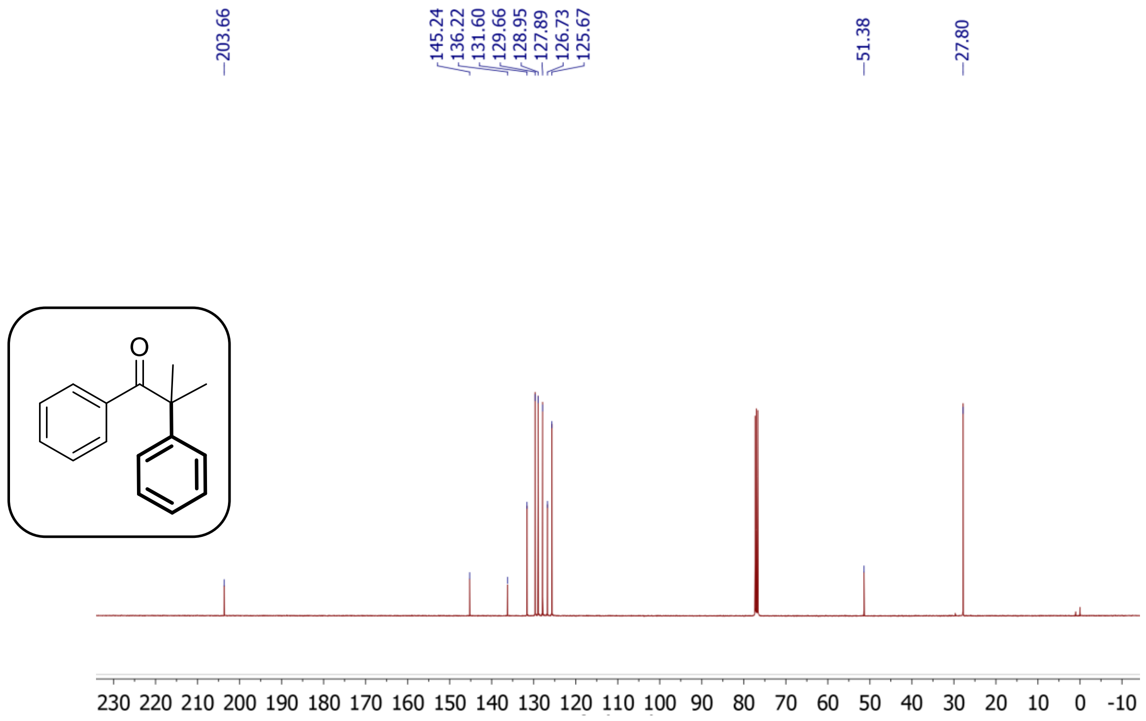

Supplementary Fig. 68. ^{1}H **NMR (400 MHz, CDCl$_3$) spectrum of 2-(1,1-diphenylethyl)pyridine (25)**

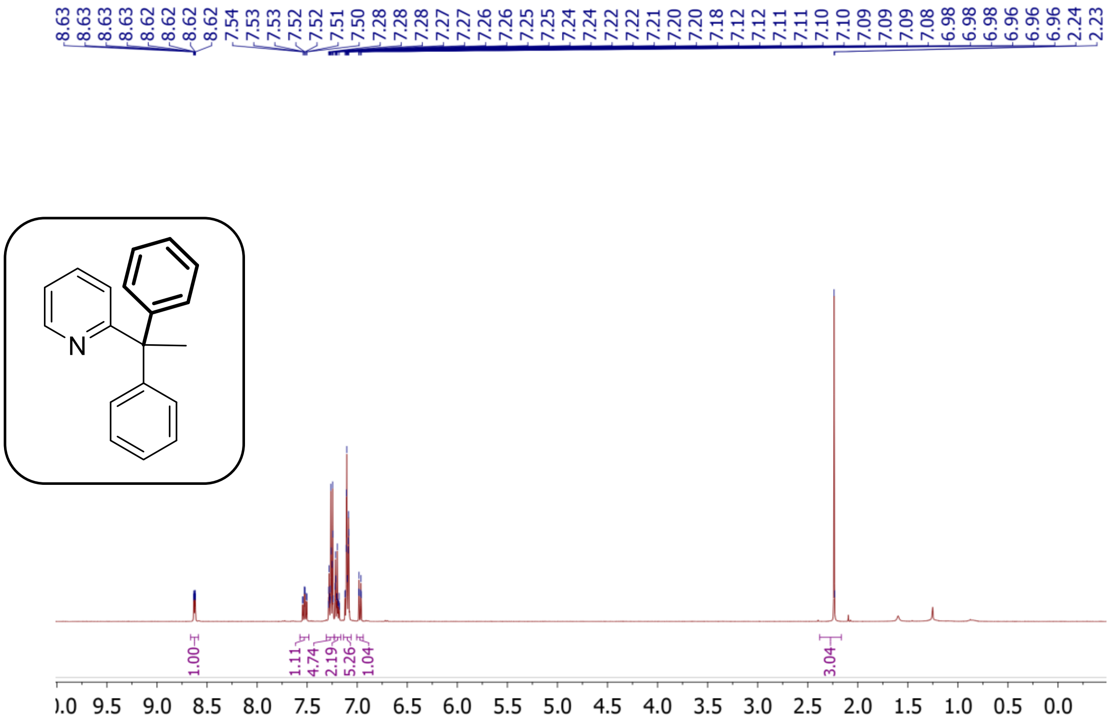

Supplementary Fig. 69. ^{13}C **NMR (100 MHz, CDCl$_3$) spectrum of 2-(1,1-diphenylethyl)pyridine (25)**

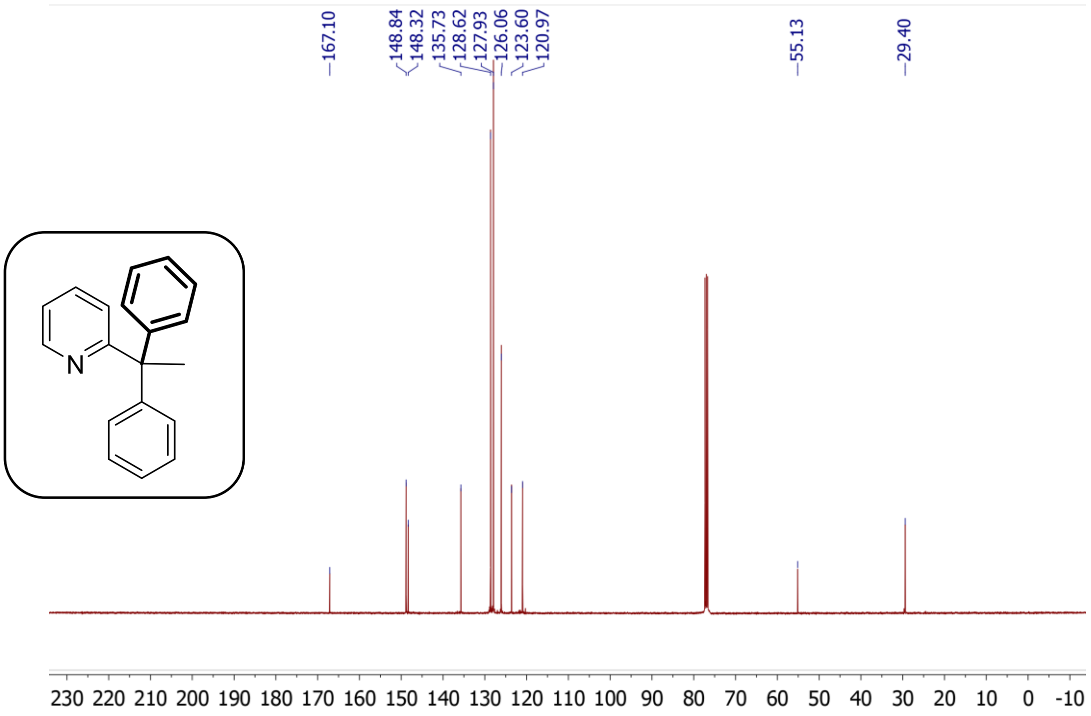

Supplementary Fig. 70. ^{1}**H NMR (400 MHz, CDCl$_3$) spectrum of 4,4',4''-(phenylmethanetriyl)tris(methylbenzene) (26).**

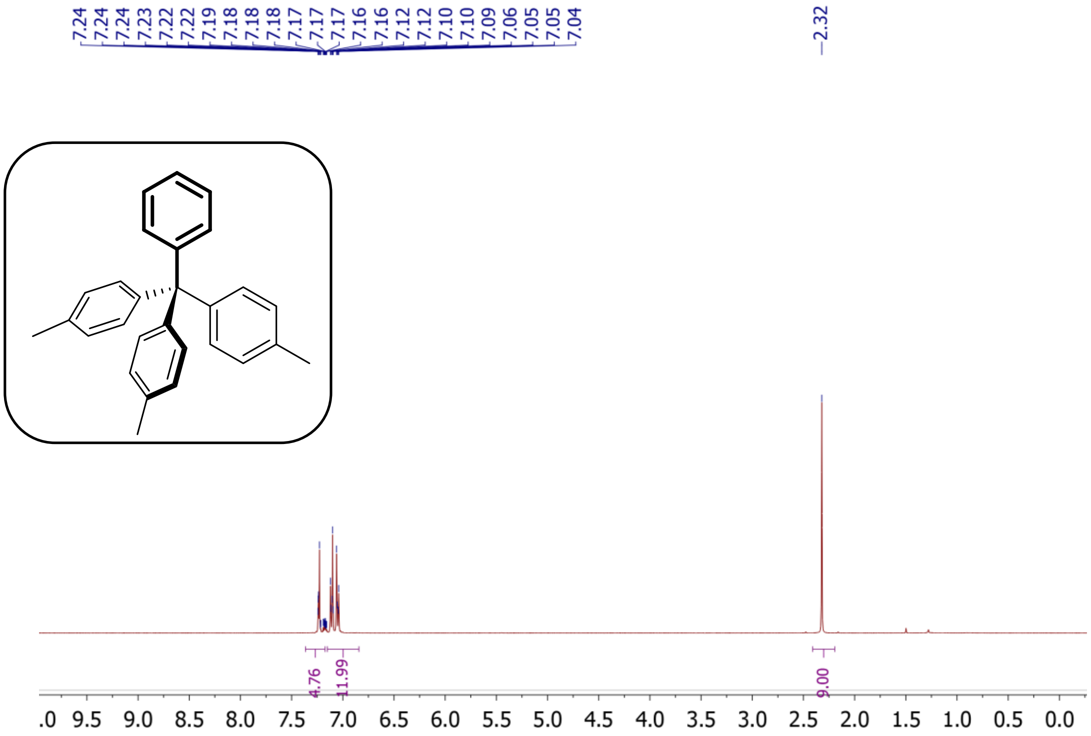

Supplementary Fig. 71. ^{13}**C NMR (100 MHz, CDCl$_3$) spectrum of 4,4',4''-(phenylmethanetriyl)tris(methylbenzene) (26).**

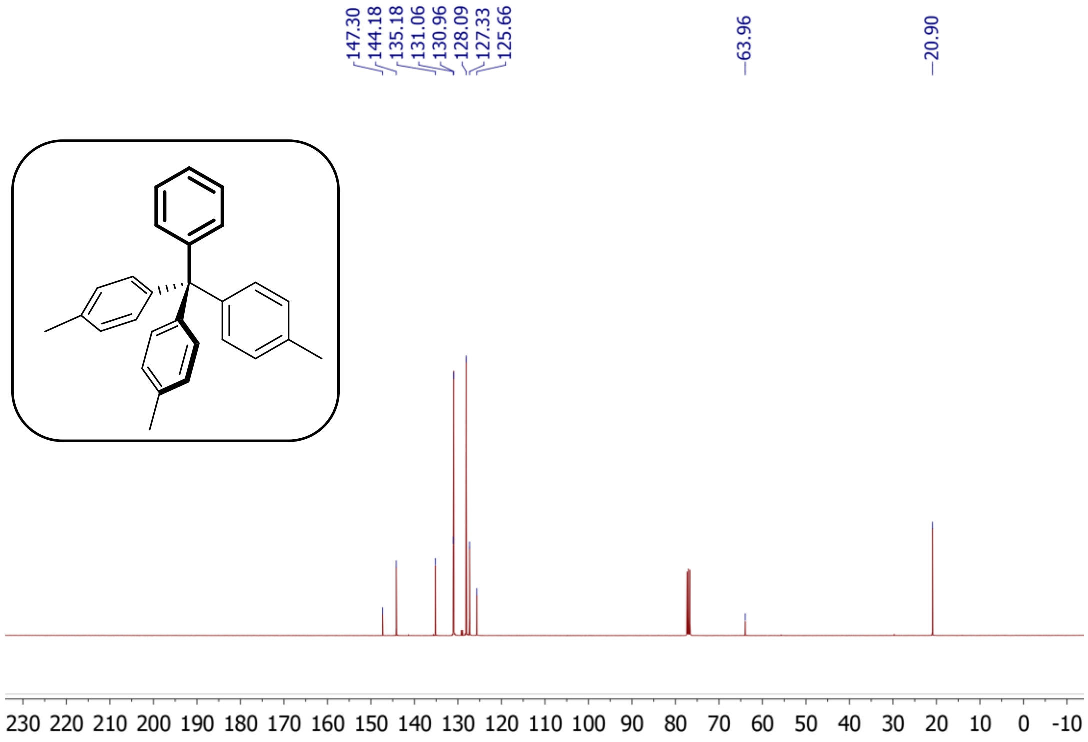

Supplementary Fig. 72. ^{1}H NMR (400 MHz, CDCl3) spectrum of 3-(pent-4-en-1-yl)-1,3-diphenylindolin-2-one (27).

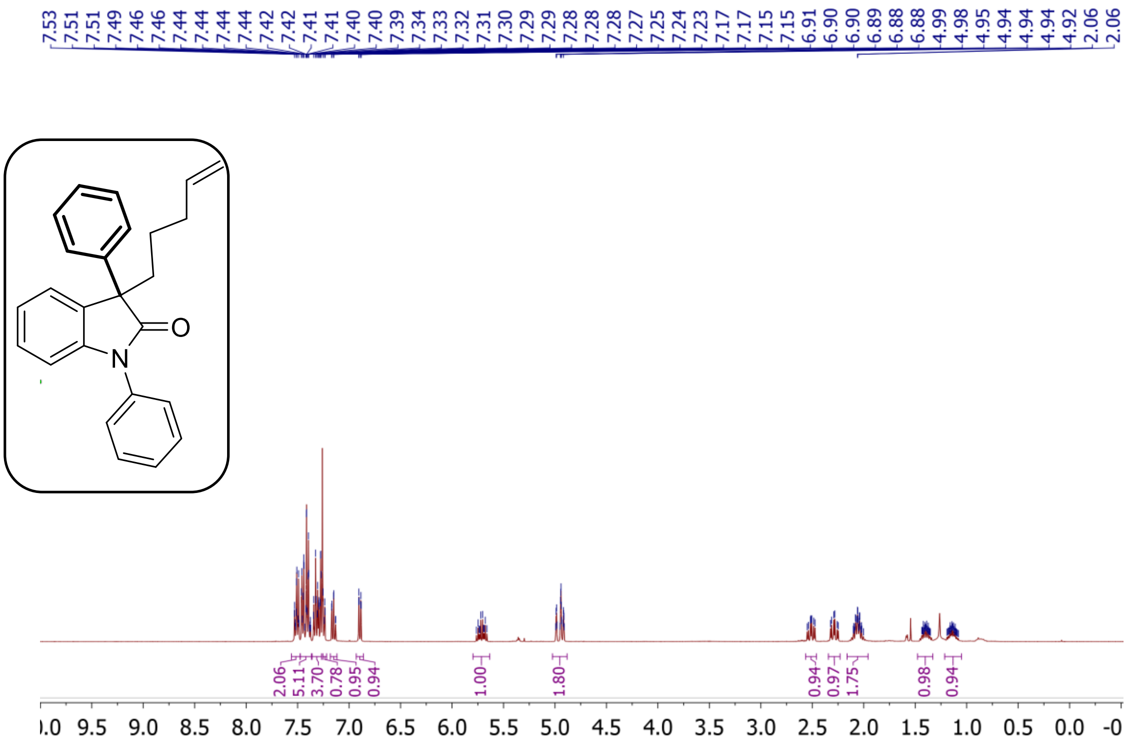

Supplementary Fig. 73. ^{13}C NMR (100 MHz, CDCl3) spectrum of 3-(pent-4-en-1-yl)-1,3-diphenylindolin-2-one (27).

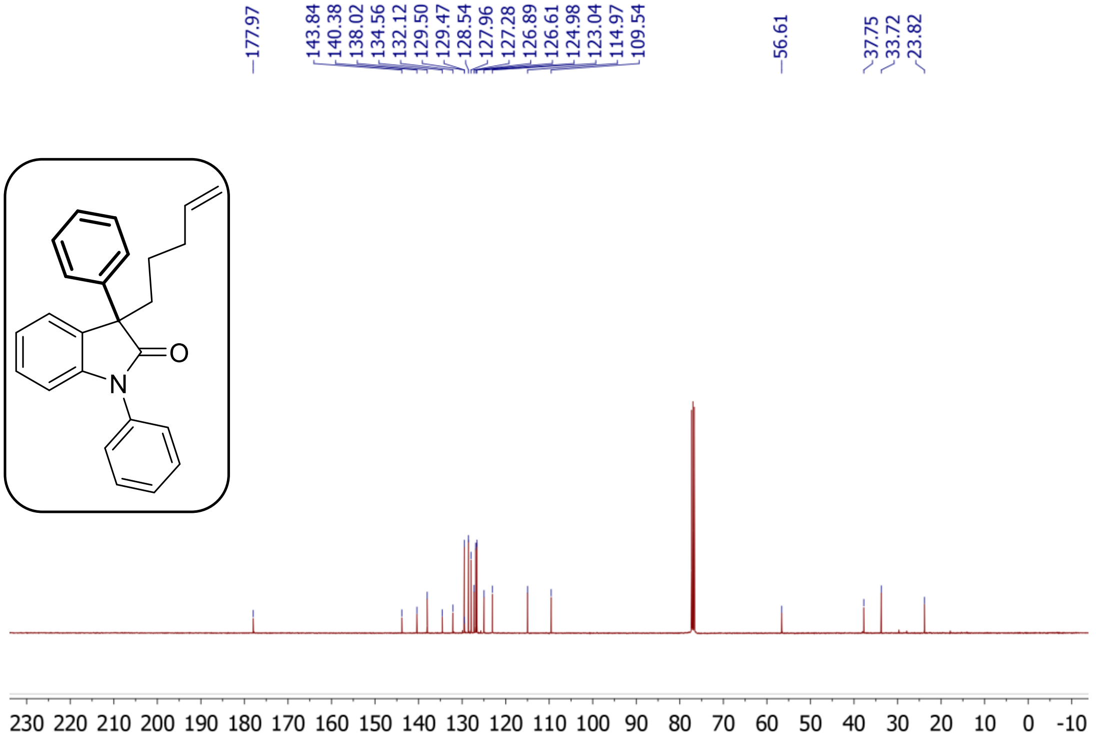

Supplementary Fig. 74. ^{1}H NMR (400 MHz, $CDCl_3$) spectrum of (3-chloro-2-fluorophenyl)(phenyl)methanone (28)

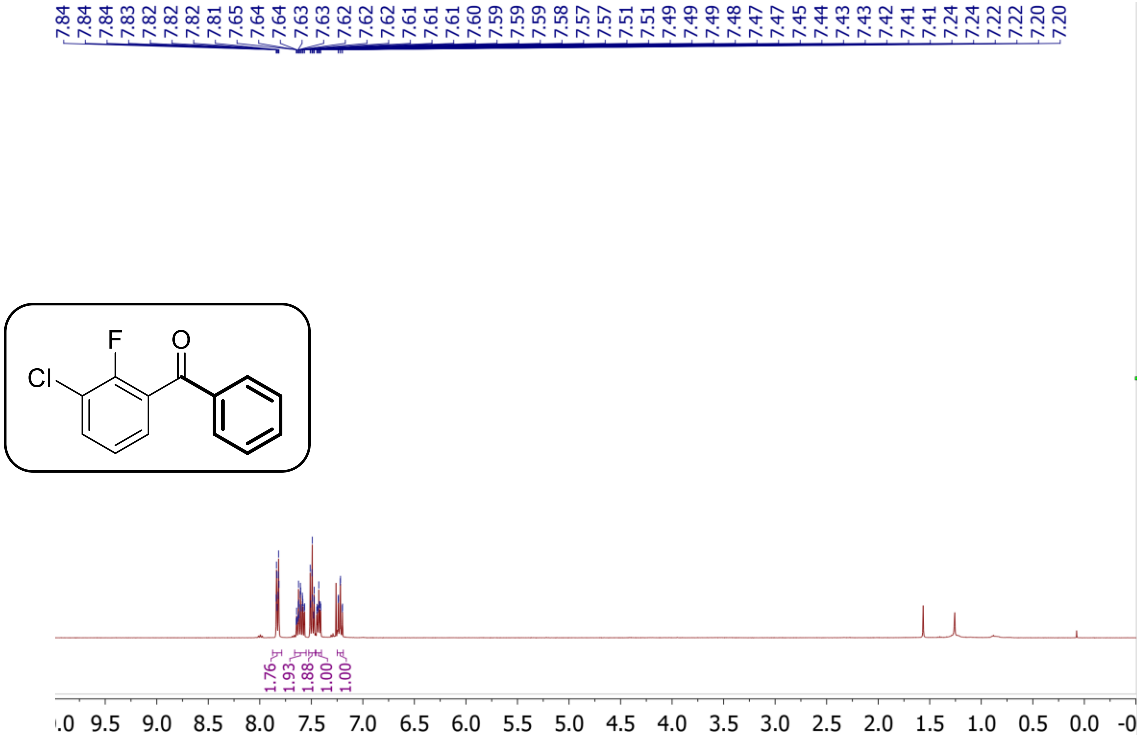

Supplementary Fig. 75. ^{13}C NMR (100 MHz, $CDCl_3$) spectrum of (3-chloro-2-fluorophenyl)(phenyl)methanone (28)

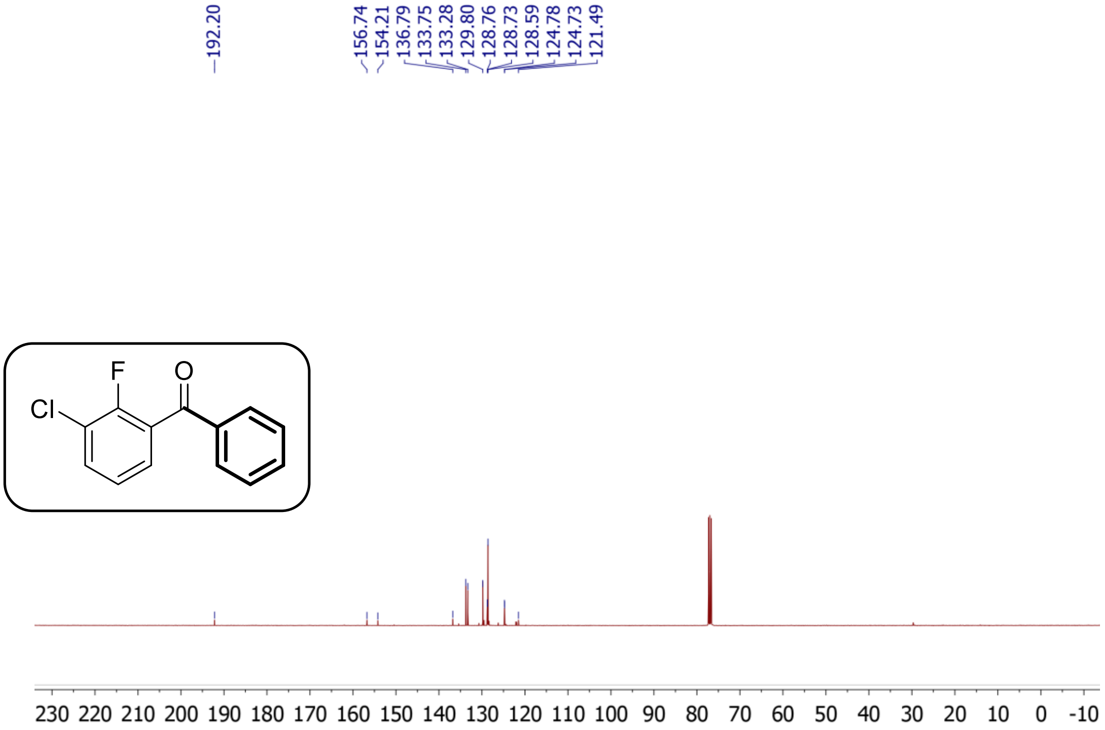

Supplementary Fig. 76. ^{19}F NMR (376 MHz, $CDCl_3$) spectrum of (3-chloro-2-fluorophenyl)(phenyl)methanone (28)

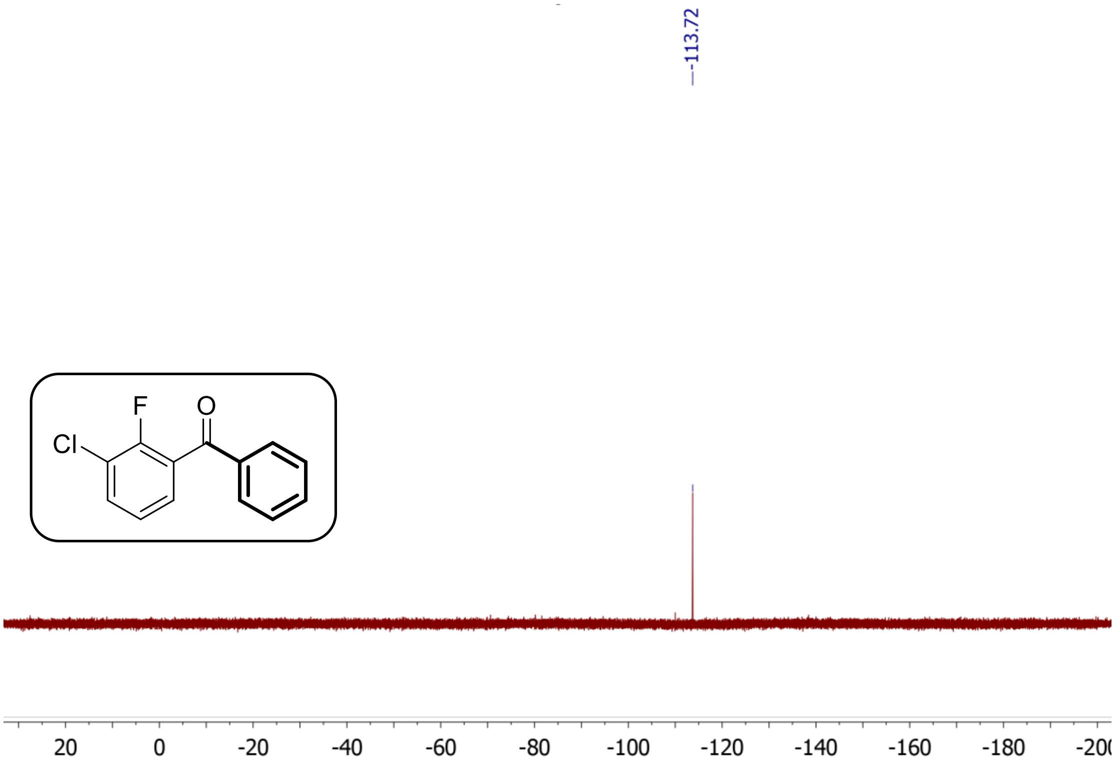

Supplementary Fig. 77. ^{1}H NMR (400 MHz, $CDCl_3$) spectrum of phenyl(4-(trifluoromethyl)phenyl)methanone (29)

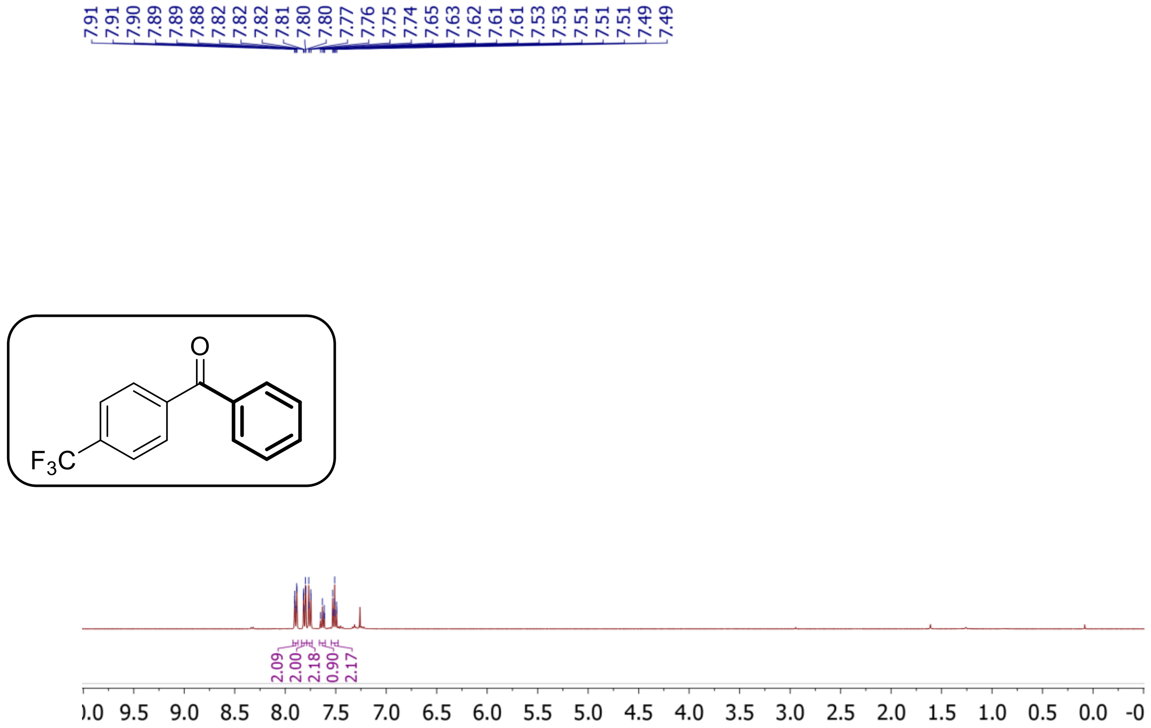

Supplementary Fig. 78. ^{13}C NMR (100 MHz, $CDCl_3$) spectrum of phenyl(4-(trifluoromethyl)phenyl)methanone (29)

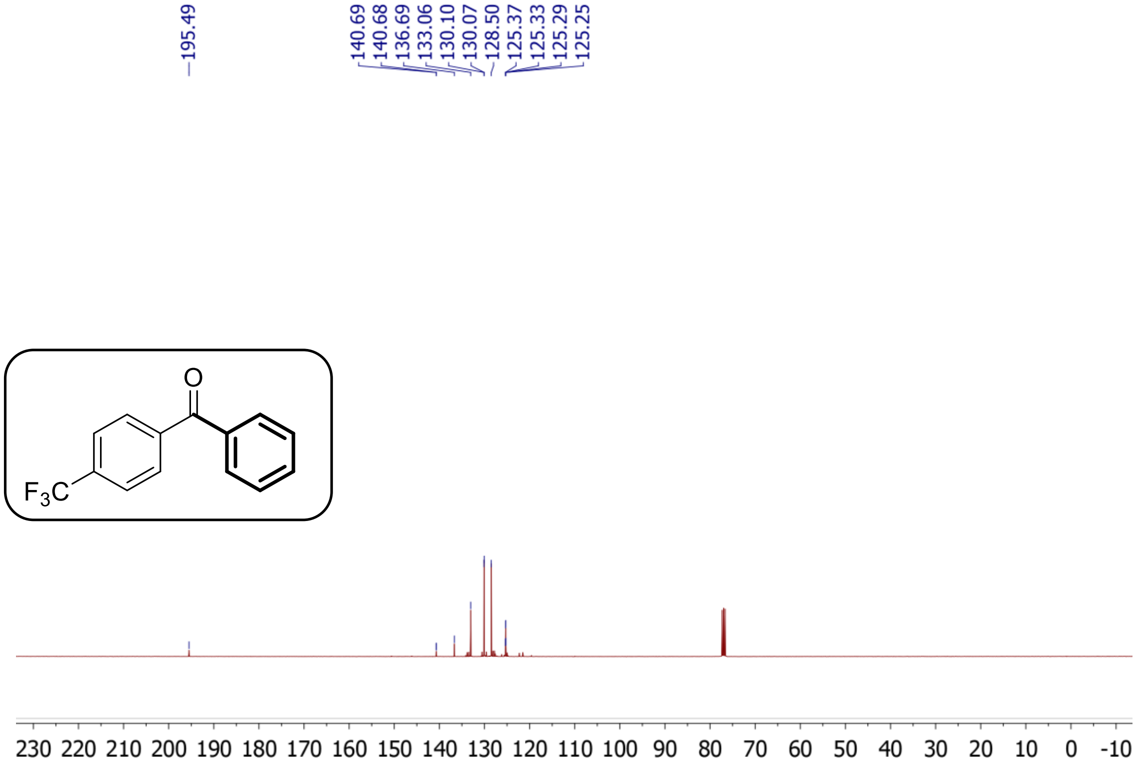

Supplementary Fig. 79. ^{19}F NMR (376 MHz, $CDCl_3$) spectrum of phenyl(4-(trifluoromethyl)phenyl)methanone (29)

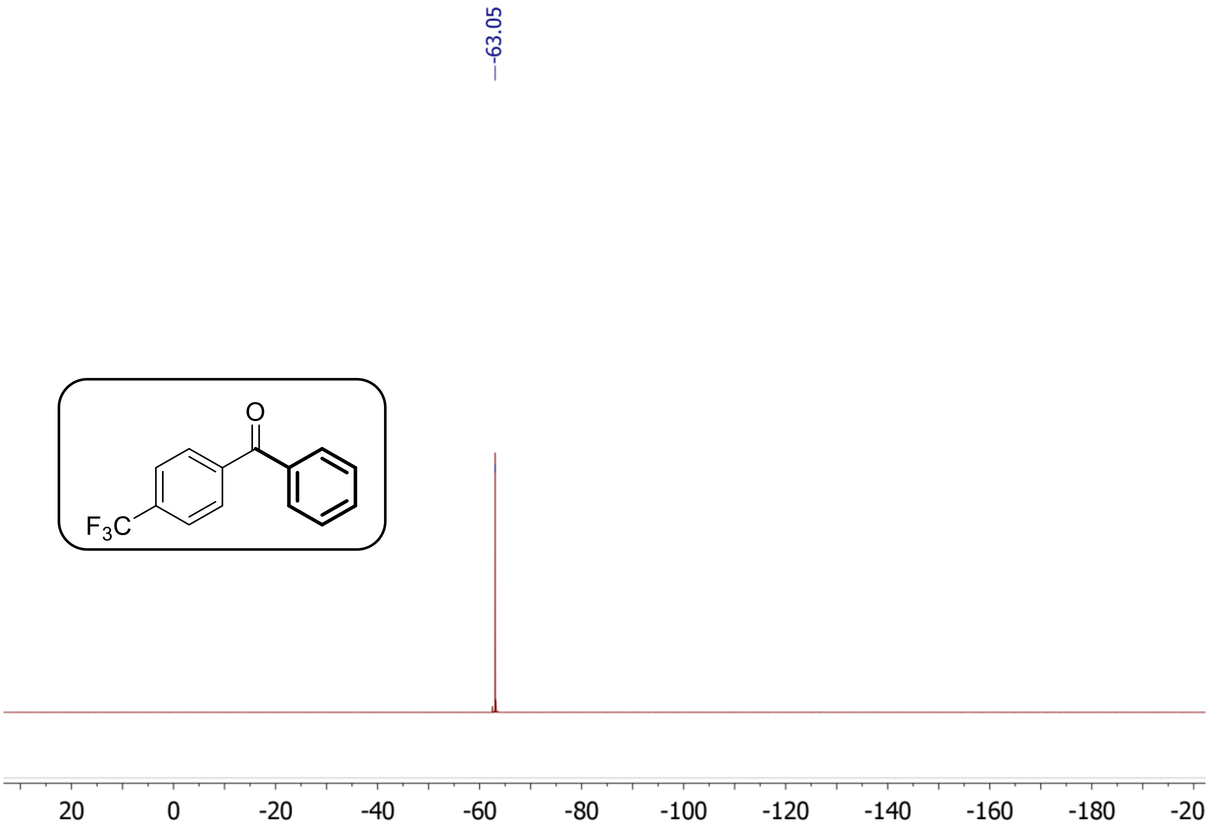

Supplementary Fig. 80. ^{1}H NMR (400 MHz, $CDCl_3$) spectrum of benzophenone (30).

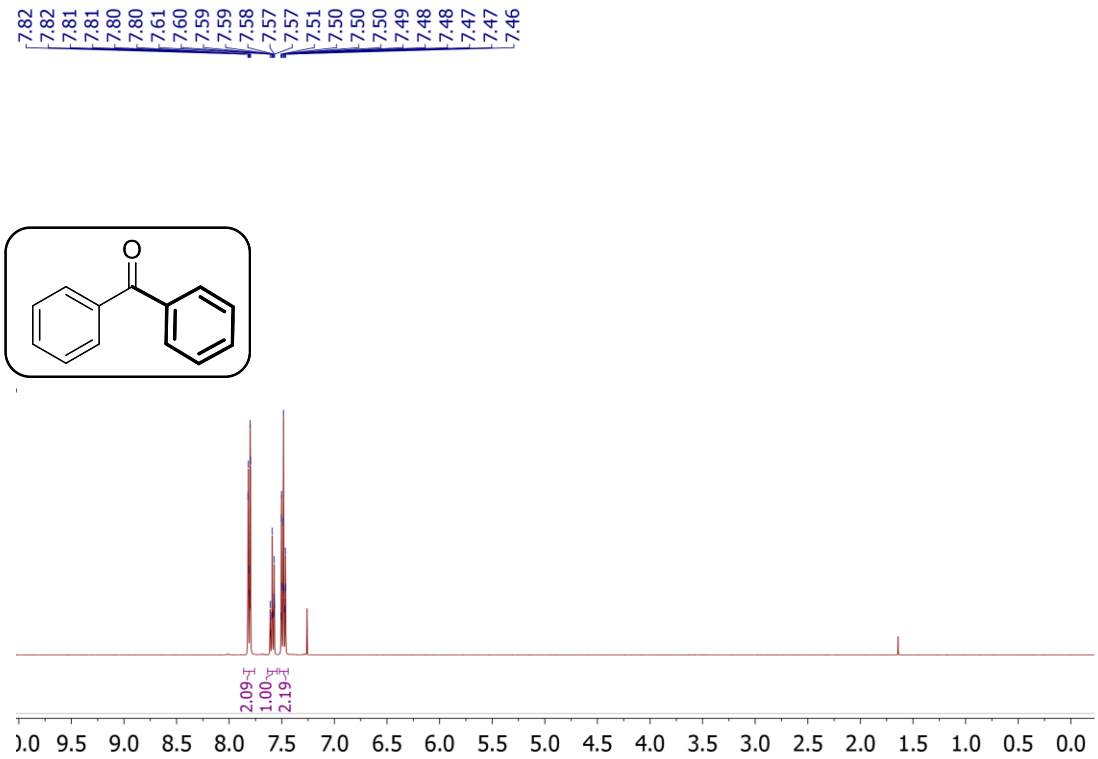

Supplementary Fig. 81. ^{13}C NMR (100 MHz, $CDCl_3$) spectrum of benzophenone (30).

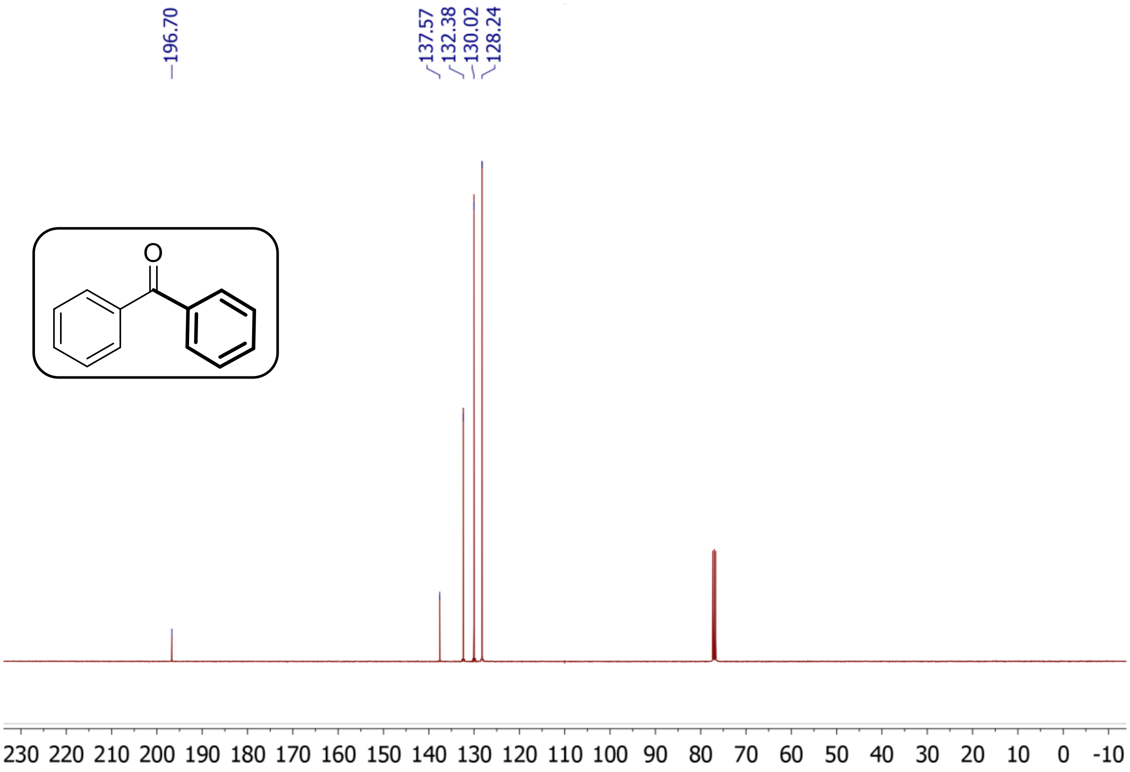

Supplementary Fig. 82. ^{1}H NMR (400 MHz, $CDCl_3$) spectrum of 1-(2,4-dimethoxyphenyl)adamantane (39).

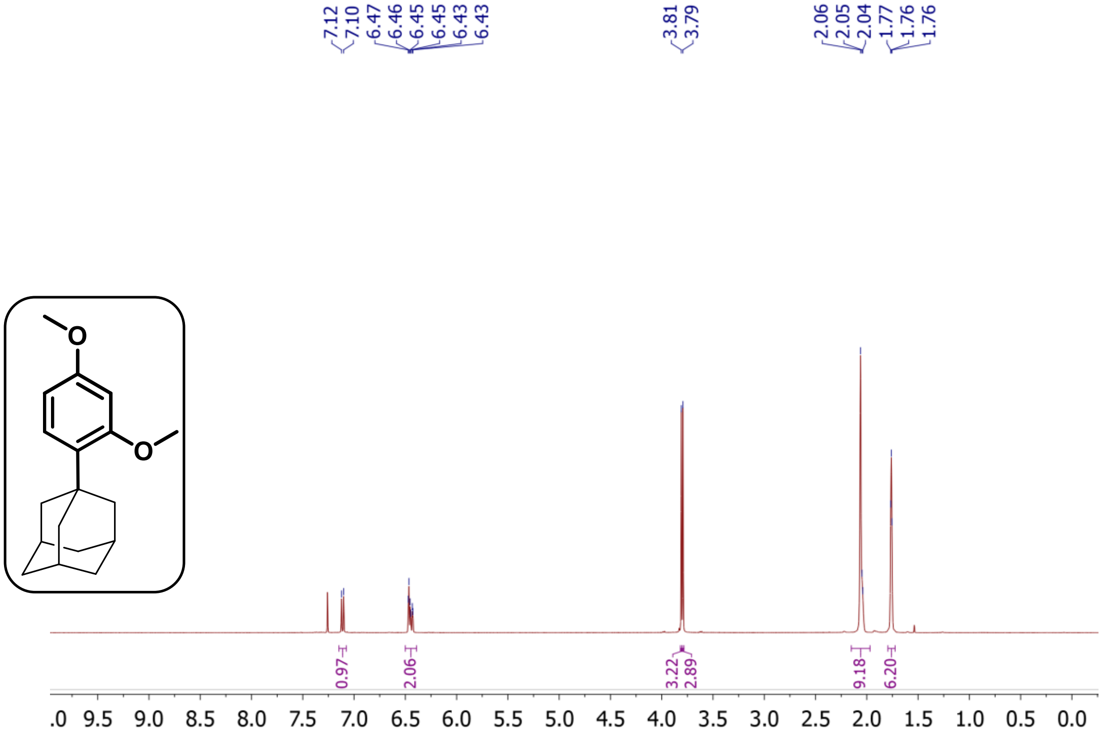

Supplementary Fig. 83. ^{13}C NMR (100 MHz, $CDCl_3$) spectrum of 1-(2,4-dimethoxyphenyl)adamantane (39).

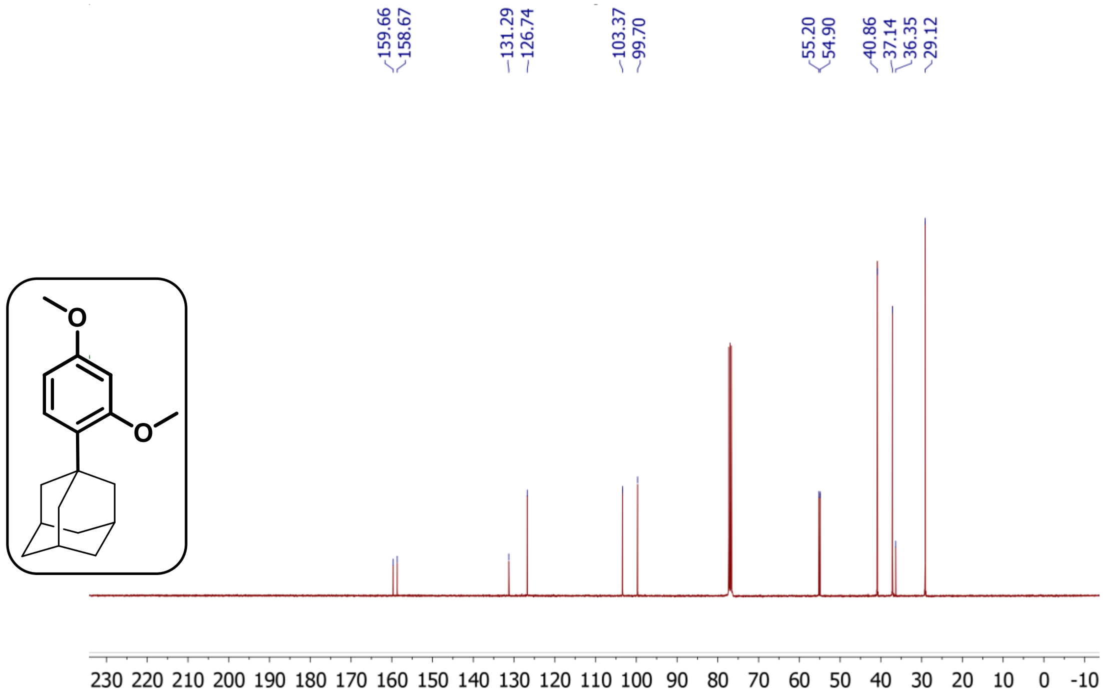

Supplementary Fig. 84. **¹H NMR (400 MHz, CDCl₃) spectrum of 2-dodecyl-3-methylbenzo[b]thiophene (51).**

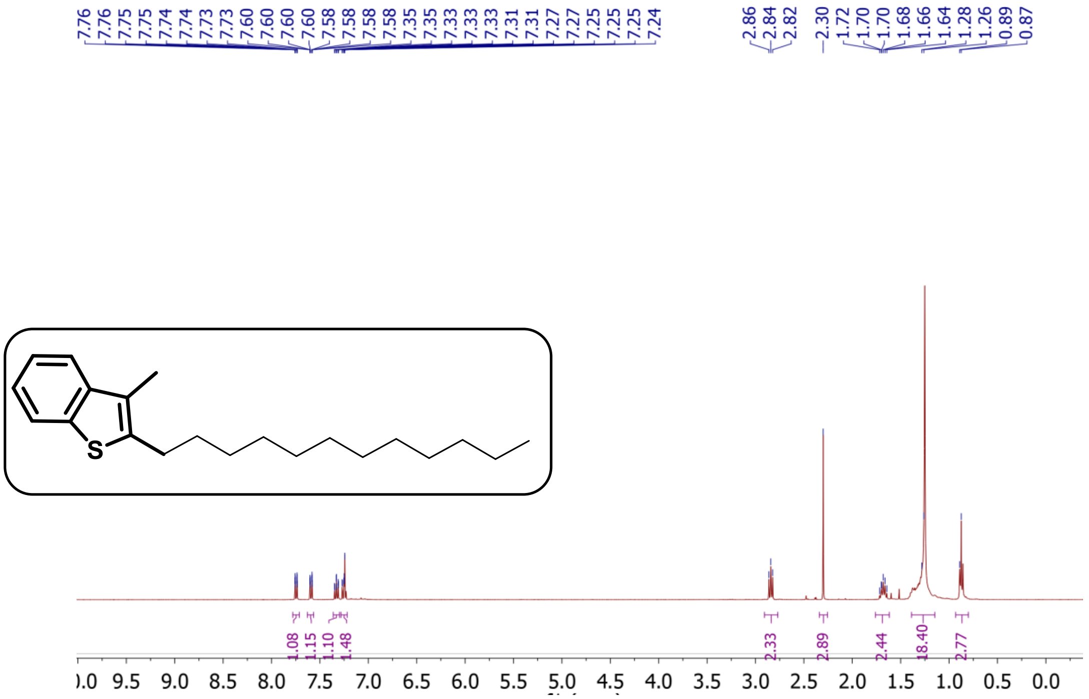

Supplementary Fig. 85. **¹³C NMR (100 MHz, CDCl₃) spectrum of 2-dodecyl-3-methylbenzo[b]thiophene (51).**

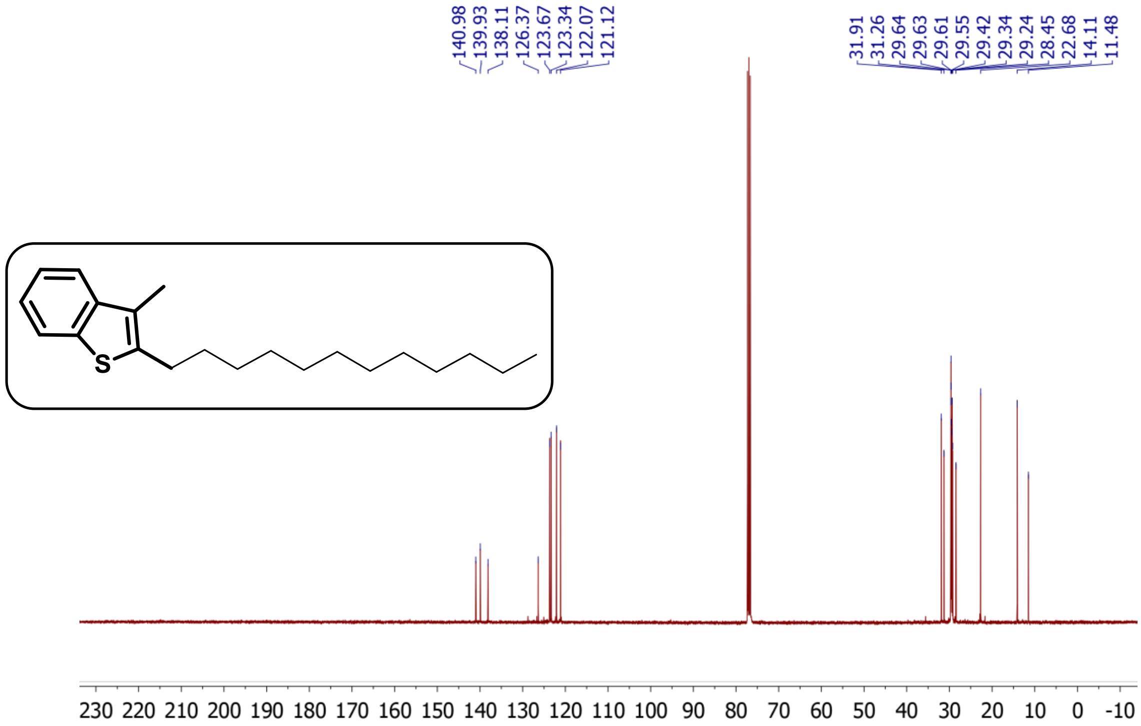

Supplementary Fig. 86. ^{1}**H NMR (400 MHz, CDCl$_3$) spectrum of 2-(3-(3-methylbenzo[b]thiophen-2-yl)propyl)isoindoline-1,3-dione (52).**

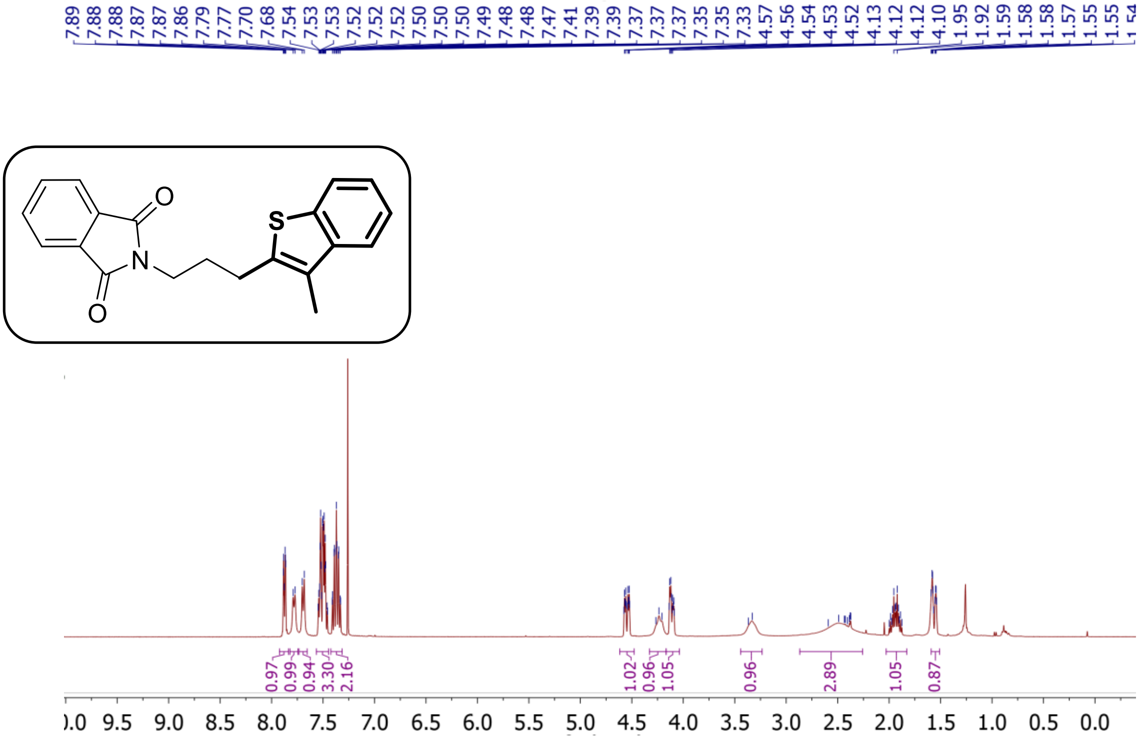

Supplementary Fig. 87. ^{13}**C NMR (100 MHz, CDCl$_3$) spectrum of 2-(3-(3-methylbenzo[b]thiophen-2-yl)propyl)isoindoline-1,3-dione (52).**

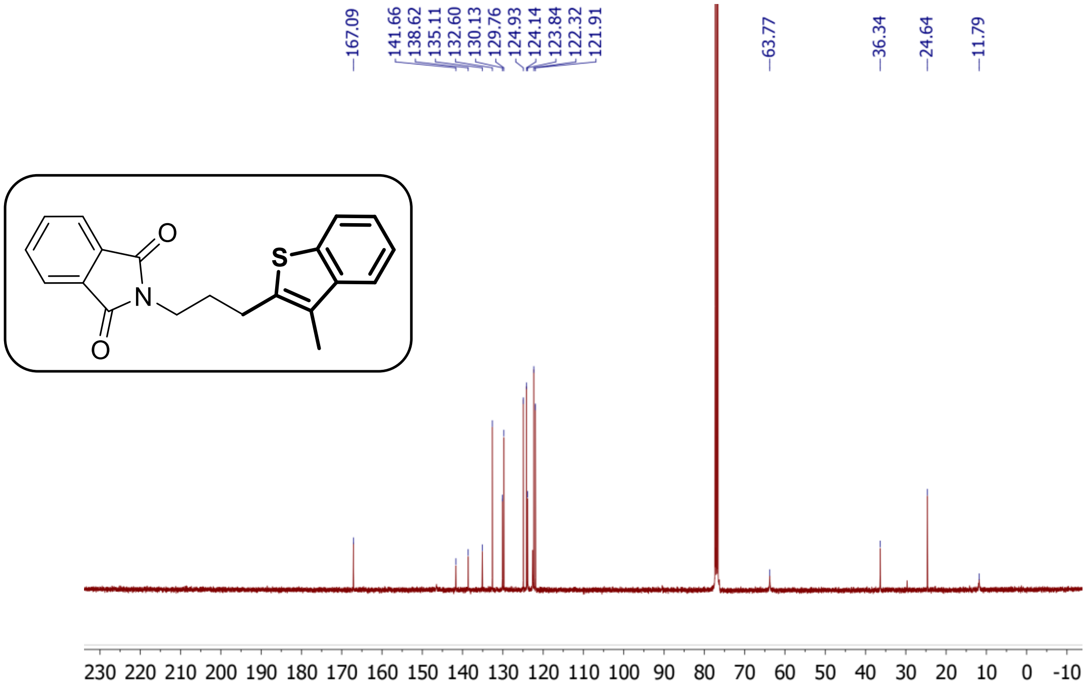

Supplementary Fig. 88. ^{1}H NMR (400 MHz, $CDCl_3$) spectrum of (4-bromo-2-methylbenzyl)ferrocene (53).

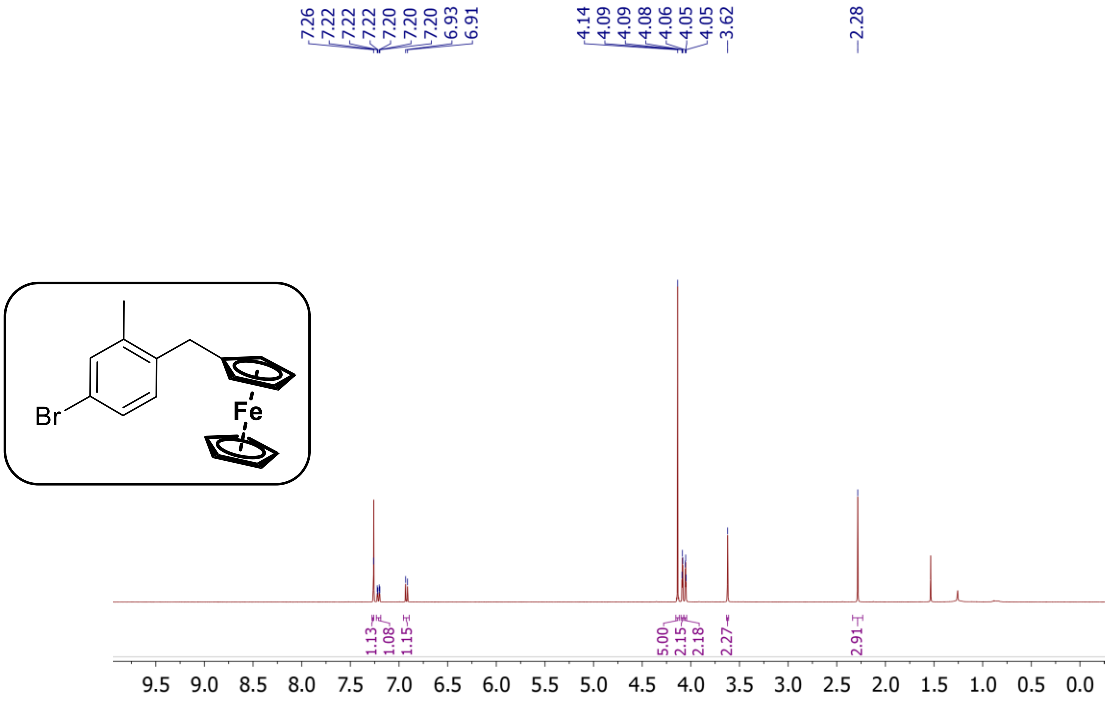

Supplementary Fig. 89. ^{13}C NMR (100 MHz, $CDCl_3$) spectrum of (4-bromo-2-methylbenzyl)ferrocene (53).

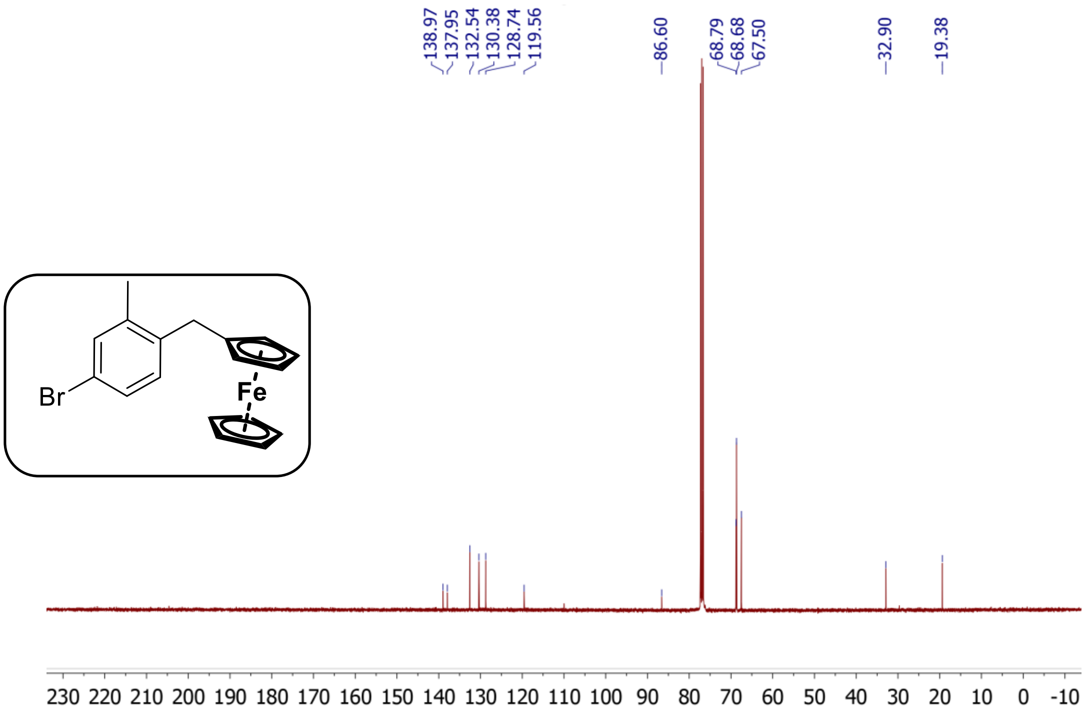

Supplementary Fig. 90. ^{1}H NMR (400 MHz, CDCl$_3$) spectrum of 5-(3-(3,4-dimethylthiophen-2-yl)butyl)benzo[d][1,3]dioxole (54).

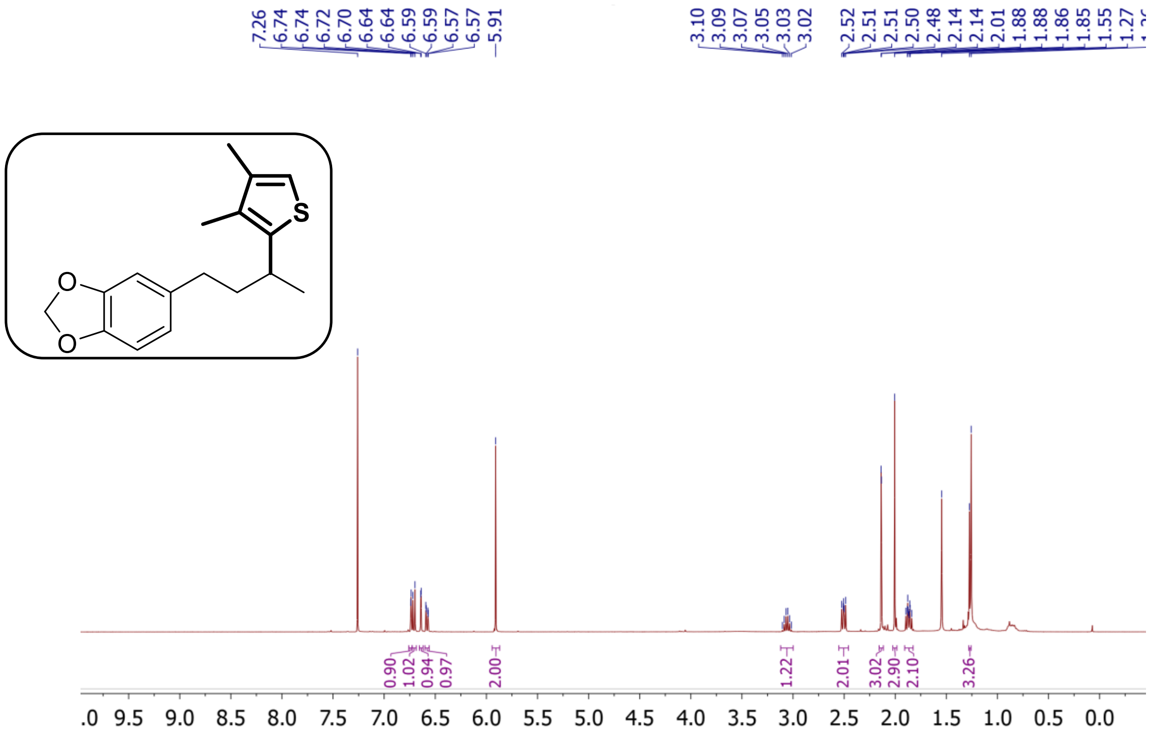

Supplementary Fig. 91. ^{13}C NMR (100 MHz, CDCl$_3$) spectrum of 5-(3-(3,4-dimethylthiophen-2-yl)butyl)benzo[d][1,3]dioxole (54).

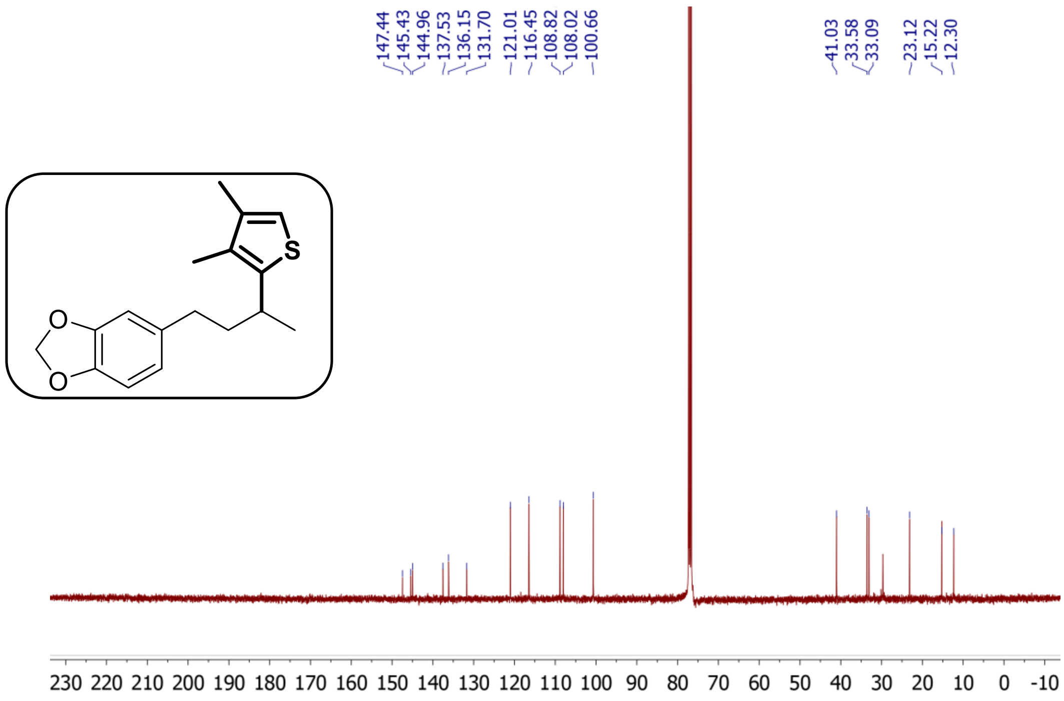

Supplementary Fig. 92. **¹H NMR (400 MHz, $CDCl_3$) spectrum of 4-(perfluorophenyl)-1,3-dioxolan-2-one (55).**

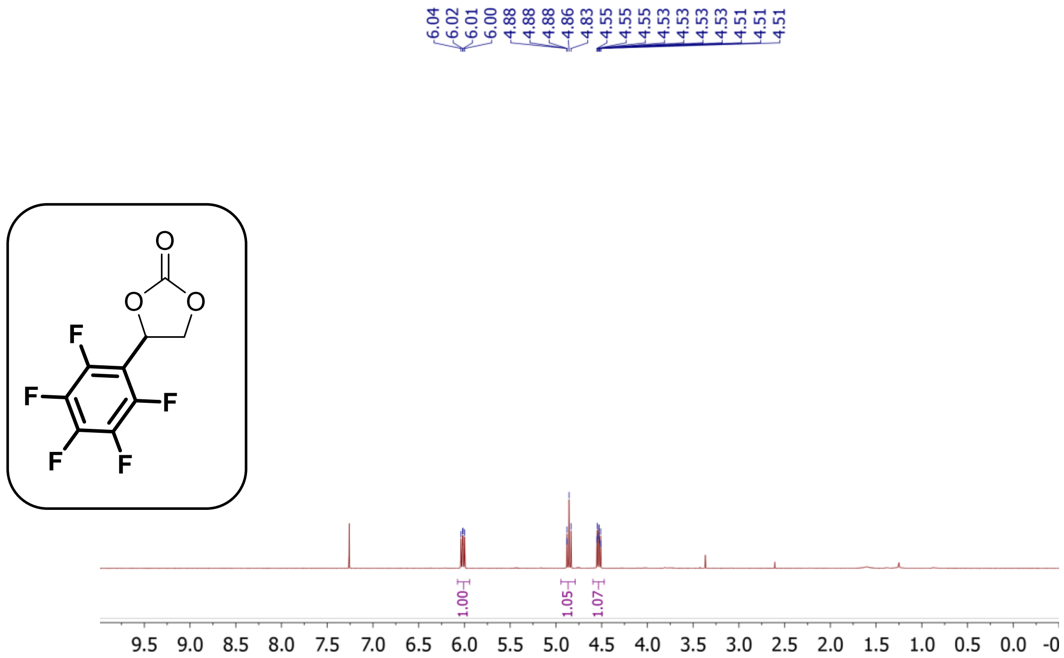

Supplementary Fig. 93. **¹³C NMR (100 MHz, $CDCl_3$) spectrum of 4-(perfluorophenyl)-1,3-dioxolan-2-one (55).**

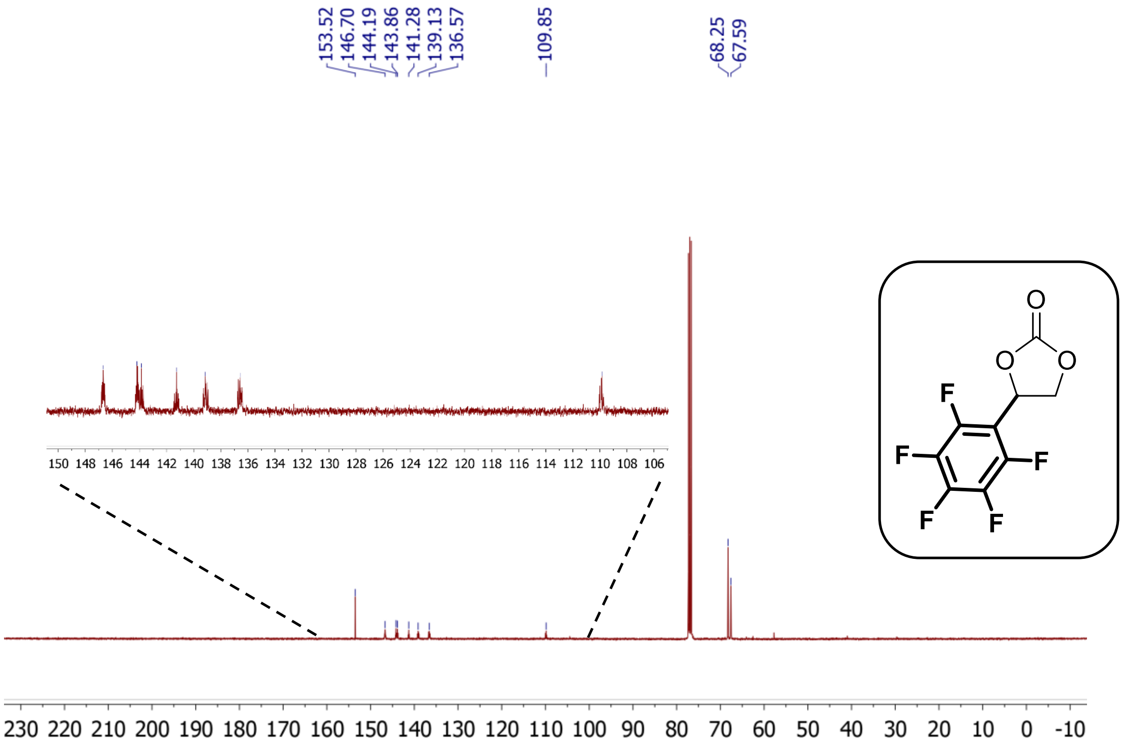

Supplementary Fig. 94. ^{19}F NMR (376 MHz, $CDCl_3$) spectrum of 4-(perfluorophenyl)-1,3-dioxolan-2-one (55).

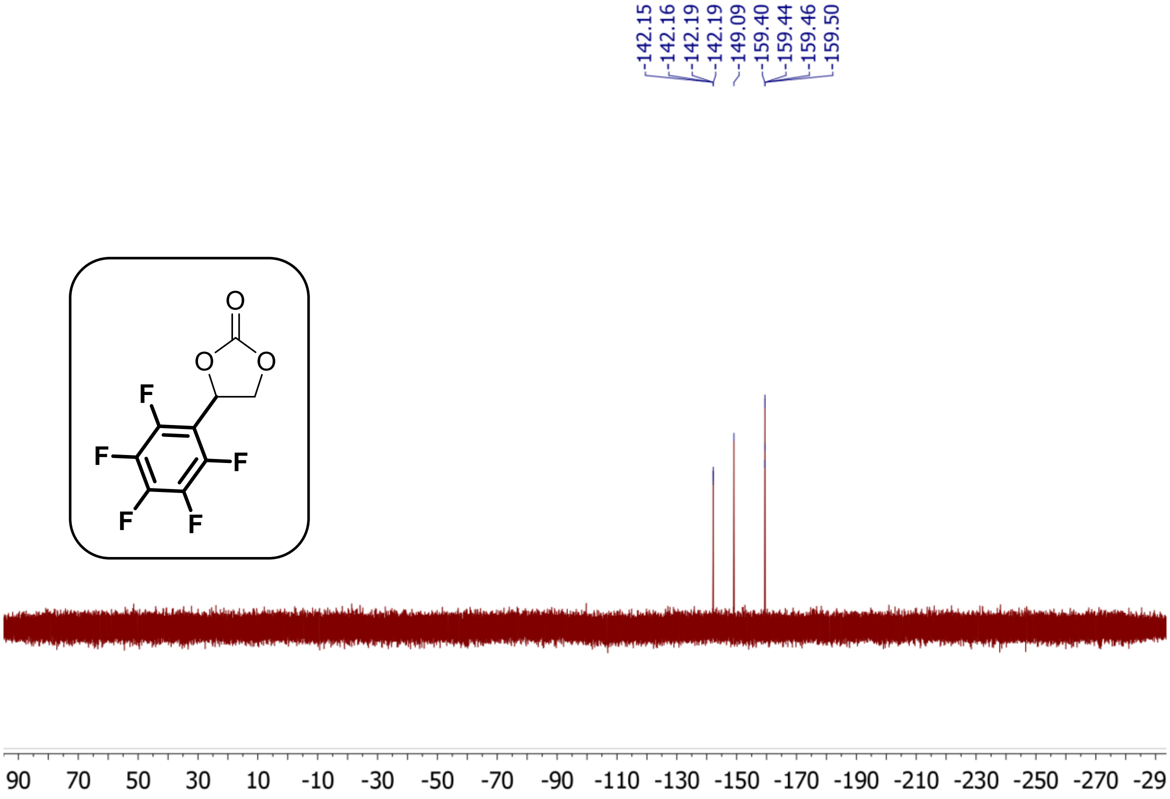

Supplementary Fig. 95. ^{1}H NMR (400 MHz, $CDCl_3$) spectrum of 2-((4-bromophenyl)(phenyl)methyl)-1,3,5-trimethylbenzene (56).

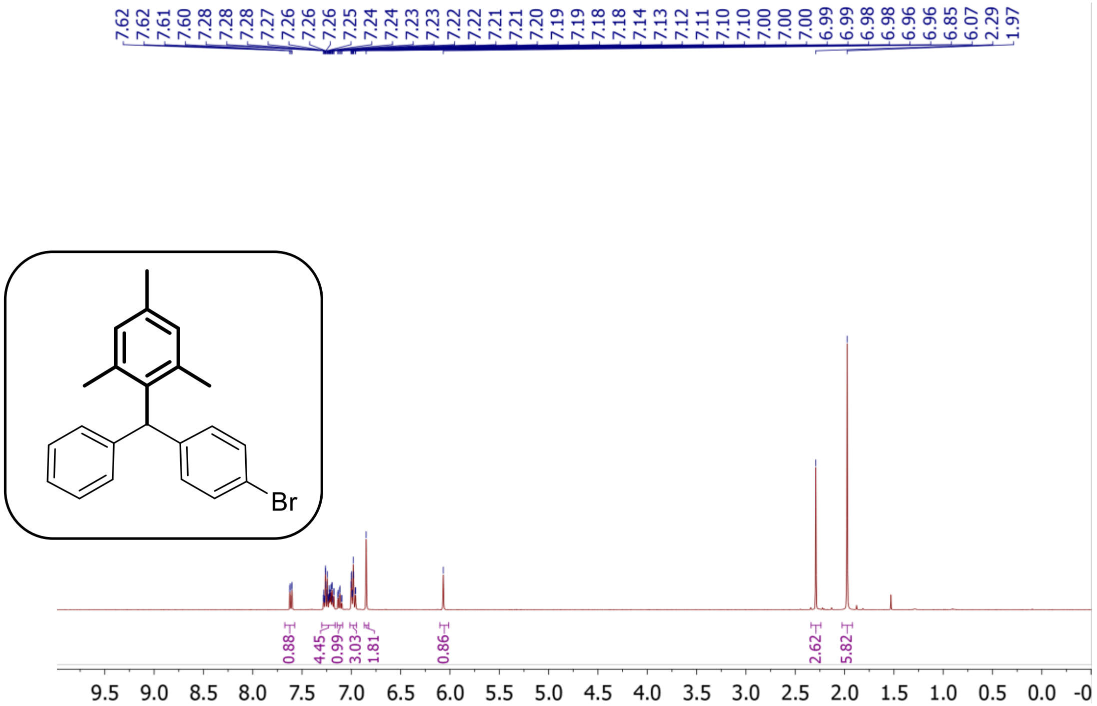

Supplementary Fig. 96. ^{13}C NMR (100 MHz, $CDCl_3$) spectrum of 2-((4-bromophenyl)(phenyl)methyl)-1,3,5-trimethylbenzene (56).

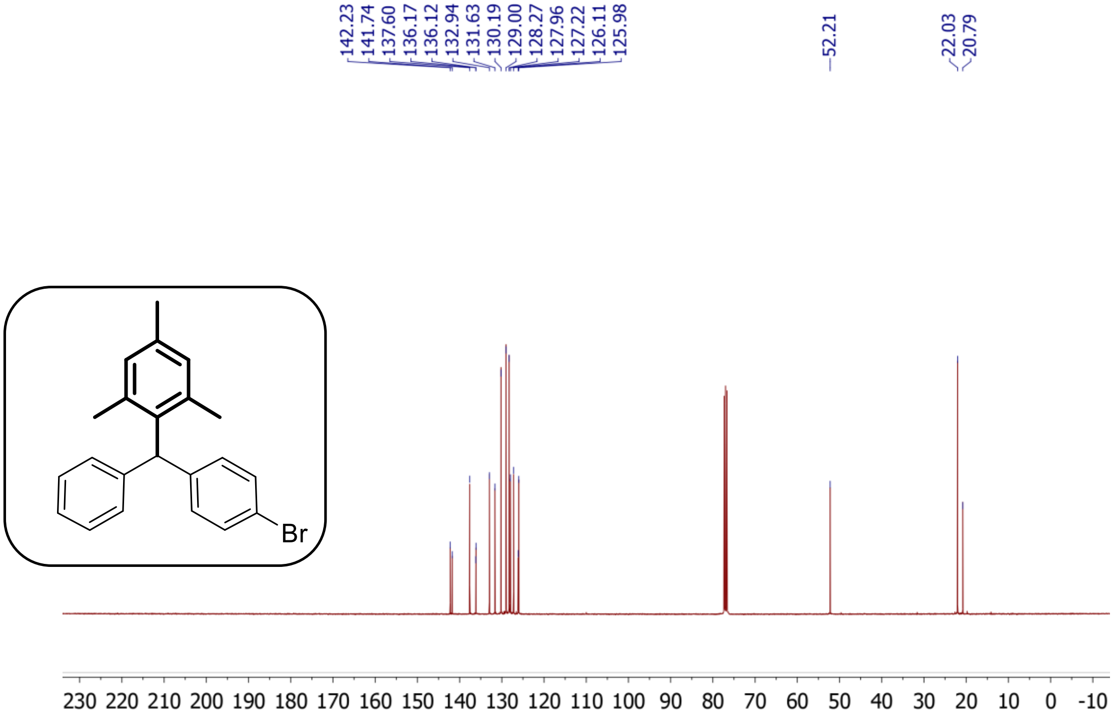

Supplementary Fig. 97. ^{1}H NMR (400 MHz, $CDCl_3$) spectrum of 1-((4-bromophenyl)(phenyl)methyl)-2,3,4,5,6-pentafluorobenzene (57).

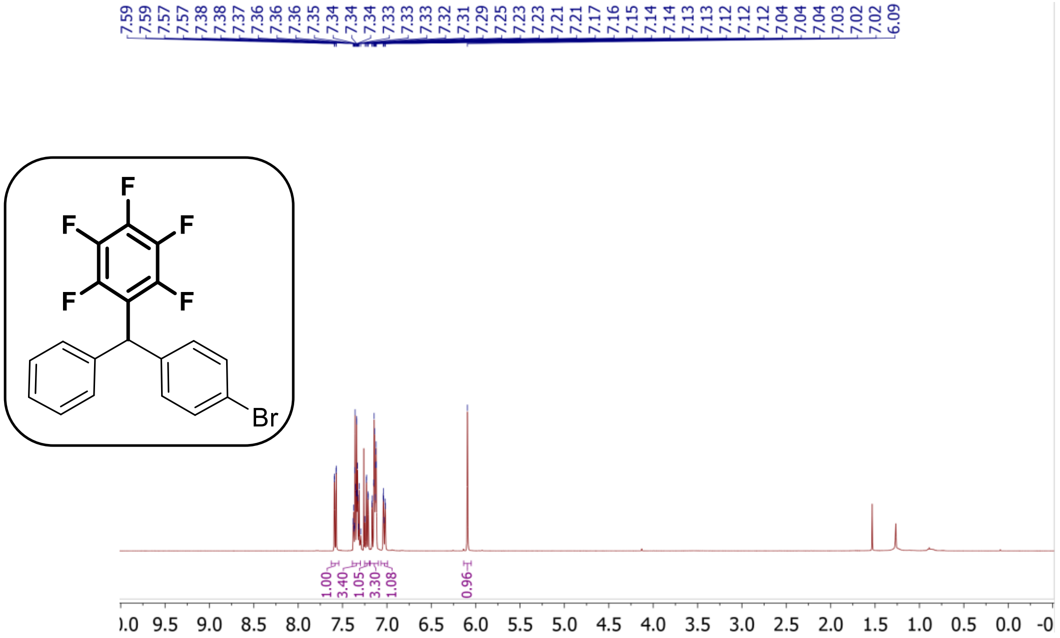

Supplementary Fig. 98. ^{13}C NMR (100 MHz, $CDCl_3$) spectrum of 1-((4-bromophenyl)(phenyl)methyl)-2,3,4,5,6-pentafluorobenzene (57).

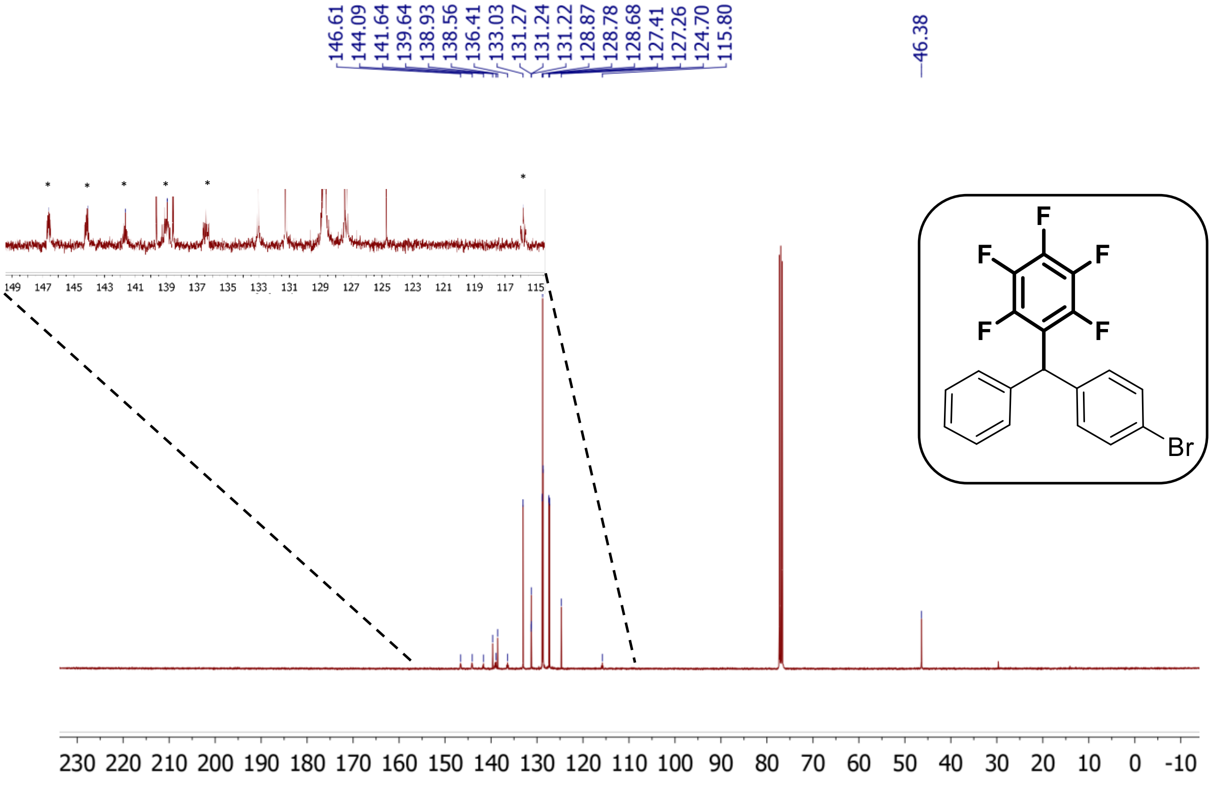

Supplementary Fig. 99. ^{19}F NMR (376 MHz, $CDCl_3$) spectrum of 1-((4-bromophenyl)(phenyl)methyl)-2,3,4,5,6-pentafluorobenzene (57).

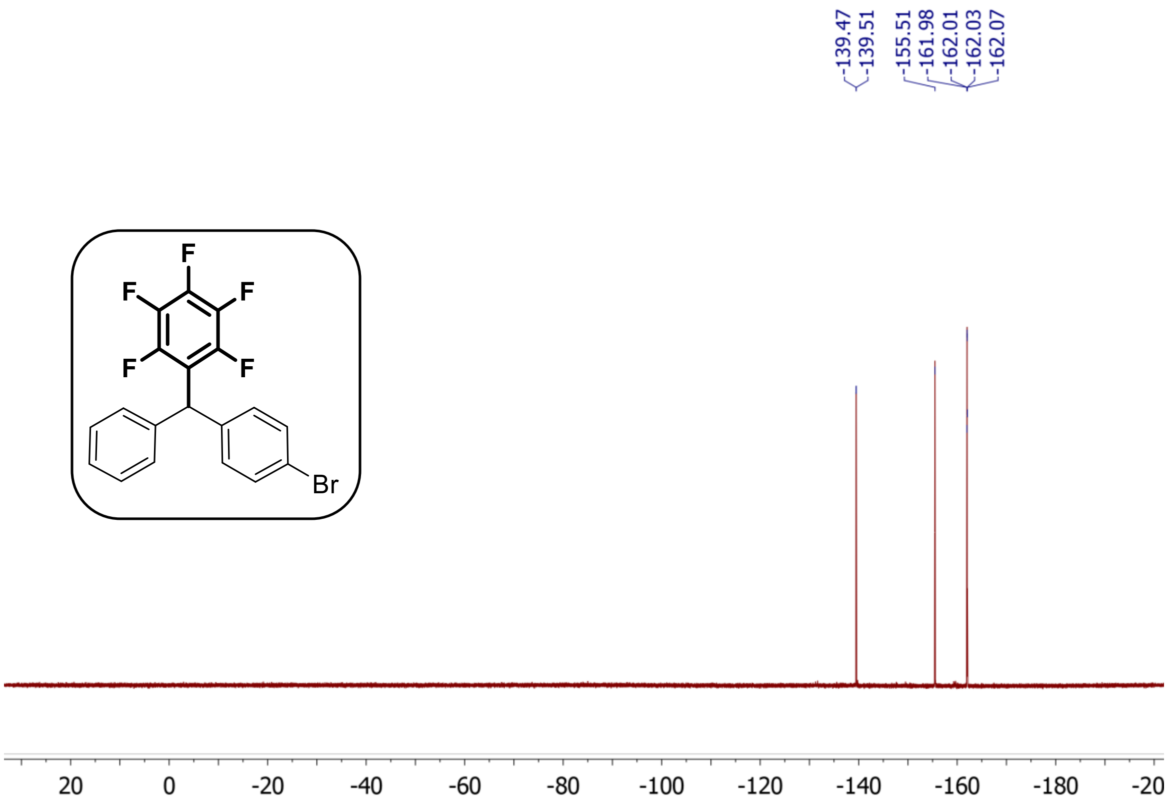

Supplementary Fig. 100. ^{1}H NMR (400 MHz, $CDCl_3$) spectrum of 4-((4-chlorophenyl)(o-tolyl)methyl)benzonitrile (58).

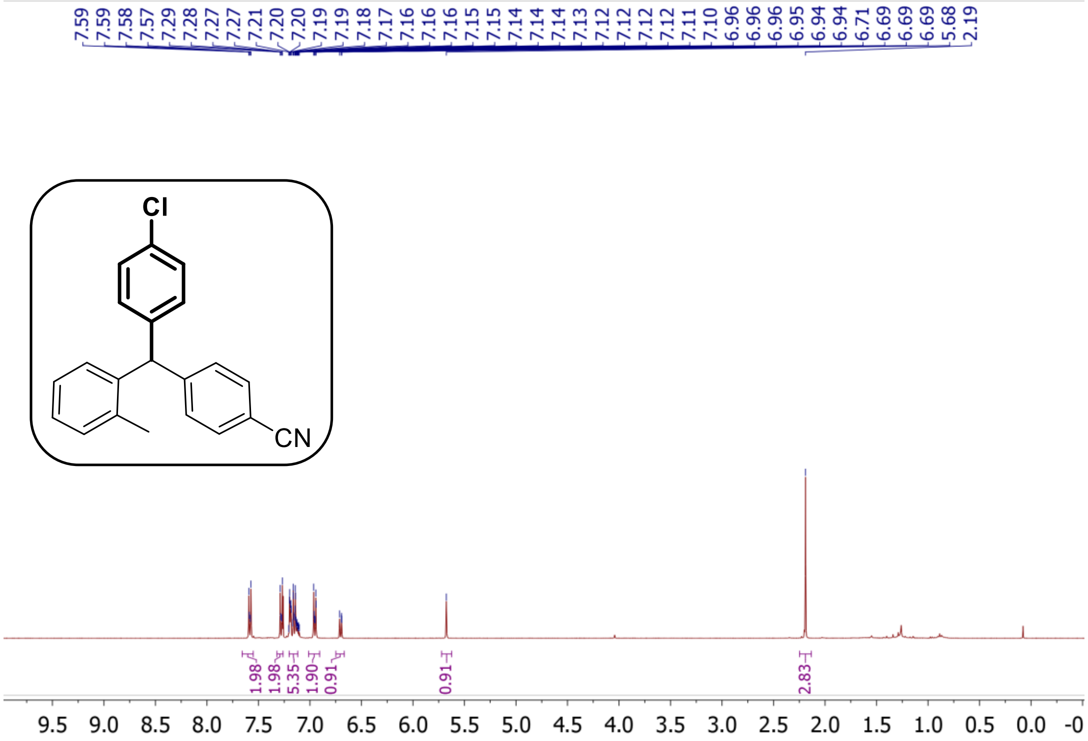

Supplementary Fig. 101. ^{13}C NMR (100 MHz, $CDCl_3$) spectrum of 4-((4-chlorophenyl)(o-tolyl)methyl)benzonitrile (58).

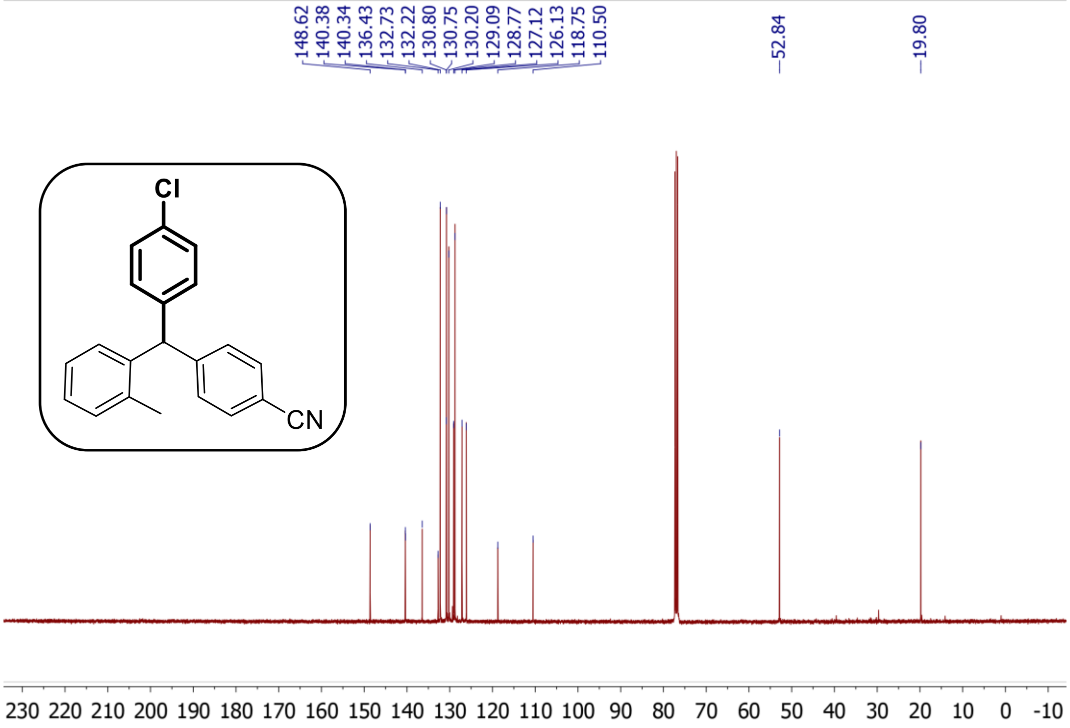

Supplementary Fig. 102. ^{1}H NMR (400 MHz, $CDCl_3$) spectrum of 1-mesityladamantane (59).

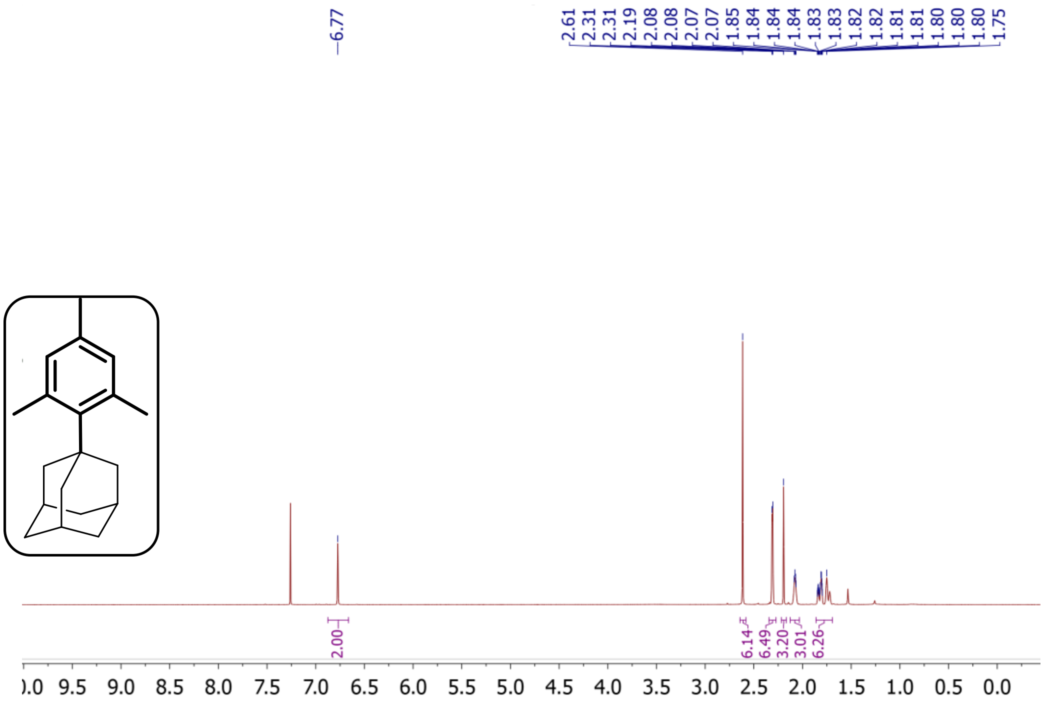

Supplementary Fig. 103. ^{13}C NMR (100 MHz, $CDCl_3$) spectrum of 1-mesityladamantane (59).

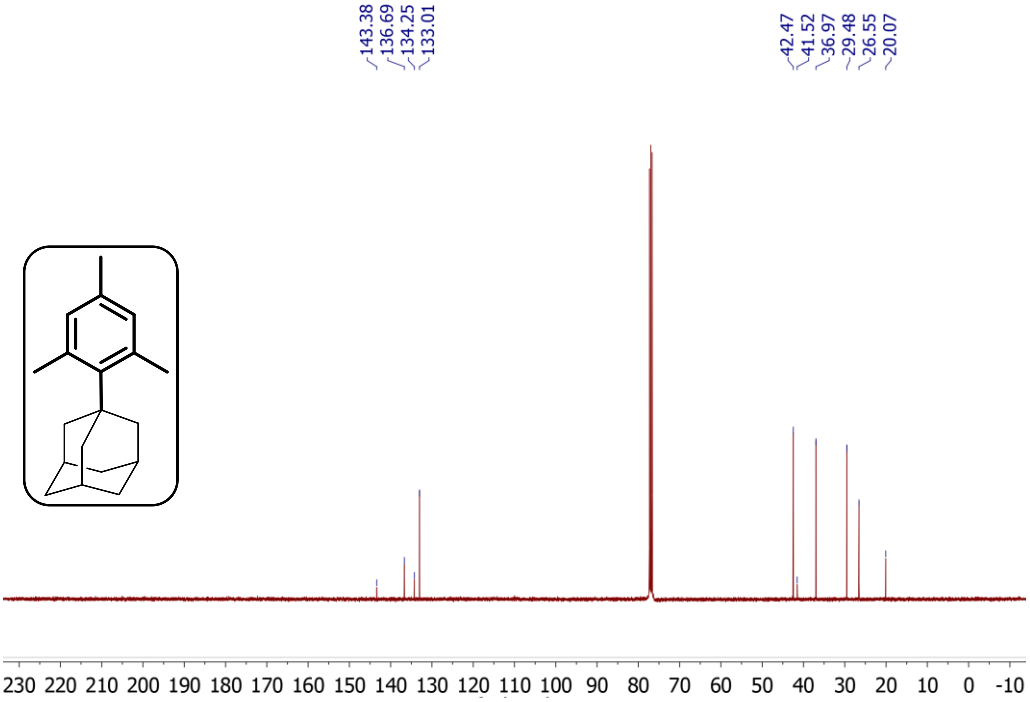

Supplementary Fig. 104. ¹H NMR (400 MHz, CDCl₃) spectrum of 1-(perfluorophenyl)adamantane (60).

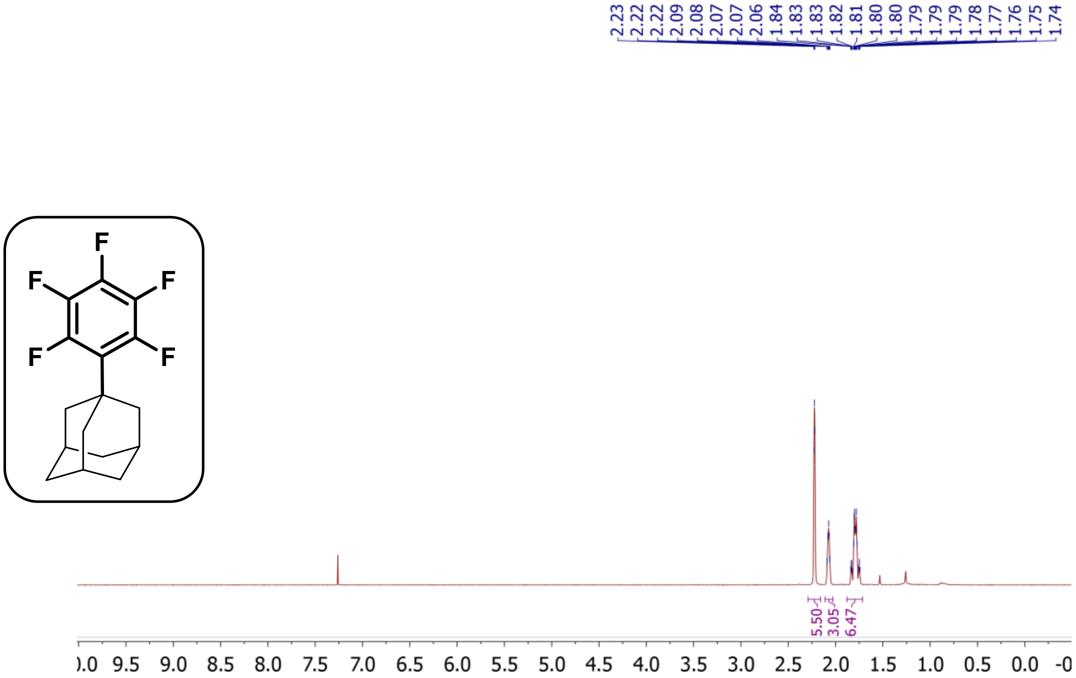

Supplementary Fig. 105. ¹³C NMR (100 MHz, CDCl₃) spectrum of 1-(perfluorophenyl)adamantane (60).

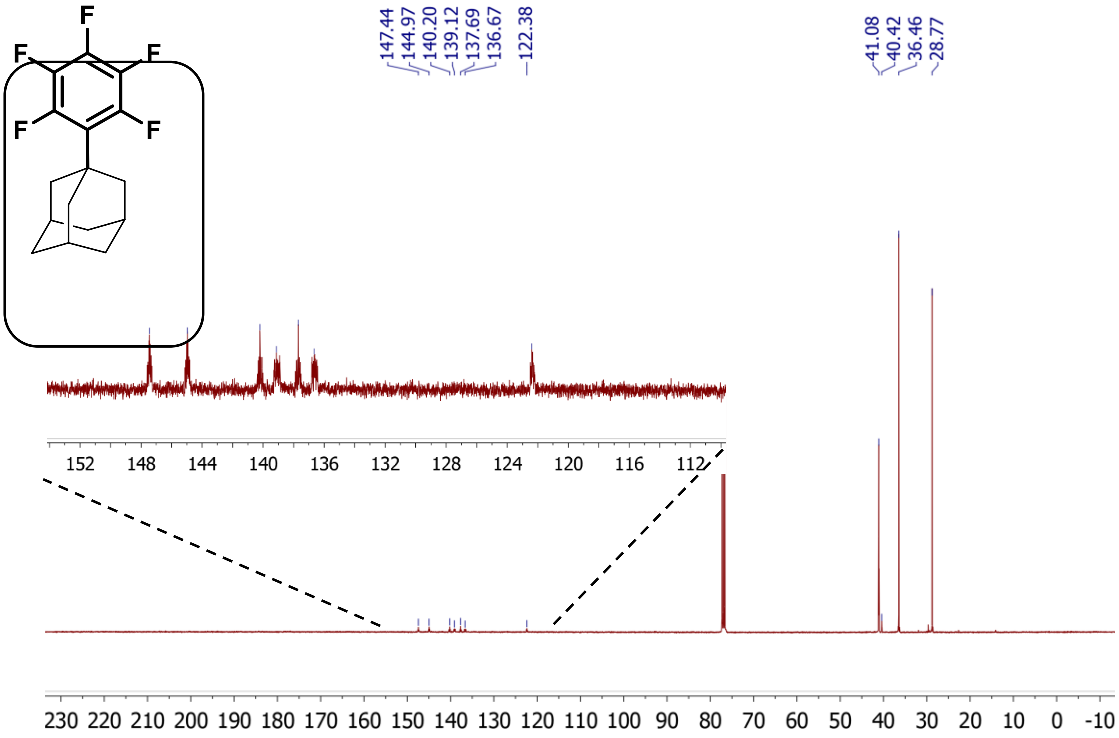

Supplementary Fig. 106. ^{19}**F NMR spectrum (376 MHz, CDCl$_3$) of 1-(perfluorophenyl)adamantane (60).**

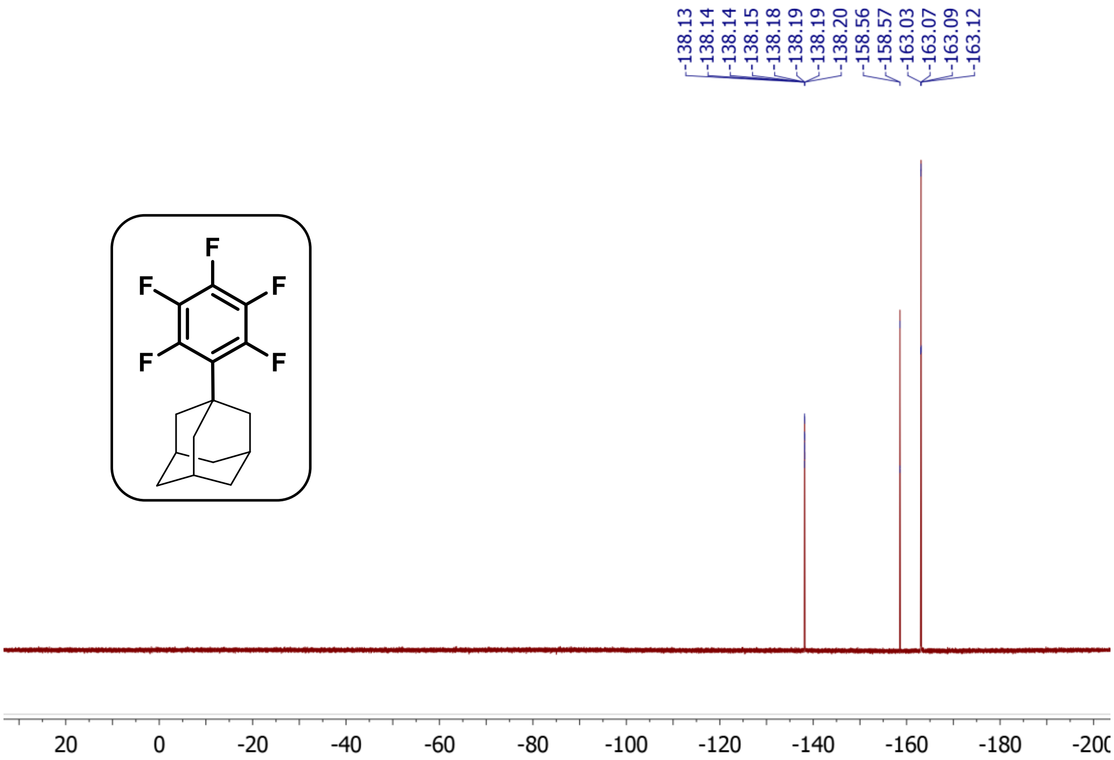

Supplementary Fig. 107. ^{1}H NMR (400 MHz, $CDCl_3$) spectrum of 1-(4-chlorophenyl)adamantane (61)

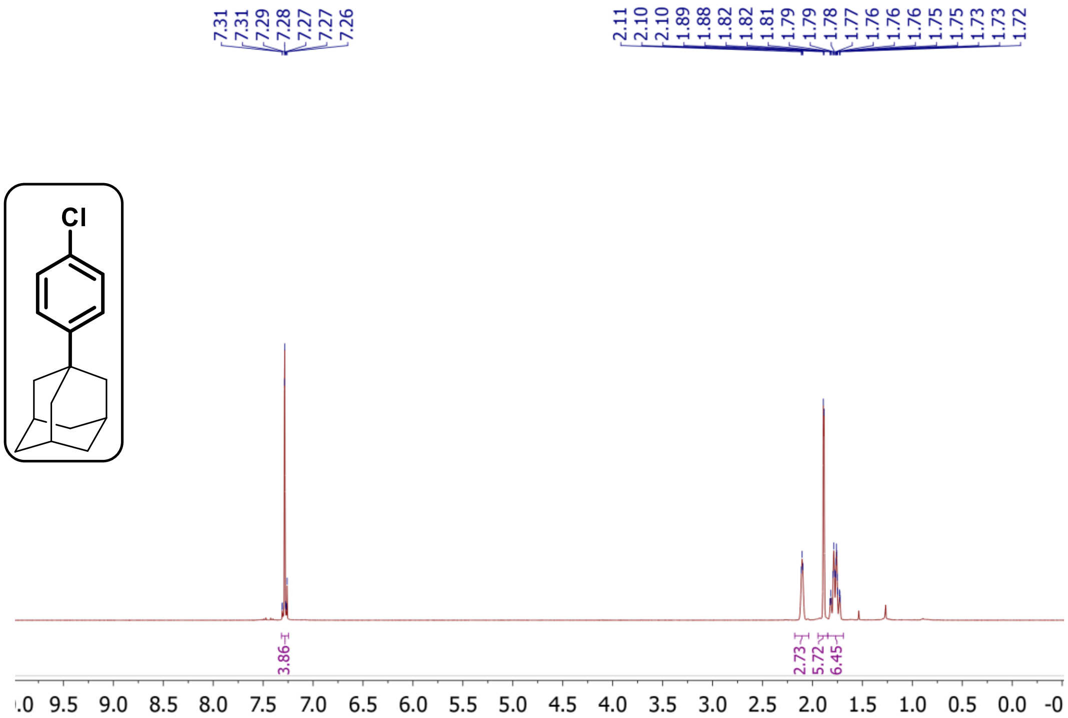

Supplementary Fig. 108. ^{13}C NMR (100 MHz, $CDCl_3$) spectrum of 1-(4-chlorophenyl)adamantane (61)

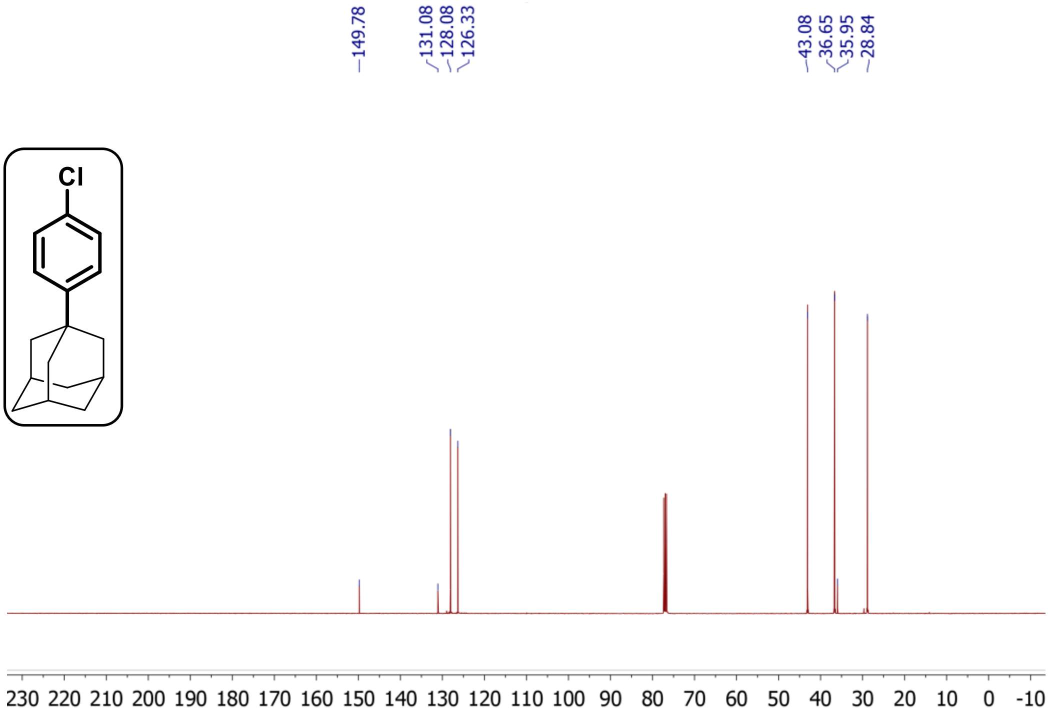

Supplementary Fig. 109. **^{1}H NMR (400 MHz, $CDCl_3$) spectrum of 1-ferrocenyladamantane (62).**

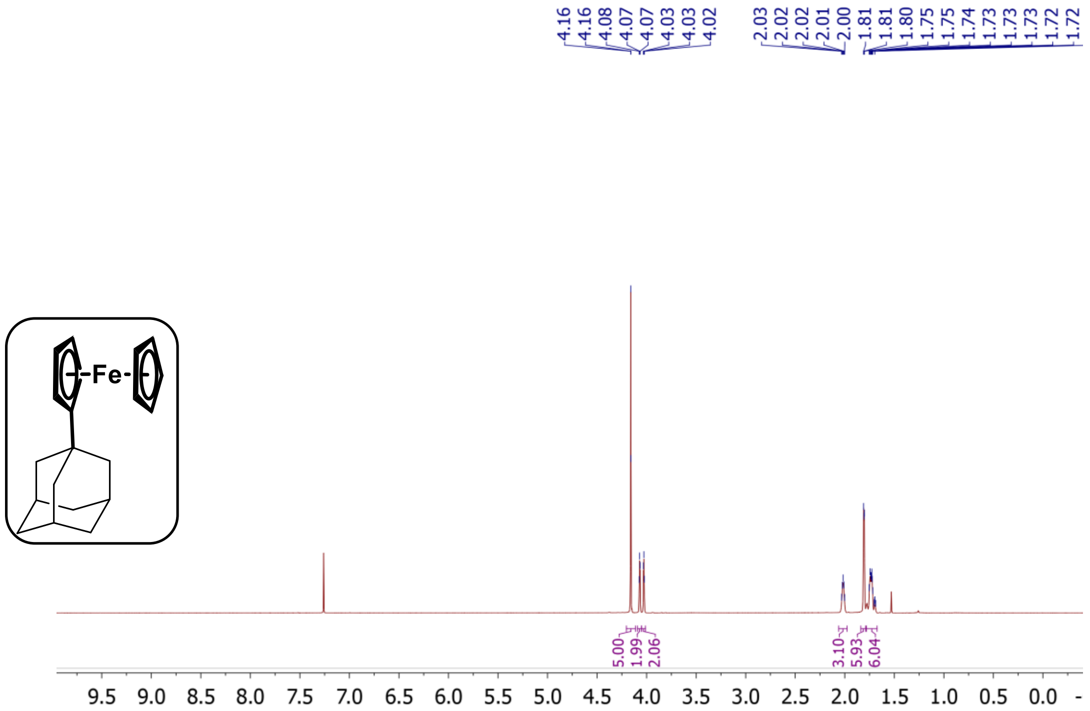

Supplementary Fig. 110. **^{13}C NMR (100 MHz, $CDCl_3$) spectrum of 1-ferrocenyladamantane (62).**

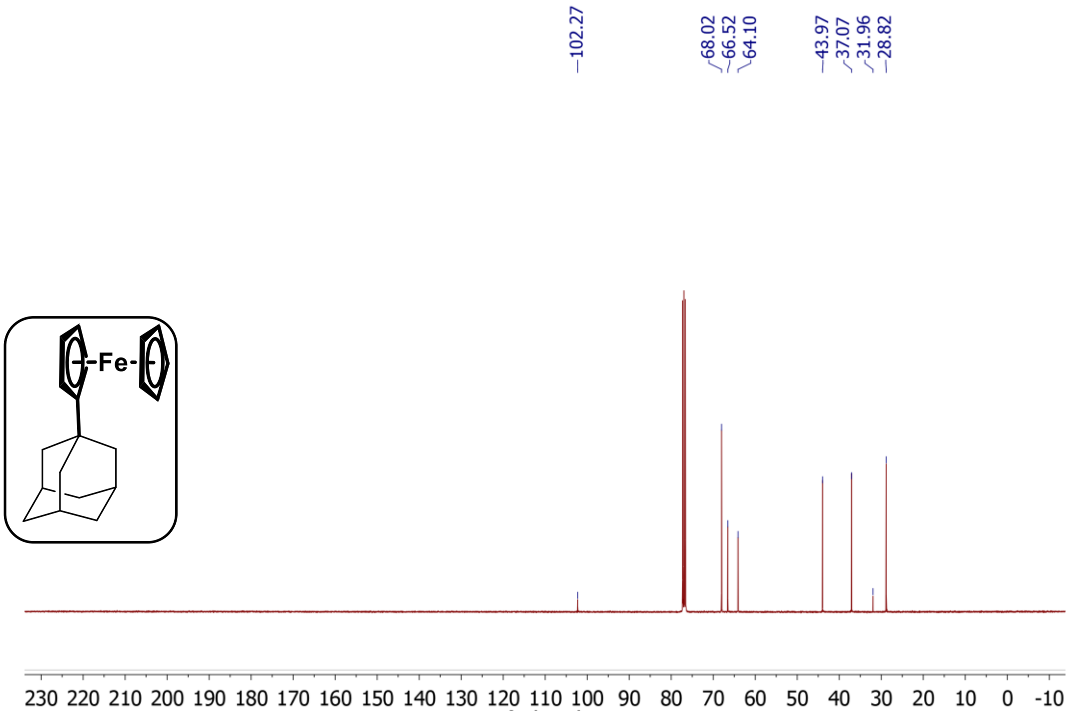

Supplementary Fig. 111. ^{1}H NMR (400 MHz, $CDCl_3$) spectrum of 3-mesityl-3-methyl-1-phenylindolin-2-one (63).

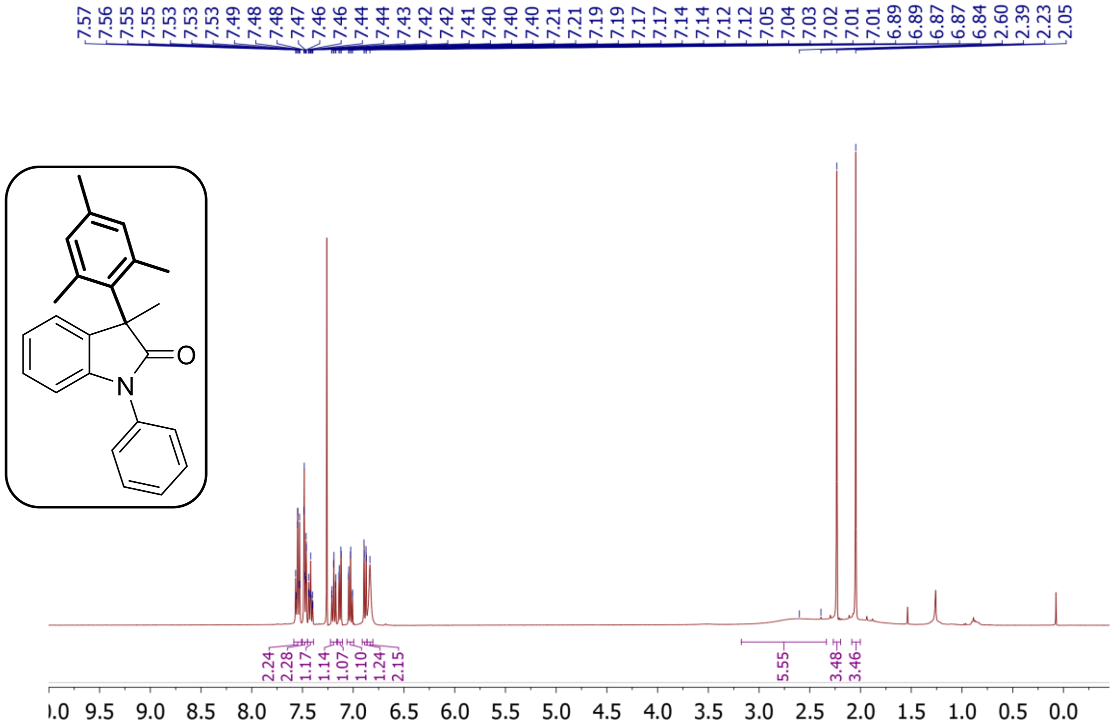

Supplementary Fig. 112. ^{13}C NMR (100 MHz, $CDCl_3$) spectrum of 3-mesityl-3-methyl-1-phenylindolin-2-one (63).

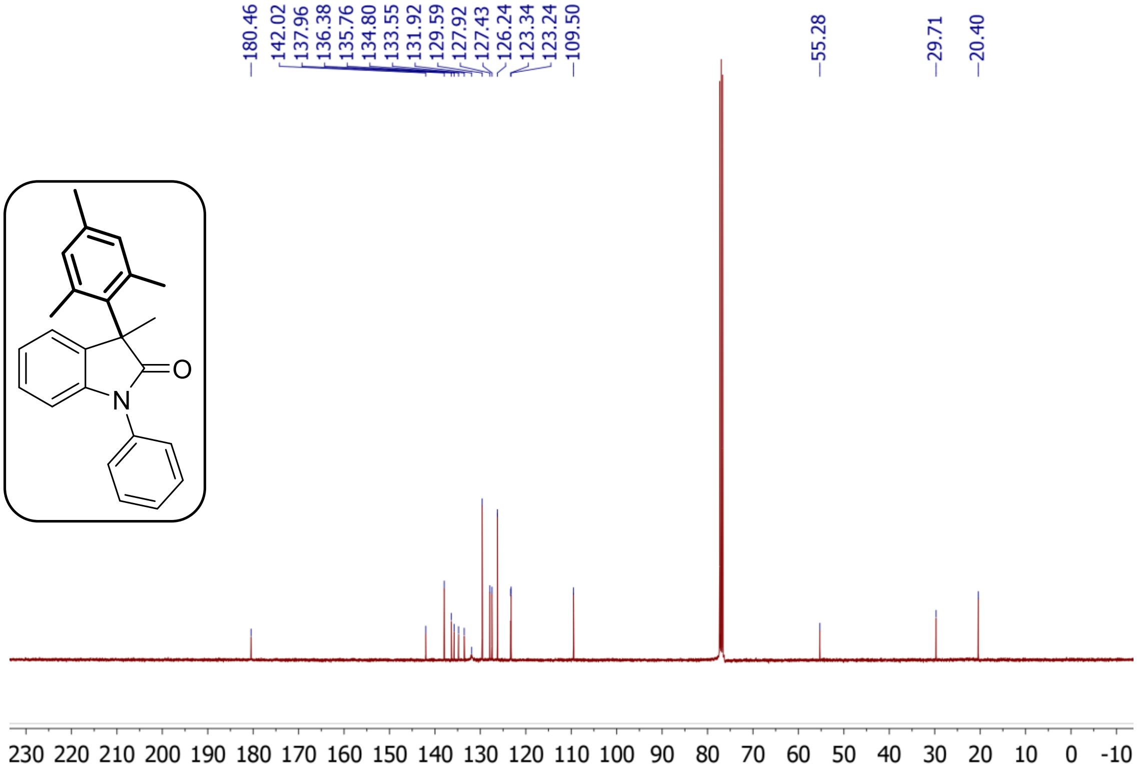

Supplementary Fig. 113. ¹H NMR (400 MHz, $CDCl_3$) spectrum of 3-methyl-3-(perfluorophenyl)-1-phenylindolin-2-one (64).

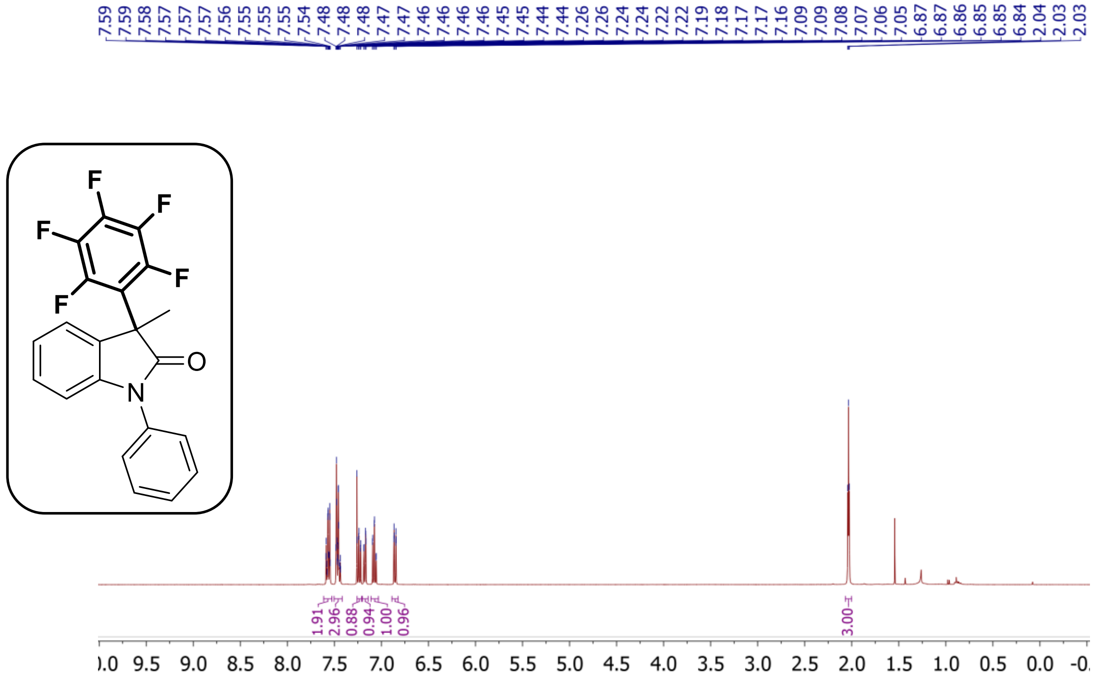

Supplementary Fig. 114. ¹³C NMR (100 MHz, $CDCl_3$) spectrum of 3-methyl-3-(perfluorophenyl)-1-phenylindolin-2-one (64).

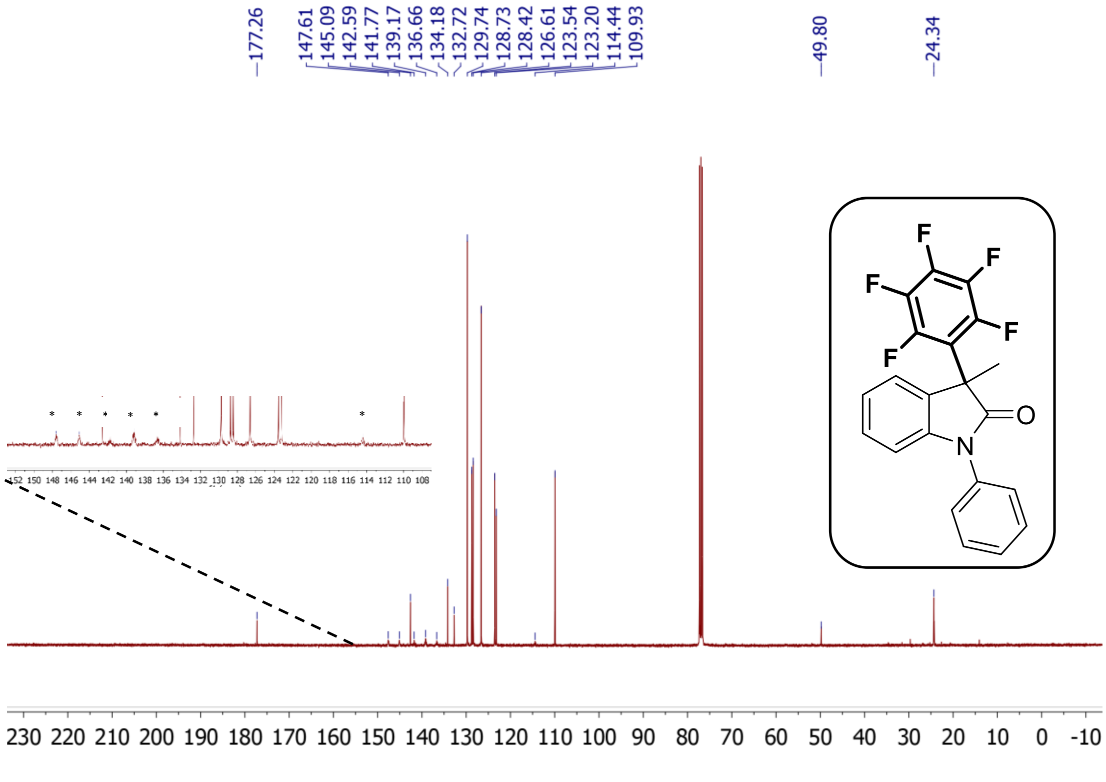

Supplementary Fig. 115. **^{19}F NMR (376 MHz, $CDCl_3$) spectrum of 3-methyl-3-(perfluorophenyl)-1-phenylindolin-2-one (64).**

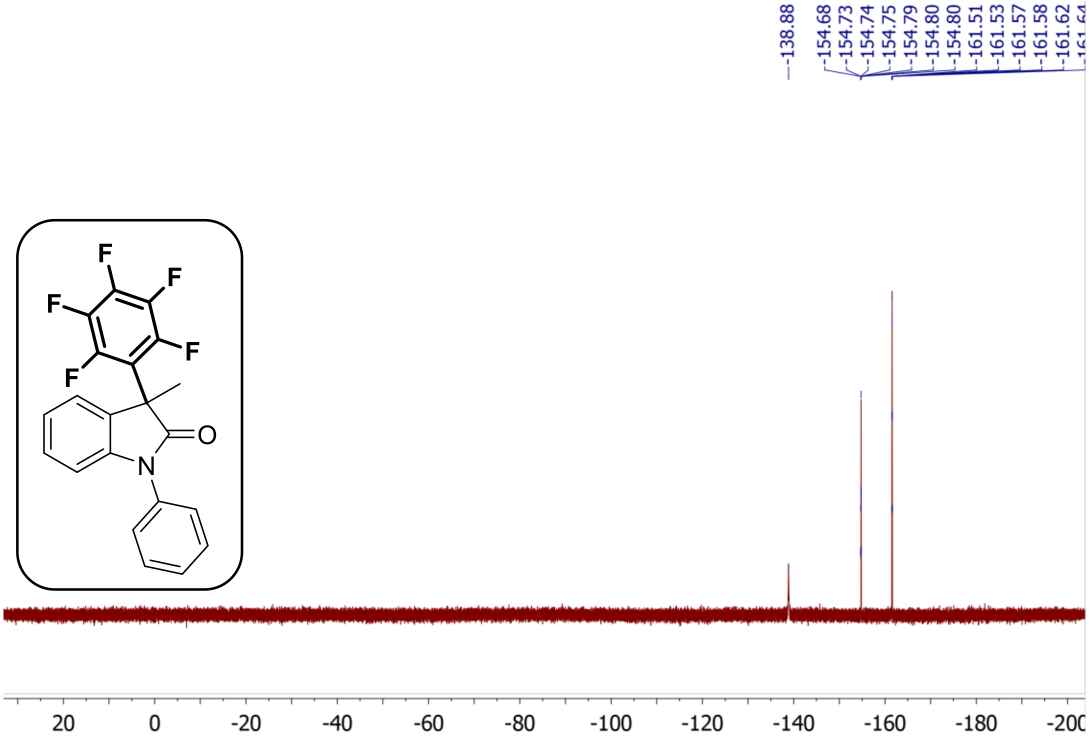

Supplementary Fig. 116. ^{1}H NMR (400 MHz, $CDCl_3$) spectrum of 3-methyl-3-(3-methylbenzo[b]thiophen-2-yl)-1-phenylindolin-2-one (65).

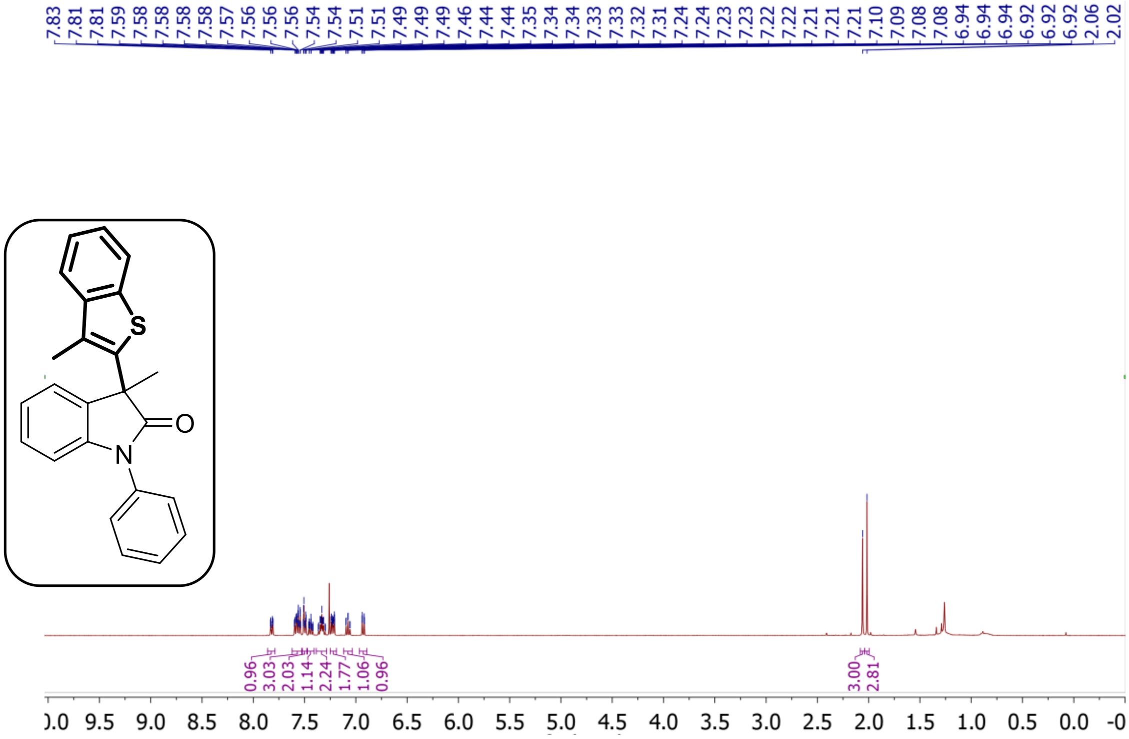

Supplementary Fig. 117. ^{13}C NMR (100 MHz, $CDCl_3$) spectrum of 3-methyl-3-(3-methylbenzo[b]thiophen-2-yl)-1-phenylindolin-2-one (65).

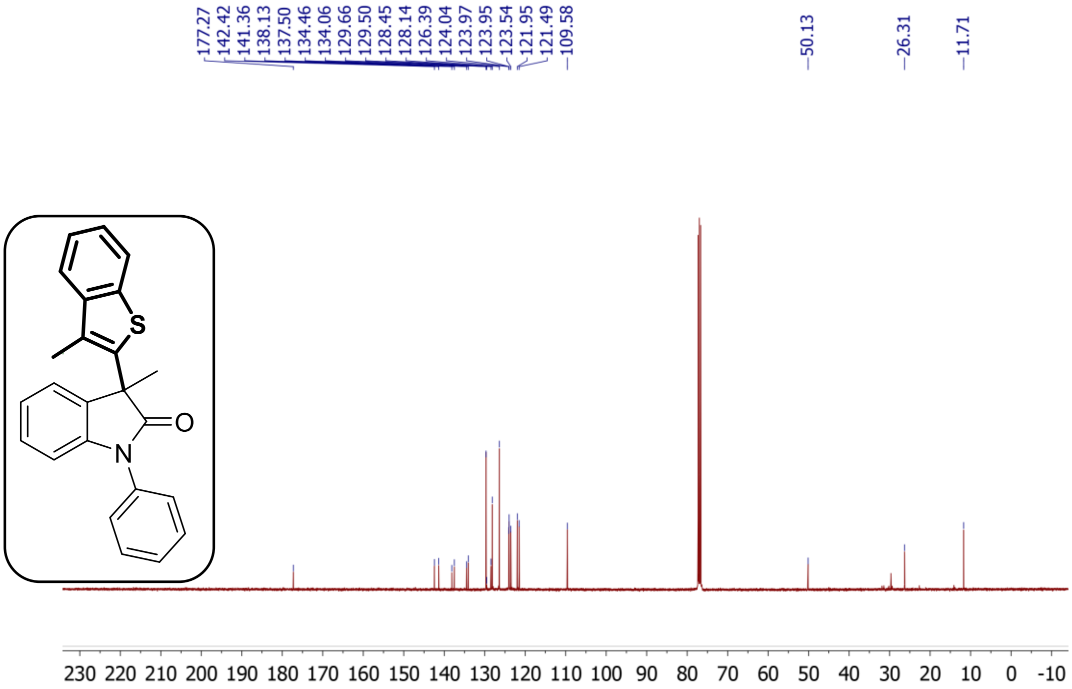

Supplementary Fig. 118. ^{1}H NMR (400 MHz, $CDCl_3$) spectrum of 3-methyl-3-(3-methylbenzofuran-2-yl)-1-phenylindolin-2-one (66).

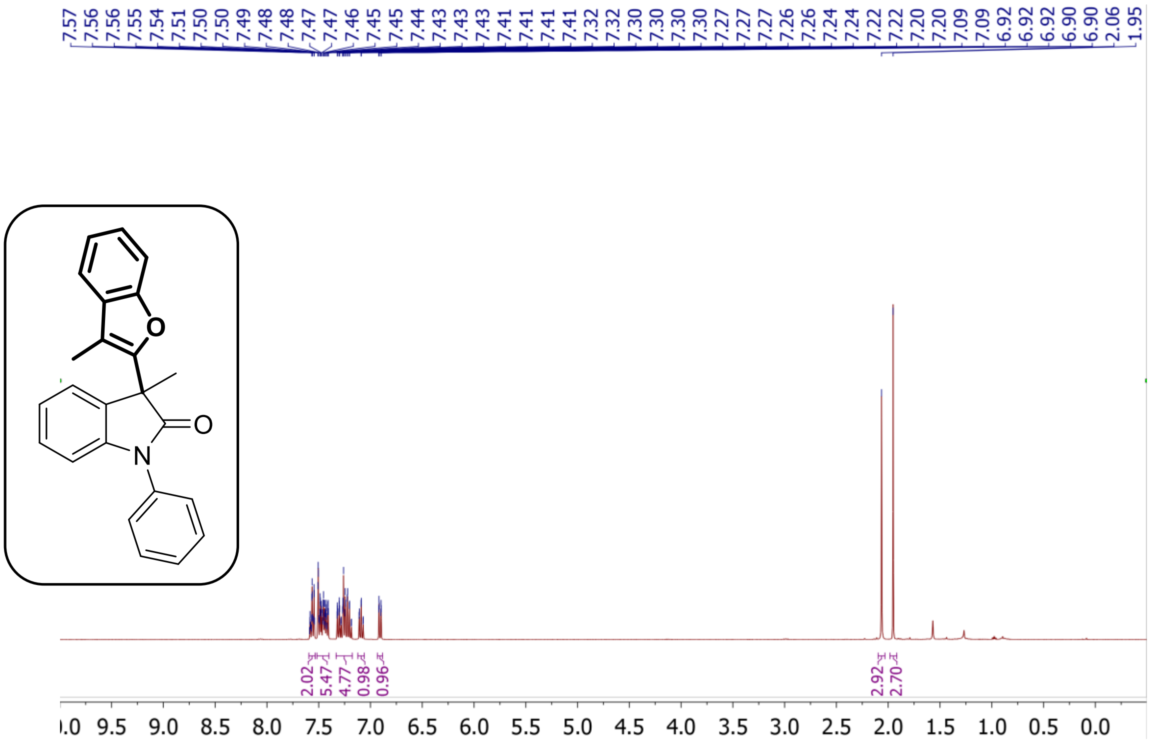

Supplementary Fig. 119. ^{13}C NMR (100 MHz, $CDCl_3$) spectrum of 3-methyl-3-(3-methylbenzofuran-2-yl)-1-phenylindolin-2-one (66).

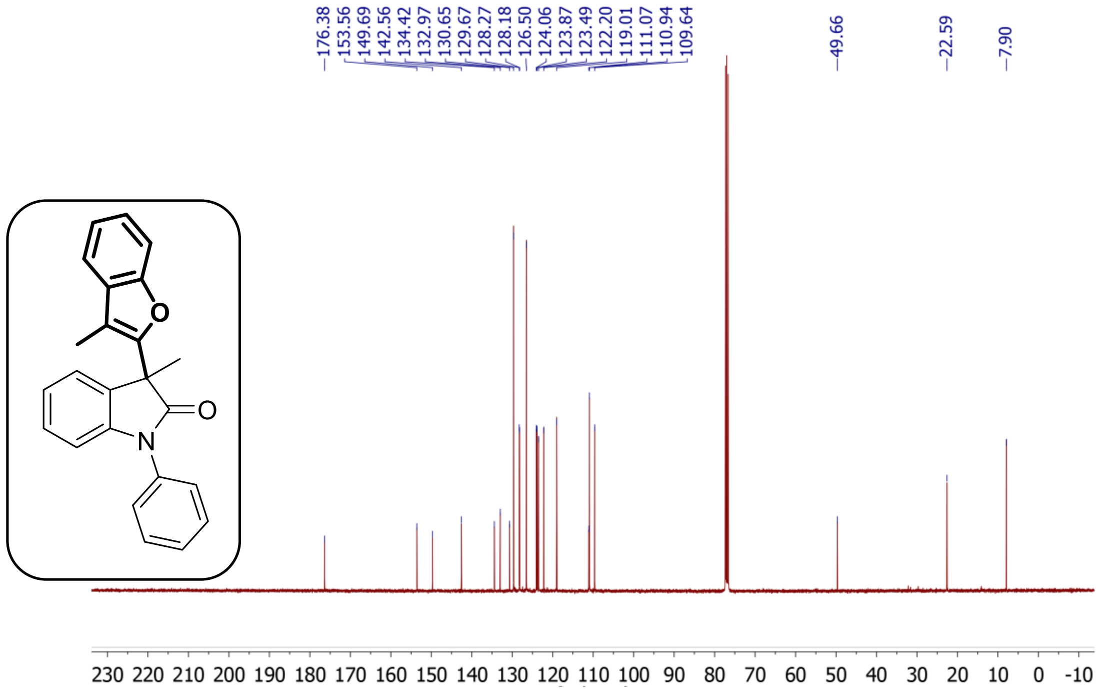

Supplementary Fig. 120. ^{1}**H NMR (400 MHz, CDCl**$_3$**) spectrum of 2-(adamantan-1-yl)-3-methylbenzofuran (67).**

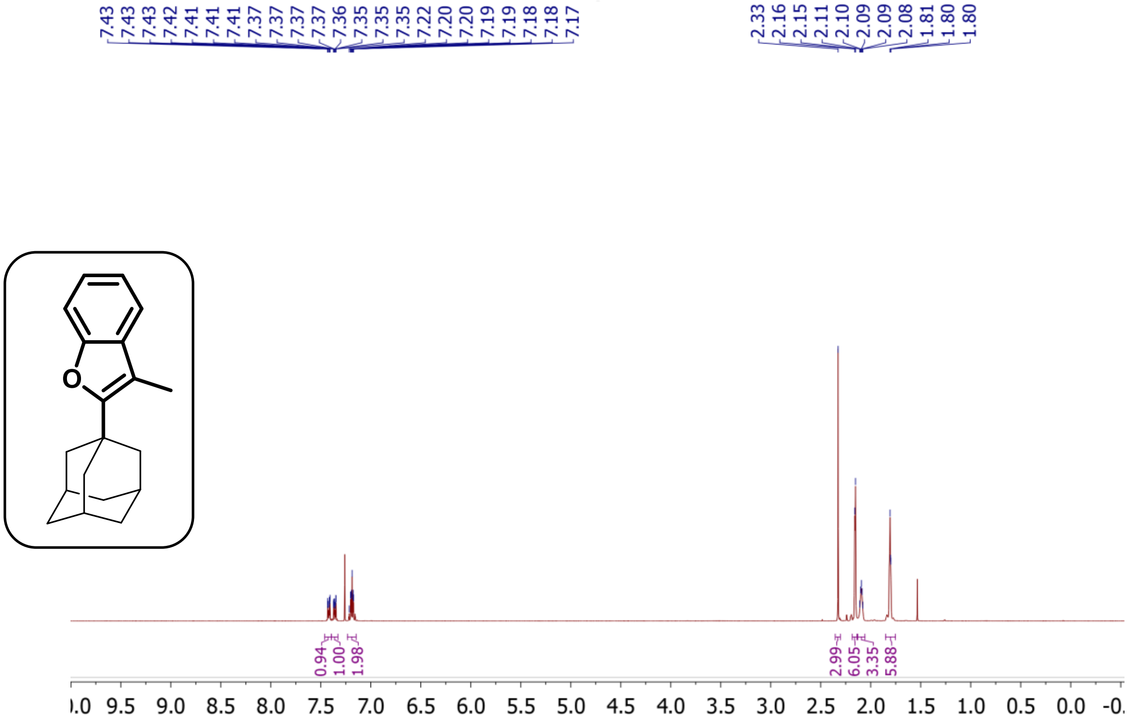

Supplementary Fig. 121. ^{13}**C NMR (100 MHz, CDCl**$_3$**) spectrum of 2-(adamantan-1-yl)-3-methylbenzofuran (67).**

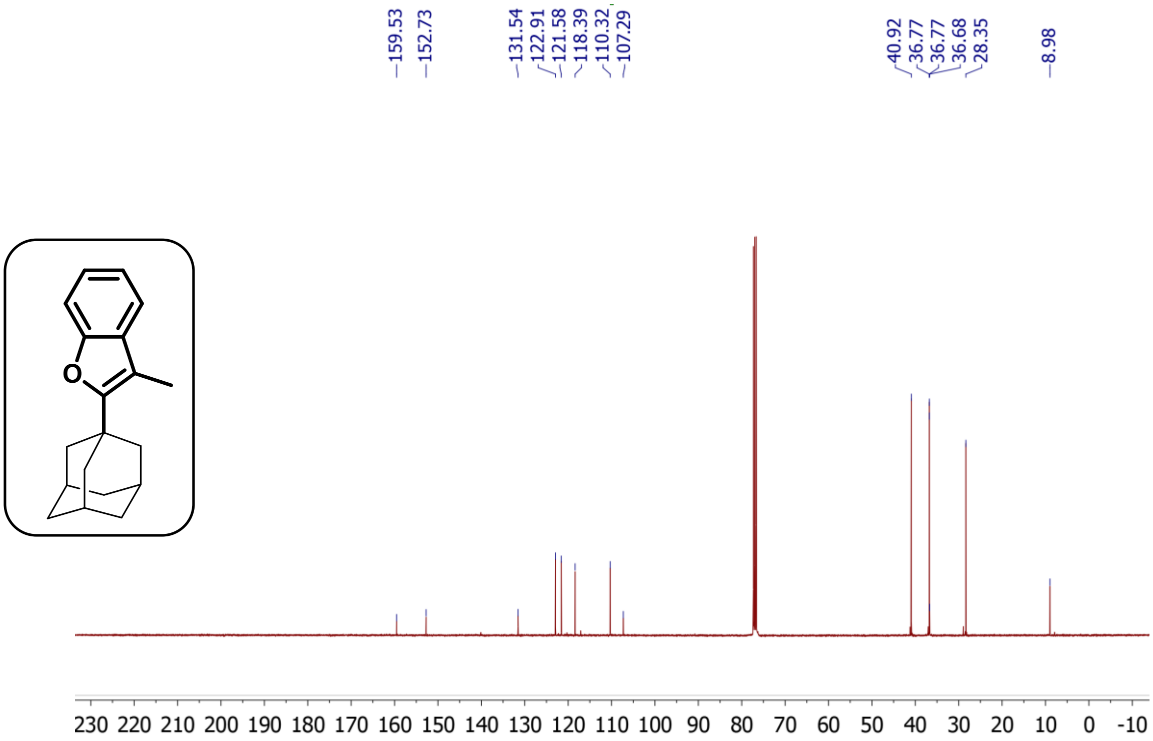

Supplementary Fig. 122. **^{1}H NMR (400 MHz, $CDCl_3$) spectrum of 2-(adamantan-1-yl)-3-methylbenzo[b]thiophene (68).**

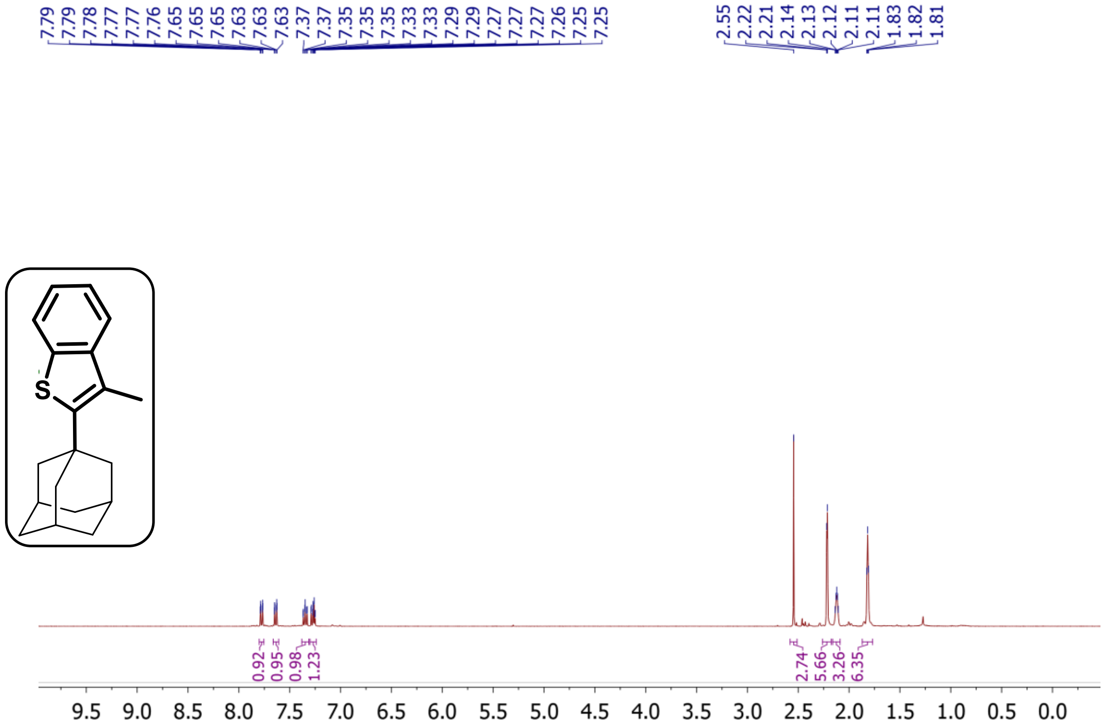

Supplementary Fig. 123. **^{13}C NMR (100 MHz, $CDCl_3$) spectrum of 2-(adamantan-1-yl)-3-methylbenzo[b]thiophene (68).**

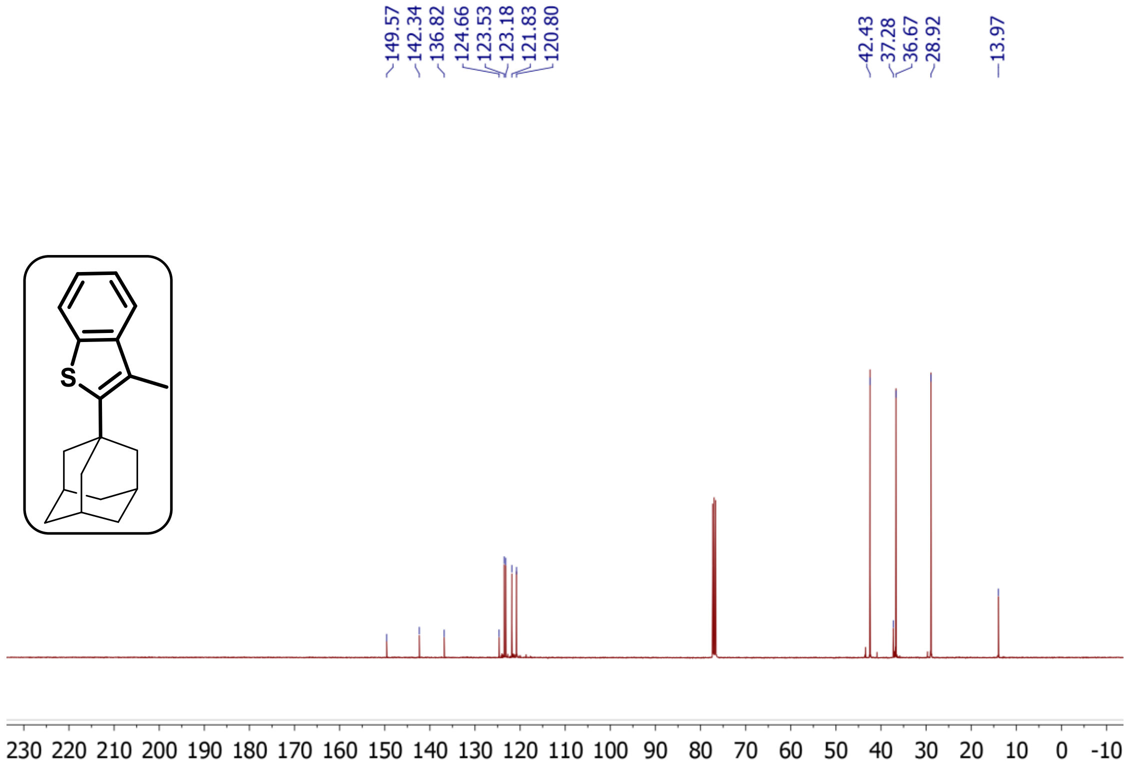

Supplementary Fig. 124. **^{1}H NMR (400 MHz, $CDCl_3$) spectrum of 2-(adamantan-1-yl)-3,4-dimethylthiophene (69).**

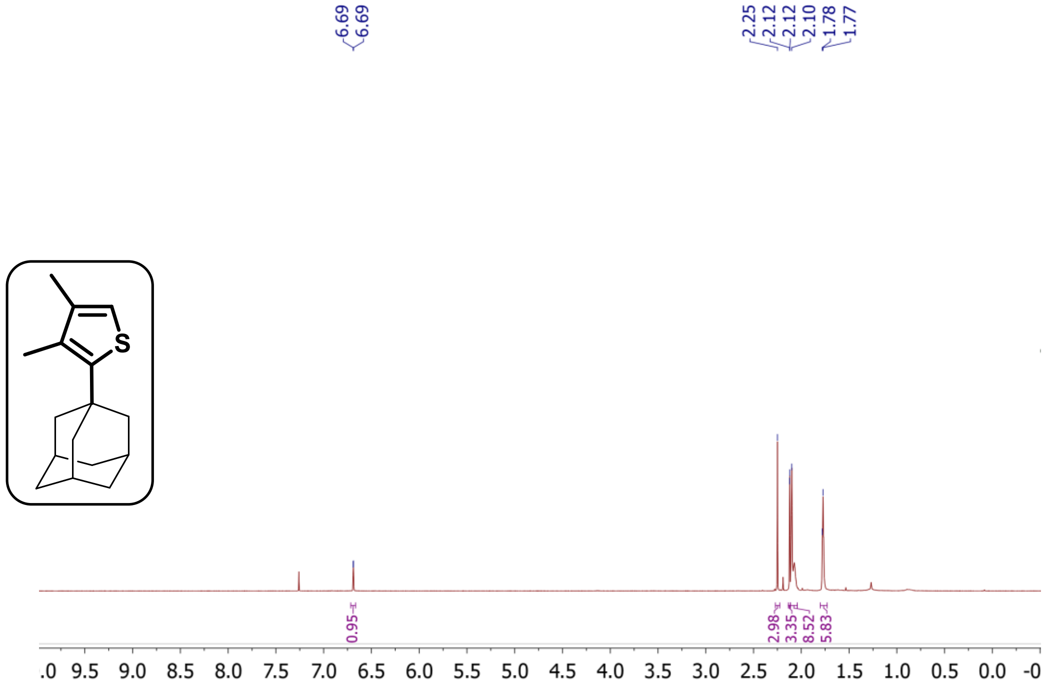

Supplementary Fig. 125. **^{13}C NMR (100 MHz, $CDCl_3$) spectrum of 2-(adamantan-1-yl)-3,4-dimethylthiophene (69).**

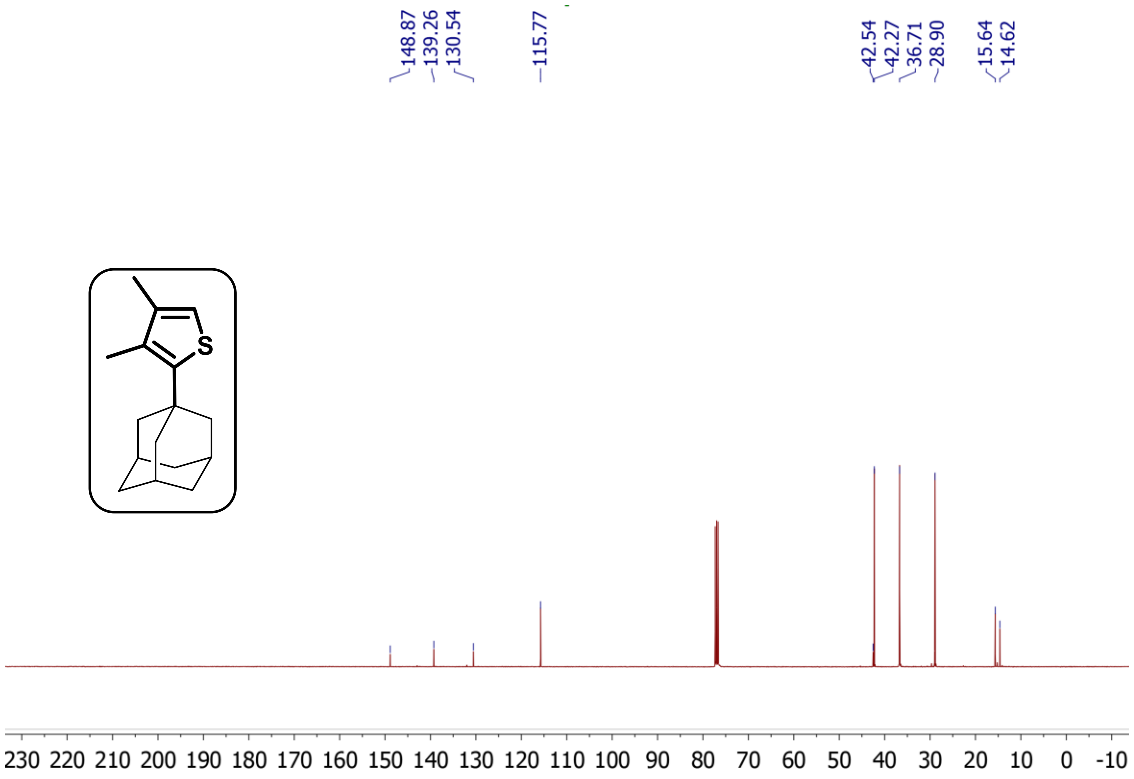

Supplementary Fig. 126. ^{1}**H NMR (400 MHz, CDCl**$_3$**) spectrum of (1*S*,2*S*,4*S*,5*R*)-2-((*S*)-(6-methoxyquinolin-4-yl)(phenyl)methyl)-5-vinylquinuclidene (71).**

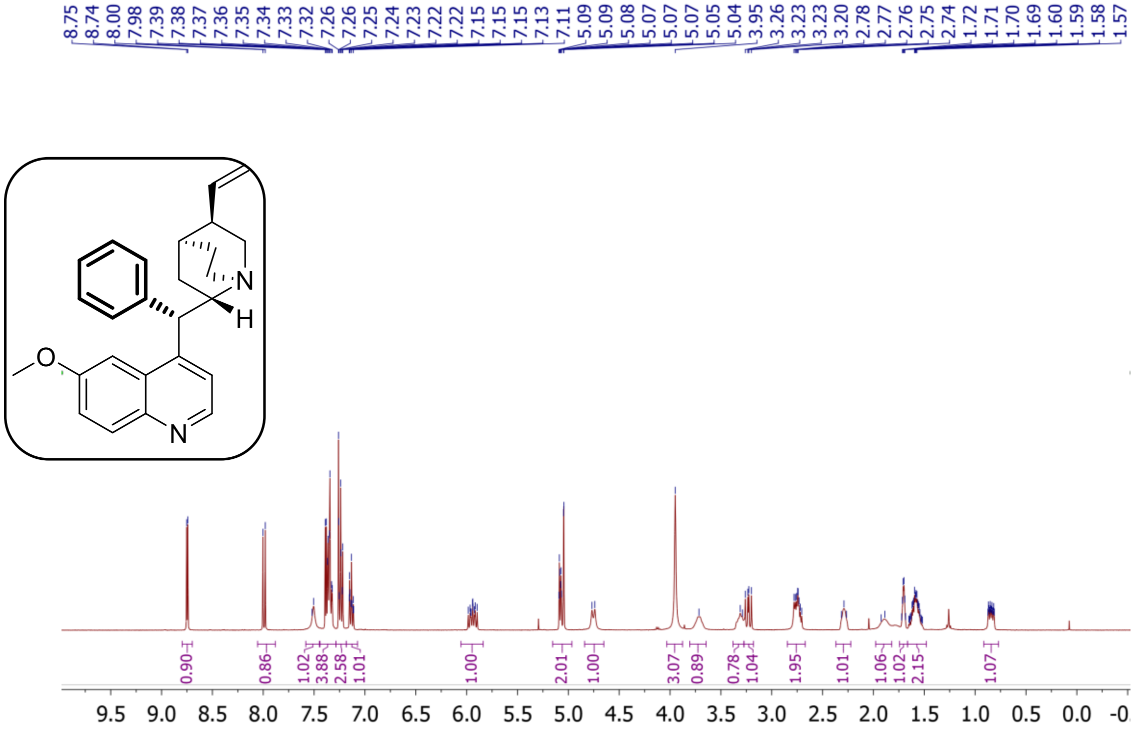

Supplementary Fig. 127. ^{13}**C NMR (100 MHz, CDCl**$_3$**) spectrum of (1*S*,2*S*,4*S*,5*R*)-2-((*S*)-(6-methoxyquinolin-4-yl)(phenyl)methyl)-5-vinylquinuclidene (71).**

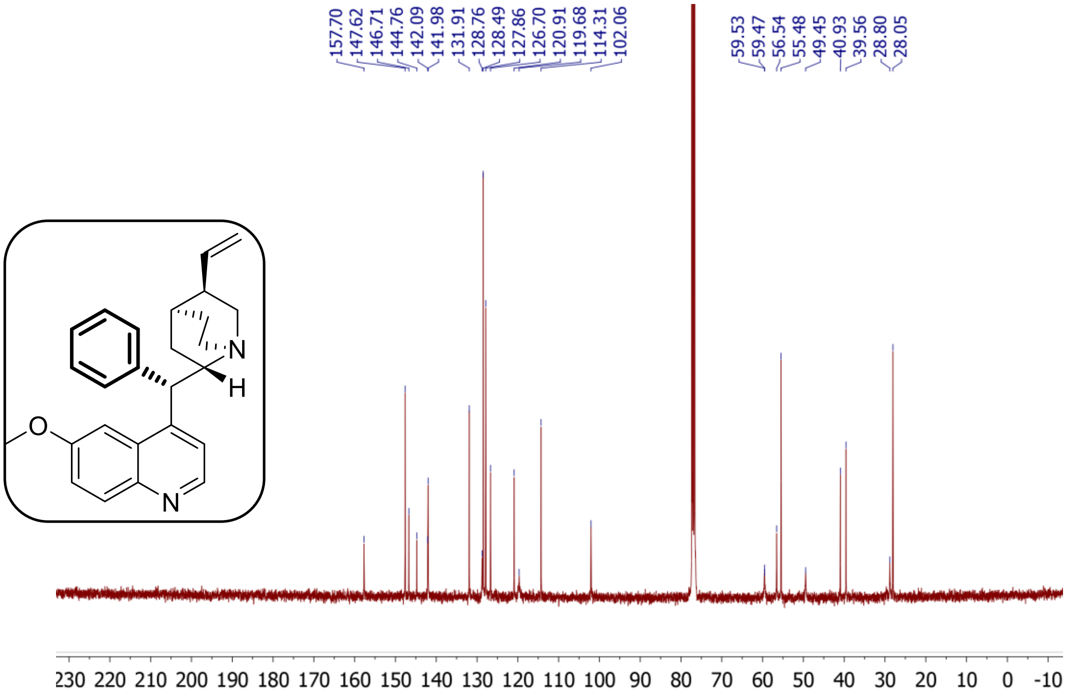

Supplementary Fig. 128. ^{1}**H NMR (400 MHz,** $CDCl_3$**) spectrum of 5-((2-chlorophenyl)(4-fluorophenyl)(phenyl)methyl)pyrimidine (72).**

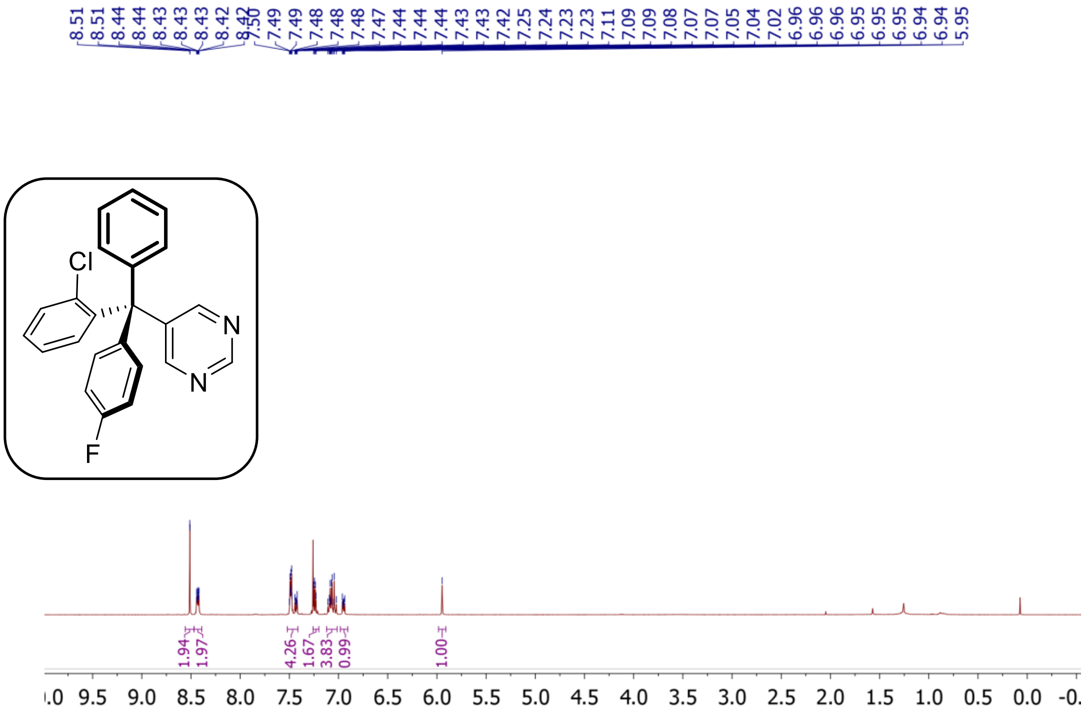

Supplementary Fig. 129. ^{13}**C NMR (100 MHz,** $CDCl_3$**) spectrum of 5-((2-chlorophenyl)(4-fluorophenyl)(phenyl)methyl)pyrimidine (72).**

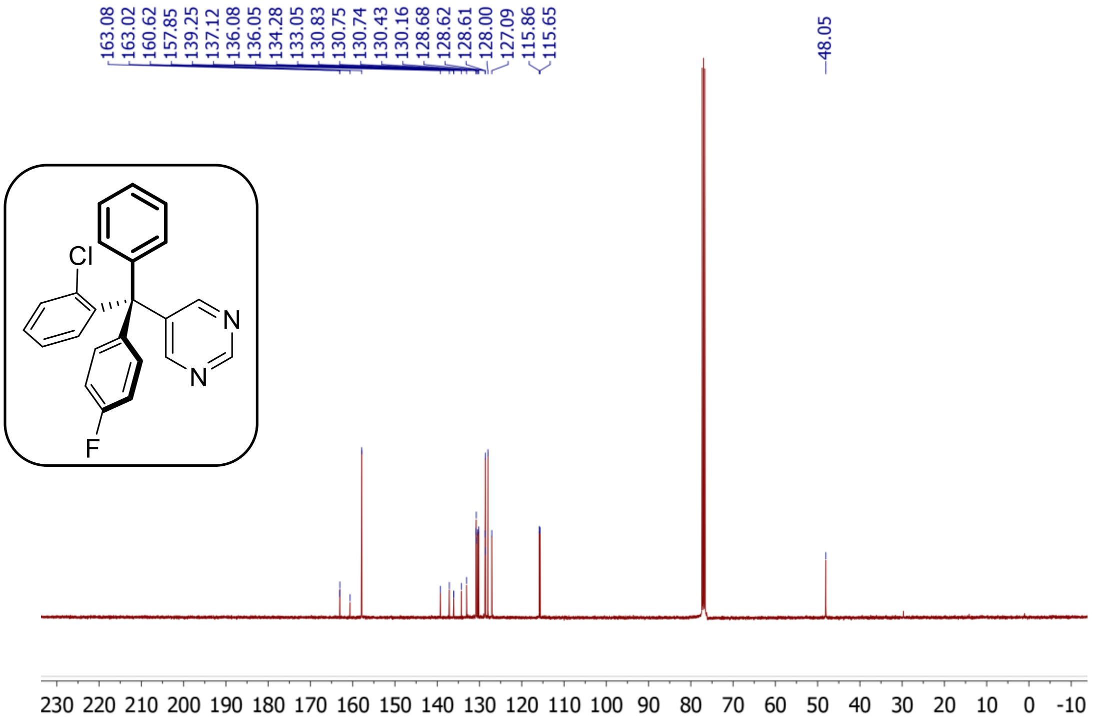

Supplementary Fig. 130. ^{19}**F NMR (376 MHz, CDCl$_3$) spectrum of 5-((2-chlorophenyl)(4-fluorophenyl)(phenyl)methyl)pyrimidine (72).**

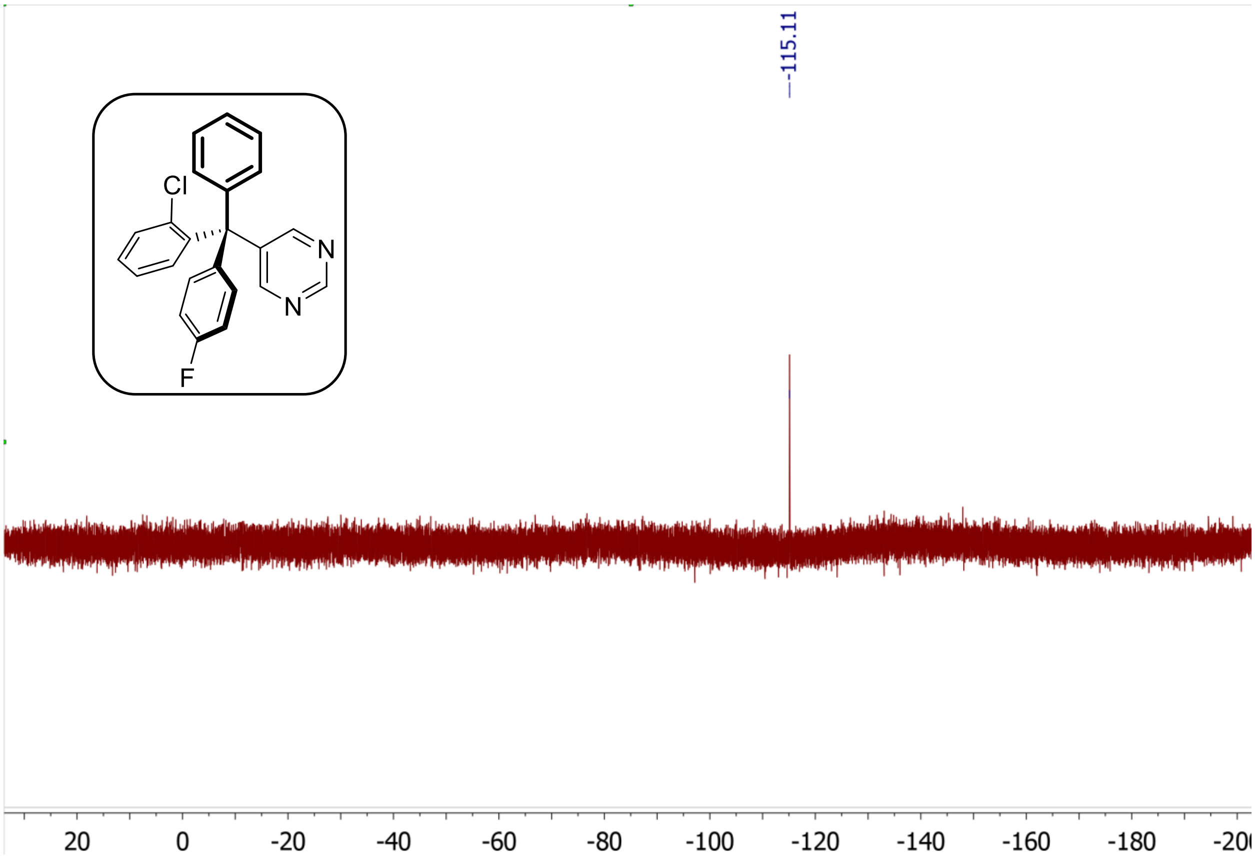

Supplementary Fig. 131. ^{1}H NMR (400 MHz, $CDCl_3$) spectrum of 2-((1-benzylpiperidin-4-yl)methyl)-5,6-dimethoxy-2-phenyl-2,3-dihydro-1H-inden-1-one (73).

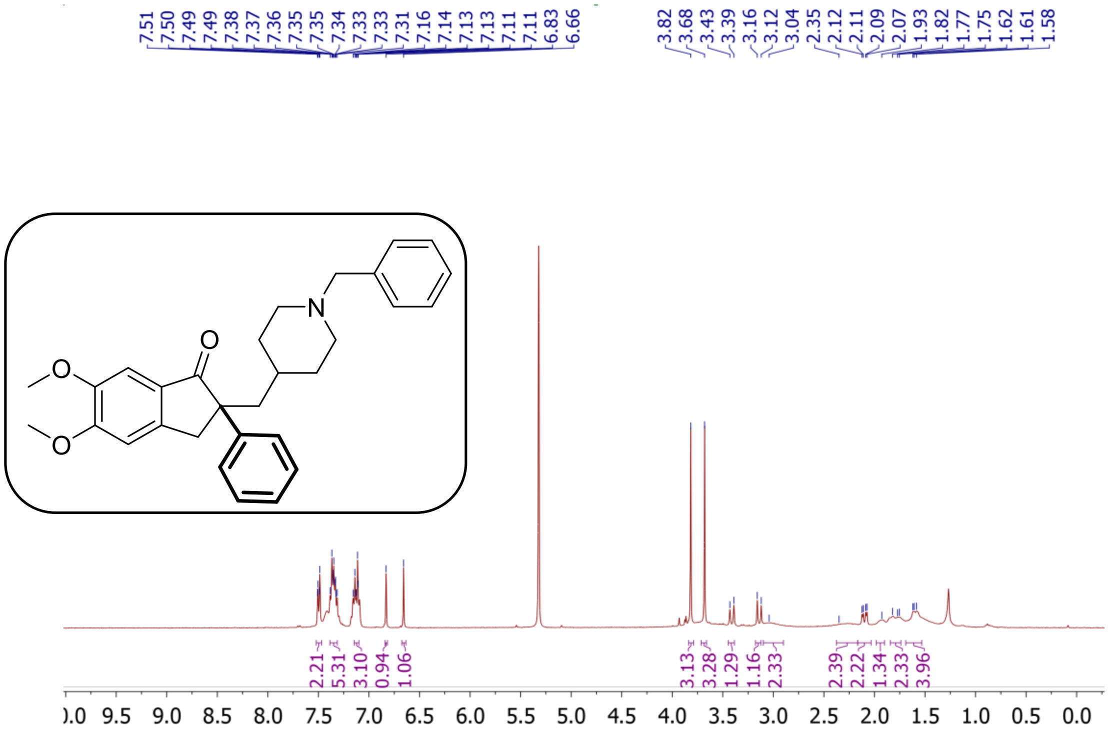

Supplementary Fig. 132. ^{13}C NMR (100 MHz, $CDCl_3$) spectrum of 2-((1-benzylpiperidin-4-yl)methyl)-5,6-dimethoxy-2-phenyl-2,3-dihydro-1H-inden-1-one (73).

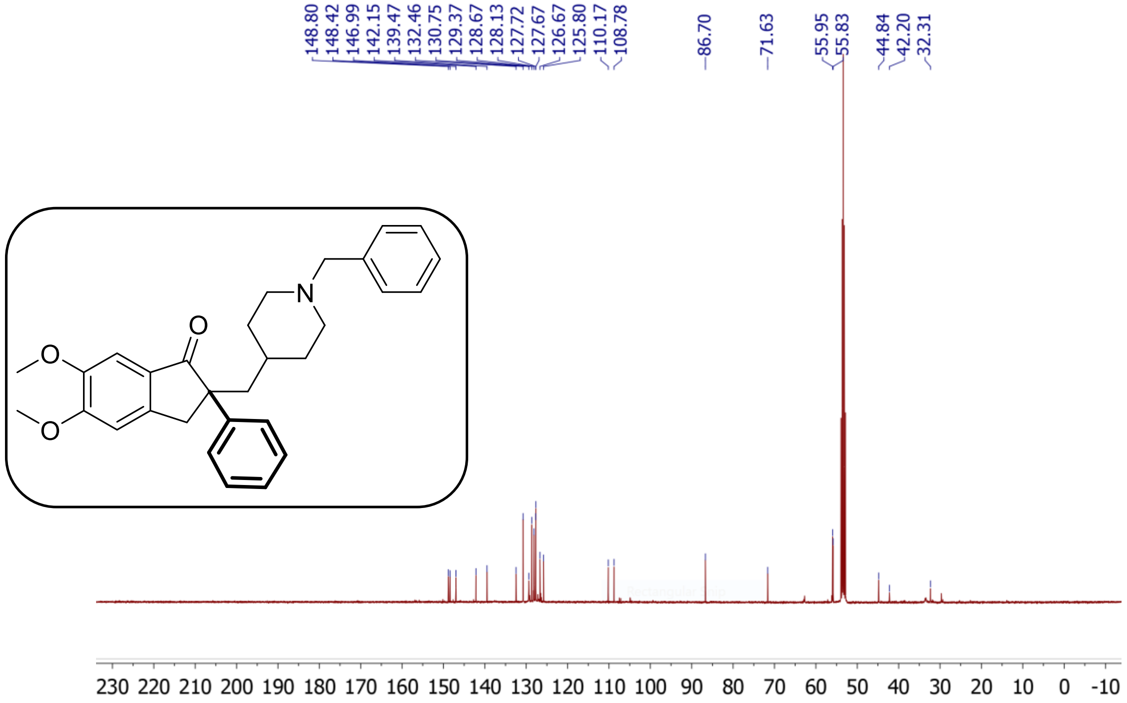

Supplementary Fig. 133. ^{1}H NMR (400 MHz, CDCl$_3$) spectrum of 4-ethyl-4-phenyl-1,12-dihydro-14H-pyrano[3',4':6,7]indolizino[1,2-b]quinoline-3,14(4H)-dione (47).

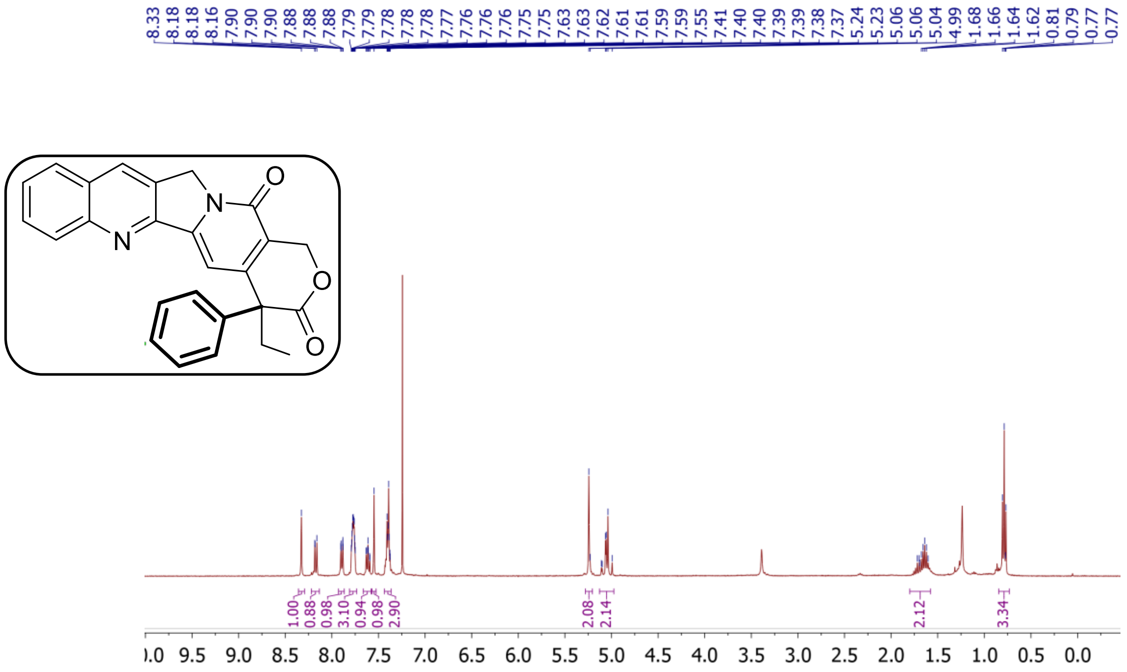

Supplementary Fig. 134. ^{13}C NMR (100 MHz, CDCl$_3$) spectrum of 4-ethyl-4-phenyl-1,12-dihydro-14H-pyrano[3',4':6,7]indolizino[1,2-b]quinoline-3,14(4H)-dione (47).

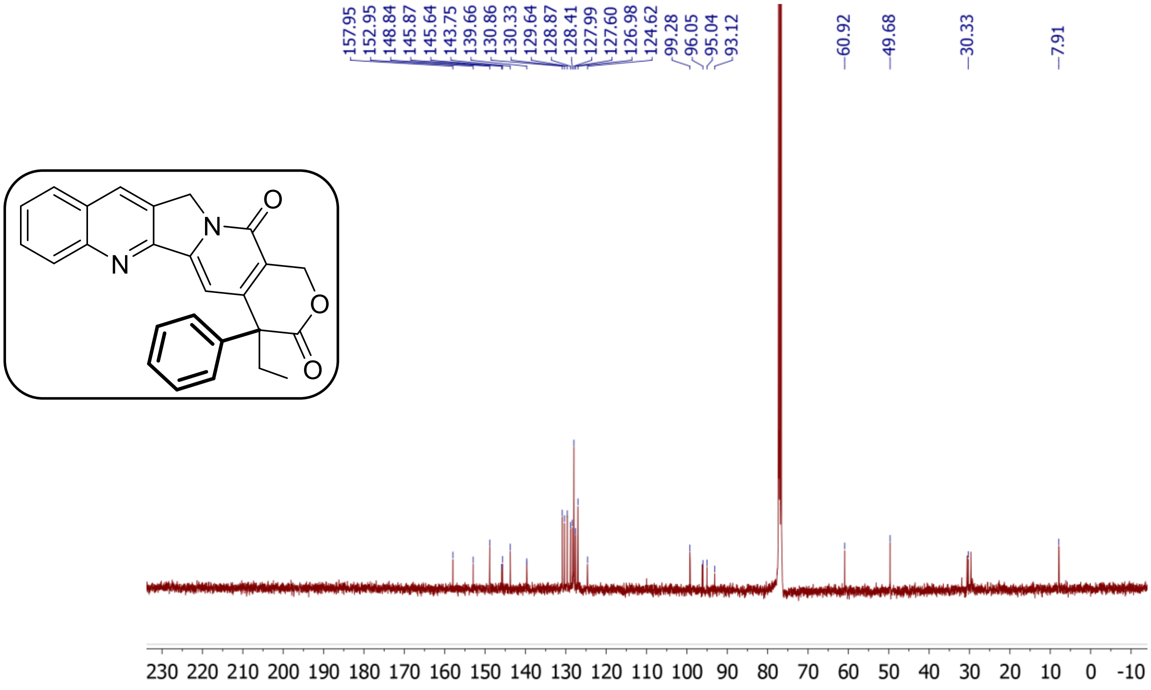

Supplementary Fig. 135. **^{1}H NMR (400 MHz, $CDCl_3$) spectrum of (1S,2S,4S,5R)-2-((S)-(4-chlorophenyl)(6-methoxyquinolin-4-yl)methyl)-5-vinylquinuclidine (74).**

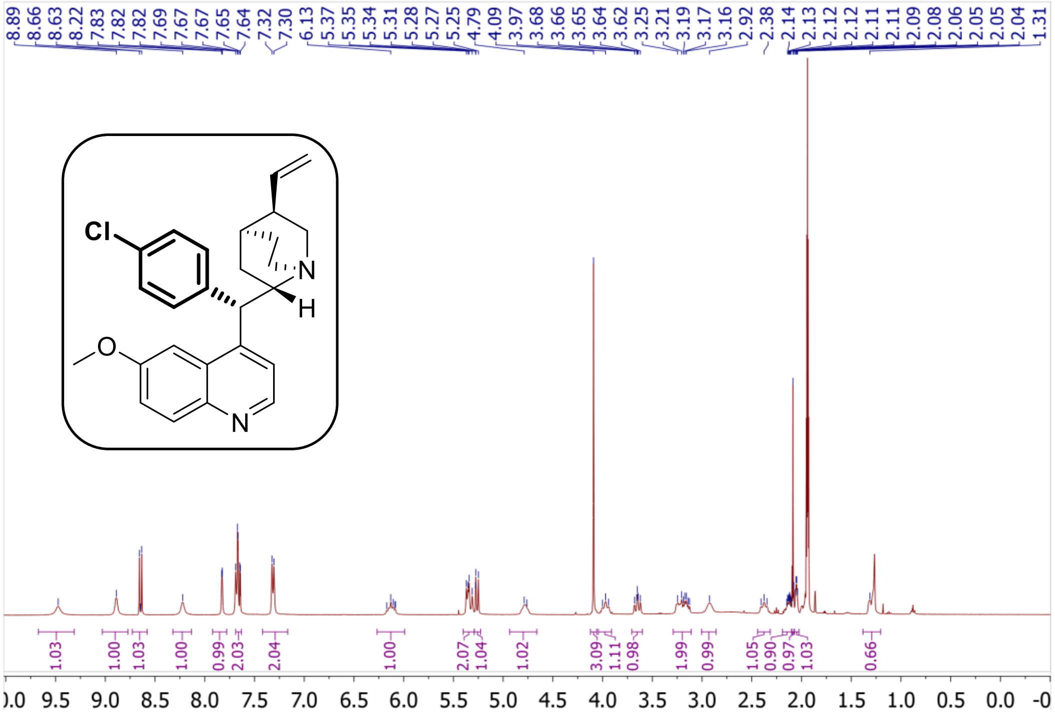

Supplementary Fig. 136. **^{13}C NMR (100 MHz, $CDCl_3$) spectrum of (1S,2S,4S,5R)-2-((S)-(4-chlorophenyl)(6-methoxyquinolin-4-yl)methyl)-5-vinylquinuclidine (74).**

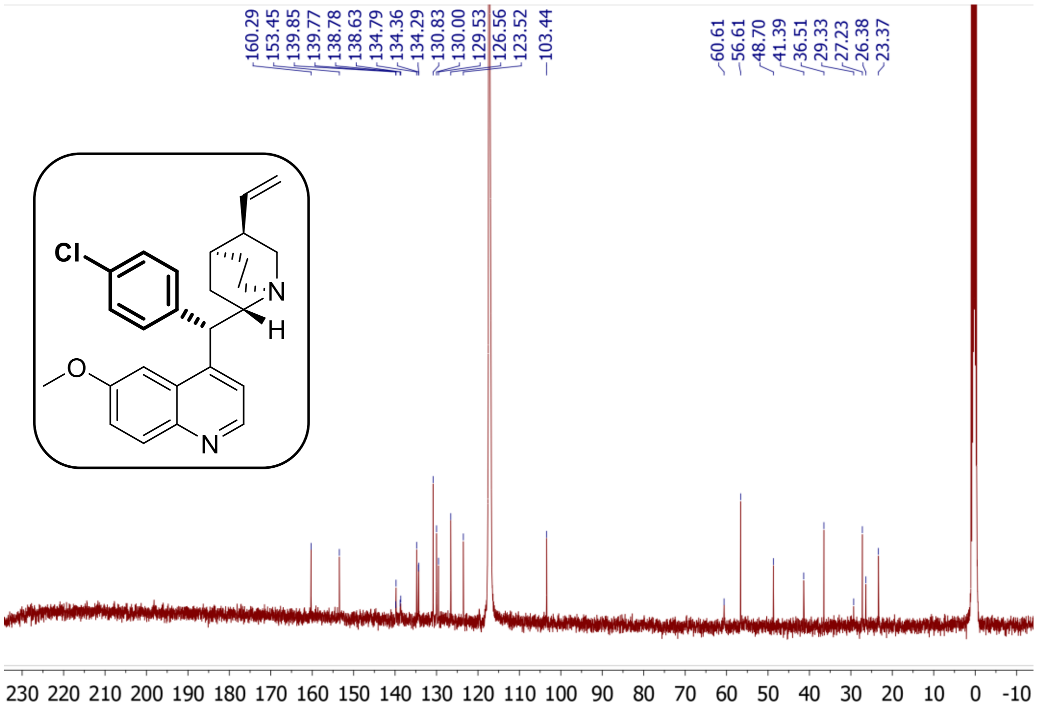

Supplementary Fig. 137. ^{1}**H NMR (400 MHz, ACN-d$_3$) spectrum of dimesitylzinc.**

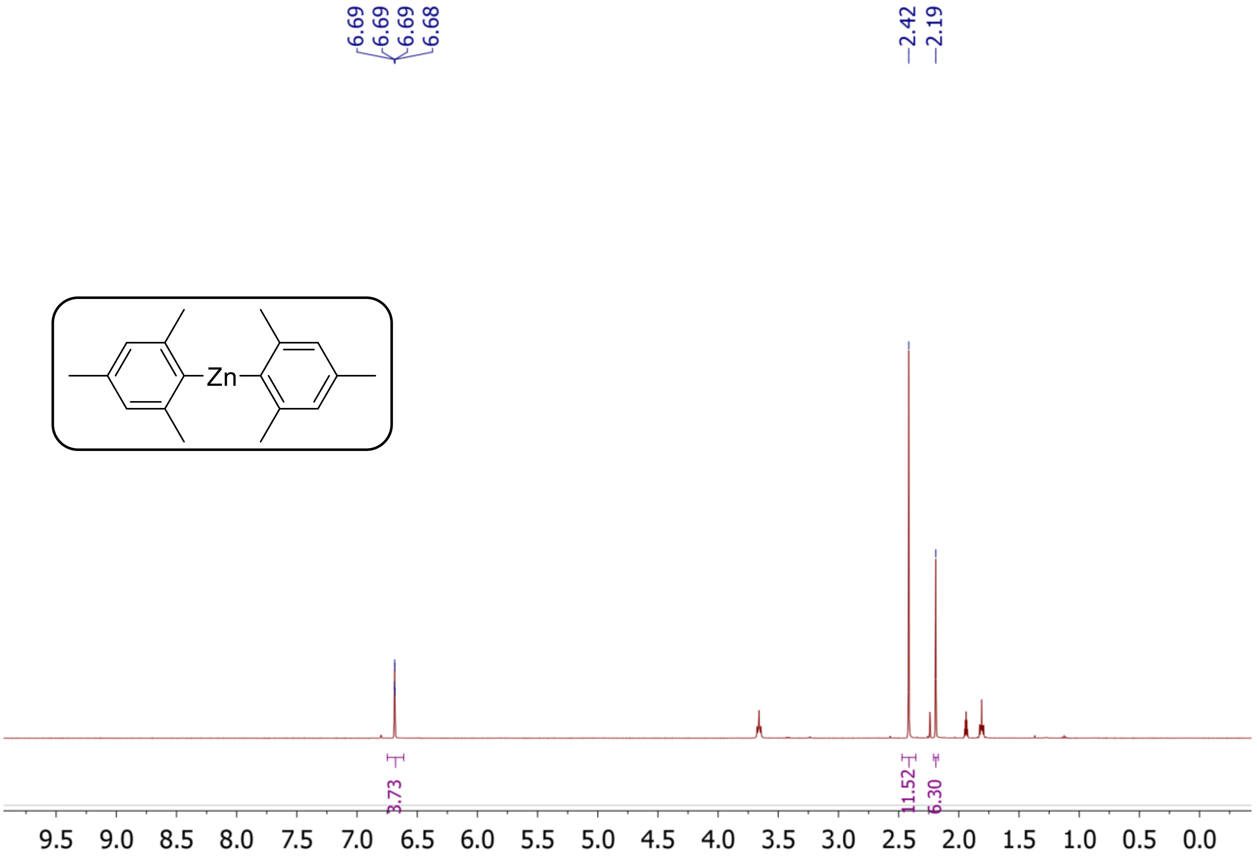

Supplementary Fig. 138. ^{1}**H NMR (400 MHz, ACN-d$_3$) spectrum of bis(4-chlorophenyl)zinc • 2 THF.**

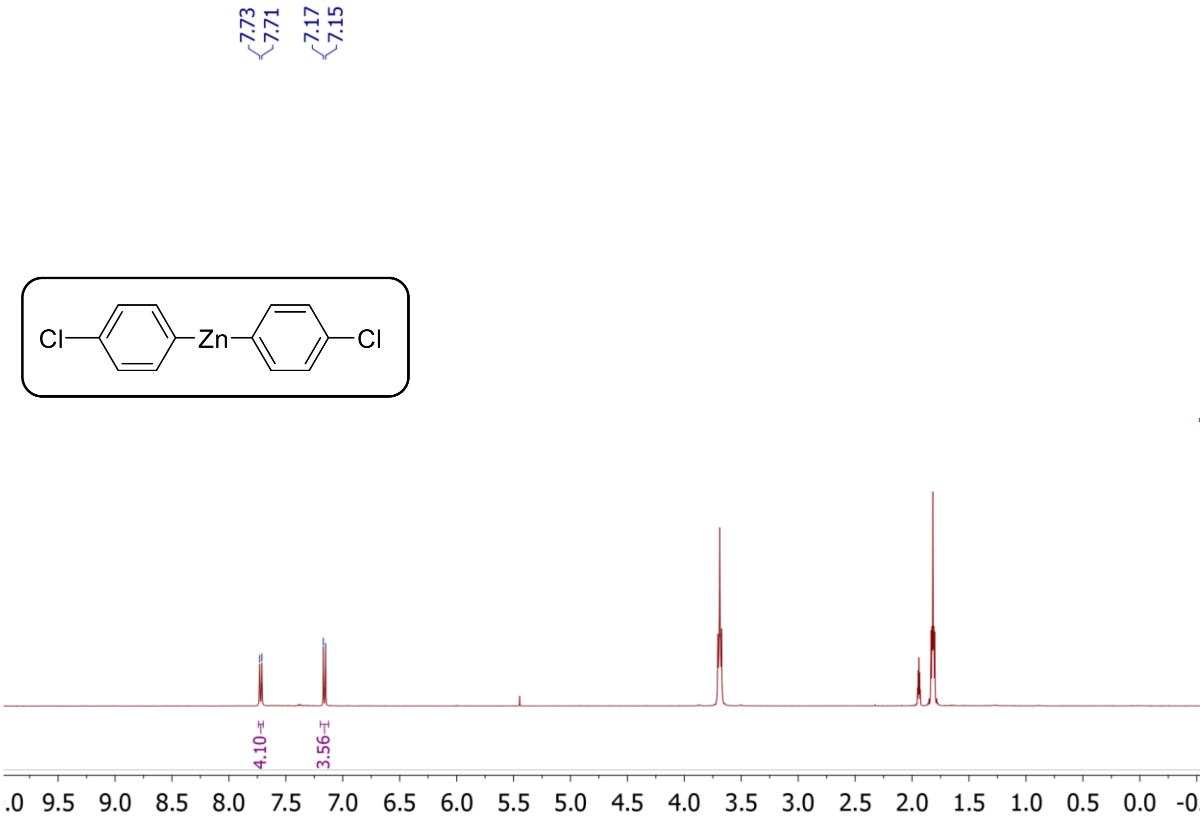

Supplementary Fig. 139. ¹H NMR (400 MHz, ACN-d3) spectrum of bis(3-methylbenzo[b]thiophen-2-yl)zinc•THF.

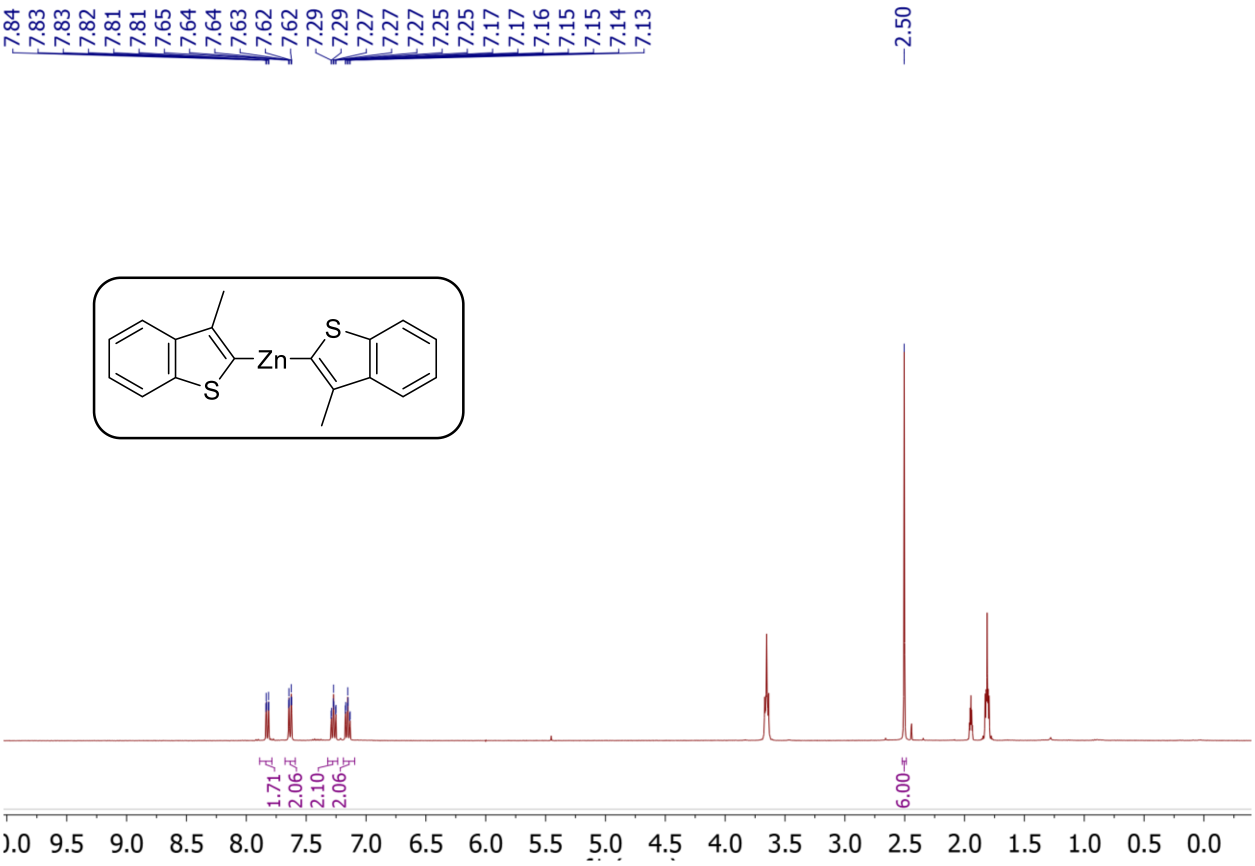

Supplementary Fig. 140. **^{1}H NMR (400 MHz, ACN-d$_3$) spectrum of bis(3,4-dimethylthiophen-2-yl)zinc • 2 THF.**

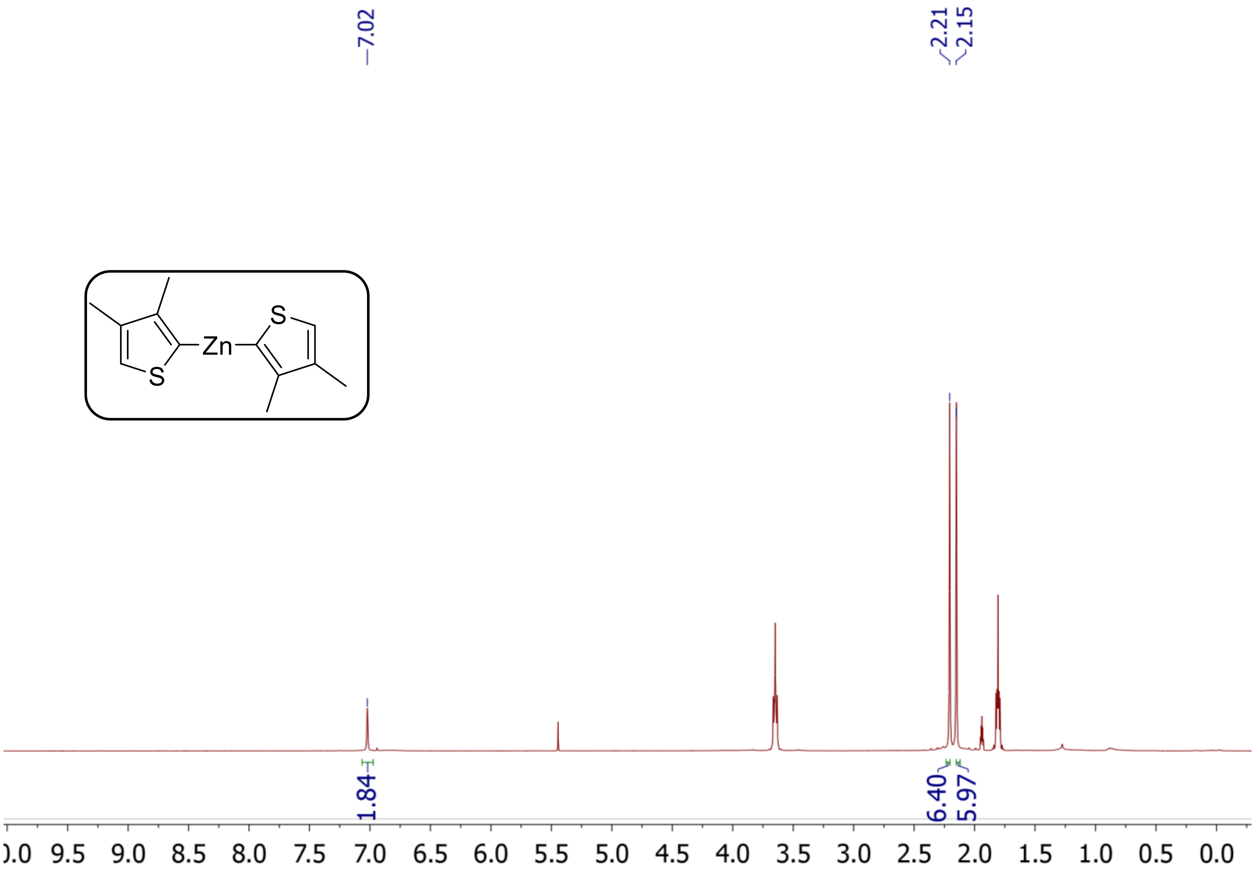

Supplementary Fig. 141. ^{1}**H NMR (400 MHz, ACN-d**$_3$**) spectrum of diferrocenylzinc.**

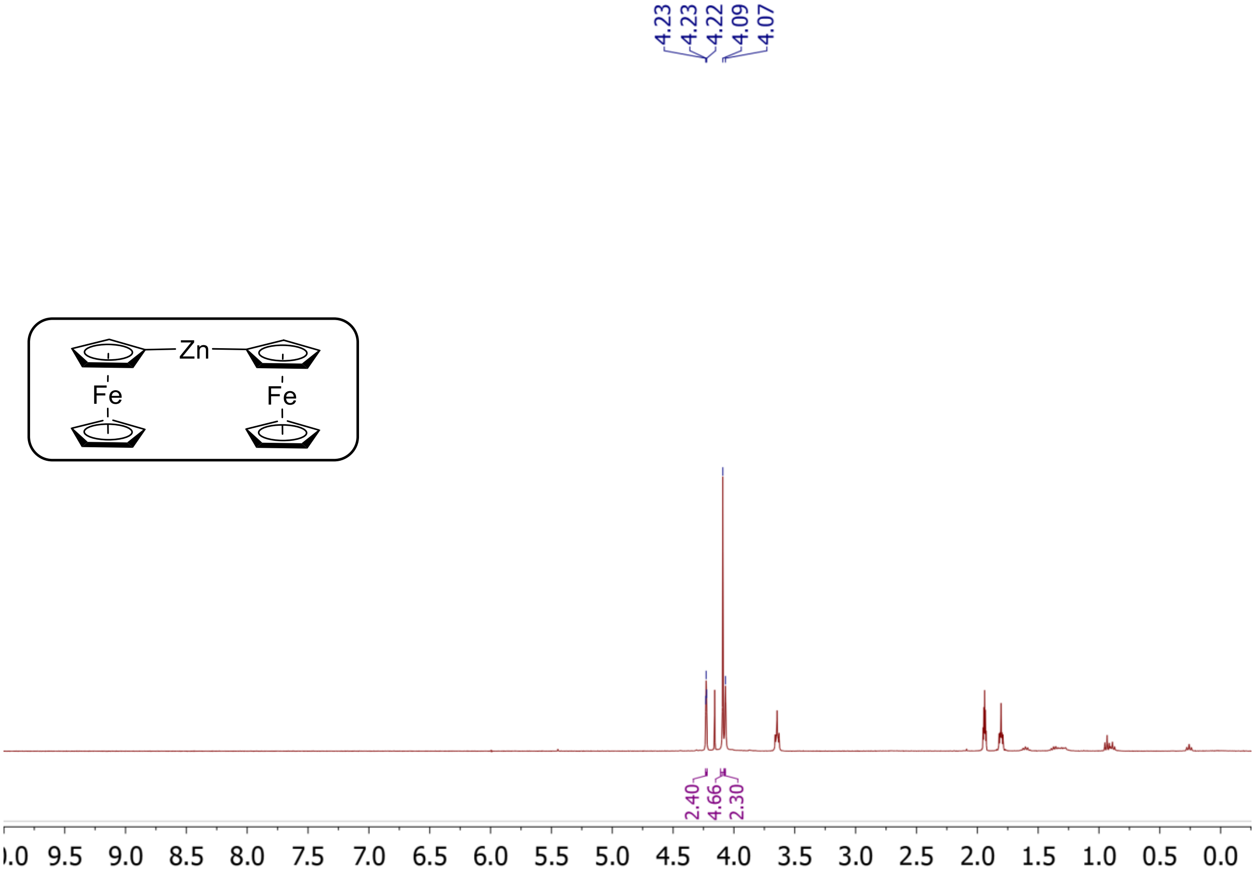

Supplementary Fig. 142. ^{1}**H NMR (400 MHz, ACN-d$_3$) spectrum of bis(3-methylbenzofuran-2-yl)zinc • THF.**

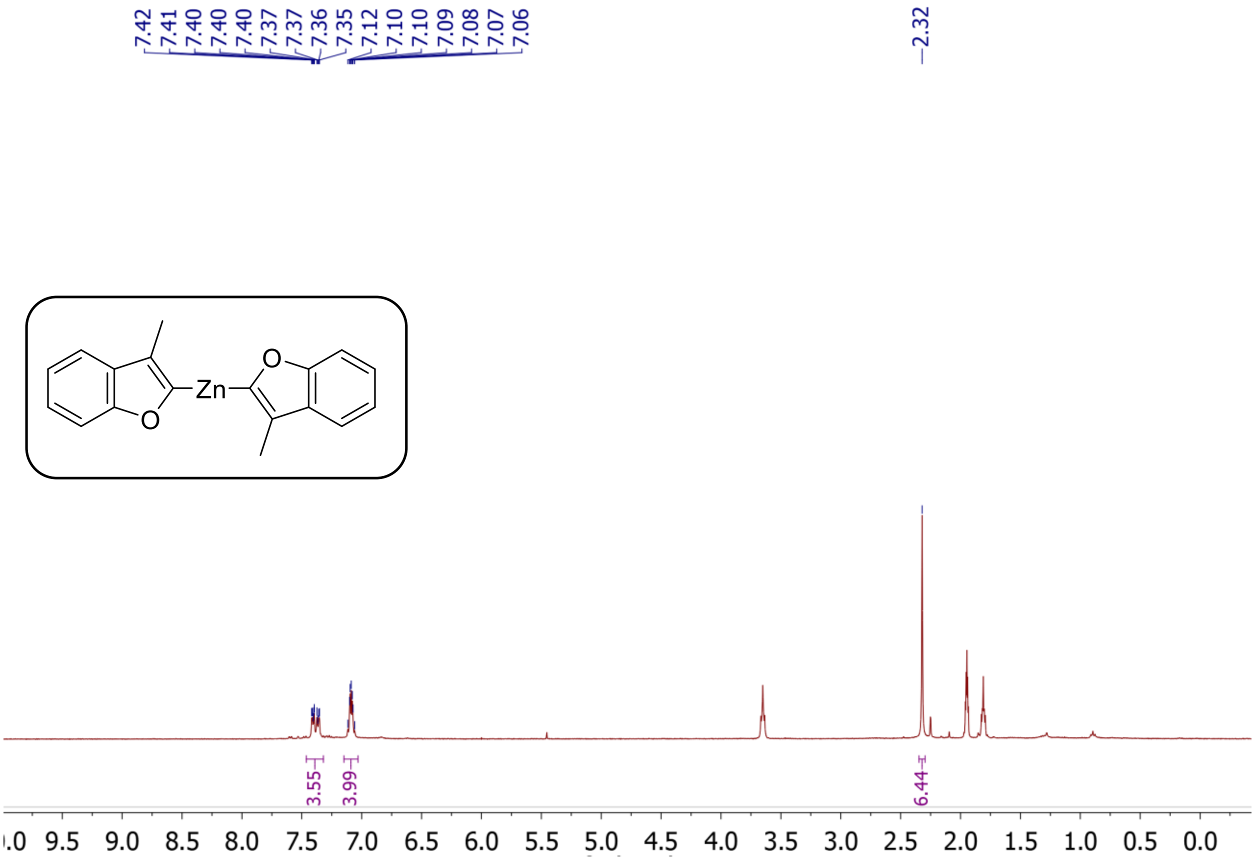

Supplementary Fig. 143. ^{1}H NMR spectrum (400 MHz, $CDCl_3$) of triphenylfluoromethane.

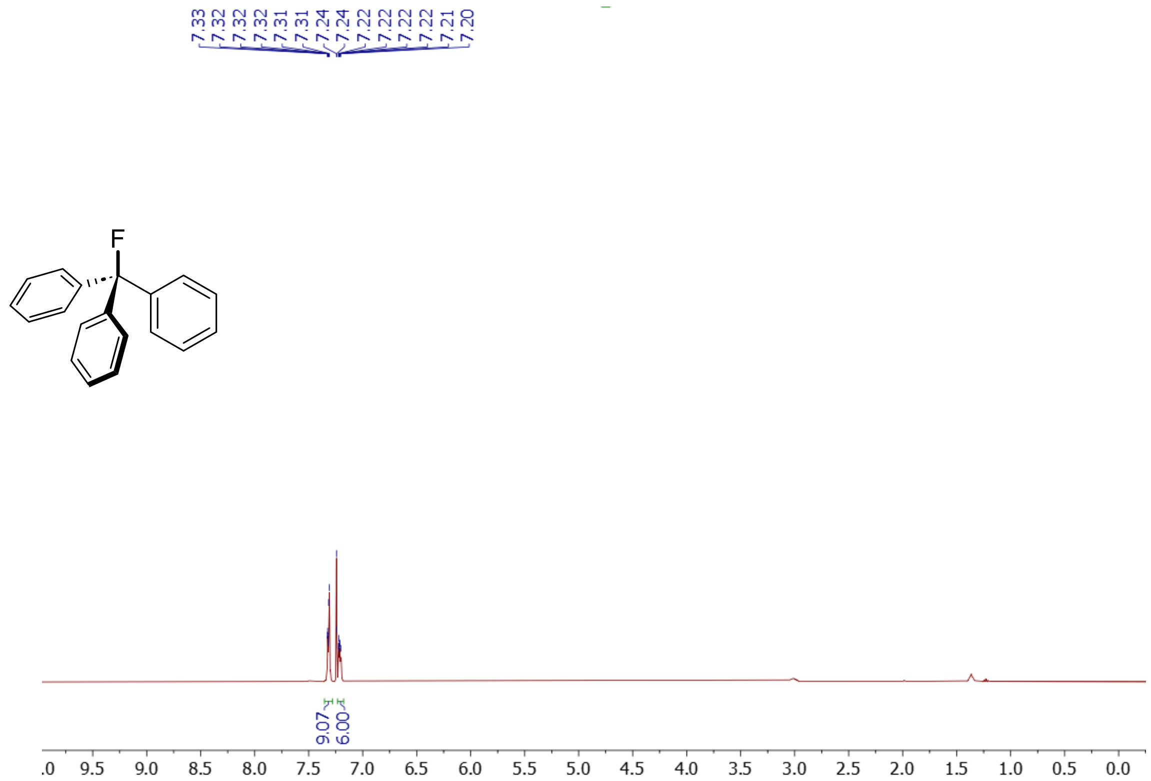

Supplementary Fig. 144. ^{19}F NMR spectrum (376 MHz, $CDCl_3$) of triphenylfluoromethane.

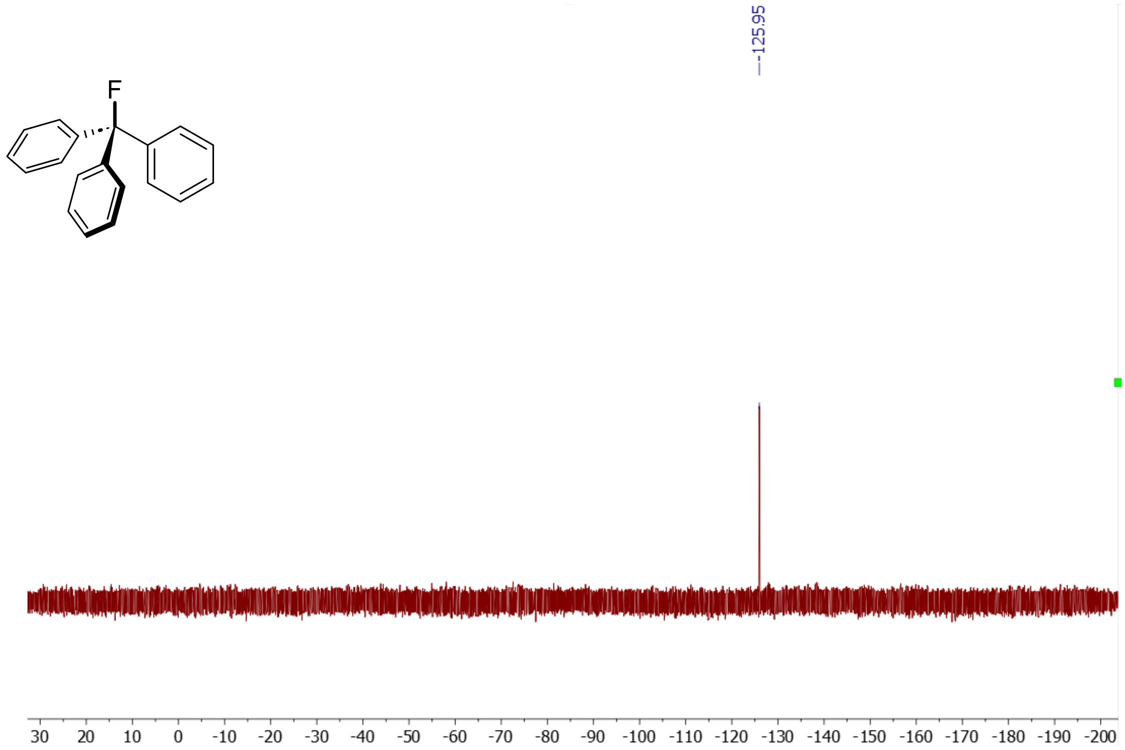

Supplementary Fig. 145. ^{1}H NMR spectrum (400 MHz, $CDCl_3$) of 3-fluoro-3-methyl-1-phenylindolin-2-one.

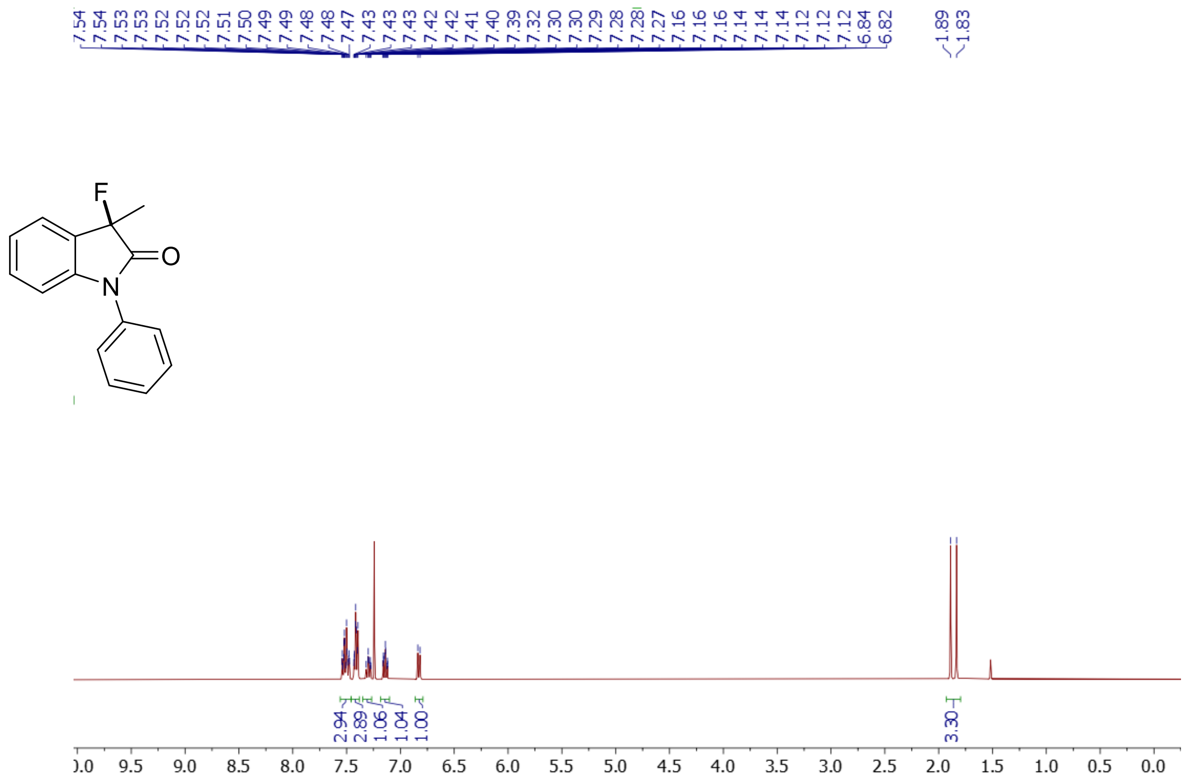

Supplementary Fig. 146. ^{19}F NMR spectrum (376 MHz, $CDCl_3$) of 3-fluoro-3-methyl-1-phenylindolin-2-one.

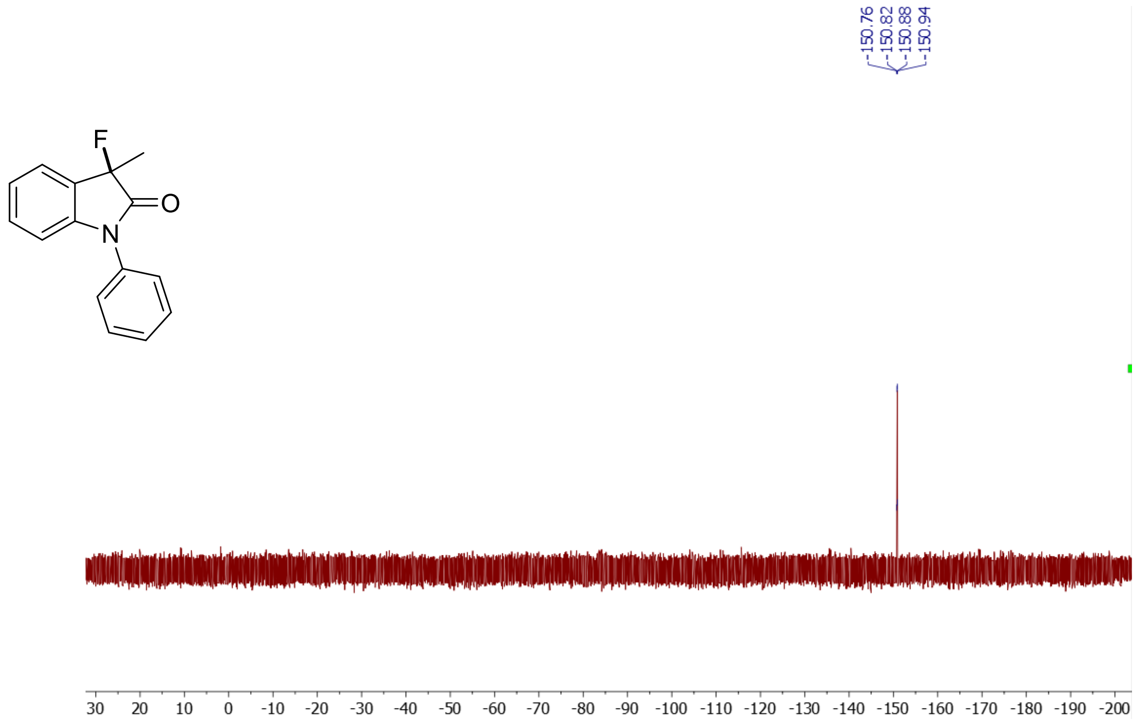

Supplementary Fig. 147. ^{1}H NMR spectrum (400 MHz, $CDCl_3$) of (3-fluorobutyl)benzene.

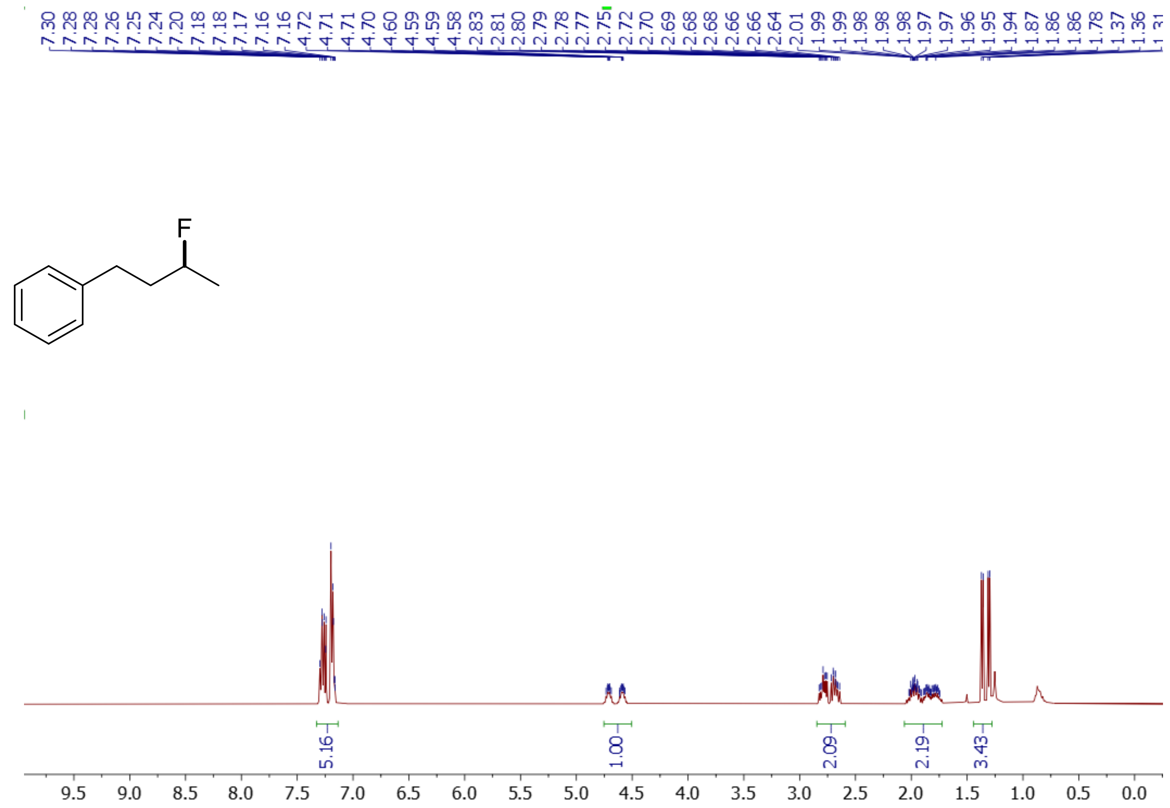

Supplementary Fig. 148. ^{19}F NMR spectrum (376 MHz, $CDCl_3$) of (3-fluorobutyl)benzene.

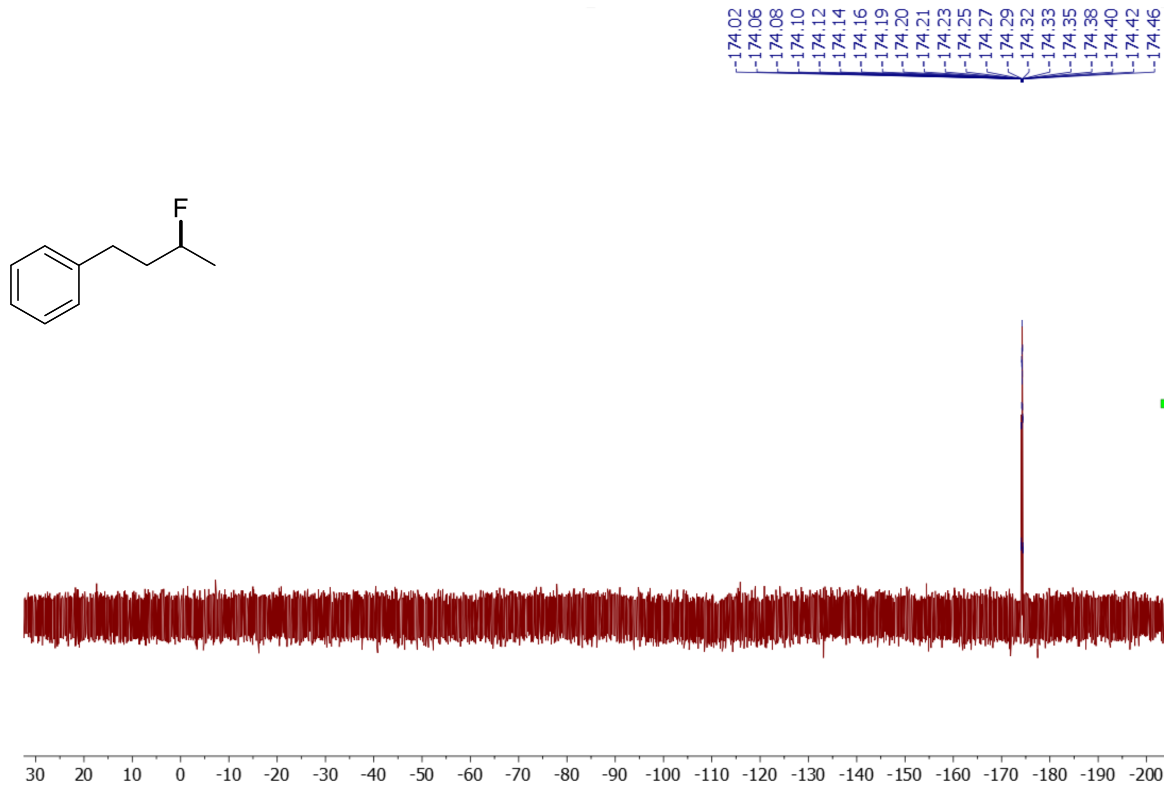

Supplementary Fig. 149. ^{1}H NMR spectrum (400 MHz, $CDCl_3$) of 1-fluorodec-2-yne.

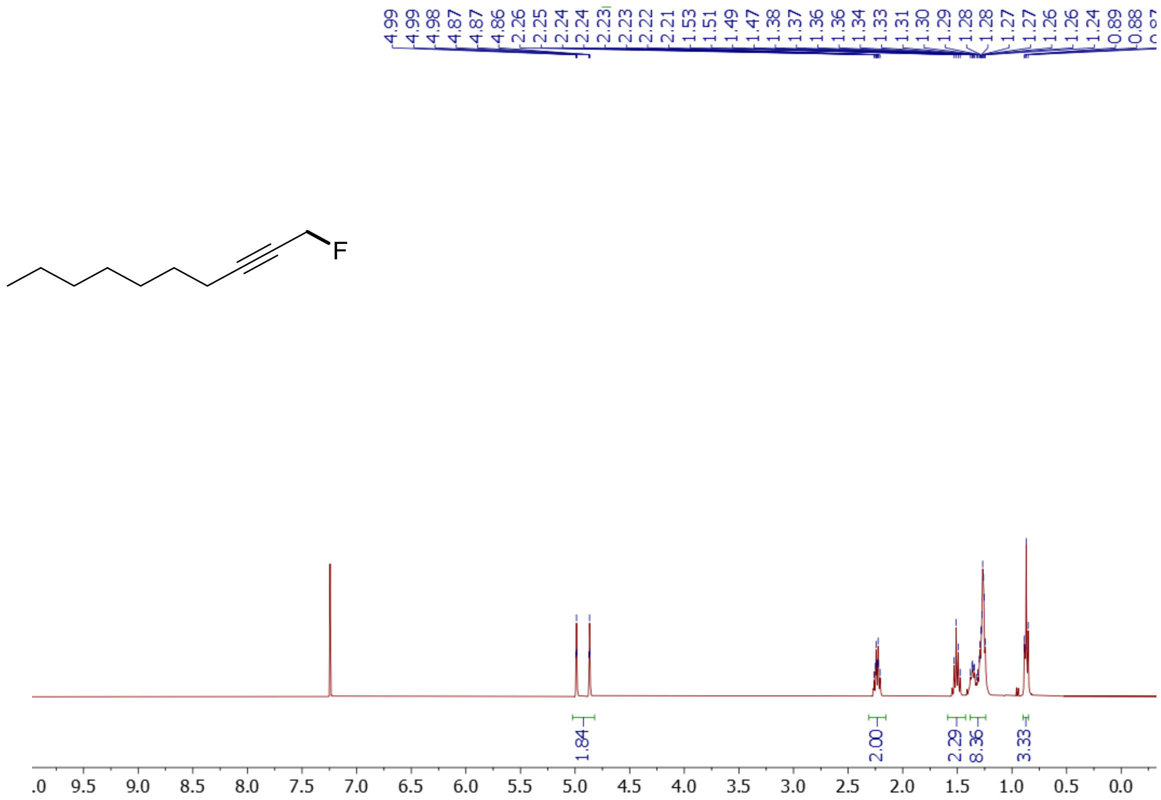

Supplementary Fig. 150. ^{19}F NMR spectrum (376 MHz, $CDCl_3$) of 1-fluorodec-2-yne.

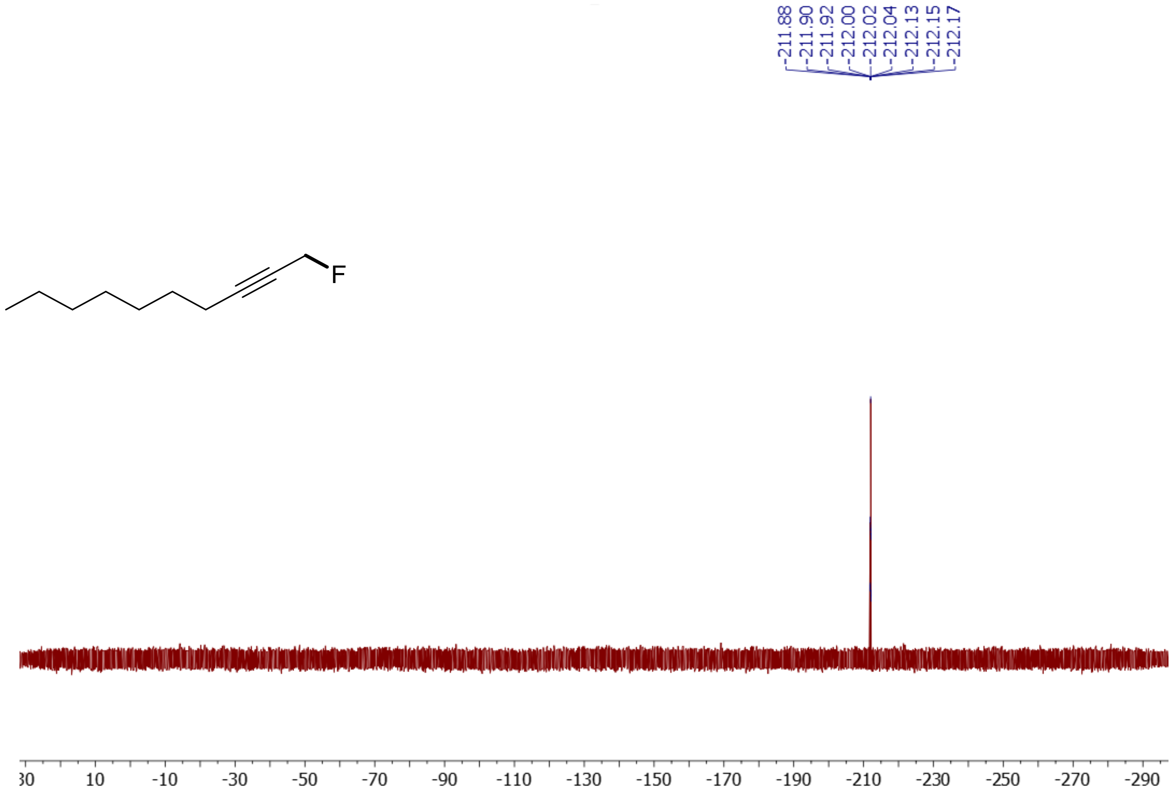

Supplementary Fig. 151. ^{1}H NMR spectrum (400 MHz, $CDCl_3$) of 1-(fluoromethyl)-4-nitrobenzene.

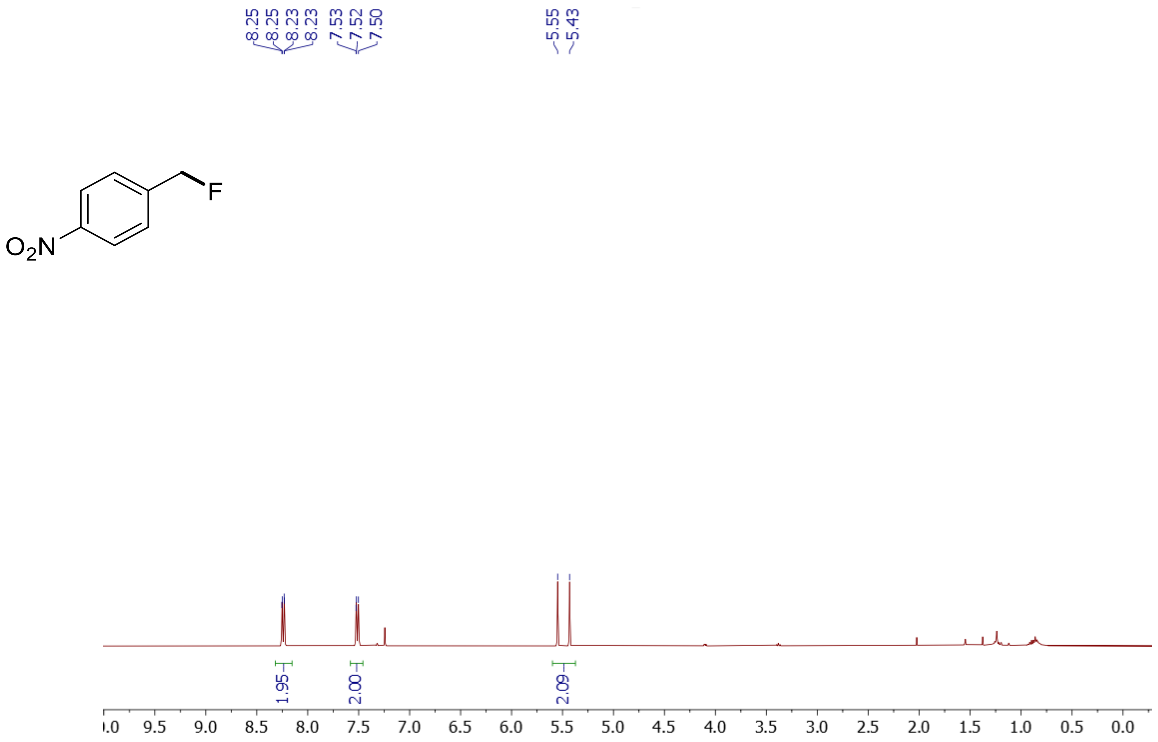

Supplementary Fig. 152. ^{19}F NMR spectrum (376 MHz, $CDCl_3$) of 1-(fluoromethyl)-4-nitrobenzene.

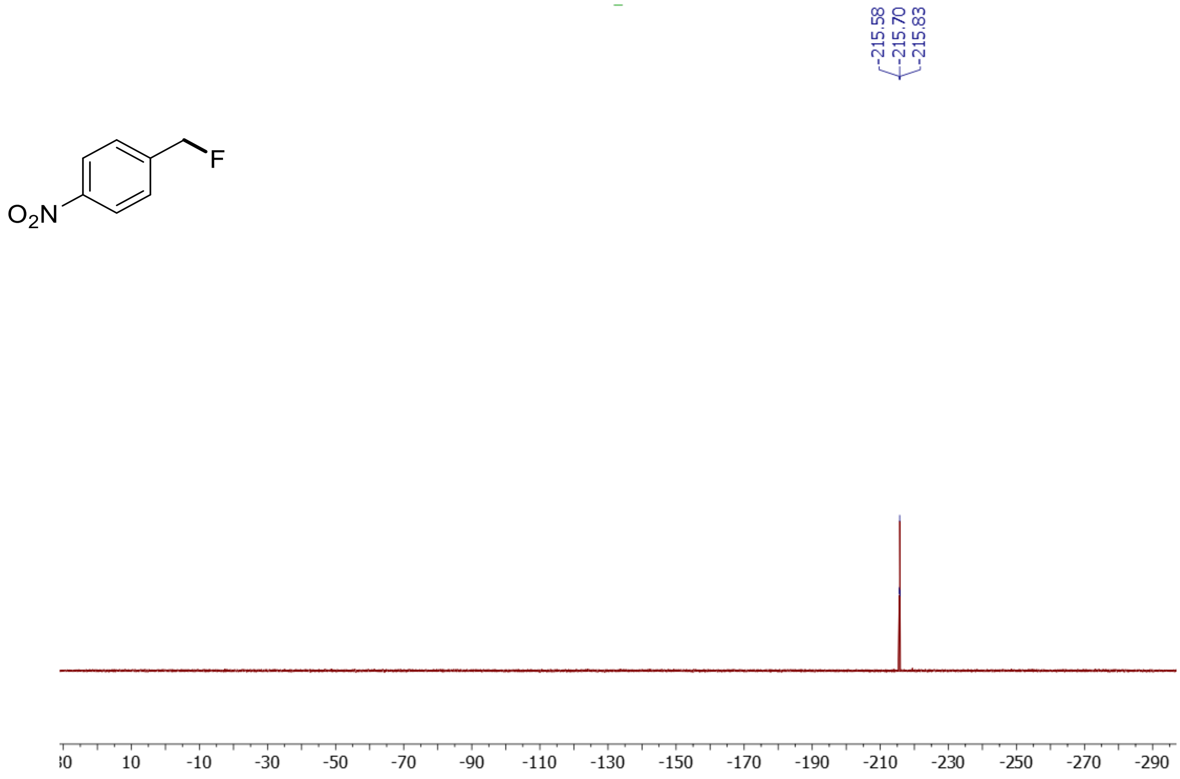

Supplementary Fig. 153. **^{1}H NMR spectrum (400 MHz, $CDCl_3$) of 1-(3-fluorobutyl)-4-methoxybenzene.**

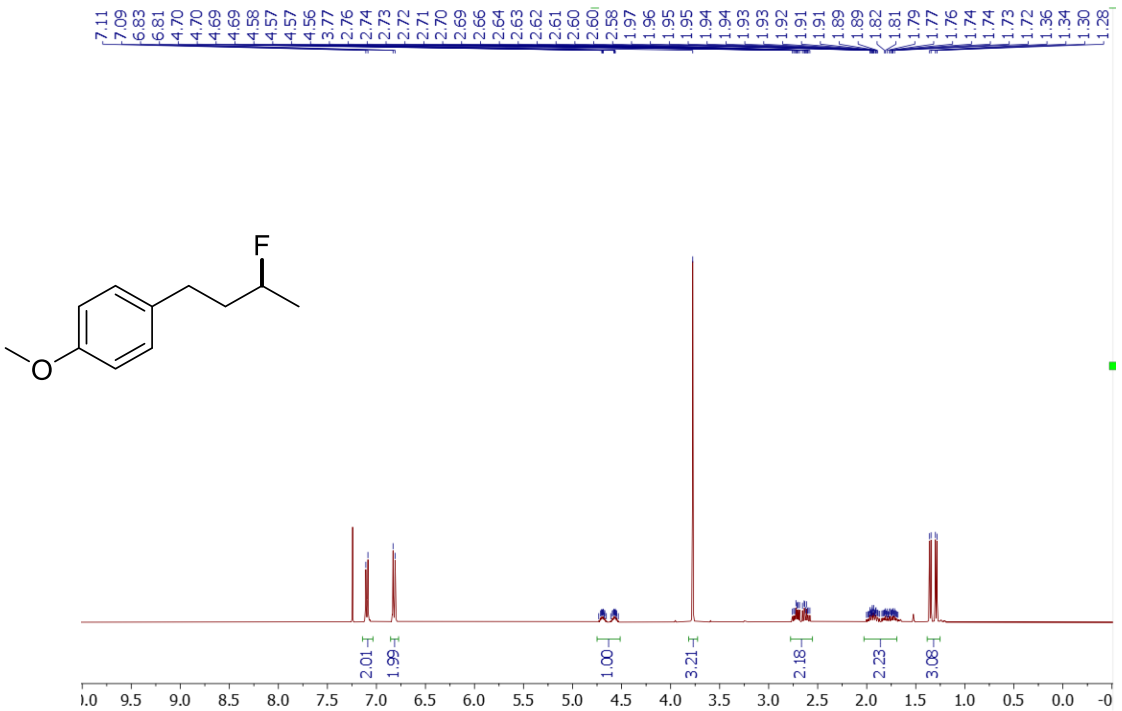

Supplementary Fig. 154. **^{19}F NMR spectrum (376 MHz, $CDCl_3$) of 1-(3-fluorobutyl)-4-methoxybenzene.**

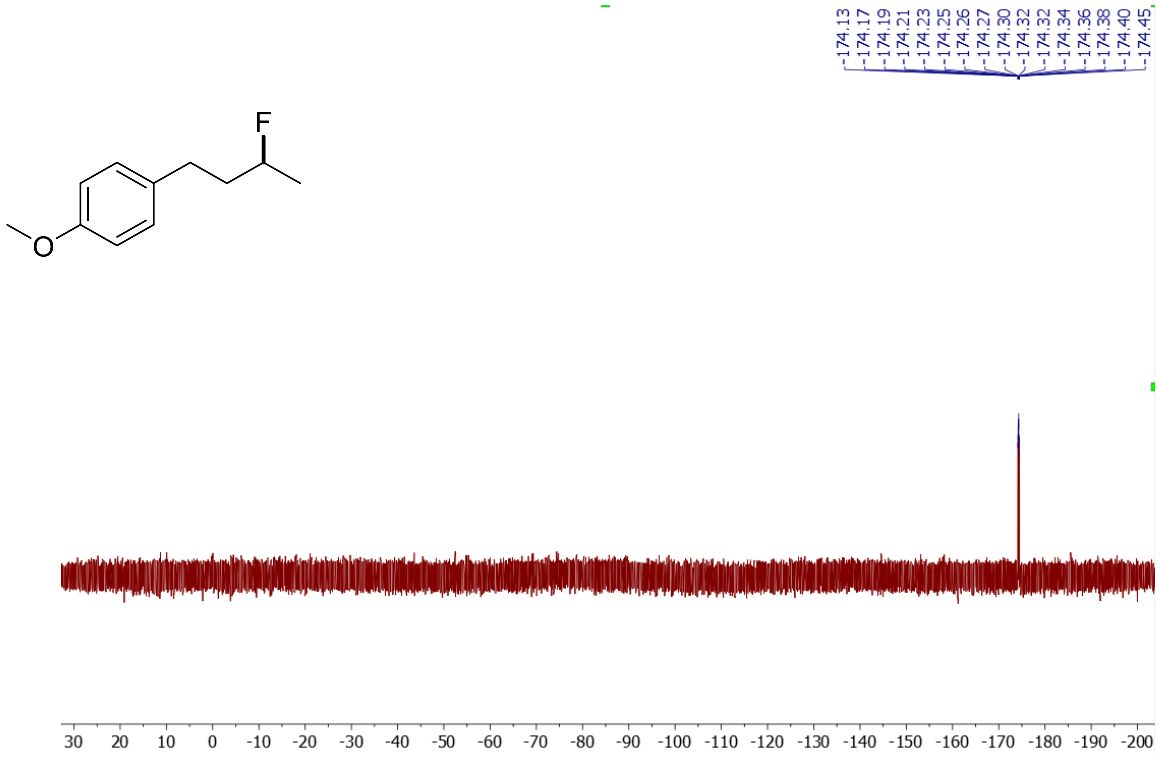

Supplementary Fig. 155. ^{1}**H NMR spectrum (400 MHz, CDCl**$_3$**) of 1-(1-fluoroethyl)-3-nitrobenzene.**

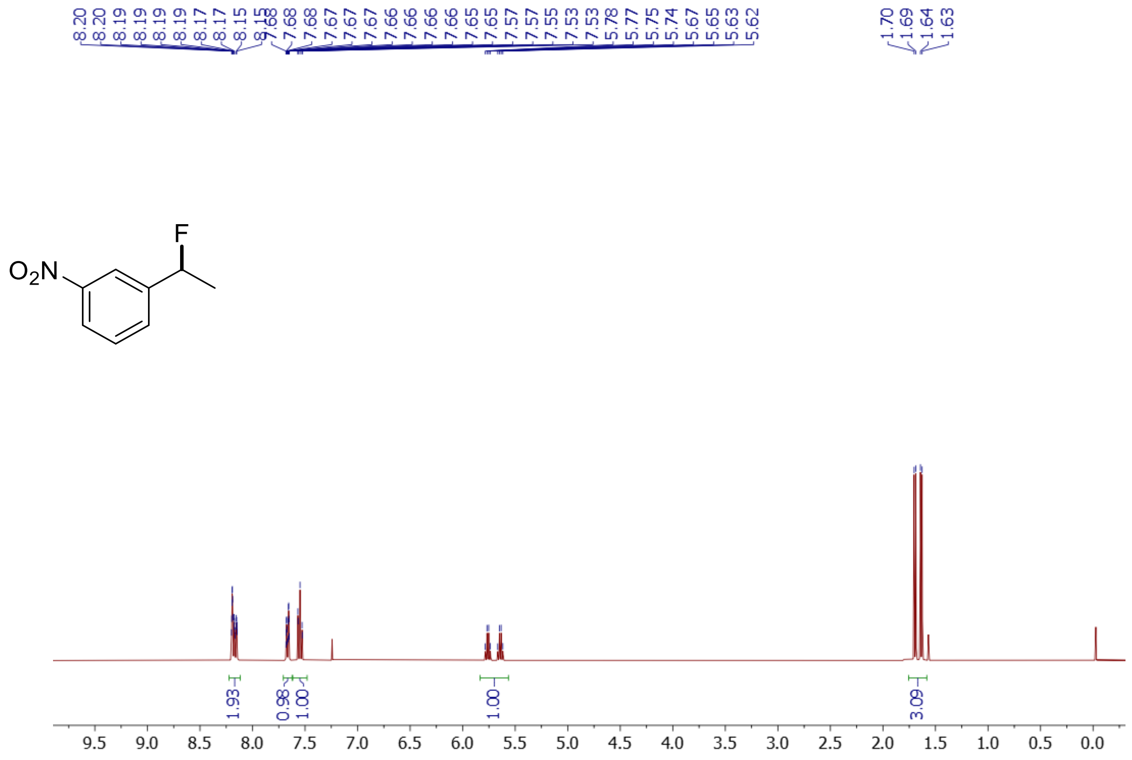

Supplementary Fig. 156. ^{19}**F NMR spectrum (376 MHz, CDCl**$_3$**) of 1-(1-fluoroethyl)-3-nitrobenzene.**

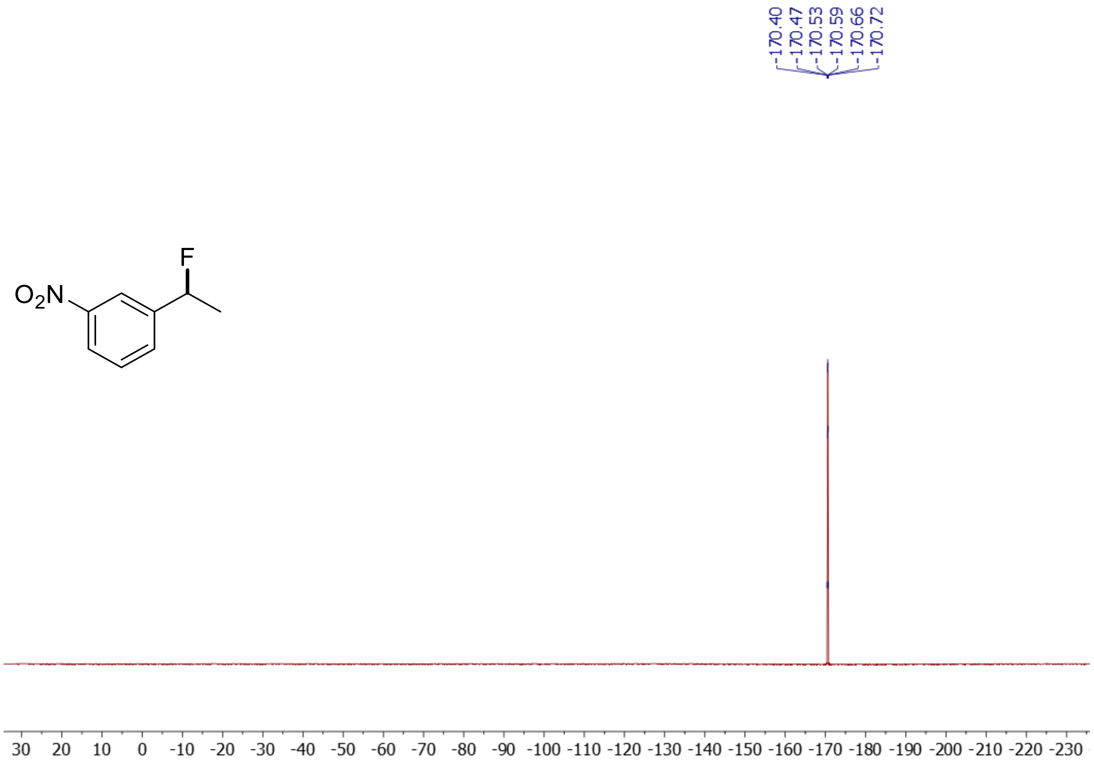

Supplementary Fig. 157. ^{1}H NMR spectrum (400 MHz, $CDCl_3$) of (cyclohexylfluoromethyl)benzene.

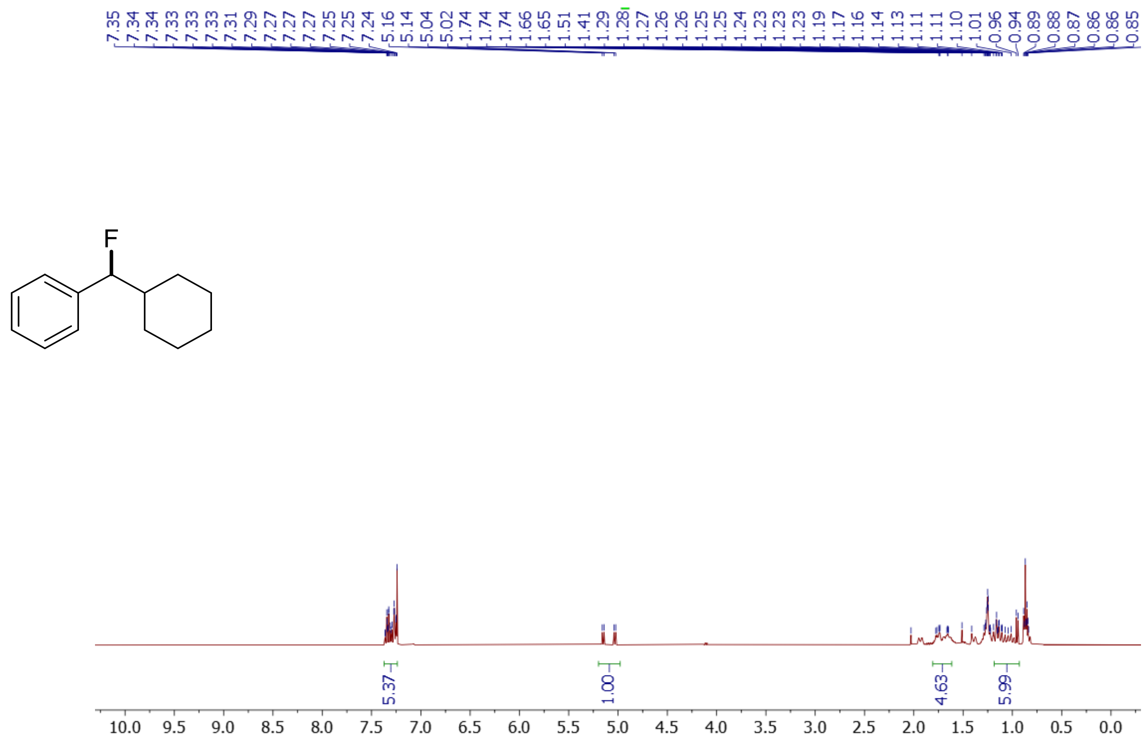

Supplementary Fig. 158. ^{19}F NMR spectrum (376 MHz, $CDCl_3$) of (cyclohexylfluoromethyl)benzene.

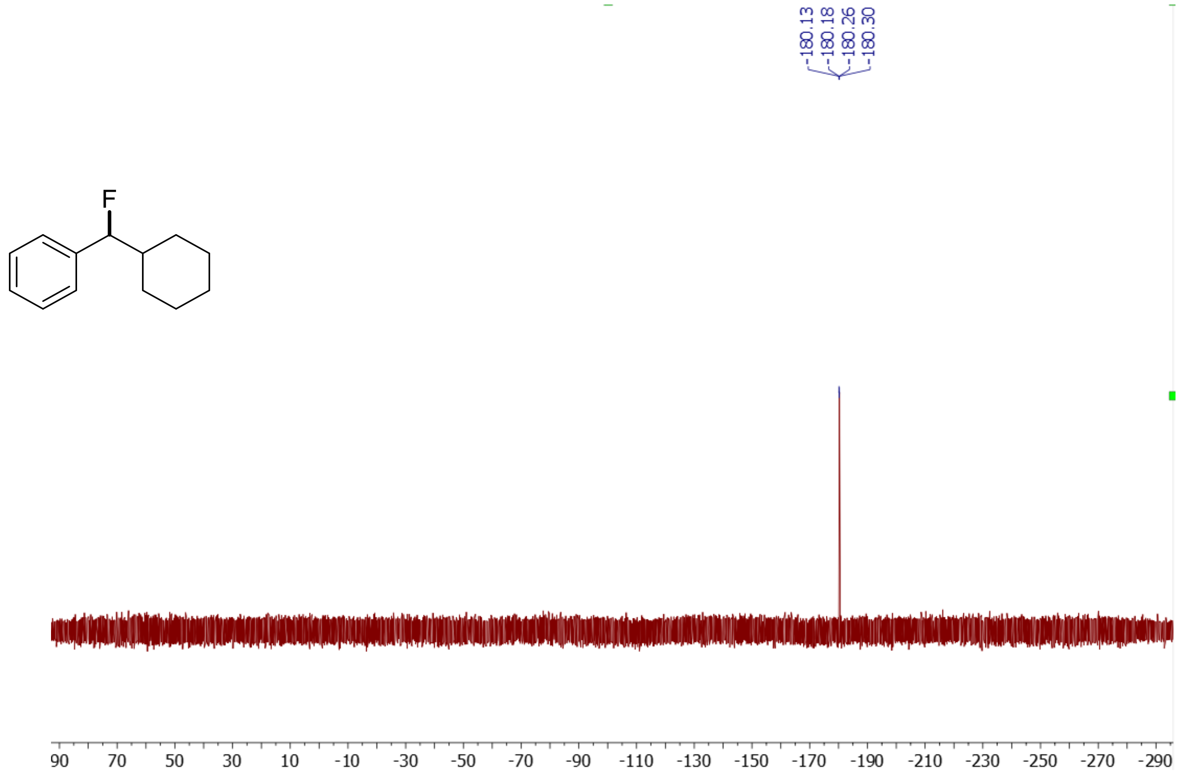

Supplementary Fig. 159. ^{1}**H NMR spectrum (400 MHz, CDCl**$_3$**) of (3-fluorobut-1-yn-1-yl)benzene.**

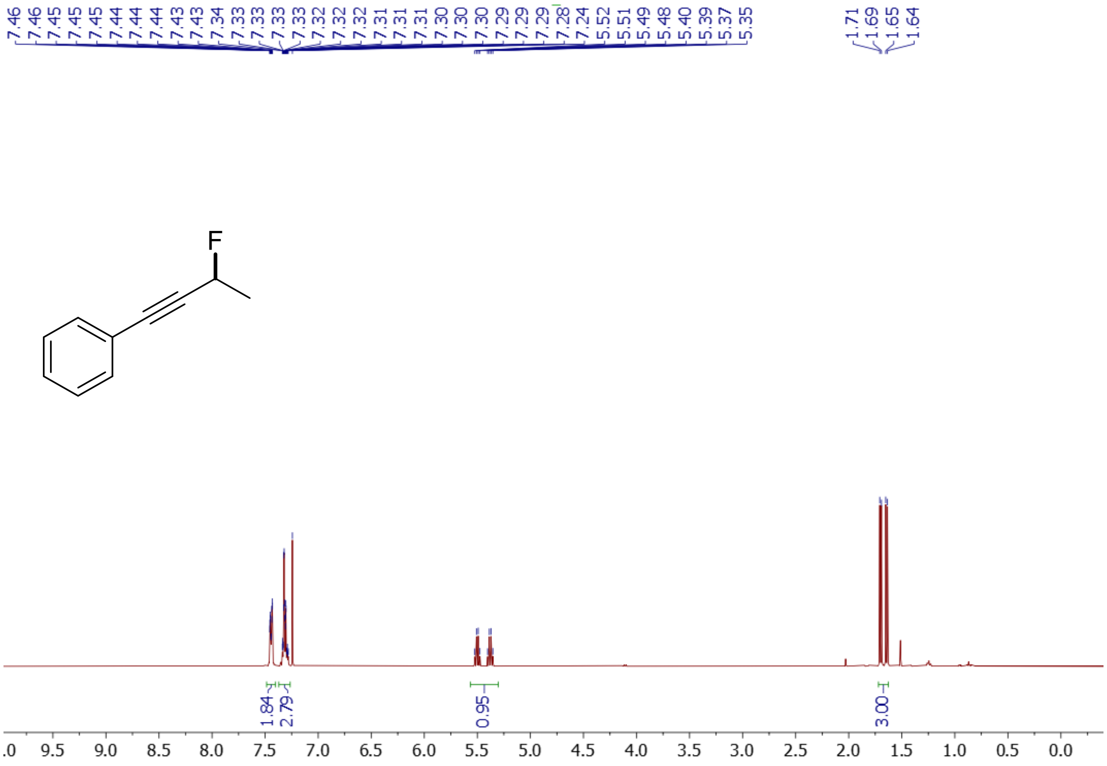

Supplementary Fig. 160. ^{19}**F NMR spectrum (376 MHz, CDCl**$_3$**) of (3-fluorobut-1-yn-1-yl)benzene.**

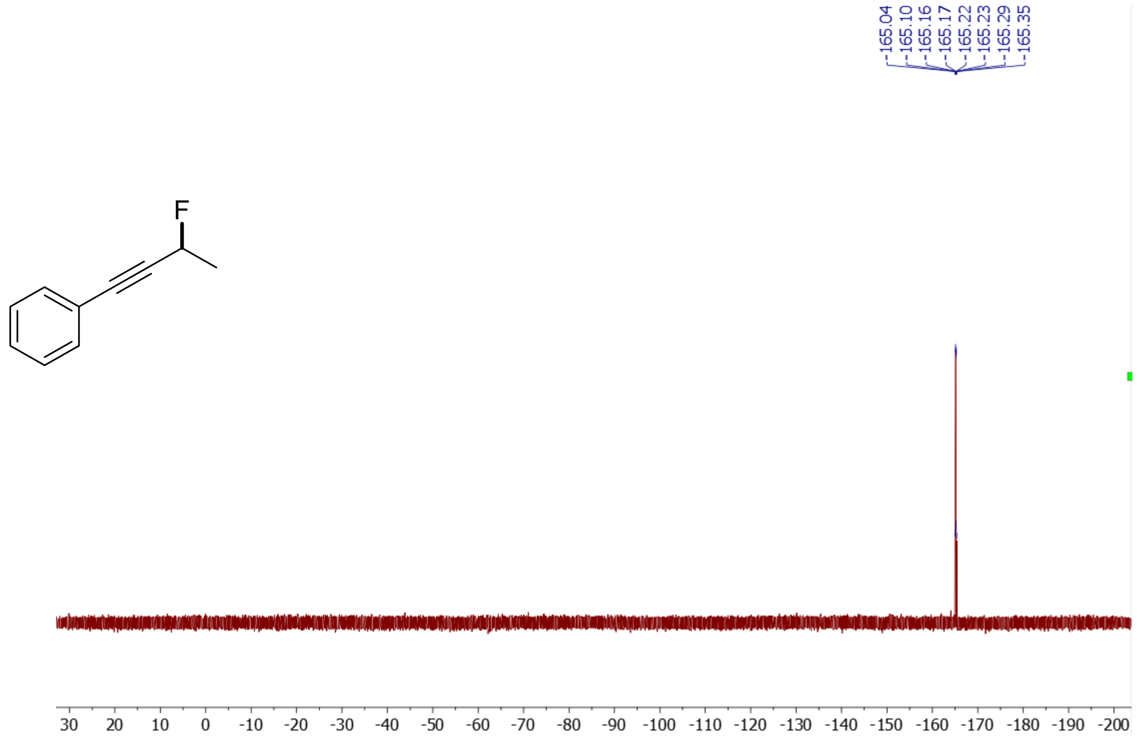

Supplementary Fig. 161. ^{1}H NMR spectrum (400 MHz, $CDCl_3$) of 4-(fluoro(*o*-tolyl)methyl)benzonitrile.

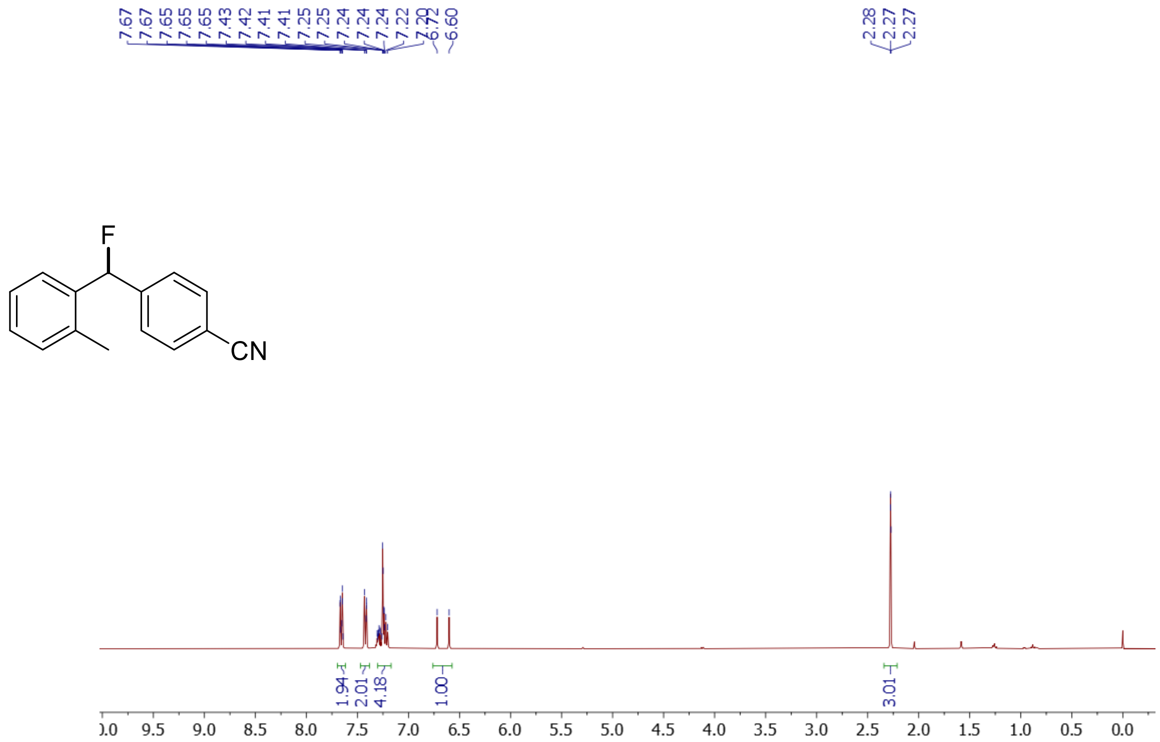

Supplementary Fig. 162. ^{19}F NMR spectrum (376 MHz, $CDCl_3$) of 4-(fluoro(*o*-tolyl)methyl)benzonitrile.

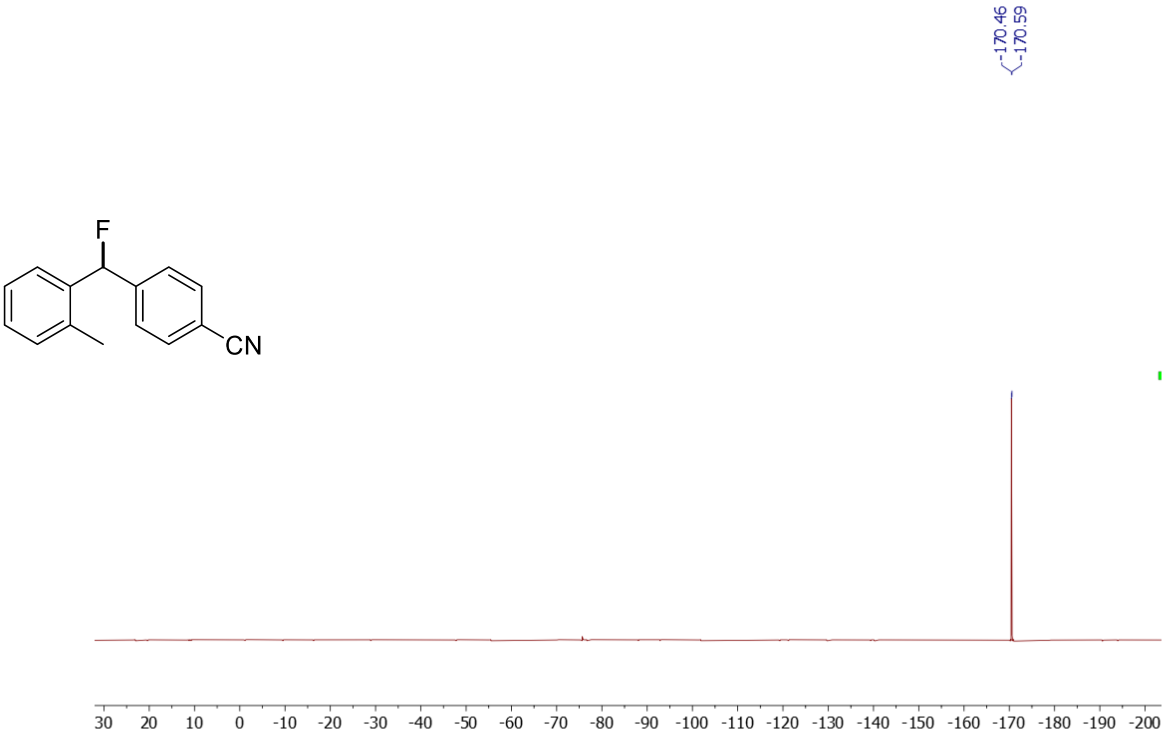

Supplementary Fig. 163. ^{1}H NMR spectrum (400 MHz, $CDCl_3$) of 2-fluoro-2-methyl-1-phenylpropan-1-one.

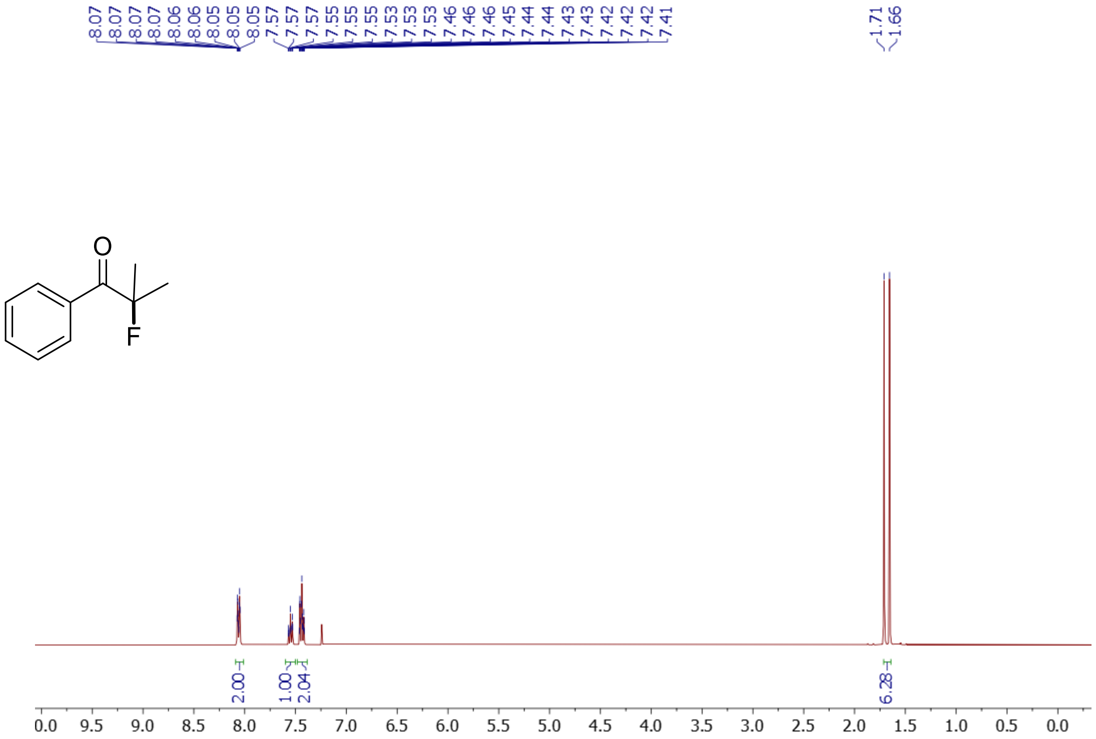

Supplementary Fig. 164. ^{19}F NMR spectrum (376 MHz, $CDCl_3$) of 2-fluoro-2-methyl-1-phenylpropan-1-one.

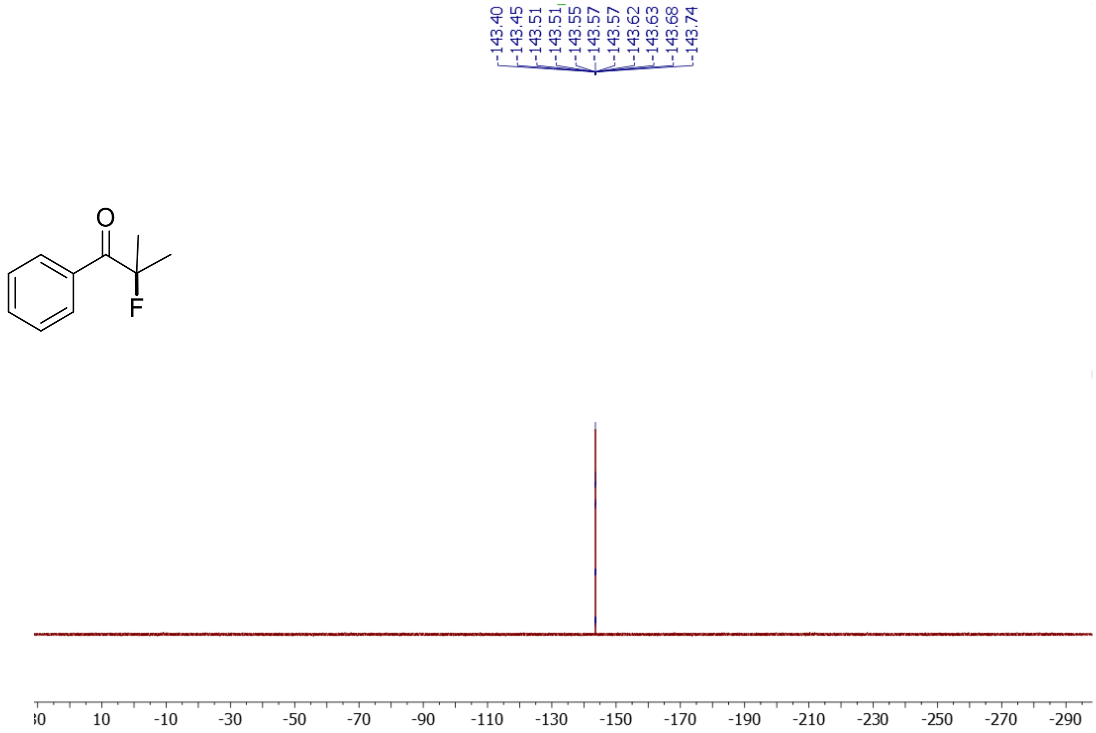

Supplementary Fig. 165. ^{1}**H NMR spectrum (400 MHz, CDCl$_3$) of 2-(1-fluoro-1-phenylethyl)pyridine.**

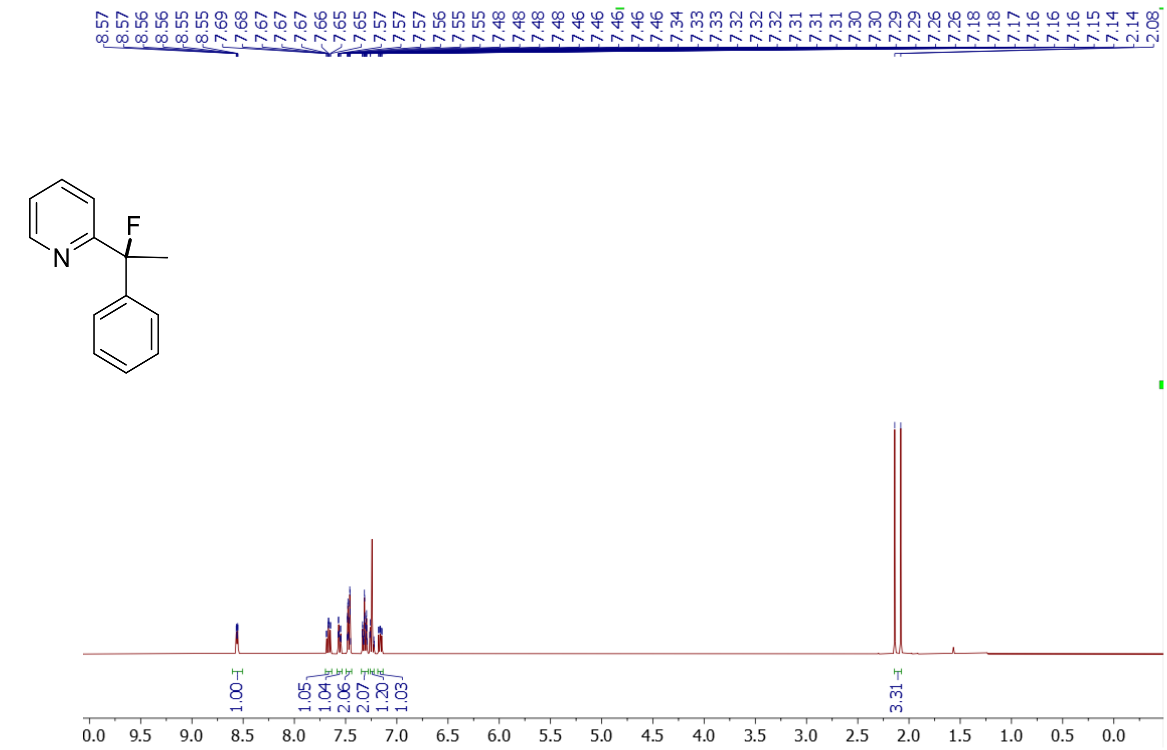

Supplementary Fig. 166. ^{19}**F NMR spectrum (376 MHz, CDCl$_3$) of 2-(1-fluoro-1-phenylethyl)pyridine.**

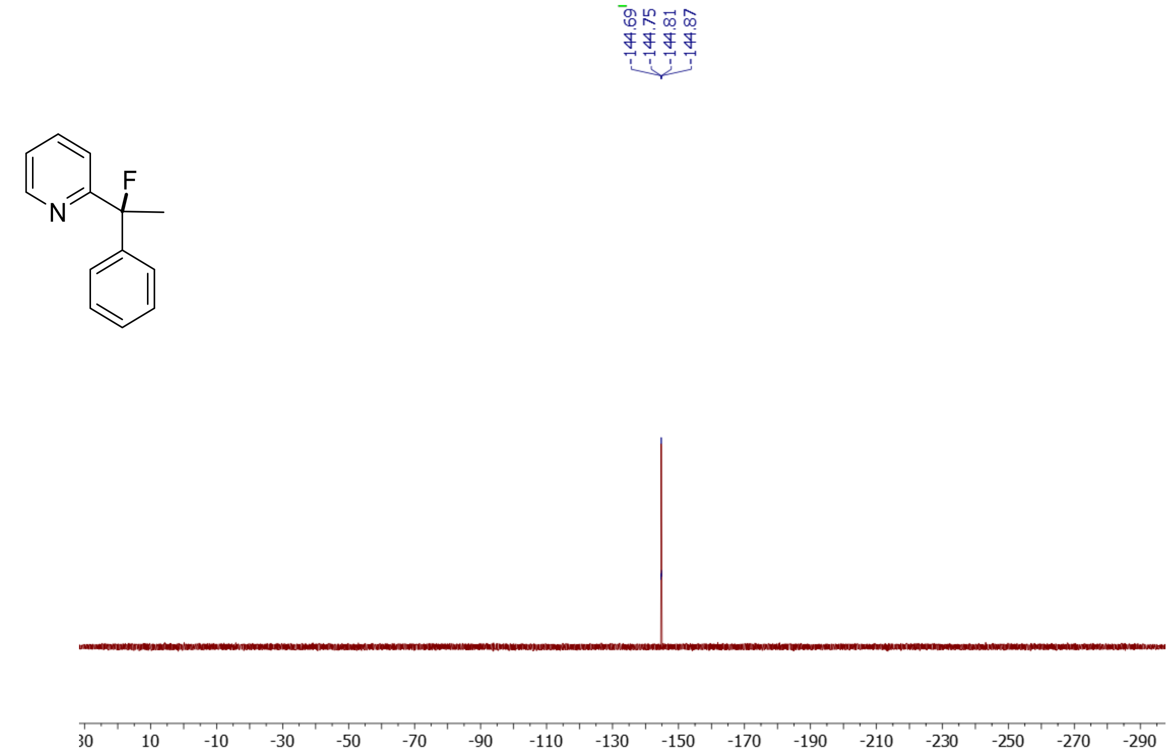

Supplementary Fig. 167. ^{1}**H NMR spectrum (400 MHz, CDCl$_3$) of tri(4-tolyl)fluoromethane.**

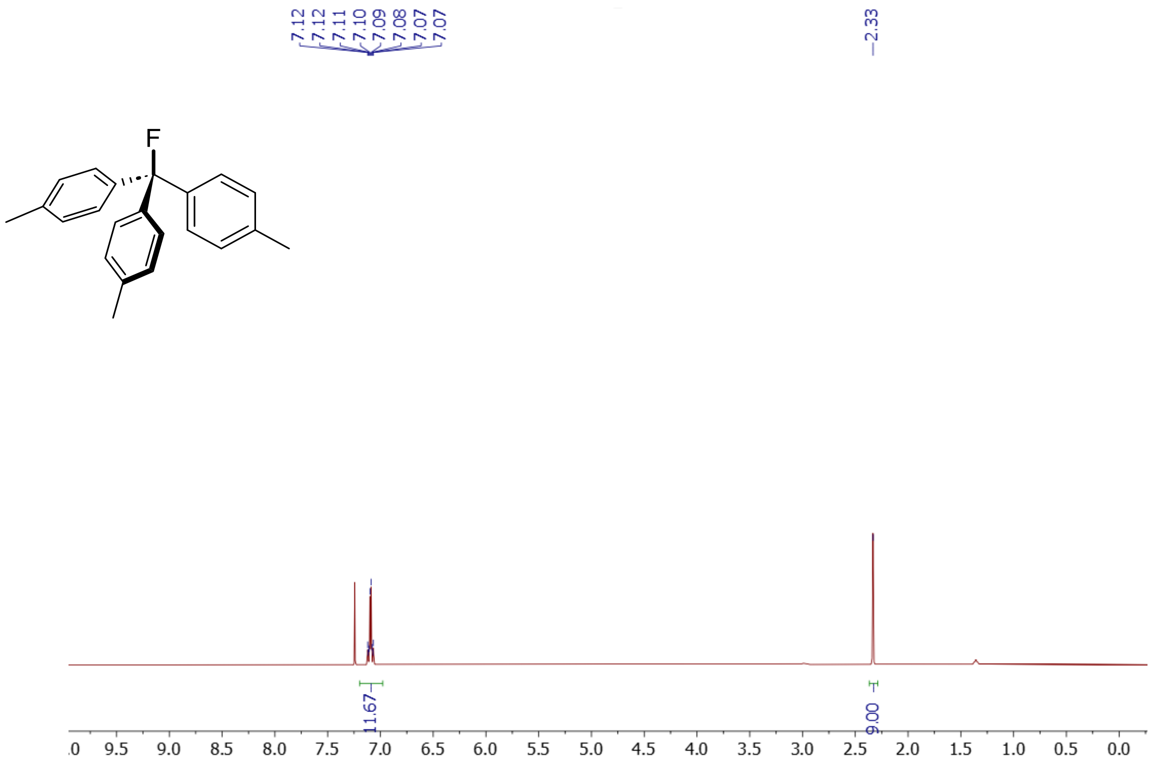

Supplementary Fig. 168. ^{19}**F NMR spectrum (376 MHz, CDCl$_3$) of tri(4-tolyl)fluoromethane.**

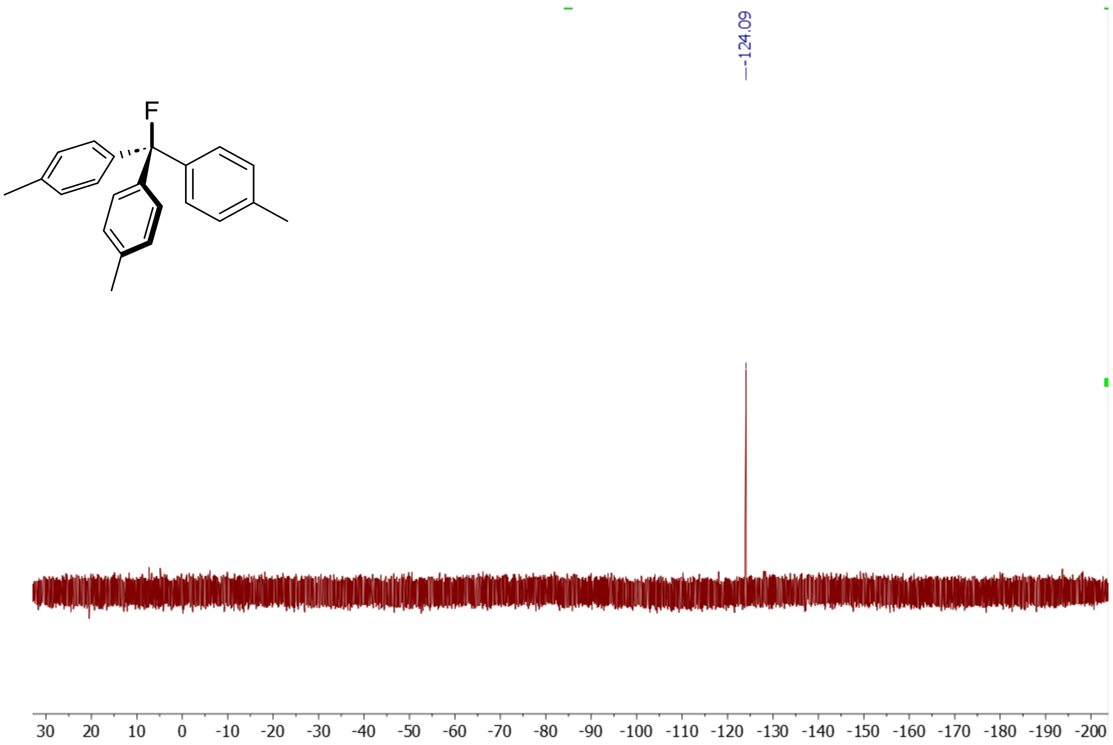

Supplementary Fig. 169. ^{1}**H NMR spectrum (400 MHz, CDCl$_3$) of (1*S*,2*S*,4*S*,5*R*)-2-((*S*)-fluoro(6-methoxyquinolin-4-yl)methyl)-5-vinylquinuclidene.**

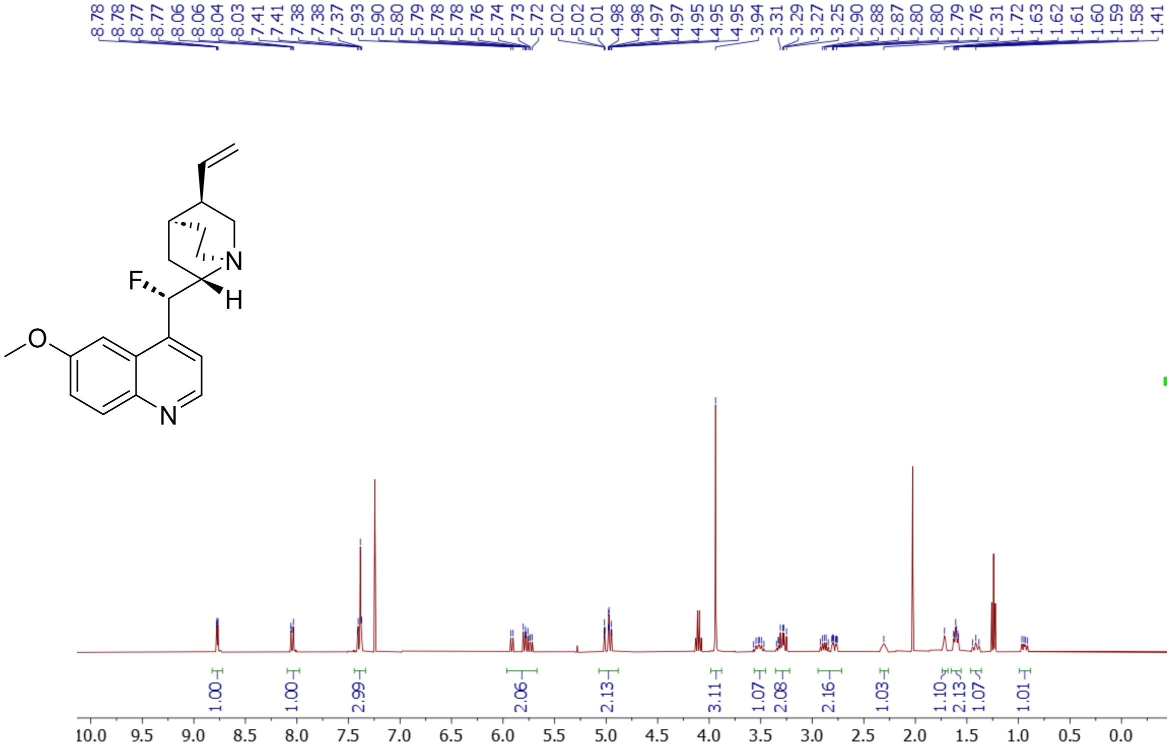

Supplementary Fig. 170. ^{19}**F NMR spectrum (376 MHz, CDCl$_3$) of (1*S*,2*S*,4*S*,5*R*)-2-((*S*)-fluoro(6-methoxyquinolin-4-yl)methyl)-5-vinylquinuclidene.**

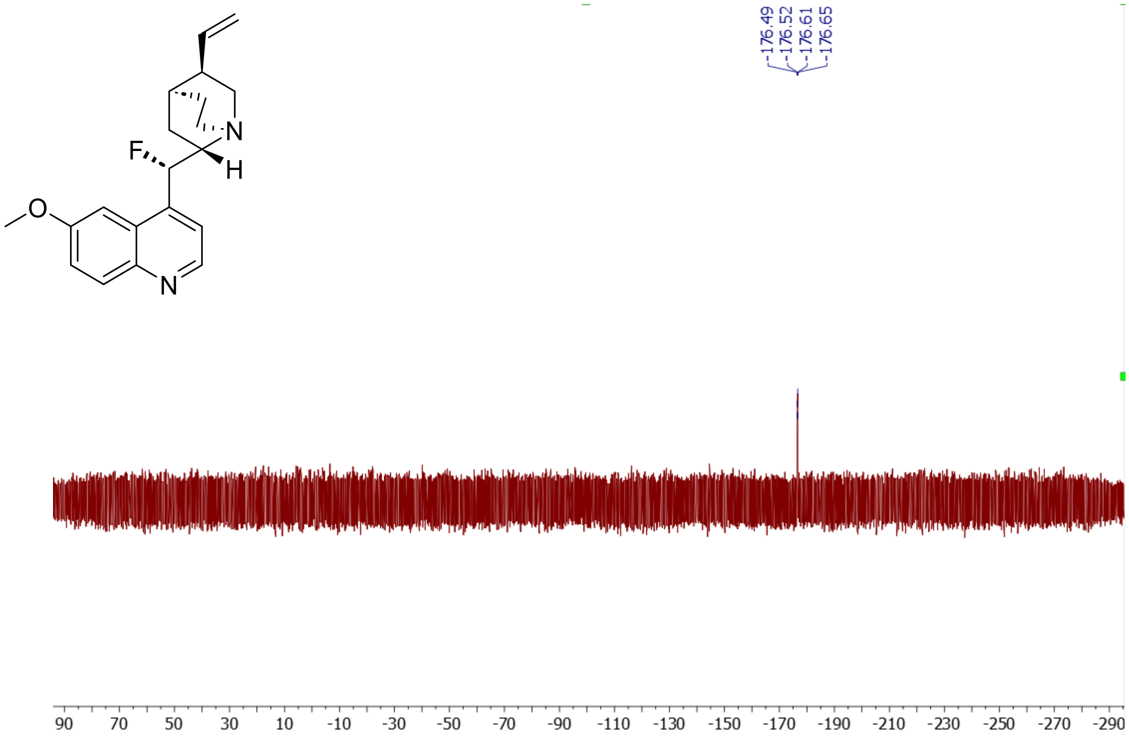

Supplementary Fig. 171. **^{1}H NMR spectrum (400 MHz, $CDCl_3$) of 5-((2-chlorophenyl)fluoro(4-fluorophenyl)methyl)pyrimidine.**

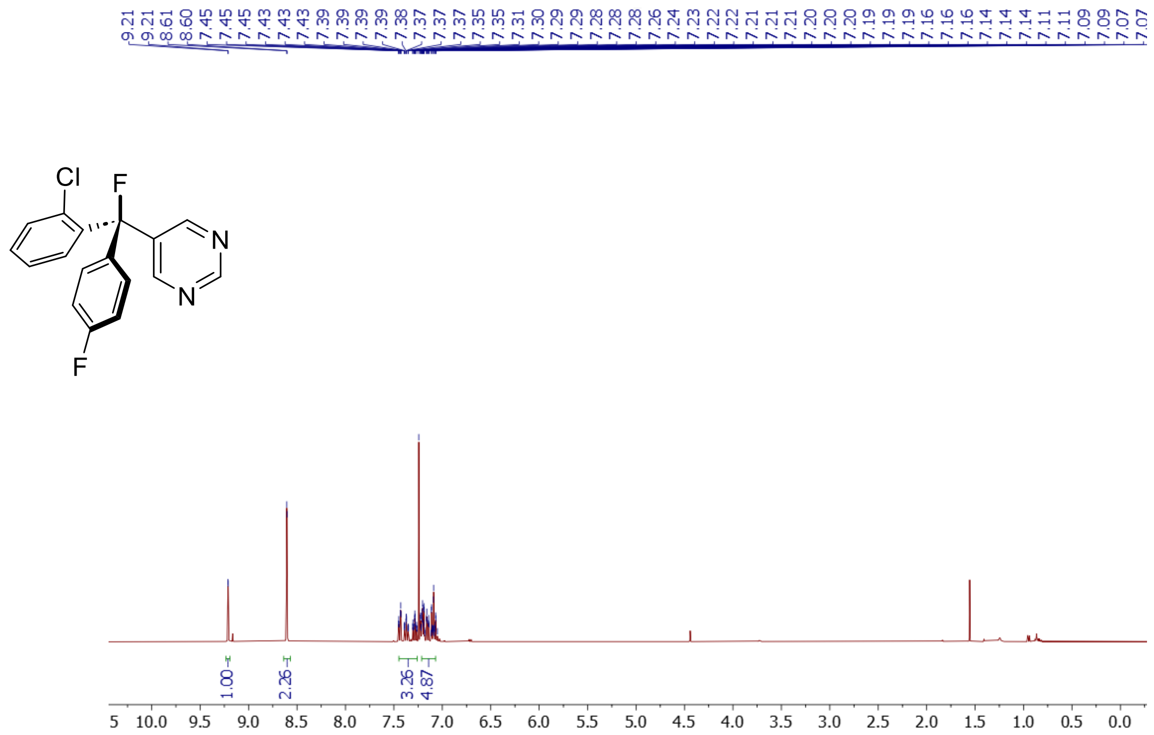

Supplementary Fig. 172. **^{19}F NMR spectrum (376 MHz, $CDCl_3$) of 5-((2-chlorophenyl)fluoro(4-fluorophenyl)methyl)pyrimidine.**

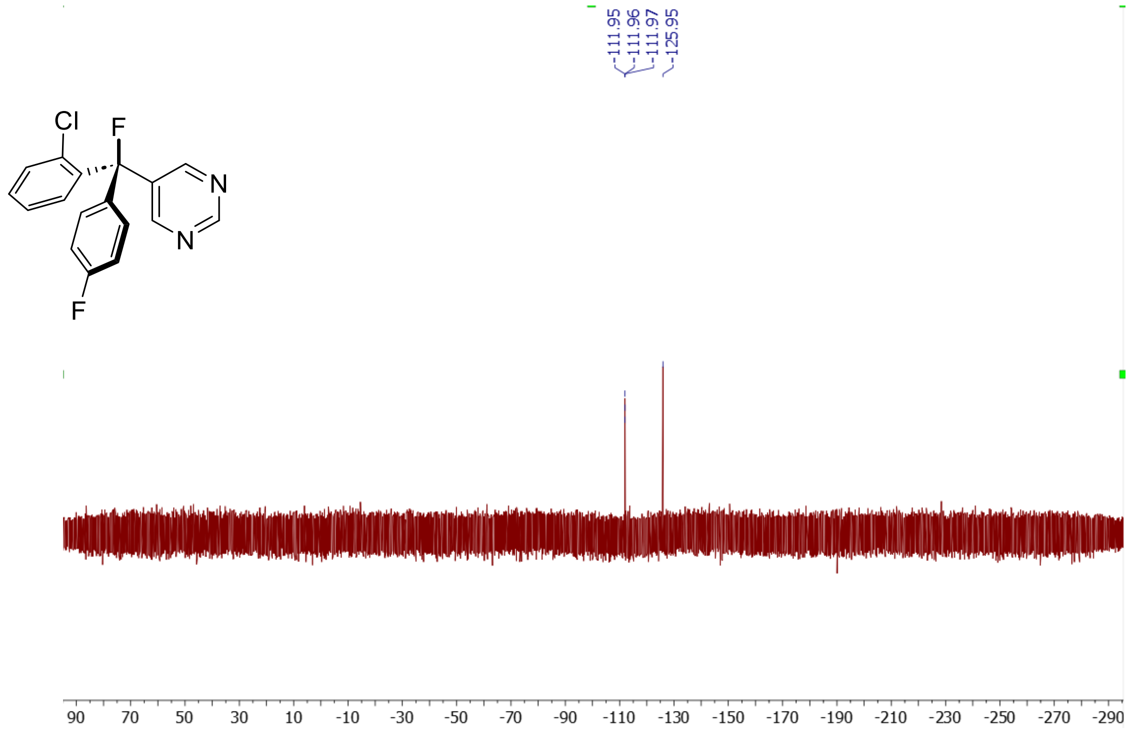

Supplementary Fig. 173. ^{1}**H NMR spectrum (400 MHz, CDCl₃) of 2-((1-benzylpiperidin-4-yl)methyl)-2-fluoro-5,6-dimethoxy-2,3-dihydro-1H-inden-1-one.**

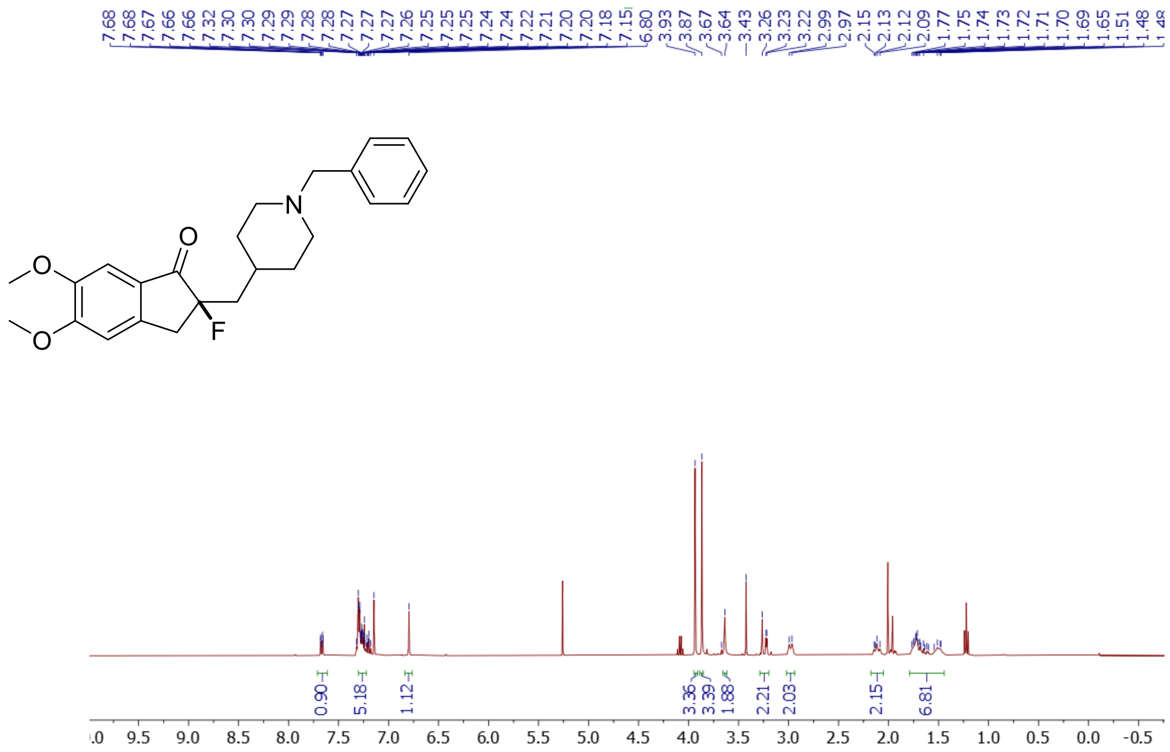

Supplementary Fig. 174. ^{19}**F NMR spectrum (376 MHz, CDCl₃) of 2-((1-benzylpiperidin-4-yl)methyl)-2-fluoro-5,6-dimethoxy-2,3-dihydro-1H-inden-1-one.**

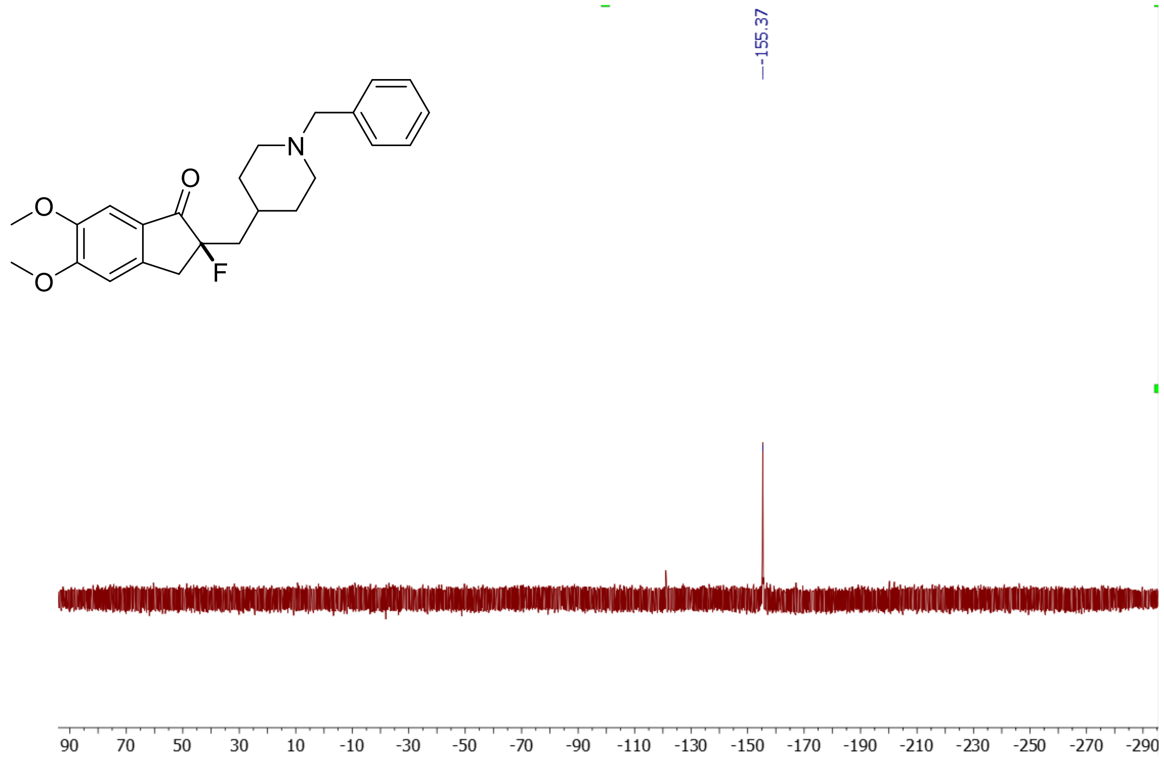

Supplementary Fig. 175. ^{1}**H NMR spectrum (400 MHz, CDCl**$_3$**) of 4-ethyl-4-fluoro-1,12-dihydro-14H-pyrano[3',4':6,7]indolizino[1,2-b]quinoline-3,14(4H)-dione.**

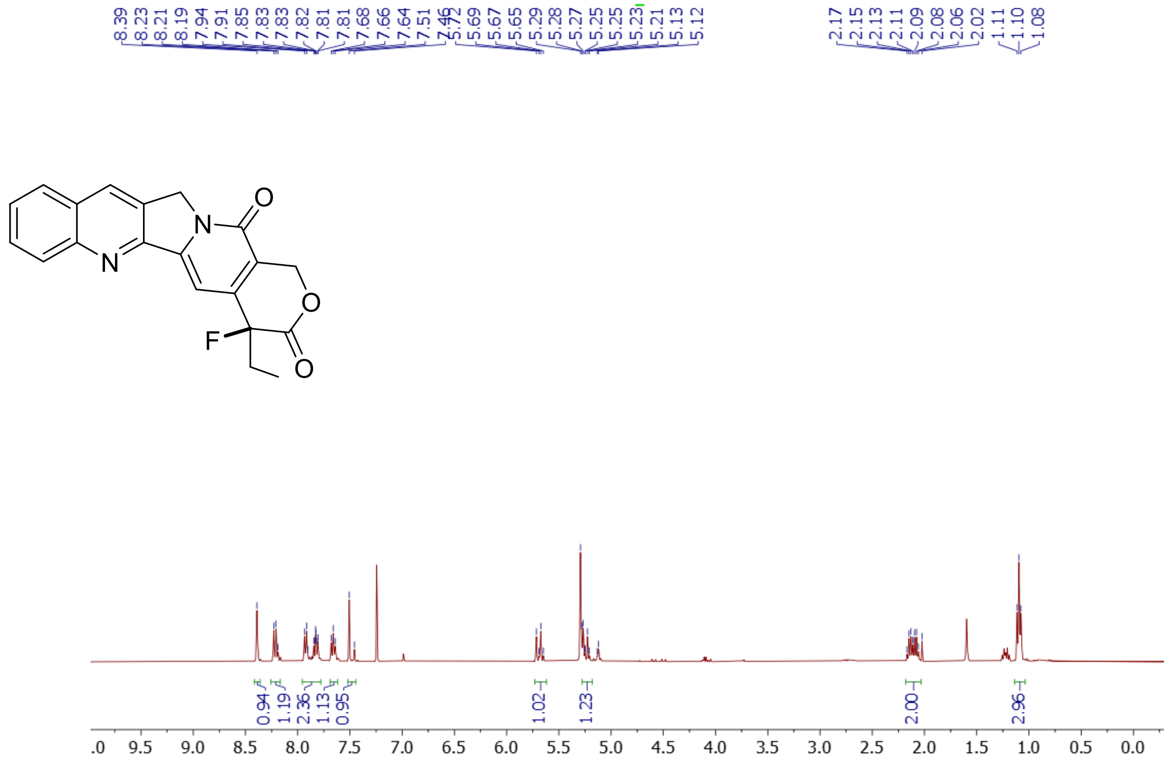

Supplementary Fig. 176. ^{19}**F NMR spectrum (376 MHz, CDCl**$_3$**) of 4-ethyl-4-fluoro-1,12-dihydro-14H-pyrano[3',4':6,7]indolizino[1,2-b]quinoline-3,14(4H)-dione.**

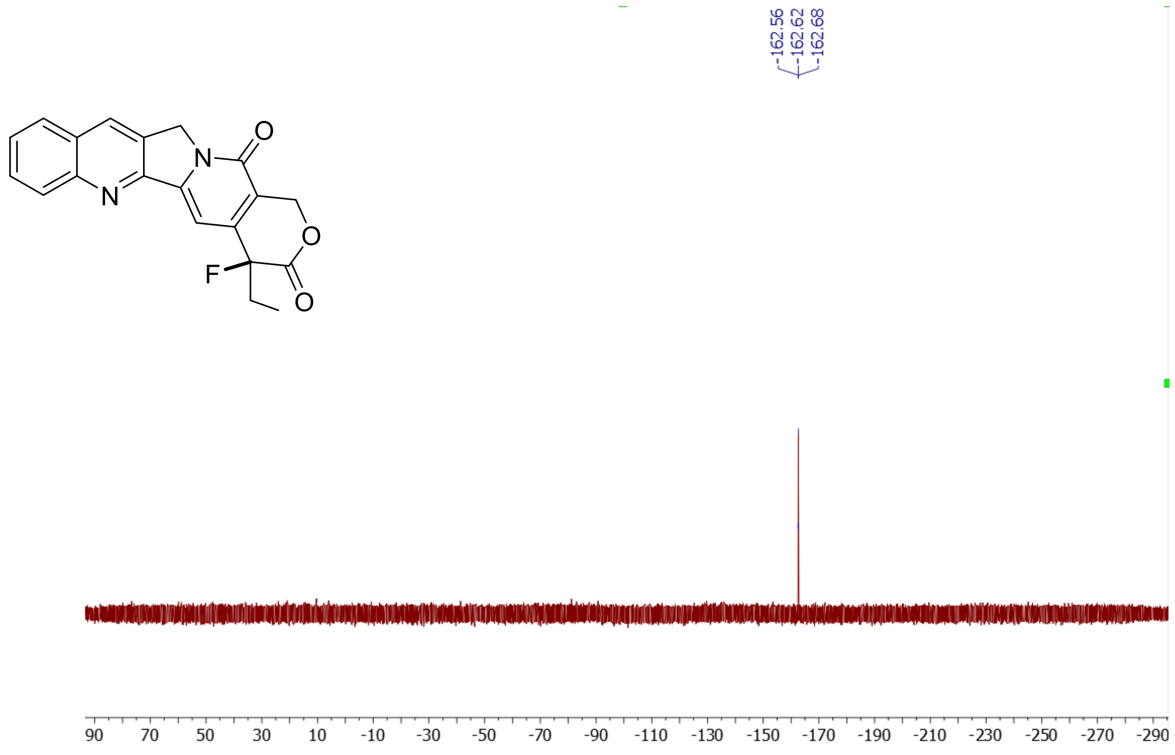

Supplementary Fig. 177. **^{1}H NMR spectrum (400 MHz, $CDCl_3$) of 2-(3-fluoropropyl)isoindoline-1,3-dione.**

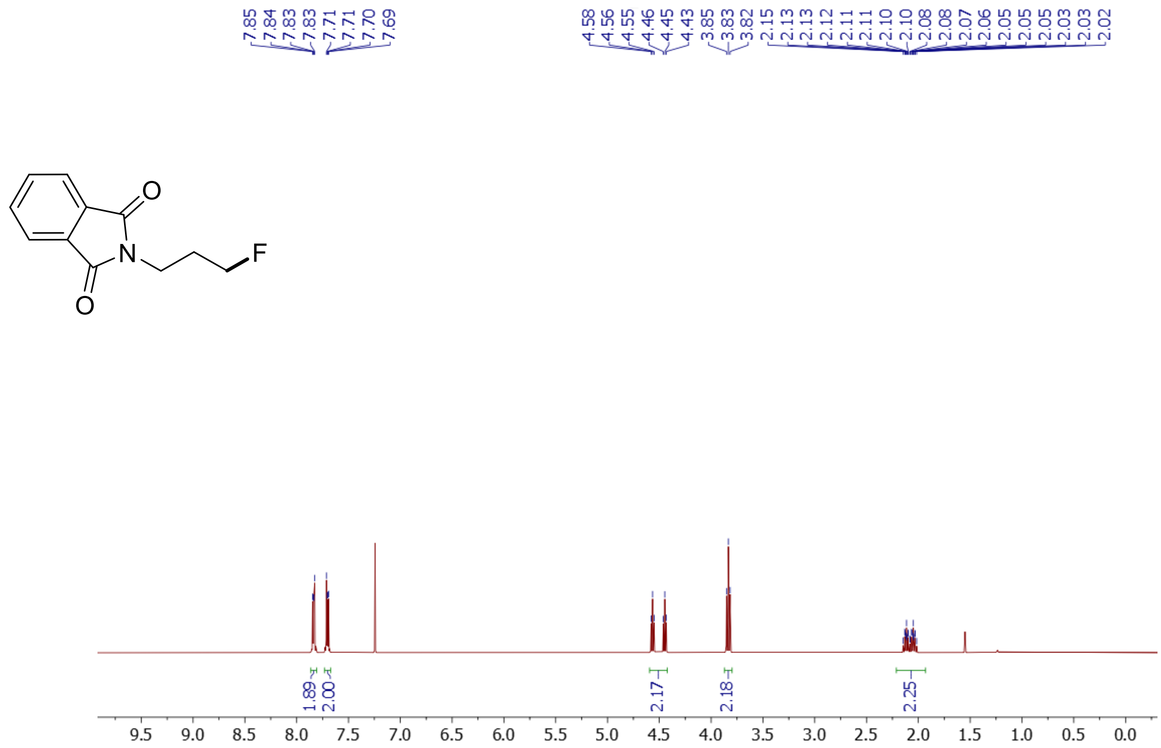

Supplementary Fig. 178. **^{19}F NMR spectrum (376 MHz, $CDCl_3$) of 2-(3-fluoropropyl)isoindoline-1,3-dione.**

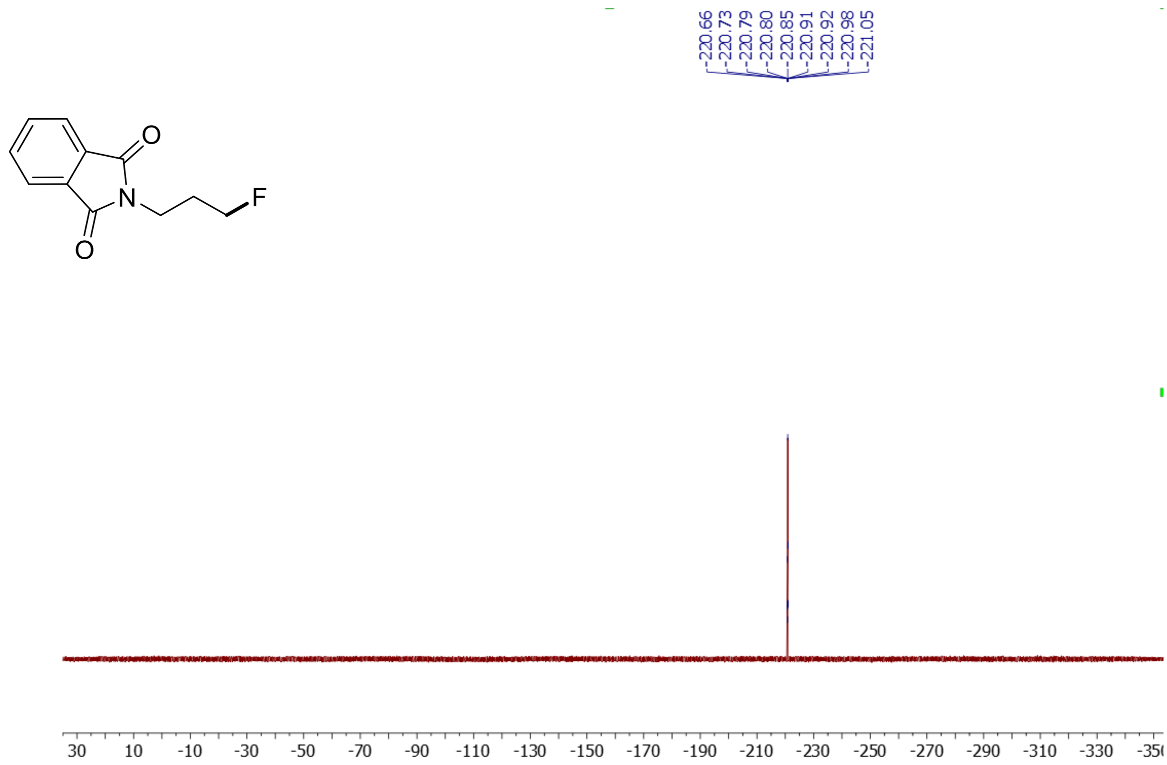

Supplementary Fig. 179. **^{1}H NMR spectrum (400 MHz, $CDCl_3$) of 5-(3-fluorobutyl)benzo[d][1,3]dioxole.**

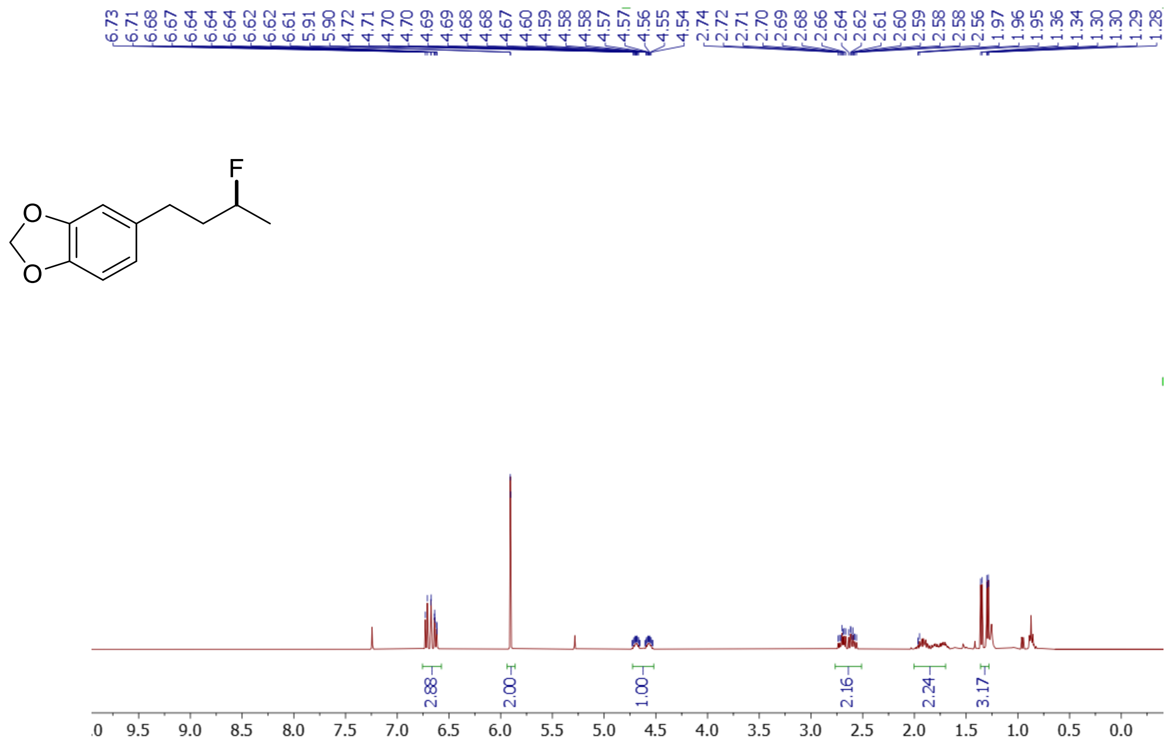

Supplementary Fig. 180. **^{19}F NMR spectrum (376 MHz, $CDCl_3$) of 5-(3-fluorobutyl)benzo[d][1,3]dioxole.**

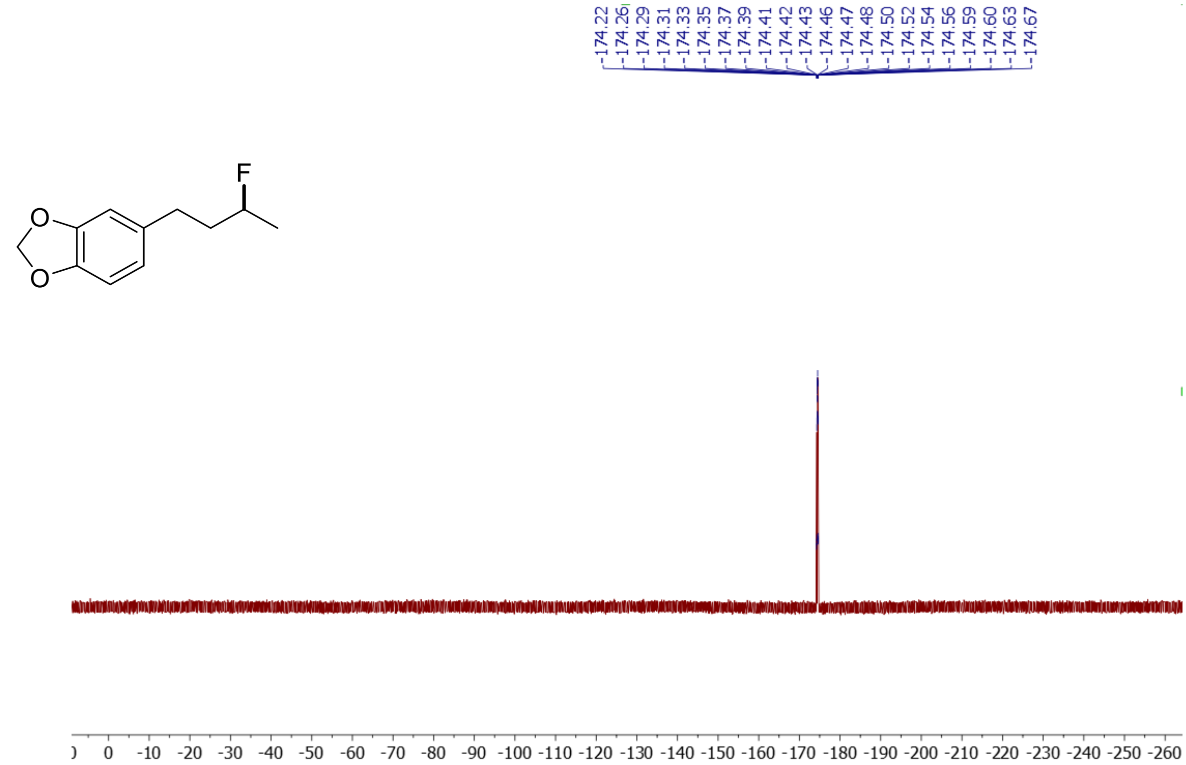

Supplementary Fig. 181. ^{1}H NMR spectrum (400 MHz, $CDCl_3$) of 1-bromo-4-(fluoro(phenyl)methyl)benzene.

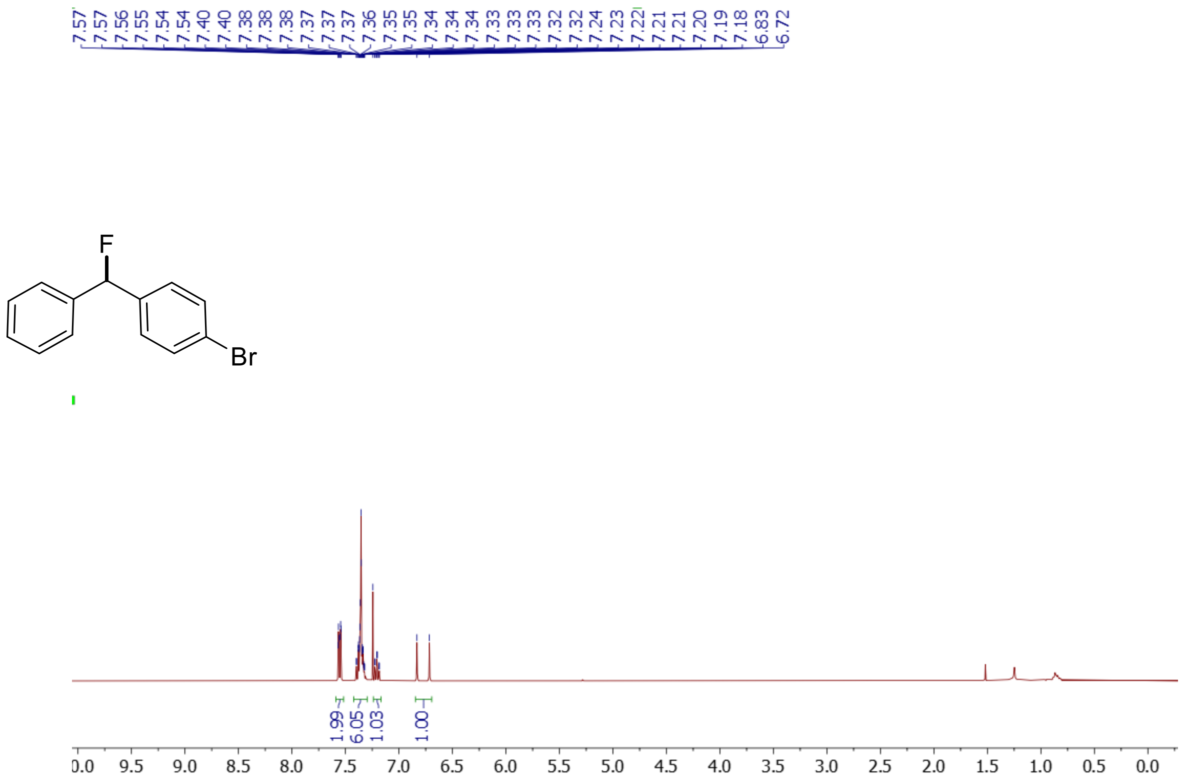

Supplementary Fig. 182. ^{19}F NMR spectrum (376 MHz, $CDCl_3$) of 1-bromo-4-(fluoro(phenyl)methyl)benzene.

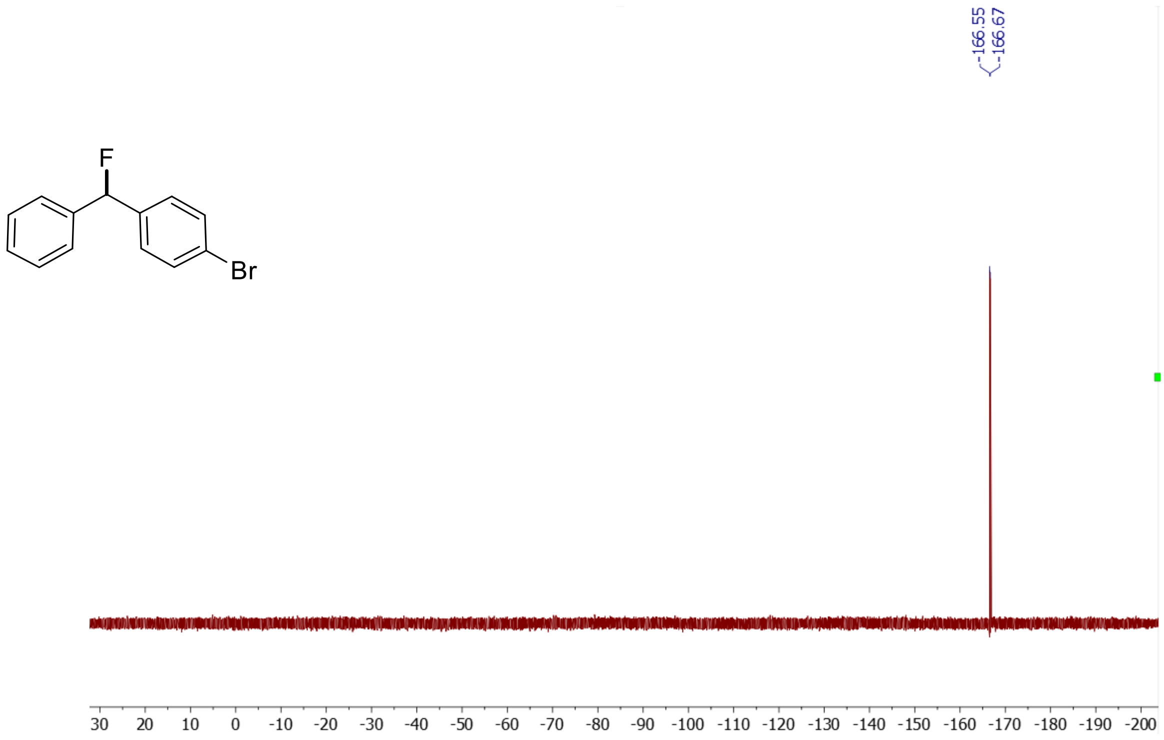

7. X-Ray Crystallography

Single crystals of each compound were mounted under parabar oil on a Mitegen micromount and immediately placed in a cold nitrogen stream at 100(2) K prior to data collection. Data were collected on either a Bruker D8 Quest equipped with a Photon100 CMOS detector and a Mo ImS source or a Bruker DUO equipped with an APEXII CCD detector and Mo fine-focus sealed source. Data were integrated with the Bruker SAINT program. Structure solution and refinement was performed using the SHELXTL/PC suite[1] and ShelXle.[2] Intensities were corrected for Lorentz and polarization effects and an empirical absorption correction was applied using Blessing's method as incorporated into the program SADABS.[3] Non-hydrogen atoms were refined with anisotropic thermal parameters. Hydrogen atoms were included in idealized positions unless otherwise noted.

Supplementary Fig. 183. X-ray Structure of (1*S*,2*S*,4*S*,5*R*)-2-((*S*)-(6-methoxyquinolin-4-yl)(phenyl)methyl)-5-vinylquinuclidine • 2 HCl (71).

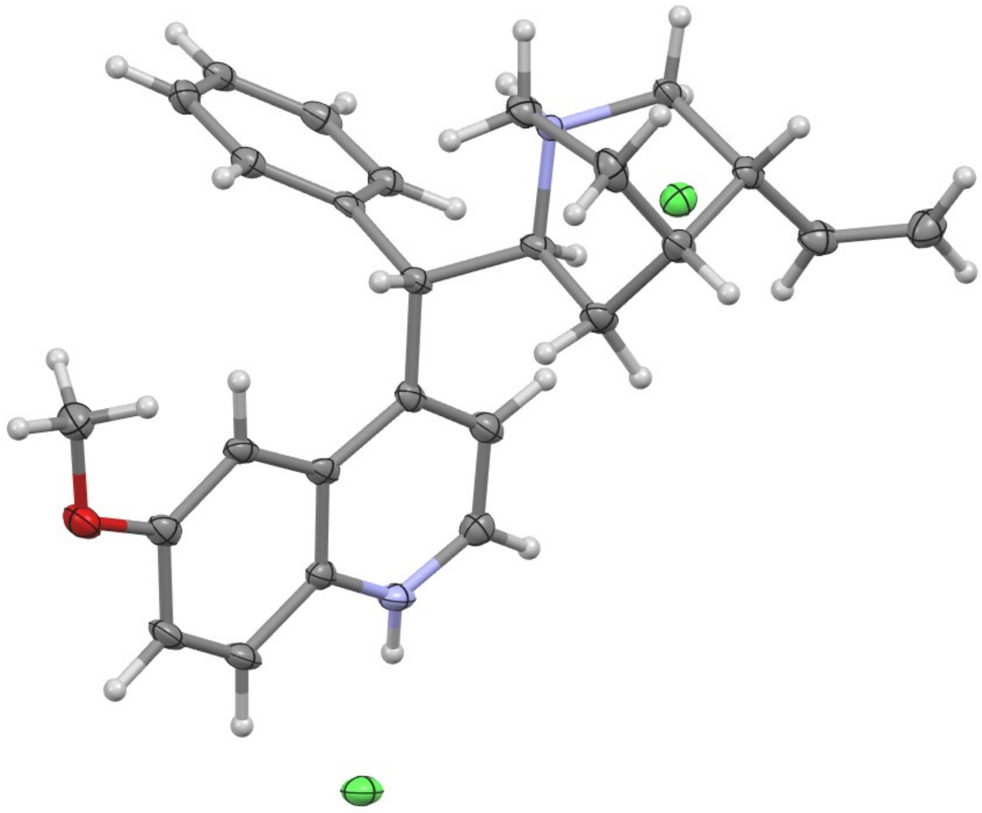

X-ray structure of (71): Three molecules of water are omitted for clarity. N-H hydrogen atoms were found in the difference map and fixed at a distance of 0.88 Å. Absolute structure parameter = 0.046 (0.016). Thermal ellipsoids are displayed at the 50% probability level. For single crystal X-ray analysis, compound (**71**) was titrated with aqueous 1 M HCl, and the HCl salt was crystallized via slow evaporation of a saturated acetone solution. Justification for the B-level alert (PLAT410_ALERT_2_B Short Intra H...H Contact): H2 and H18 are held in relatively close contact due to the sterics of the cation and the intermolecular hydrogen bonding between the cation, anions, and water molecules. (CCDC 2220189)

Supplementary Fig. 184. X-ray Structure of (1S,2S,4S,5R)-2-((S)-(4-chlorophenyl)(6-methoxyquinolin-4-yl)methyl)-5-vinylquinuclidine • 2 HCl (74).

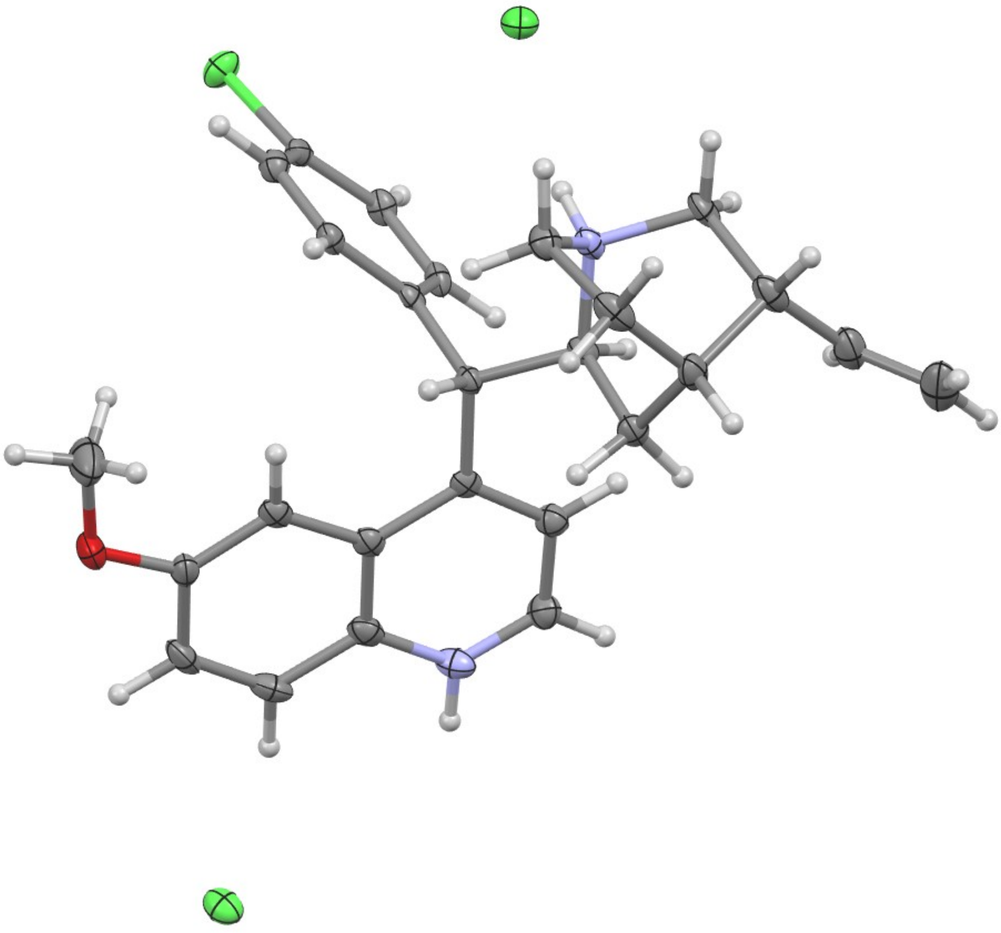

X-ray Structure of (74): One molecule of water and one molecule of methanol are omitted for clarity. N-H hydrogen atoms were found in the difference map and fixed at a distance of 0.88 Å. Absolute structure parameter = 0.004 (0.030). Thermal ellipsoids are displayed at the 50% probability level. For single crystal X-ray analysis, compound (**74**) was titrated with aqueous 1 M HCl, and the HCl salt was crystallized via evaporation of a saturated methanol solution. (CCDC 2220187)

Supplementary Fig. 185. X-ray structure of diferrocenylzinc:

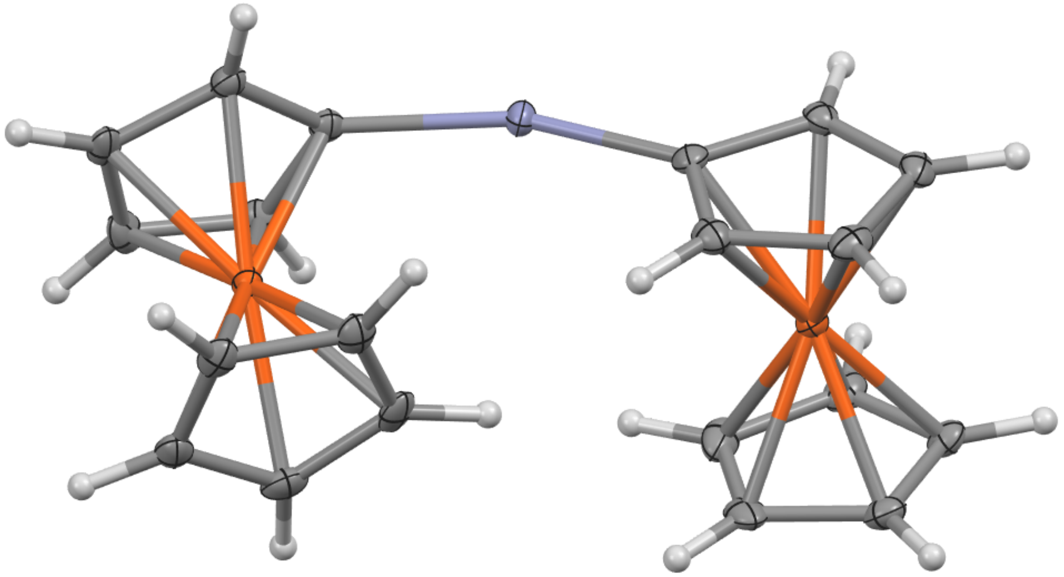

X-ray structure of diferrocenylzinc: One molecule of diferrocenylzinc among two crystallographically distinct species is shown for clarity. Thermal ellipsoids are displayed at the 50% probability level. Single crystals were grown from a saturated pentane solution at – 40 °C. (CCDC 2220190)

Supplementary Fig. 186. X-ray structure of 1-ferrocenyladamantane (62):

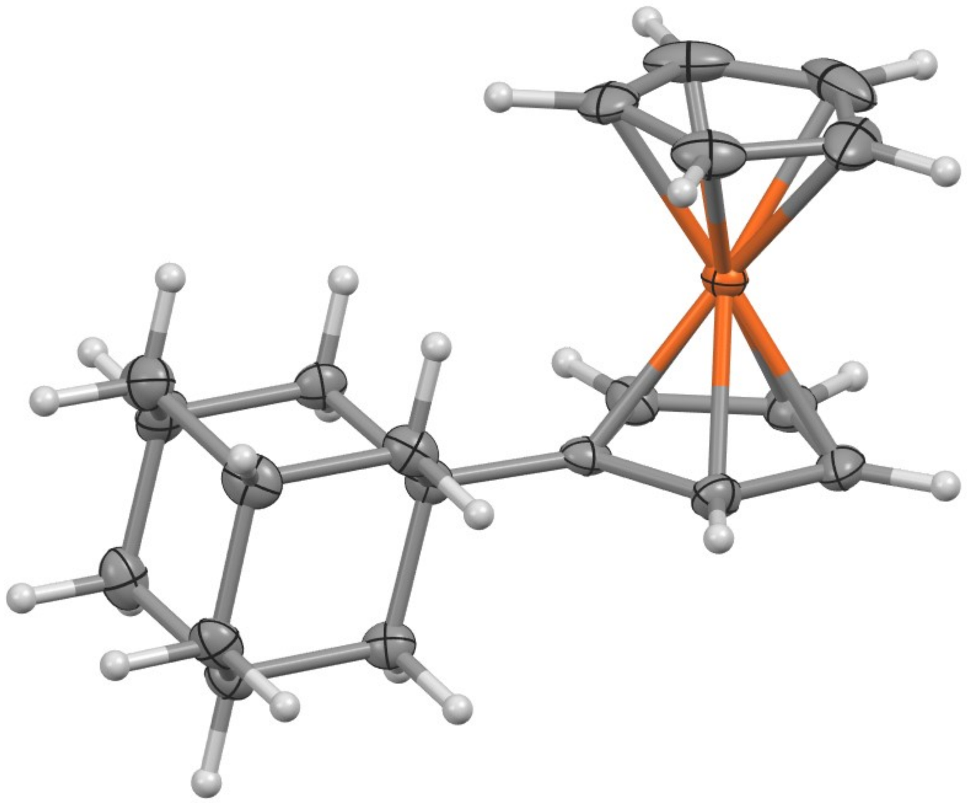

X-ray structure of (62): One molecule of (**62**) among two crystallographically distinct species is shown for clarity. Thermal ellipsoids are displayed at the 50% probability level. X-ray quality single crystals were grown via evaporation of a saturated pentane solution. (CCDC 2220188)

Supplementary Fig. 187. X-ray structure of (3-mesityl-3-methyl-1-phenylindolin-2-one (63).

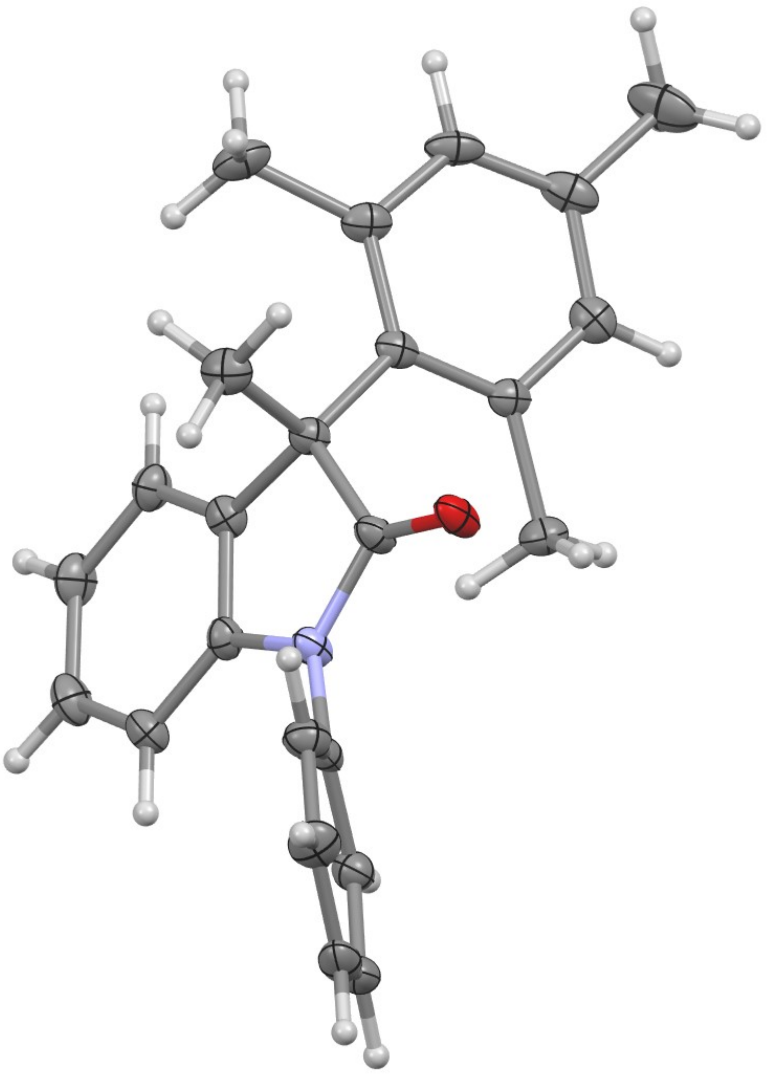

X-ray structure of (63): Thermal ellipsoids are displayed at the 50% probability level. Single crystals for X-ray analysis were grown from evaporation of a saturated pentane solution. (CCDC 2220191)

Supplementary Fig. 188. X-ray structure of 3-methyl-3-(3-methylbenzofuran-2-yl)-1-phenylindolin-2-one (66).

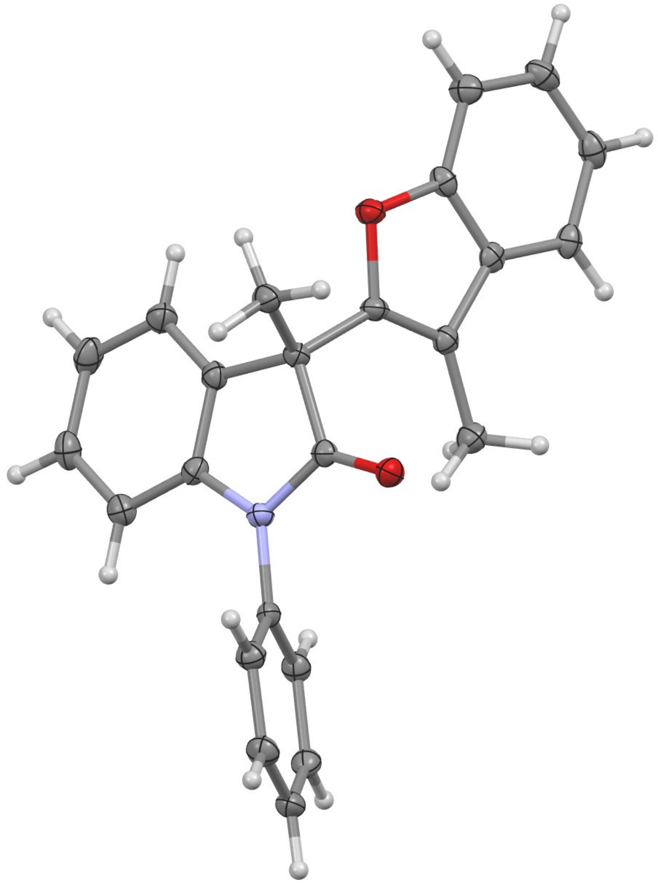

X-ray structure of (66): Thermal ellipsoids are displayed at the 50% probability level. X-ray quality single crystals were grown from evaporation of a saturated ethyl acetate solution. (CCDC 2220422)

Supplementary Fig. 189. X-ray structure of 1-(2,4-dimethoxyphenyl)adamantane (39):

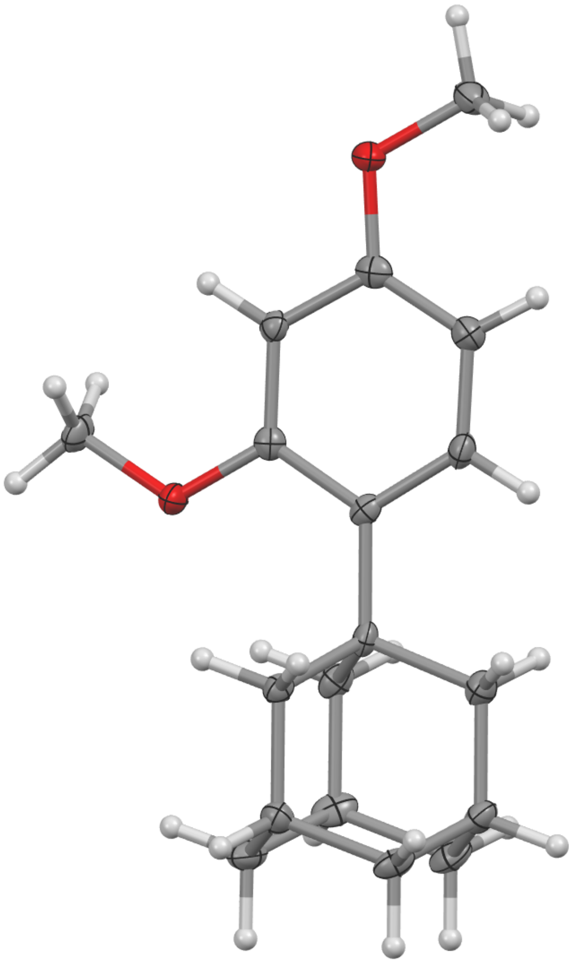

X-ray structure of (39): One molecule of (**39**) among two crystallographically distinct species is shown for clarity. Thermal ellipsoids are displayed at the 50% probability level. Single crystals were grown from slow evaporation of a pentane solution. (CCDC 2220185)

Supplementary Fig. 190. X-ray structure of 3-methyl-1,3-diphenylindolin-2-one (7).

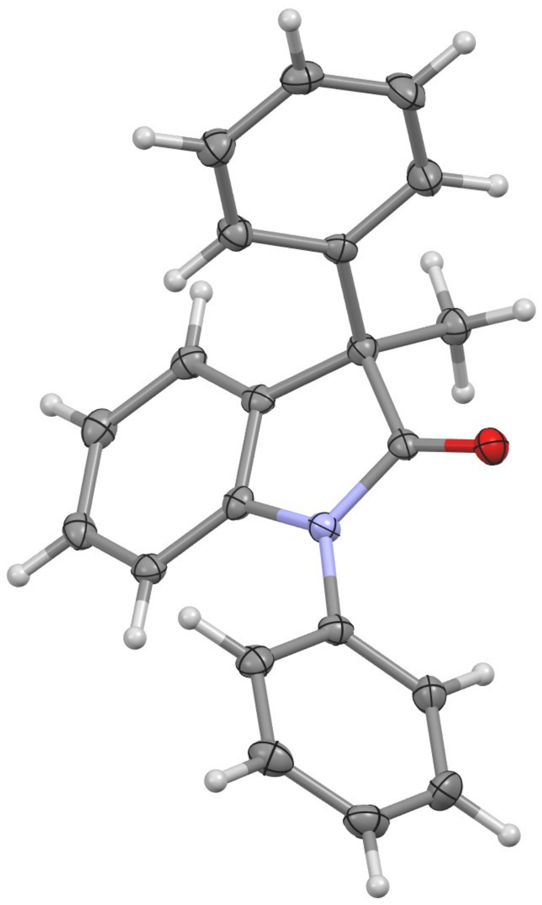

X-ray structure of (7): Thermal ellipsoids are displayed at the 50% probability level. X-ray quality single crystals were grown from evaporation of a saturated ethyl acetate solution. (CCDC 2220186)

Supplementary Fig. 191. The asymmetric unit of (33).

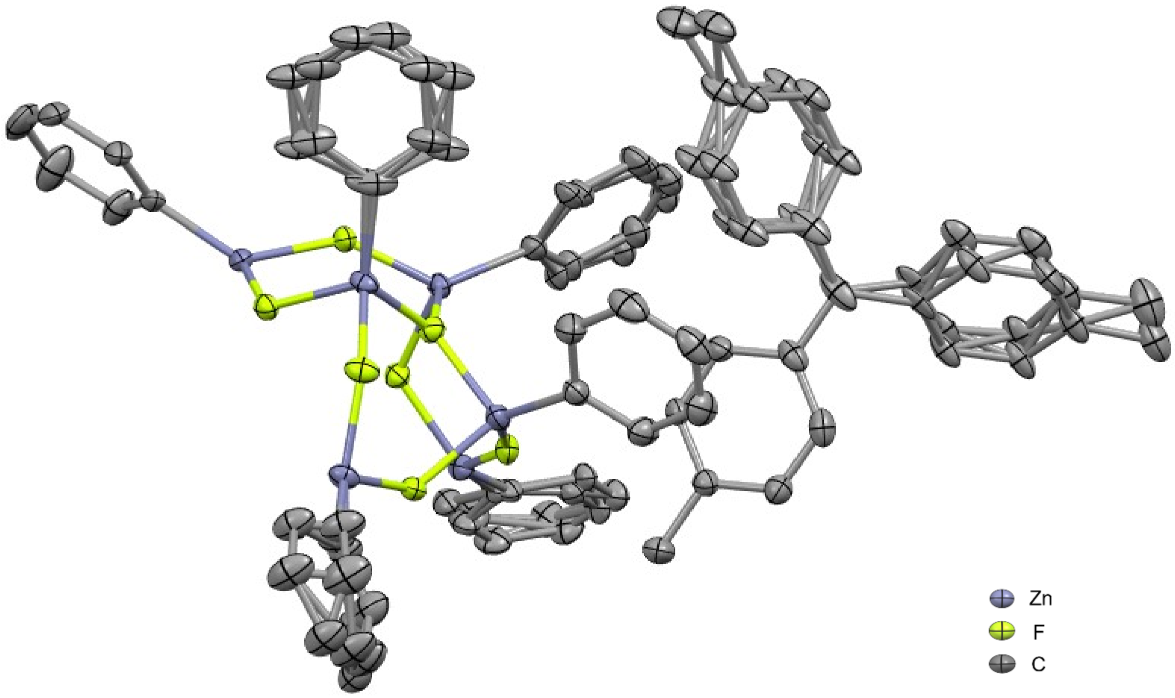

The asymmetric unit of (33): The asymmetric unit of (33) consists of one cation and one half of the dianion. Four phenyl rings of the dianion are disordered over two orientations. The like C-C and Zn-C distances were restrained to be similar. The C7/C7B atom pair was constrained to have equal x,y,z positions and equal anisotropic displacement parameters. Two of the aryl groups on the cation are disordered over two positions. The like C-C distances were restrained to be similar. Similar displacement amplitudes were imposed on disordered sites overlapping by less than the sum of the van der Waals radii. A total of eight highly disordered dichloromethane solvate molecules per unit cell were removed from the model via the Squeeze routine in PLATON. Thermal ellipsoids are displayed at the 50% probability level. Single crystals were grown from a solution in dichloromethane at -40 °C. Select bond lengths and angles: Zn1-F1 2.0261(16) Å, Zn1-F4 1.9198(19) Å, Zn2-C7 1.945(3) Å, Zn4-F3-Zn2 118.95°(8), Zn4-F3-Zn5 120.91°(8), Zn2-F3-Zn5 120.03°(8), Zn1-F4-Zn5 149.07°(10), Zn2-F2-Zn3 142.80°(9). (CCDC 2220184)

Supplementary Fig. 192. X-ray characterization of a diphenylzinc trimer:

In a nitrogen-filled glovebox, diphenylzinc (200 mg, 0.91 mmol) was dissolved in minimal toluene (4 mL), filtered through a 0.2 μm PVDF syringe filter, and the resulting solution was allowed to stand at -40 °C overnight. Colorless crystals of a diphenylzinc trimer were afforded, in addition to crystals of the known dimeric species.[4]

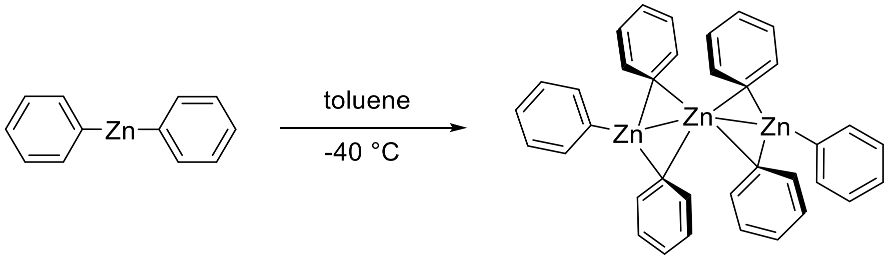

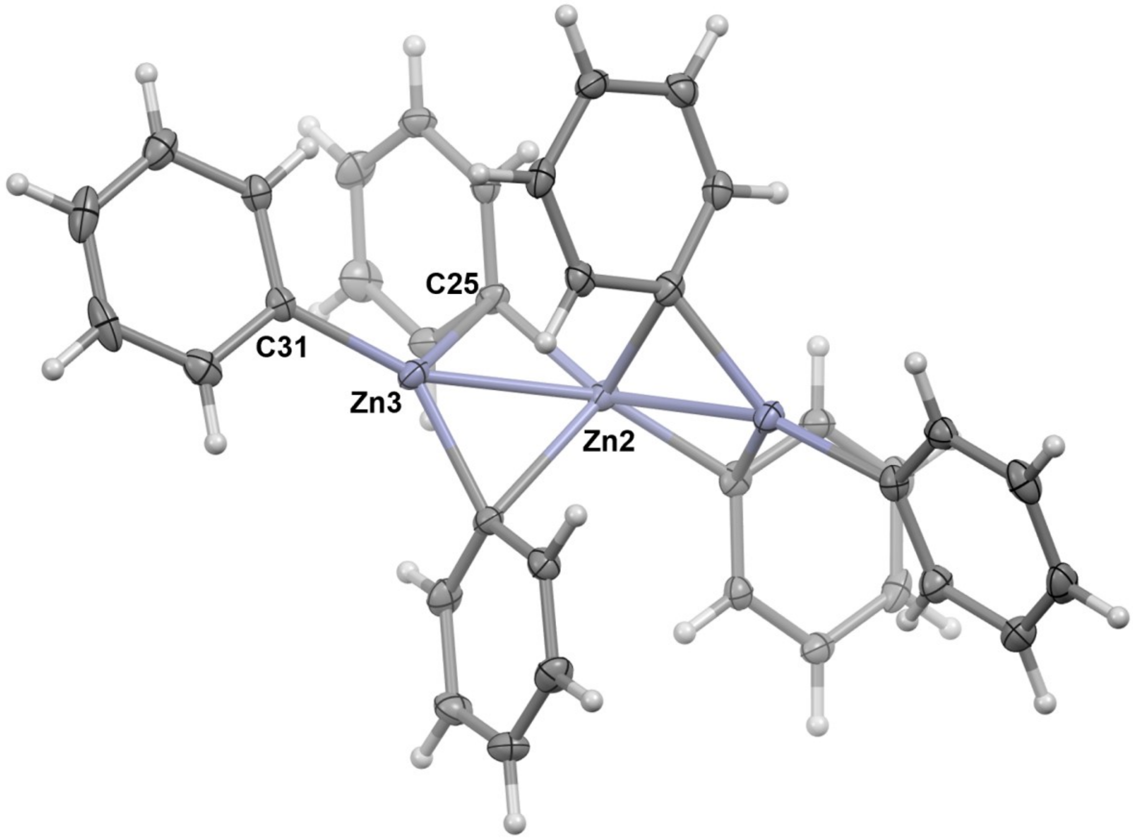

X-ray structure of a diphenylzinc trimer: Thermal ellipsoids are displayed at the 50% probability level. Zn3-C31 1.948(3) Å, Zn3-Zn2 2.5845(7) Å, Zn3-C25 2.376(3) Å, Zn2-C25 2.018(3) Å, Zn3-Zn2-Zn1 117.312°(19), Zn1-C1 1.951(3) Å, Zn1-C7 2.024(3) Å, Zn1-C13 2.351(3) Å. (CCDC 2261444)

Supplementary Fig. 193. Isolation and Characterization of Ion Pair (1).

In a nitrogen-filled glovebox, A TBAF solution in THF (120 µL, 0.12 mmol) was evaporated under reduced pressure to remove the solvent. The resulting residue was redissolved in toluene (1.0 mL). Diphenylzinc (26 mg, 0.12 mmol) was added, and the mixture was stirred for 30 minutes at room temperature affording ion pair (**1**). X-ray quality single crystals of ion pair (**1**) were obtained from dichloroethane solution layered with one volume of pentane at -40 °C.

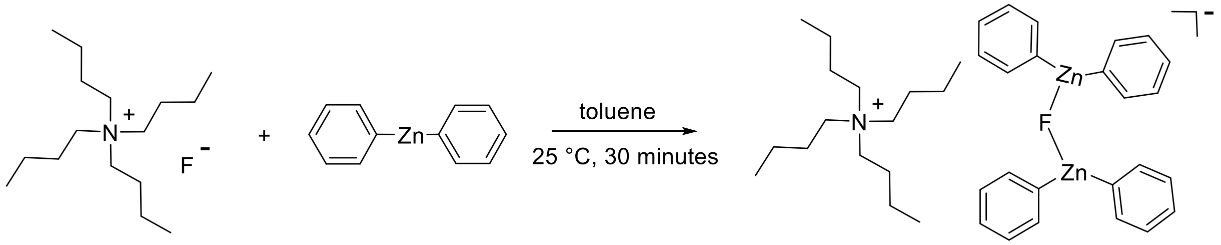

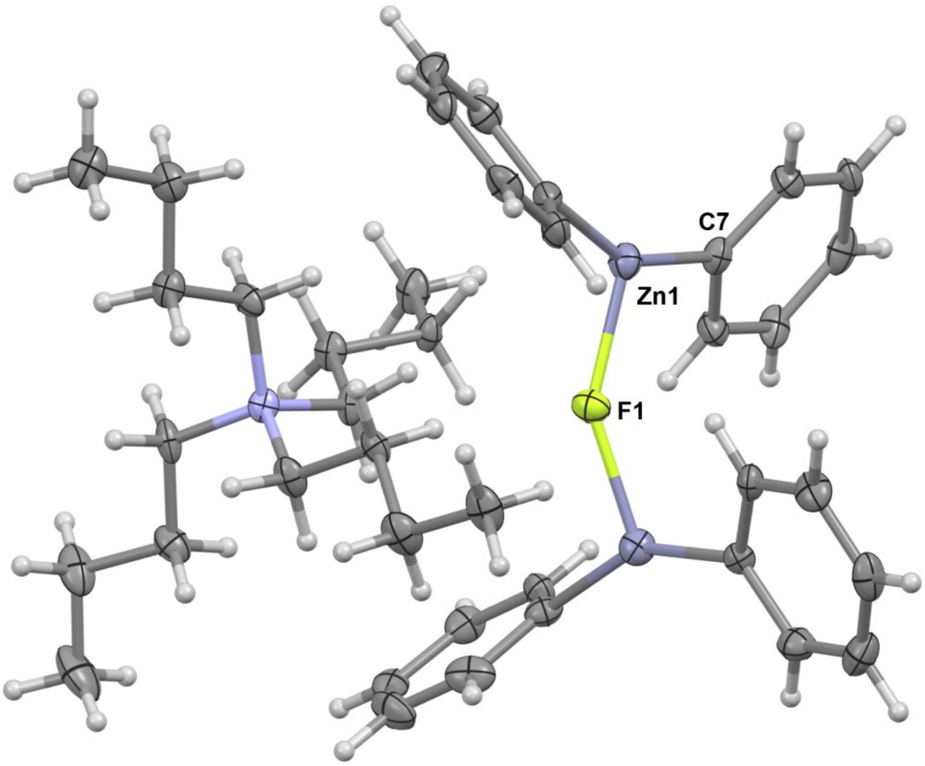

X-ray structure of (1): Thermal ellipsoids are displayed at the 50% probability level. X-ray quality single crystals were grown from dichloroethane solution layered with one volume of pentane at -40 °C. Thermal ellipsoids are displayed at the 50% probability level. Zn1-C7 1.955(5) Å, Zn1-C1 1.967(6) Å, Zn2-C13 1.969(6) Å, Zn2-C19 1.970(6) Å, Zn1-F1 1.990(3) Å, Zn2-F1 1.996(3) Å, C7-Zn1-C1 149.9°(2), C7-Zn1-F1 105.90°(19), Zn1-F1-Zn2 148.04°(18). (CCDC 2261445)

Supplementary Fig. 194. Characterization of an unsolvated lithium triarylzincate complex (2):

In a nitrogen-filled glovebox, diphenylzinc (100 mg, 0.45 mmol) was dissolved in toluene. Phenyl lithium (38.0 mg, 0.45 mmol) was added as solid, and the mixture was allowed to stir at room temperature for 30 minutes. The mixture was filtered through a 0.2 μm PVDF syringe filter and allowed to stand at room temperature, affording crystals of (**2**) within one hour.

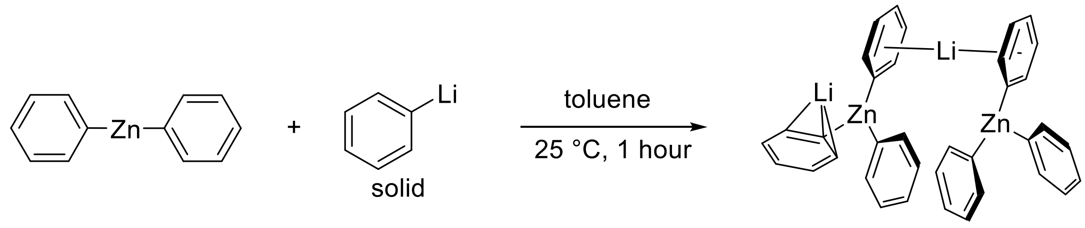

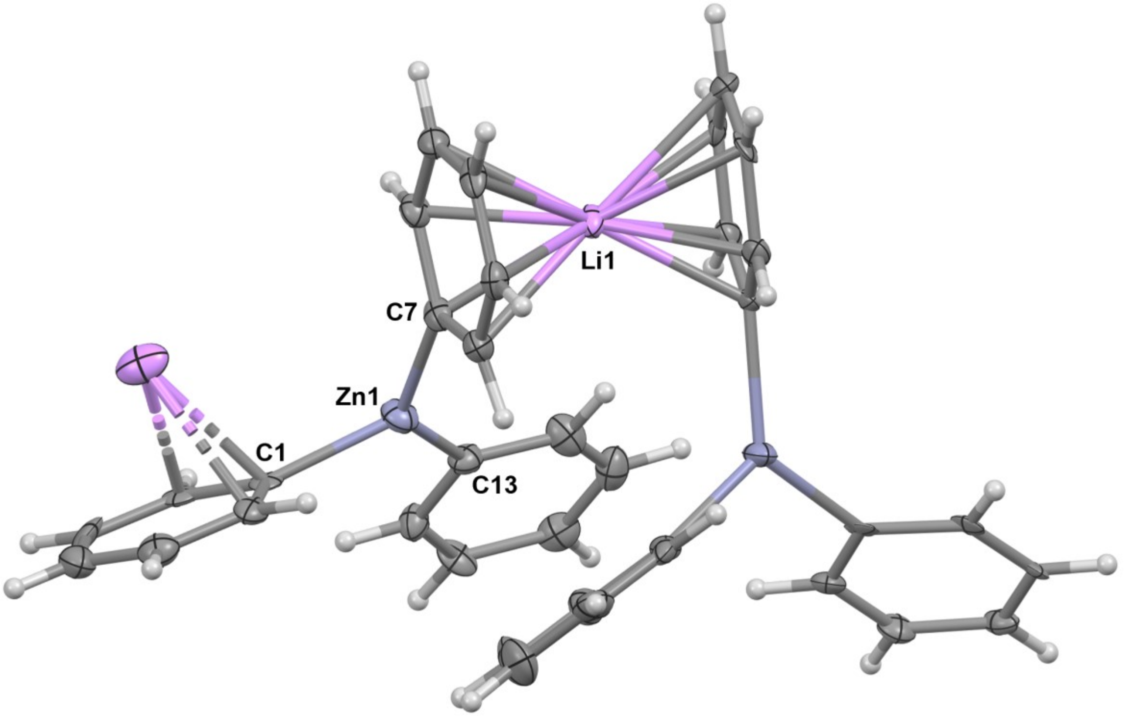

X-ray structure of (2): Thermal ellipsoids are displayed at the 50% probability level. X-ray quality single crystals were grown from a saturated toluene solution at 25 °C. An additional lithium triphenyl zincate species in the asymmetric unit has been omitted for clarity. Zn1-C1 2.03(1) Å, Zn1-C7 2.03(1) Å, Zn1-C13 1.98(1) Å, Zn2-C19 2.015(10) Å, Zn2-C31 2.036(11) Å, Zn2-C25 2.046(11) Å, Zn3-C49 1.998(11) Å, Zn3-C43 2.028(12) Å, Zn3-C37 2.037(11) Å, C13-

Zn1-C7 120.2°(5), C13-Zn1-C1 133.5°(5), C7-Zn1-C1 106.3°(4). Justification for the B-level alert (PLAT341_ALERT_3_B Low Bond Precision on C-C Bonds): This crystal diffracted weakly at high angle and due to icing issues only a minimal amount of data could be collected. Attempts to collect more, intense data were unsuccessful (CCDC 2261447)

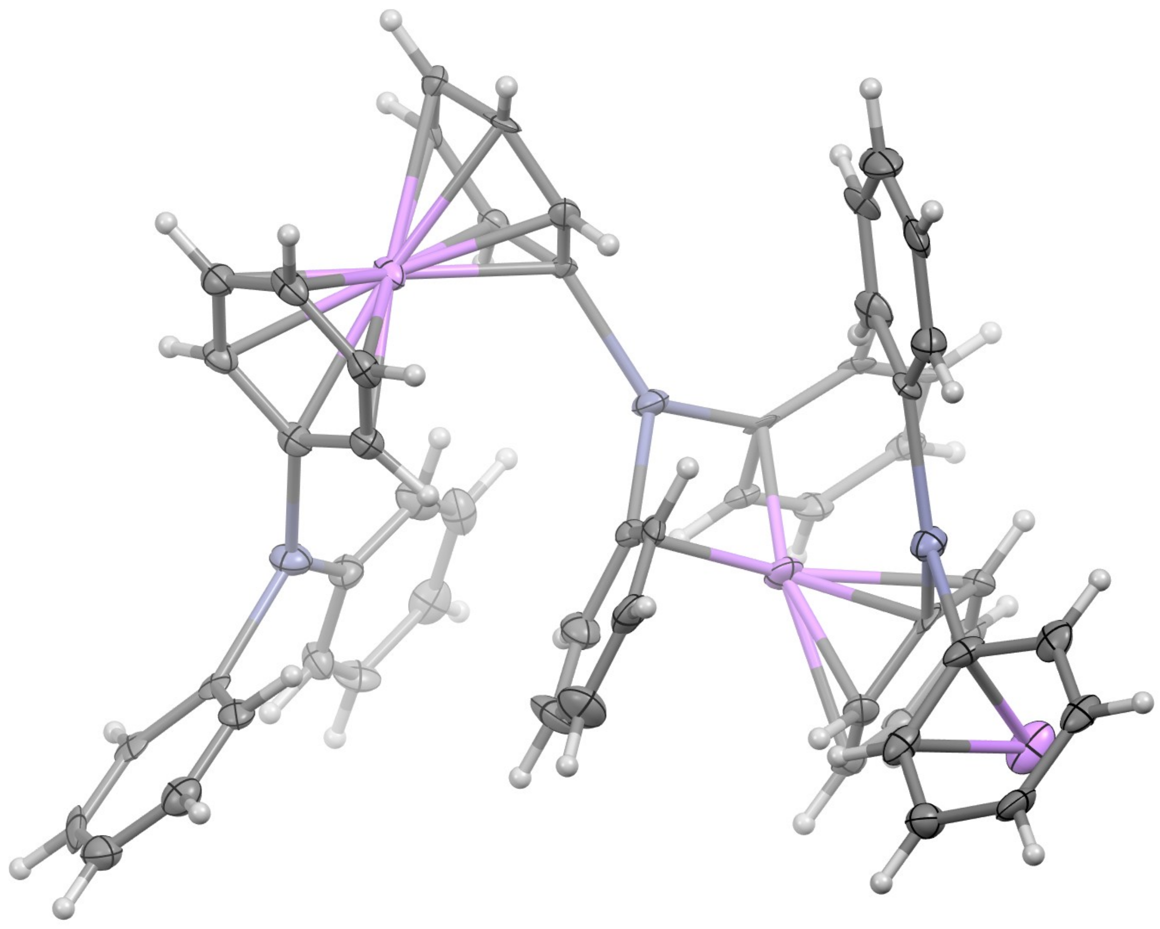

Complete asymmetric unit of (2). Thermal ellipsoids are displayed at the 50% probability level. X-ray quality single crystals were grown from a saturated toluene solution at 25 °C. (CCDC 2261447)

8. References

1. Sheldrick, G. M. Crystal Structure Refinement with *shelxl*. *Acta Crystallogr. C Struct. Chem.* **71**, 3–8 (2015).

2. Hübschle, C. B., Sheldrick, G. M., Dittrich, B. *shelxle*: A Qt Graphical User Interface for *shelxl*. *J. Appl. Crystallogr*. **44**, 1281–1284 (2011).

3. Bruker (2016). APEX3, SADABS, SAINT, SHELXTL, XCIF, XPREP. Bruker AXS, Inc., Madison, Wisconsin, USA.

4. Markies, P. R., Schat, G., Akkerman, O. S., Bickelhaupt, F. Coordinational Behavior of Solvent-Free Diorganylzinc Compounds: The Remarkable X-ray Structure of Dimeric Diphenylzinc. *Organometallics* **9**, 2243–2247 (1990).

5. Dryzhakov, M., Moran, J. Autocatalytic Friedel–Crafts Reactions of Tertiary Aliphatic Fluorides Initiated by $B(C_6F_5)_3{\cdot}H_2O$. *ACS Catal*. **6**, 3670–3673 (2016).

6. Middleton, W. J. New Fluorinating Reagents. Dialkylaminosulfur Fluorides. *J. Org. Chem*. **40**, 574-578 (1975).

7. Tramontini, M.; Angiolini, L.; Fouquey, C.; Jacques, J. Stereochemistry of Amino-Carbonyl Compounds – VII, Absolute and Relative Configuration of some Diastereomeric 1,3-Amino-Alcohols. *Tetrahedron* **29**, 4183-4187 (1973).

8. Tangirala, R. S.; Dixon, R.; Yang, D.; Ambrus, A.; Antony, S.; Agama, K.; Pommier, Y.; Curran, D. P. Total and Semisynthesis and in vitro Studies of Both Enantiomers of 20-Fluorocamptothecin. *Bioorg. Med. Chem. Lett.* **15**, 4736-4740 (2005).

9. Ratsch, M., Ye, C., Yang, Y., Zhang, A., Evans, A. M., Börjesson, K. All-carbon-linked Continuous Three-dimensional Porous Aromatic Framework Films with Nanometer-precise Controllable Thickness. *J. Am. Chem. Soc*. **142**, 6548–6553 (2020).

10. Mangunuru, H. P. R., Malapit, C. A., Haddad, N., Reeves, J. T., Qu, B., Rodriguez, S., Lee, H., Yee, N. K., Song, J. J., Busacca, C. A., Senanayake, C. H. Enantioselective Arylation of Oxindoles Using Modified bi-dime Ligands. *Synthesis* **50**, 4435–4443 (2018).

11. Bauer, G., Wodrich, M. D., Scopelliti, R., Hu, X. Iron Pincer Complexes as Catalysts and Intermediates in Alkyl–aryl Kumada Coupling Reactions. *Organometallics* **34**, 289–298 (2014).

12. Uemura, M., Yorimitsu, H., Oshima, K. Synthesis of $Cp^{*}CH_2PPh_2$ and its use as a Ligand for the Nickel-catalysed Cross-coupling Reaction of Alkyl Halides with Aryl Grignard Reagents. *Chem. Commun.* 4726 (2006).

13. Burns, M. J., Fairlamb, I. J., Kapdi, A. R., Sehnal, P., Taylor, R. J. Simple Palladium(II) Precatalyst for Suzuki−Miyaura Couplings: Efficient Reactions of Benzylic, Aryl, Heteroaryl, and Vinyl Coupling Partners. *Org. Lett.* **9**, 5397–5400 (2007).
14. Pearson, D. M., Conley, N. R., Waymouth, R. M. Palladium-catalyzed Carbonylation of Diols to Cyclic Carbonates. *Adv. Synth. Catal.* **353**, 3007–3013 (2011).
15. Yang, C.-T., Zhang, Z.-Q., Liang, J., Liu, J.-H., Lu, X.-Y., Chen, H.-H., Liu, L. Copper-catalyzed Cross-coupling of Nonactivated Secondary Alkyl Halides and Tosylates with Secondary Alkyl Grignard Reagents. *J. Am. Chem. Soc.* **134**, 11124–11127 (2012).
16. Balaraman, K., Wolf, C. Palladium and Nickel Catalyzed Suzuki Cross-Coupling with Alkyl Fluorides. *Org. Lett.* **23**, 8994–8999 (2021).
17. Shinohara, R., Ogawa, N., Kawashima, H., Wada, K., Saito, S., Yamazaki, T., Kobayashi, Y. Sn2 Reaction of Diarylmethyl Anions at Secondary Alkyl and Cycloalkyl Carbons. *Eur. J. Org. Chem.* **2019**, 1461–1478 (2019).
18. Saito, T., Nishimoto, Y., Yasuda, M., Baba, A. Direct Coupling Reaction Between Alcohols and Silyl Compounds: Enhancement of Lewis Acidity of Me_3SiBr Using $InCl_3$. *Chem. Inform.* **38** (2007).
19. Yin, X., Zheng, L., Mohammadlou, A., Cagnon, B. R., Wulff, W. D. Resolution of Vaulted Biaryl Ligands via Borate Esters of Quinine and Quinidine. *J. Org. Chem.* **85**, 10432–10450 (2020).
20. Kawatsura, M., Hartwig, J. F. Simple, Highly Active Palladium Catalysts for Ketone and Malonate Arylation: Dissecting the Importance of Chelation and Steric Hindrance. *J. Am. Chem. Soc.* **121**, 1473–1478 (1999).
21. Chantal, L. Process for Preparing a Poly(aryl ether ketone) Using a High Purity 4,4'-difluorobenzophenone. US2011213115A1, September 1, 2011.
22. Zhong, Y., Han, W. Iron-catalyzed Carbonylative Suzuki Reactions Under Atmospheric Pressure of Carbon Monoxide. *Chem. Commun.* **50**, 3874–3877 (2014).
23. Zhao, H., Han, W. Ligand-free Palladium-catalyzed Oxidative Carbonylative Homocoupling of Arylboron Reagents at Ambient Pressure. *Eur. J. Org. Chem.* **2016**, 4279–4283 (2016).
24. Hayakawa, M., Aoyama, T., Nakaoka, K., Kosuge, M., Ouchi, A. Integration of a Four-step Reaction into One-pot Under the Coexistence of Silica-gel-supported Acid and Base Reagents:

Synthesis of Benzo- and Naphthothiophenes Using $NaHSO_4/SiO_2$ and Na_2CO_3/SiO_2. *Synthesis*. **51**, 2572–2578 (2019).
25. Shang, R., Fu, Y., Wang, Y., Hu, Q., Yu, H.-Z., Liu, L. Copper-catalyzed Decarboxylative Cross-coupling of Potassium Polyfluorobenzoates with Aryl Iodides and Bromides. *Angew. Chem. Int. Ed.* **48**, 9350–9354 (2009).
26. Hwang, H.-S., Joo, S.-R., Kim, S.-H. Adamantylzinc Bromides: Direct Preparation and Application to Cross-coupling Reactions. *B. Korean Chem. Soc.* **36**, 2769–2772 (2015).
27. Balaraman, K., Wolf, C. Chemodivergent C_{sp3}-F Bond Functionalization and Cross-Electrophile Alkyl-Alkyl Coupling with Alkyl Fluorides. *Science Adv.* **8**, eabn7819 (2022).
